# Some Gram-negative Lipoproteins Keep Their Surface Topology When Transplanted from One Species to Another and Deliver Foreign Polypeptides to the Bacterial Surface*[Fn FN2]

**DOI:** 10.1074/mcp.M116.065094

**Published:** 2017-05-08

**Authors:** Laura Fantappiè, Carmela Irene, Micaela De Santis, Alessandro Armini, Assunta Gagliardi, Michele Tomasi, Matteo Parri, Valeria Cafardi, Serena Bonomi, Luisa Ganfini, Francesca Zerbini, Ilaria Zanella, Chiara Carnemolla, Luca Bini, Alberto Grandi, Guido Grandi

**Affiliations:** From the ‡Synthetic and Structural Vaccinology Unit, CIBIO, University of Trento, Via Sommarive, 9, 38123 Povo, Trento, Italy;; §Functional Proteomics Lab., Department of Life Sciences, University of Siena, Via Aldo Moro 2, 53100 Siena, Italy;; ¶Toscana Life Sciences Scientific Park, Via Fiorentina, 1 53100, Siena, Italy

## Abstract

In Gram-negative bacteria, outer membrane-associated lipoproteins can either face the periplasm or protrude out of the bacterial surface. The mechanisms involved in lipoprotein transport through the outer membrane are not fully elucidated. Some lipoproteins reach the surface by using species-specific transport machinery. By contrast, a still poorly characterized group of lipoproteins appears to always cross the outer membrane, even when transplanted from one organism to another. To investigate such lipoproteins, we tested the expression and compartmentalization in *E. coli* of three surface-exposed lipoproteins, two from *Neisseria meningitidis* (Nm-fHbp and NHBA) and one from *Aggregatibacter actinomycetemcomitans* (Aa-fHbp). We found that all three lipoproteins were lipidated and compartmentalized in the *E. coli* outer membrane and in outer membrane vesicles. Furthermore, fluorescent antibody cell sorting analysis, proteolytic surface shaving, and confocal microscopy revealed that all three proteins were also exposed on the surface of the outer membrane. Removal or substitution of the first four amino acids following the lipidated cysteine residue and extensive deletions of the C-terminal regions in Nm-fHbp did not prevent the protein from reaching the surface of the outer membrane. Heterologous polypeptides, fused to the C termini of Nm-fHbp and NHBA, were efficiently transported to the *E. coli* cell surface and compartmentalized in outer membrane vesicles, demonstrating that these lipoproteins can be exploited in biotechnological applications requiring Gram-negative bacterial surface display of foreign polypeptides.

Bacterial lipoproteins are a class of membrane-anchored proteins which play key roles in bacterial physiology and pathogenesis. In Gram-positive bacteria, lipoproteins cross the membrane and remain attached on its surface through their lipid chains, whereas in Gram-negative bacteria they can be found in three different cellular compartments: (1) attached to the periplasmic side of the inner membrane (IM), (2) attached to the periplasmic side of the outer membrane (OM)^1^, and (3) exposed on the surface of the outer membrane.

The mechanisms involved in lipoprotein biosynthesis, as well as their localization either on the inner membrane, or transported to the periplasmic side of the outer membrane, have been elucidated (1–5). However, the mechanisms that determine whether a lipoprotein remains attached to the inner leaflet of the OM, or is presented on the bacterial surface, are still not well characterized.

In general, surface-exposed lipoproteins can be tentatively divided into two groups. The first group of lipoproteins reaches the bacterial surface through dedicated transport machinery, including Type II Secretion System (T2SS) (6), Type V Secretion System (T5SS) (7, 8), the beta-barrel assembly machine (Bam) complex (9–13), and species-specific flippases (14). As far as the flippases are concerned, their existence has been postulated by a few authors (15, 16) and very recently experimentally supported by Hooda and coworkers. These authors reported that the inactivation of the nmb0313 gene, which encodes for surface lipoprotein assembly modulator 1, (*slam1*) in *Neisseria menigitidis* impairs the surface exposition of three lipoproteins, the transferring binding protein (TbpB), the lactoferrin binding protein (LbpB), and the factor H binding protein (fHbp) (17). The authors also showed that the same proteins can reach the surface of *E. coli* only when coexpressed with Slam1. The second group of surface-exposed lipoproteins includes those that move from the cytoplasm to the surface even when transplanted from one Gram-negative species to another. Reports describing these lipoproteins are limited to few examples. For instance, *Salmonella enterica* YaiW and *Vibrio cholerae* VolA (Vibrio outer membrane lysophospholipase) surface lipoproteins were shown to maintain their surface location when expressed in *E. coli* (18, 19). Furthermore, in contrast to what published by Hooda and coworkers (17), Konar *et al*. had previously shown that in *E. coli* the *N. meningitidis* fHbp spontaneously reached the cell surface in a structurally functional conformation, as demonstrated by its capacity to bind Factor H (20).

The scope of the present work is 2-fold. First, we intend to provide additional evidence on the existence of this second group of lipoproteins that spontaneously reach the surface of Gram-negative bacteria. We have investigated the transport of the *N. meningitidis* factor H binding protein (from here on named Nm-fHbp) in *E. coli*, to understand whether it reaches the cell surface, as shown by Konar *et al*., or it strictly requires the Slam1 protein, as reported by Hooda and coworkers. We have also used *E. coli* to study two other surface-exposed lipoproteins, the *Neisseria* heparin binding protein (NHBA) and the *Aggregatibacter actinomycetemcomitans* factor H binding protein (from here on named Aa-fHbp). We show that, similarly to what occurs in their natural hosts, all three proteins are exposed on the surface of *E. coli*, supporting the evidence that the final destiny of some lipoproteins is built in their structure and that their transport machinery is conserved among some Gram-negative species. Second, we want to investigate whether lipoproteins, which spontaneously cross the outer membrane can be exploited to deliver heterologous polypeptides to the bacterial surface. The first reports on bacterial display of recombinant proteins appeared in the middle of the 1980s and it was soon anticipated that this strategy could be exploited for the surface display of antigens and enzymes. At present, surface exposition of heterologous polypeptides in Gram-negative bacteria is achieved by creating genetic fusions with several “chaperone” proteins. These include autotransporters belonging to T5SS (21), ice nucleation proteins (22), and truncated forms of integral outer membrane proteins (23–25). The efficiency with which chaperone proteins deliver their passengers to the bacterial surface varies from one polypeptide to another. Therefore, the identification of novel transport systems could be advantageous for biotechnological applications. We show that *E. coli* can be efficiently decorated with foreign antigens by simply fusing them to the C termini of lipoproteins, which spontaneously cross the outer membrane, thus expanding the armamentarium of transporters available for the optimal surface exposition of foreign polypeptides.

## MATERIALS AND METHODS

### 

#### 

##### Bacterial Strains and Culture Conditions

The list of bacterial strains described in this work are reported in supplemental Table S2. *E. coli* strains HK100 and BL21 were routinely grown in LB broth at 37 °C and used for cloning and expression experiments, respectively. BL21Δ*ompA* strain was generated as previously described (26). Stock preparations of strains in LB + 15% glycerol were stored at −80 °C. Each bacterial manipulation was started using an overnight culture from a frozen stock. When required ampicillin or chloramphenicol were added to final concentration of 100 μg/ml and 30 μg/ml, respectively.

##### Construction of Plasmids

The polymerase incomplete primer extension (PIPE) cloning method (27) was used for plasmid construction. Nm-*fHbp* and *nhba* genes were amplified by PCR from MC58 *Neisseria meningitidis* serogroup B genome using primers fHbp-F/fHbp-R and NHBA-F/NHBA-R (supplemental Table S3), respectively. fHbp-F and NHBA-F primers were designed to amplify the genes with their natural leader sequence and lipobox. The plasmid pET21b^+^ used in the PIPE reaction was amplified using the pET-R and pET-ΔHIS-F primers (supplemental Table S3). Finally, the PCR products were mixed together and used to transform *E. coli* competent cells, obtaining pET21_Nm-fHbp and pET21_NHBA plasmids. pET21_Nm-(ΔGly)fHbp, carrying the deletion of four glycine residues downstream of the lipobox, and pET21_Nm-(Ala_4_) fHbp, bearing the substitution of the same residues were engineered by site-directed mutagenesis using the two sets of primers: fHbpΔgly-F/fHbpΔgly-R and fHbpGly>ALAF/fHbpGly>ALA R, respectively (supplemental Table S3). pET21_Nm-fHbp(ΔDomB/C) and pET21_Nm-fHbp (ΔDomC) plasmids were generated amplifying pET21_Nm-fHbp plasmid using the following primer pairs: pET-ΔHIS-F/fHbpΔdomB/domC-R, pET-ΔHIS-F/fHbpΔdomC-R. Following PCR amplification, the linear DNA molecule was circularized by PIPE. pET21_fHbp_C>A and pET21_NHBA_C>A were generated using the primers: FHBP C>A-R/FHBPC>A-F and NHBAC>A-R/NHBAC>A-F. The forward primers bear a GC mismatch allowing the substitution of the cysteine at position +1 with an alanine residue. The identity of each construct was verified by DNA sequencing. To generate the pACYC-slam1 plasmid, *slam1* gene (*nmb0313*) was PCR amplified from MC58 *Neisseria meningitidis* serogroup B genome using slam-F/slam-R primers (supplemental Table S3). The plasmid pACYC was amplified using pacyc-F/pacyc-R primers (supplemental Table S3). Finally, the PCR products were mixed together and used to transform *E. coli* competent cells, obtaining pACYC-slam1 plasmid. The correctness of the cloning was verified by sequence analysis using primers pacyc-up1/pacyc-down1. To coexpress Slam1 with either Nm-fHbp or NHBA, electro-competent cells of BL21Δ*ompA*, BL21, and BL21C43 strains carrying either pET21_Nm-fHbp or pET21_NHBA plasmid were transformed with pACYC-slam1 and colonies were selected on LB agar plates supplemented with 100 μg/ml ampicillin and 30 μg/ml chloramphenicol.

The 828bp Aa-fHbp gene (EnsemblBacteria gene ID HMPREF9996_00541) from *A. actinomycetemcomitans Y4* was synthetized as dsDNA by GeneArt™ (Thermo Fisher Scientific) (the wild-type GTG start codon was substituted with ATG start codon). The synthetic gene was cloned into pET21b^+^ plasmid fused to an 8-HIS-tag at the C terminus for subsequent detection of the protein using an anti-HIS-tag polyclonal antibodies. To this aim, the synthetic DNA was amplified by PCR using AgfHbp_F and AgfHbp_R primers. The pET21b^+^ plasmid backbone was amplified with pETHIS-F and pET2-R primers (supplemental Table S3). To clone three copies of the EGFRvIII peptide as translational fusion to the C terminus of Nm-fHbp and NHBA proteins, a DNA fragment, named vIIIx3, coding for three copies of EGFRvIII (LEEKKGNYVVTDH), each separated by Gly-Ser, was amplified from pUC-vIII using the primer couples vIII-F/vIII-R (for Nm-fHbp fusion) and NHBA-vIII-F1/NHBA-vIII-R1 (for NHBA fusion). pUC-vIII (Geneart) is a pUC derivative carrying a synthetic DNA encoding the three of EGRFvIII epitope. In parallel pET21-Nm-fHbp and pET21-NHBA plasmids were PCR amplified using the primer couples nohisflag/fHbpR2 and NHBA-vIII-F/NHBA-vIII-R, respectively (supplemental Table S3). Finally, the appropriate PCR products were mixed together and used to transform *E. coli* competent cells, obtaining pET-Nm-fHbp-vIII and pET-NHBA-vIII plasmids. To fuse five copies of the MUC1 peptide (GVTSAPDTRPAPGSTAPPAH) to the C terminus of Nm-fHbp domain A DNA fragment, named MUC1X5, coding for five copies of MUC1 peptide was constructed by assembling ten oligonucleotides (supplemental Table S3) with short overlapping sequence by polymerase cycling assembly. Subsequently the entire fragment was PCR amplified with primers RMUCFH and FMUCDomA. In parallel, pET21_Nm-fHbp(ΔDomB/ΔDomC) plasmid was linearized by PCR amplification using primers FHBP-F1 and FHBPDA-R (supplemental Table S3). Finally, the linearized plasmid and the MUC1X5 fragment were mixed together and used to transform in *E. coli* competent cells, generating pET-Nm-fHbpDomA-MUC1 plasmid.

##### Bacterial Total Lysate and OMV Preparation

Plasmids containing the genes of interest were used to transform BL21Δ*ompA* strain. Recombinant clones were grown in 200 ml LB medium (starting OD_600_ = 0.05) and, when the cultures reached an OD_600_ value of 0.5, protein expression was induced by addition of 1 mm IPTG. After 2 h, outer membrane vesicles (OMVs) were collected from culture supernatants by filtration through a 0.22 μm pore size filter (Millipore) followed by high-speed centrifugation (200,000 × *g* for 2 h). Pellets containing OMVs were finally resuspended in 1× PBS. Total bacterial lysates were prepared by suspending bacterial cells from 1 ml cultures (centrifuged at 13,000 × *g* for 5 min) in sodium dodecyl sulfate-polyacrylamide gel electrophoresis (SDS-PAGE) Laemli buffer (Bio-Rad, Hercules, CA) and heated at 100 °C for 5 min. Proteins were separated by 4–12% or 10% SDS-PAGE gel (Invitrogen, Carlsbad, CA), run in MES buffer (Invitrogen) and finally stained with Coomassie Blue.

##### Western Blotting Analysis

Total lysates were prepared from bacteria grown in LB broth. Liquid cultures were pelleted in a bench-top centrifuge and suspended in SDS-PAGE loading buffer in an appropriate volume to normalize cell density to a final OD_600_ of 10. Each sample (10 μl) was then separated on a 4–12% SDS-PAGE gel. Proteins separated on polyacrylamide gels were then transferred onto nitrocellulose membranes by standard methods. The filters were blocked either 1 h at room temperature or overnight at 4 °C by agitation in blocking solution (10% skimmed milk and 0.05% Tween in PBS). Primary antibodies or sera were diluted in 3% skimmed milk containing 0.05% Tween in PBS and incubated one hour at room temperature. The polyclonal antibodies against Nm-fHbp and NHBA were obtained from Genscript by immunizing rabbits with specific synthetic peptides (SVRKNEKLKLAAQGC for Nm-fHbp and CGSKSVDGIIDSGDD for NHBA) conjugated with KLH protein. Anti-MBP (maltose binding protein) monoclonal antibody and anti-HisTAG antibodies were purchased from New England Biolabs (Ipswich, MA) and Roche (Basel, Switzerland), respectively. After three washing steps in PBS containing 0.05% Tween, the filters were incubated in a 1:2000 dilution of peroxidase-conjugated anti-rabbit or anti-mouse immunoglobulin (Dako) in 3% skimmed milk and 0.05% Tween in PBS for one hour, and after three washing steps, antibody binding was detected by using the SuperSignal West Pico chemiluminescent substrate (Pierce, Waltham, MA).

##### Proteinase K Protection Assay

To investigate proteins localization in bacterial cells, strains expressing Nm-fHbp, Aa-fHbp and NHBA proteins were grown in LB medium supplemented with 100 μg/ml Ampicillin. When the cultures had reached an OD_600_ value of 0.5, protein expression was induced by addition of 1 mm IPTG. After 2 h bacteria were harvested by centrifugation at 3500 × *g* for 10 min at 4 °C and washed three times in PBS. Cells were resuspended in PBS to a final density of 2 × 10^9^ cells/ml. Proteinase K (Fermentas) was added to a final concentration of 100 μg/ml. After incubation for 30 min at 37 °C the reaction was stopped by addition of 5 μl of the peptidase inhibitor, phenylmethhylsulfonyl fluoride (PMSF; Sigma-Aldrich). The suspension was centrifuged at 9000 × *g* for 5 min and pellets were resuspended in SDS-PAGE loading buffer and boiled for 5 min. Samples were loaded on a 4–12% polyacrylamide gel and Western blotting analysis was performed using specific antibodies. MBP protein was used as a control of the integrity of the bacterial cells after protease treatment.

To investigate protein localization in OMVs 10 μg of proteinase K (Fermentas, Waltham, MA) were added to 15 μg of intact or solubilized (in 1% SDS) OMVs purified from strains expressing Nm-fHbp, Aa-fHbp or NHBA proteins, and the mixtures were then incubated at 37 °C for 60 min. After proteinase K inactivation with 10 mm phenylmethylsulfonyl fluoride (PMSF; Sigma Aldrich) samples were loaded on a 4–12% or 10% polyacrylamide gel and Western blotting analysis was performed as previously described. MBP protein was used as a control of the integrity of the OMVs after protease treatment.

##### Quality Control of OMV Preparations by Mass Spectrometry Analysis

##### A) Experimental Design and Statistical Rationale

To demonstrate that the OMV purification protocol described above consistently provided high quality vesicles not contaminated with cytoplasmic and/or inner membrane proteins, OMVs were prepared three times from the supernatants of BL21Δ*ompA* strain. The three OMV preparations were analyzed by 1D SDS-PAGE and the similarity of the protein profiles assessed by comparison of the Coomassie Blue-stained protein bands of the three samples. Subsequently, one OMV preparation was analyzed twice by two-dimensional (2D) electrophoresis (see below) to verify the consistency of the two-dimensional protein separation. Finally, protein species separated by 2D and consistently present in all technical replicates were identified by mass fingerprinting (PMF). The parameter used to accept identifications was the default Mascot protein score greater than 56, corresponding to a 5% probability that the observed match was a random event (*p* < 0.05). The number of matched peptides and the extend of sequence coverage were also considered to confirm protein ID.

##### B) Two-dimensional Gel Electrophoresis (2-DE)

OMV samples were resuspended in denaturing buffer containing 7 m Urea, 2 m Thiourea, 4% (w/v) CHAPS, 1% (w/v) DTE, and 2% (v/v) TritonX100, and then precipitated in cold acetone (1:4) overnight at −20 °C. After centrifugation at 15,000 × *g* at 4 °C for 15 min protein pellets were resuspended in 350 μl of denaturation buffer and 0.2% (v/v) carrier ampholyte in the case of analytical gels or and 0.2% (v/v) carrier ampholyte in the case of analytical and Western blotting-preparative gels or 2% (v/v) carrier ampholyte for MS-preparative gels, and they were separated by 2-DE. 2-DE was performed using the Immobiline-polyacrylamide system, as previously described (28, 29). IEF was carried out on nonlinear wide-range immobilized pH gradients (IPG) (pH 3–10; 18-cm-long IPG strips; GE Healthcare, Uppsala, Sweden), using the Ettan™ IPGphor system (GE Healthcare). Strips for analytical runs were rehydrated with protein sample for 1 h at 0 V and for 8 h at 30 V, at 16 °C. Strips were then focused setting the following voltage steps at 16 °C: 200 V for 1 h, from 300 V to 3500 V in 30 min, 3500 V for 3 h, from 3500 V to 8000 V in 30 min, 8000 V for 3 h, 10,000 V until a total of 80,000 Vh was reached. MS-preparative strip was rehydrated with protein sample for 1 h at 0 V and overnight at 30 V, at 16 °C. IEF was achieved, at 16 °C, applying sequentially: 200 V for 8 h, from 200 V to 3500 V in 2 h, 3500 V for 2 h, from 3500 V to 5000 V in 2 h, 5000 V for 3 h, from 5000 V to 8000 V in 1 h, 8000 V for 1 h, from 8000 V to 10,000 V in 1 h, 10,000 V until a total of 100,000 Vh was reached. After IEF, strips were equilibrated for 12 min in 6 m urea, 30% (v/v) glycerol, 2% (w/v) SDS, 0.05 m Tris-HCl pH 6.8, 2% (w/v) DTE; and then for further 5 min in 6 m urea, 30% (v/v) glycerol, 2% (w/v) SDS, 0.05 m Tris-HCl pH 6.8, 2.5% (w/v) iodoacetamide, and bromphenol blue in trace.

SDS-PAGE was carried out, at 10 °C, on house-made 9–16% polyacrylamide linear gradient gels (18 cm × 20 cm × 1.5 mm) at 40 mA/gel constant current, until the dye front reached the bottom of the gel. Analytical gels and MS-preparative gels were stained with ammoniacal silver nitrate (30, 31) and MS-compatible silver staining (32), respectively, and they were scanned using the ImageScanner III (GE Healthcare). 2-DE gel images were analyzed with ImageMaster 2D Platinum v. 6 software (GE Healthcare).

##### C) Protein Identification by Mass Spectrometry

Protein identification was carried out by peptide mass fingerprinting (PMF) (33, 34). Spots of interest were manually excised from silver stained MS-preparative gels, destained, as previously described (35), and acetonitrile dehydrated. After spot rehydration in trypsin solution (Sigma Aldrich), in-gel protein digestion was performed by an overnight incubation at 37 °C. 1.25 μl of each protein digestion was directly spotted onto the MALDI target and air-dried. Then, 0.75 μl of matrix solution (a solution of 5 mg/ml alpha-cyano-4-hydroxycynnamic acid in 50% v/v acetonitrile and 0.5% v/v trifluoroacetic acid) was added to the dried samples and allowed to dry again. Mass spectra were acquired using an Ultraflex III MALDI-TOF/TOF mass spectrometer (Bruker Daltonics, Billerica, MA), equipped with a 200 Hz smartbeam™ I laser, in reflector positive mode with a laser frequency set to 100 Hz. Spectra were analyzed by Flex Analysis software v.3.0. Calibration of acquired spectra was provided using, as internal standard, the 842.509 and 2211.105 *m*/*z* peptides originating from trypsin autoproteolysis. The resulting mass lists were filtered for contaminant removal: mass matrix-related ions, trypsin auto-lysis and keratin peaks. The raw MS data files have been deposited to the ProteomeXchange Consortium (http://proteomecentral.proteomexchange.org) via the PRIDE partner repository (http://www.ebi.ac.uk/pride/archive/) with the data set identifier PXD005732.

PMF searching was carried out in Swiss-Prot/TrEMBL database (version 2016_07; 551705 sequences; 197114987 residues) set for *Escherichia coli* using Mascot (Matrix Science Ltd., London, UK, http://www.matrixscience.com) on-line available software. The experimental and theoretical peptide fingerprinting patterns Δmass was less than 100 ppm, and trypsin was selected as the digestion enzyme with one allowed missed cleavage. Alkylation of cysteine by carbamidomethylation was assumed as fixed modification, whereas oxidation as possible modification. Mascot output files have been deposited to the ProteomeXchange Consortium via the PRIDE partner repository (see above).

##### Fluorescence-Activated Cell Sorting (FACS) Analysis

Twenty milliliters of LB medium, containing the appropriate antibiotic, was inoculated at OD_600_ = 0.05 starting from an overnight culture of each transformant. Cultures were then grown until OD_600_ = 0.5 (2.5 × 10^8^ CFU/ml) and expression of the proteins was induced by addition of 1 mm IPTG and further incubation for 2 h. BL21Δ*ompA E. coli* strain transformed with pET21b^+^ empty vector was used as negative control. Bacterial cells from 1 ml were harvested by centrifugation at 10,000 × *g* for 5 min at 4 °C and resuspended in PBS + 1% BSA dilution buffer to obtain 2 × 10^7^ CFU/ml cells. Aliquots of 50 μl were then dispensed in a round bottom 96-well plate.

Primary antibodies against proteins of interest were diluted at 10 μg/ml and 5 μl of each dilution was added in the wells containing bacteria suspension and incubated 1 h on ice. For antibody binding competition experiment, 1 or 10 μg/ml of the fHbp-derived peptide*_35–50_* or the NHBA-derived peptide*_408–421_* used for the preparation of anti-fHbp and anti-NHBA antibodies were added in the wells. Each well was then washed twice with 200 μl PBS + 1% BSA buffer. Subsequently, 20 μl of commercial Alexa Fluor® 488 labeled anti-rabbit secondary antibodies diluted 1:200 in dilution buffer were added in each well and incubated 1 h on ice. Each well was then washed twice with 200 μl PBS + 1% BSA buffer and the plate was centrifuged at 4000 × *g* for 5 min. Samples were then resuspended in 2% formaldehyde solution, incubated 15 min at 4 °C and then centrifuged at 4000 × *g* for 5 min. Samples were resuspended in 130 μl of PBS and data were acquired by using BD FACS Canto II cell analyzer.

##### Confocal Microscopy Analysis

To verify Nm-fHbp and NHBA localization on the cell surface, 20 ml of LB medium was inoculated at OD_600_ = 0.05 starting from an overnight culture of each recombinant strain. Cultures were grown until the OD_600_ = 0.5 (2.5 × 10^8^ CFU/ml) and proteins expression was induced by addition of 1 mm IPTG and further incubation for 2 h at 37°C. Bacterial cells from 1 ml were harvested by centrifugation at 6000 × *g* for 5 min at 4 °C and resuspended in 2% formaldehyde solution, incubated 15 min at 4 °C, and then centrifuged at 6000 × *g* for 5 min. Samples used for intracellular analyses were then incubated with 0.1% Brj96 for 5 min at room temperature. Bacteria were washed three times with 1 ml PBS, suspended in 1 ml of blocking buffer (PBS containing 0.1% BSA and 10% normal goat serum), and incubated 20 min at room temperature.

Primary antibodies against proteins of interest were diluted in PBS containing 0.1% BSA at 10 μg/ml and 100 μl of each dilution was added in each bacteria suspension and incubated 1 h at room temperature. After two washes with PBS-0.1% BSA, bacteria were incubated for 20 min at room temperature with secondary antibodies, either goat anti-mouse Alexa Fluor® 488 or goat anti-rabbit-Alexa Fluor® 594 conjugated anti-IgG (Molecular Probes, Eugene, OR) at 1:400 final dilution. Labeled bacteria were washed twice with PBS supplemented with 0.1% BSA, and allowed to adhere to polylysine slides (Thermo Scientific) for 20 min at room temperature. Slides were mounted with ProLong Gold antifade reagent (Thermo Scientific). Confocal microscopy analysis was performed with a Leica SP5 microscope and images were obtained using Leica LASAF.

##### Triton X-114 Protein Separation from OMVs

Protein lipidation was assessed by phase partitioning with Triton X-114 (36). One hundred micrograms of OMVs (10–15 μl) were diluted in 450 μl of PBS, then ice cold 10% TritonX-114 was added to 1% final concentration and the OMV-containing solution was incubated at 4 °C for 1 h under shaking. The solution was then heated at 37 °C for 10 min and the aqueous phase was separated from the detergent by centrifugation at 13,000 × *g* for 10 min. Proteins in both phases were then precipitated by standard chloroform/methanol procedure, separated by SDS-PAGE electrophoresis and the protein of interest visualized by Western blotting.

##### Bioinformatics Analysis

Nm-fHbp var.1 and Aa-fHbp sequence alignments were performed with clustalx 2.1 complete alignment. The identity percentage among the two sequence was calculated by blast protein/protein: http://blast.ncbi.nlm.nih.gov/Blast.cgi.

## RESULTS

### 

#### 

##### Selection of N. meningitidis Lipoproteins

Nm-fHbp and NHBA are two *N. meningitidis* surface-exposed lipoproteins that play important roles in pathogenesis. Nm-fHbp enables the bacteria to evade the complement system (37), whereas NHBA carries an arginine-rich region responsible for heparin binding, which correlates with an increased survival of *N. meningitidis* in human serum. Structural studies have shown that the three genetic variants of Nm-fHbp (Nm-fHbp var.1, var.2 and var.3) (38) are organized in three domains, called A, B, C (39). Domain A encompasses amino acids 27 to 119, Domain B starts from amino acid 120 and ends at residue 183, and finally Domain C spans from amino acid 184 to the end (amino acid 274). The protein structure consists of an eight-stranded antiparallel β-barrel (157–274) topped by a single α-helix (157–160) and a flexible linker (120–156), which connects Domain A to Domain B (39). The Factor H binding site is in Domain A and it has been shown that a single amino acid replacement (R41S) is sufficient to impair Factor H binding (40).

NHBA fold consists of an eight-stranded β-barrel that closely resembles the C-terminal domain of Nm-fHbp suggesting that the two proteins derive from a common ancestor (41).

Consistent with their biological role, Nm-fHbp and NHBA extend out of the bacterial surface and are accessible to antibodies with bactericidal activity. For this reason, the two proteins have been selected to investigate their localization when expressed in *E. coli*.

##### Nm-fHbp and NHBA Prolipoproteins are Expressed in E. coli and Cross the Inner Membrane

**Fig. 1.**
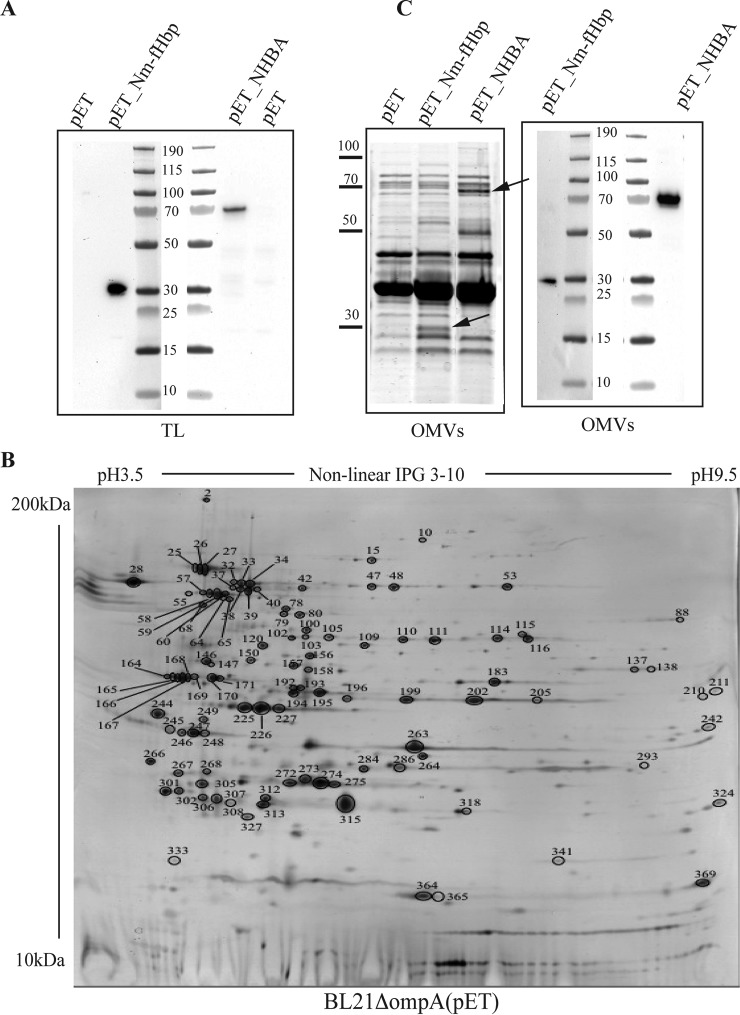
**Expression and OMV compartmentalization of Nm-fHbp and NHBA in *E. coli* BL21 Δ*ompA* strain.** *A*, *E. coli* BL21Δ*ompA* (pET), BL21Δ*ompA* (pET_Nm-fHbp) expressing fHbp and BL21Δ*ompA* (pET_NHBA) expressing NHBA were grown in LB at 37 °C. At OD_600_ = 0.5, 1 mm IPTG was added and after 2 h bacterial cells were collected by centrifugation. Twenty-five micrograms of proteins from bacterial cell extracts were separated by SDS-PAGE and transferred to nitrocellulose filters for Western blotting analysis. Detection of Nm-fHbp and NHBA was carried out using rabbit antibodies raised against 15 amino acids long synthetic peptides corresponding to Nm-fHbp and NHBA specific sequences (see Materials and Methods). *B*, 2D gel electrophoresis of OMVs from BL21Δ*ompA*(pET) strain. 150 μg of OMV proteins were first focused on a nonlinear pH 3–10 gradient and then separated on a 9–16% SDS-polyacrylamide gel. The gel was stained with ammoniacal silver nitrate and with a MS-compatible silver staining. The protein spots identified by MALDI TOF-TOF Mass Spectrometry are indicated (see supplemental Table S1). *C*, *E. coli* BL21Δ*ompA*(pET), BL21Δ*omp*(pET_Nm-fHbp) and BL21Δ*ompA*(pET_NHBA) strain were grown in LB at 37 °C. At OD_600_ = 0.5, 1 mm IPTG was added and after 2 h, OMVs were purified from culture supernatants by ultrafiltration. Each OMV preparation (25 μg) were separated by SDS-PAGE PAGE and either stained with Coomassie blue (left panel) or transferred to nitrocellulose membrane for Western blotting analysis (right panel). Detection of Nm-fHbp and NHBA was carried our using rabbit anti-Nm-fHbp and anti-NHBA antibodies.

Nm-*fHbp* var.1 (from here on indicated as Nm-fHbp) and *nhba* genes encoding full-length proliproproteins from *N. meningitidis* strain MC58 were inserted into pET21b^+^ plasmid and the derived pET21_Nm-fHbp and pET21_NHBA plasmids were used to transform *E. coli* BL21Δ*ompA* strain, producing BL21Δ*ompA*(pET21_Nm-fHbp) and BL21Δ*ompA*(pET21_NHBA) recombinant strains. As shown in the Western blotting reported in Fig. 1*A*, both proteins were expressed in *E. coli* total cell extracts.

We next asked whether Nm-fHbp and NHBA proteins could cross the inner membrane of *E. coli*. We used the BL21Δ*ompA* strain to address this question because of its ability to release abundant quantities of OMVs in the culture supernatant (26). Because OMVs are composed of OM and periplasmic proteins (42), we followed the compartmentalization of Nm-fHbp and NHBA into the OMVs to indirectly assess the capacity of the two proteins to pass through the inner membrane.

As previously shown for OMVs from other hyper-vesiculating strains (43, 44), we first verified that BL21Δ*ompA*–derived OMVs were only constituted by OM and periplasmic proteins, thus excluding contamination from cytoplasmic proteins. OMV proteins were purified by ultracentrifugation (see Materials and Methods), separated by 2D gel electrophoresis (Fig. 1*B*), and protein spots were picked and characterized by MALDI-TOF-TOF Mass Spectrometry. A total of 114 protein species were unequivocally identified, corresponding to 70 unique *E. coli* proteins. Importantly, most of them (65 proteins) were outer membrane-associated and periplasmic proteins, as reported in the UniProtKB annotation (supplemental Table S1). Of the remaining five proteins, three are annotated as inner membrane proteins and two as cytoplasmic proteins. The presence of the inner membrane proteins in OMVs could be because of either partial hydrolysis by periplasmic proteases or incorrect annotation. Notably, two of them, New lipoprotein 1 (NlpI) and YajG (unknown function), do not carry the canonical aspartic acid at position +2, which is the main inner membrane retention signal (4, 5). Furthermore, NlpI has been shown to be associated to the outer membrane and to be a virulence factor involved in the interaction with the human brain microvascular endothelial cells (45). Of the two remaining proteins annotated as cytoplasmic, one is Elongation Factor 2, a protein that is constantly found in the membrane compartments of both Gram-negative and Gram-positive bacteria (44, 46).

Having confirmed the quality of BL21Δ*ompA*-derived vesicles, OMVs were purified from the supernatants of BL21Δ*ompA*(pET21_Nm-fHbp) and BL21Δ*ompA*(pET21_NHBA) strains and the OMV-containing pellets were analyzed by SDS-PAGE. As shown in Fig. 1*C*, protein species with apparent molecular masses expected for Nm-fHbp and NHBA were visible in the OMVs from BL21Δ*ompA*(pET21_Nm-fHbp) and BL21Δ*ompA*(pET21_NHBA) strains, respectively. The identity of the two-protein species was confirmed by Western blotting using anti-Nm-fHbp and anti-NHBA specific polyclonal antibodies (Fig. 1*C*).

Fig. 1*C* also demonstrated that the protein content of the OMV preparations from BL21Δ*ompA*, BL21Δ*ompA*(pET21_Nm-fHbp), and BL21Δ*ompA*(pET21_NHBA) is almost identical, both qualitatively and quantitatively, except for the expected fusion proteins and a protein band with the apparent molecular mass of 50 kDa in the BL21Δ*ompA*(pET21_NHBA) OMV preparation. This is strongly indicative that the presence of Nm-fHbp and NHBA in the OMVs is the result of an active transport through the inner membrane rather than an artifact caused by cell lysis.

##### Nm-fHbp and NHBA are Lipidated in E. coli

**Fig. 2.**
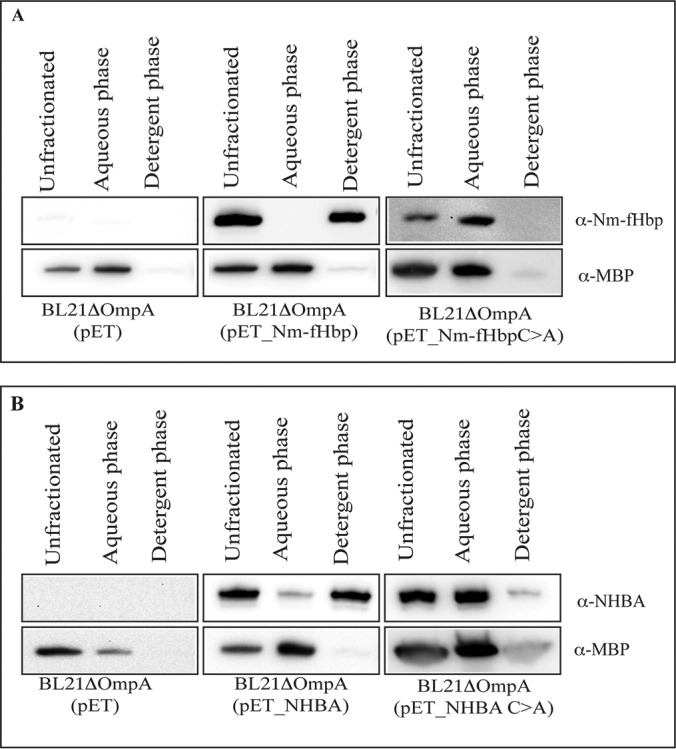
**Analysis of Nm-fHbp and NHBA lipidation by Triton X-114 fractionation of OMV proteins.** OMVs (25 μg of proteins) in 50 μl PBS were dissolved by adding 1% Triton X-114 at 4 °C and subsequently aqueous and detergent phases were partitioned by shifting the temperature at 37 °C. Unfractionated proteins from intact OMVs, hydrophilic proteins in the aqueous phase and hydrophobic proteins in the detergent phase were precipitated with chloroform/methanol, resuspended in SDS-PAGE loading buffer and separated by SDS-PAGE. Finally, proteins were transferred onto nitrocellulose filters and the presence of Nm-fHbp and NHBA in either the aqueous or detergent phases was detected by Western blotting using anti-Nm-fHbp and NHBA specific antibodies. As a control, the partitioning of the periplasmic protein MBP was also analyzed using anti-MBP antibodies. *A*, OMVs from BL21Δ*ompA*, BL21Δ*ompA*(pET_Nm-fHbp) and BL21Δ*ompA(*pET_Nm-fHbpC>A). *B*, BL21Δ*ompA*, BL21Δ*ompA*(pET_NHBA) and BL21Δ*ompA*(pET_NHBAC>A).

With the previous experiments we showed that both Nm-fHbp and NHBA cross and leave the inner membrane as indicated by the fact that they are found in secreted vesicles. Because both proteins carry a canonical lipobox, it is likely that the proteins are acylated and subsequently cleaved by the lipoprotein-specific leader peptidase (the product of *lsp* gene). The evidence that Nm-fHbp is acylated when expressed in *E. coli* was provided more than a decade ago, by Fletcher and coworkers, using mass spectrometry analysis of purified recombinant Nm-fHbp var.2 (47). To confirm that both Nm-fHbp and NHBA are acylated also when expressed in *E. coli* BL21Δ*ompA*, vesicles were solubilized at 4 °C with a 1% water solution of Triton X-114 and subsequently the samples were warmed to 37 °C to partition Triton X-114 into two phases: a detergent-rich “hydrophobic” phase and a detergent-poor “hydrophilic” phase. Membrane proteins, including lipoproteins, typically partition selectively into the Triton X-114 hydrophobic phase (36). As shown in Fig. 2, both Nm-fHb and NHBA compartmentalized in the hydrophobic phase whereas the periplasmic maltose binding protein (MBP) was retained in the aqueous phase. To further support the presence of the lipid moieties at the N termini of the proteins, the cysteine at position +1 of both Nm-fHbp and NHBA was replaced with an alanine, thus preventing the products of *lgt* (lipoprotein glyceryl transferase) and *lnt (*apolipoprotein N-acyltransferase) genes from attaching the acyl chains to the proteins. The presence of the leader peptide should however guarantee the release of the proteins into the periplasmic space and therefore their compartmentalization in the OMVs. As shown in Fig. 2 both Nm-fHbp_C>A and NHBA_C>A were present in OMVs but, similarly to the periplasmic MBP, they partitioned in the aqueous phase of Triton X-114.

##### Nm-fHbp and NHBA Are Exposed on the Surface of E. coli

**Fig. 3.**
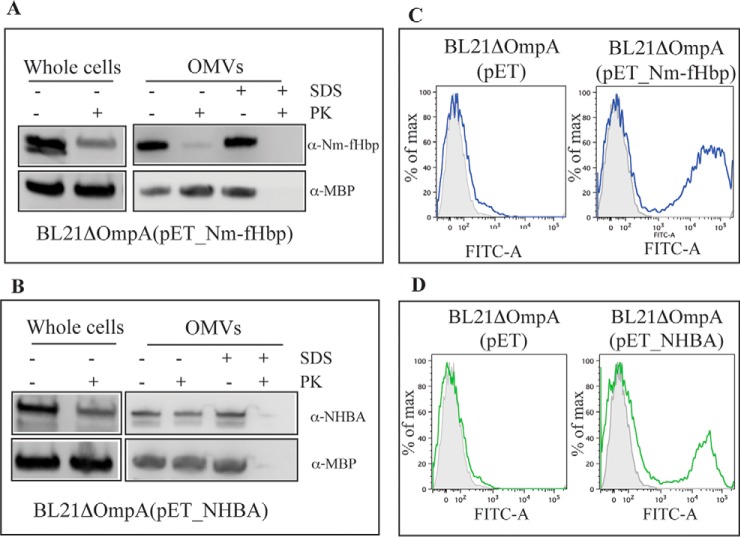
**Assessment of Nm-fHbp and NHBA localization by proteinase K surface shaving and FACS analysis.** *A*, Bacterial cells and purified OMVs from BL21Δ*ompA*(pET_Nm-fHbp) strain were incubated at 37 °C for 2 h with and without Proteinase K (PK) in the presence or absence of 1% SDS. Integrity of Nm-fHbp under the different experimental conditions was analyzed by Western blotting using Nm-fHbp specific antibodies. The integrity of the periplasmic protein MBP was also analyzed as control. *B*, Bacterial cells and purified OMVs from BL21Δ*ompA*(pET_NHBA) strain were treated as in *A* and the integrity of NHBA under the different experimental conditions was analyzed by Western blotting using NHBA-specific antibodies. *C*, Bacterial cells from BL21Δ*ompA* and BL21Δ*ompA* (pET_Nm-fHbp) strains were incubated first with anti-Nm-fHbp specific antibodies and subsequently with FITC-labeled anti-mouse secondary antibodies. Fluorescence was measured by Fluorescence-activated Cell Sorting (FACS). Gray areas represent the background fluorescence signals obtained incubating the cells with the secondary antibody only. *D*, Bacterial cells from BL21Δ*ompA* and BL21Δ*ompA*(pET_NHBA) strains were incubated first with anti-NHBA specific antibodies and subsequently with alexa fluor® 488-labeled anti-mouse secondary antibodies. Fluorescence was measured by FACS. Gray areas represent the background fluorescence signals obtained incubating the cells with the secondary antibody only.

With the previous experiments we provided strong experimental evidence that Nm-fHbp and NHBA are lipidated when expressed in *E. coli* BL21Δ*ompA* strain and that the two lipoproteins are not anchored to the inner membrane, in line with the fact that no retention signatures are present at amino acid positions +2, +3, and +4 of both proteins. Therefore, the two proteins are most likely anchored to the OM, either facing the periplasmic side or exposed on the cell surface, as occurs in *N. meningitidis*. To discriminate between the two possible localizations, we used three experimental approaches. The first approach consisted of the “shaving” of whole cells, and purified OMVs, with the proteolytic enzyme proteinase K. Only proteins on the surface are exposed to the enzyme. Therefore, after digestion with proteinase K, none of the surface anchored proteins should be detectable. Nm-fHbp and NHBA integrity was analyzed by Western blotting using antibodies raised against surface-exposed regions of the proteins. Specifically, anti-Nm-fHbp antibodies were raised against a synthetic peptide spanning from amino acid 35 to amino acid 50 involved in factor H binding, whereas anti-NHBA antibodies were against the carboxy-terminal domain of the protein (from amino acid 408 to amino acid 421). As shown in Fig. 3*A*, Nm-fHbp was no longer visible after 30-min OMV incubation with proteinase K, indicating that at least the domain recognized by the antibody was sufficiently exposed to be completely digested. Surface localization of Nm-fHbp was also confirmed by shaving experiments carried out on whole *E. coli* cells expressing Nm-fHbp. Interestingly, in *E. coli* whole cell extracts, anti-Nm-fHbp antibodies recognized two protein species differing in molecular mass by ∼2 kDa. After shaving, only the lower molecular weight band completely disappeared, in keeping with the notion that the lipidated mature form of Nm-fHbp localizes on the outer membrane, whereas the Nm-fHbp precursor carrying the leader peptide is present in the cytoplasm and/or the inner membrane and therefore not accessible to proteinase K. Proteinase K treatment of NHBA-expressing whole cells and OMVs did not significantly affect the intensity of the antibody-reacting protein band in Western blotting, indicating that the protein is either not easily accessible to proteinase K or protease resistant (Fig. 3*B*). Our second approach to investigate the surface localization of Nm-fHbp and NHBA on BL21Δ*ompA*(pET21_Nm-fHbp) and BL21Δ*ompA*(pET21_NHBA) strains was by FACS using anti-Nm-fHbp and anti-NHBA-specific antibodies. Cultures were grown to OD_600_ = 0.5, then the expression of Nm-fHbp and NHBA was induced by addition of 1 mm IPTG. Cells were collected by centrifugation, incubated with the corresponding protein-specific polyclonal antibodies and subsequently with secondary anti-rabbit antibodies labeled with Alexa Fluor® 488. Finally, cells were resuspended in 2% formaldehyde and analyzed by FACS. Both proteins were surface-associated, as shown in Fig. 3*C* and 3*D* by the positive fluorescence signal observed in a considerable fraction of the cell population.

**Fig. 4.**
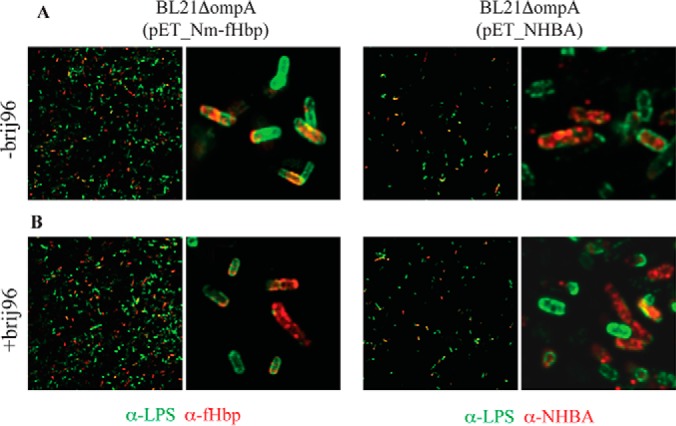
**Assessment of localization of Nm-fHbp and NHBA expressed in *E. coli* BL21Δ*ompA*.** *A*, After induction of protein expression with IPTG, bacterial cells from BL21Δ*ompA*(pET_Nm-fHbp) (left) and BL21Δ*ompA*(pET_NHBA) (right) strains were fixed with 2% formaldehyde solution and incubated with anti-Nm-fHbp and anti-NHBA antibodies, respectively, and with a mouse monoclonal antibody specific for the core region of LPS. The binding of anti-Nm-fHbp and anti-NHBA antibodies was visualized with alexa fluor® 594-labeled anti-rabbit antibodies (red), whereas anti-LPS antibody binding was followed using alexa fluor® 488-labeled anti-mouse antibody (green). *B*, Cells were treated as in (*A*) but permeabilized with 0,1% Brij96.

Finally, confocal microscopy was also used to confirm the presence of Nm-fHbp and NHBA on the surface of *E. coli*. After induction of Nm-fHbp and NHBA expression as described above, bacteria were fixed with 2% formaldehyde solution and incubated with anti-Nm-fHbp and anti-NHBA antibodies and with a mouse monoclonal antibody specific for the core region of lipopolysaccharide (LPS). The binding of anti-Nm-fHbp and anti-NHBA antibodies was detected with Alexa Fluor® 594-labeled anti-rabbit antibodies (red), while anti-LPS antibody binding was detected using Alexa Fluor® 488-labeled anti-mouse antibodies (green). As expected the BL21Δ*ompA* strain was effectively stained by anti-LPS antibodies, and not recognized by anti-Nm-fHbp and anti-NHBA antibodies (supplemental Fig. S1), whereas both Nm-fHbp and NHBA were detected on the surface of the corresponding recombinant strains (Fig. 4*A*).

The analyses from both FACS and confocal microscopy experiments revealed that only a fraction of the whole bacterial populations, visualized with the anti-LPS antibody, expressed the lipoproteins on their surface. The existence of two populations, one expressing and the other not expressing the lipoproteins, is reminiscent of the well-recognized lactose operon-associated bistability occurring when cells are subjected to suboptimal concentrations of a gratuitous inducer (48, 49) (see also Discussion). To confirm that the lack of surface staining of a subset of the bacterial population was not because of the protein retention in the cytoplasm or in the periplasm but rather to absence of expression, bacteria were stained after membrane permeabilization with 0,1% Brij96. As shown in Fig. 4*B*, differently from what occurred with antibodies against the periplasmic maltose bind protein (supplemental Fig. S1*C*), permeabilization did not substantially increase the fraction of Nm-fHbp and NHBA positive bacteria, indicating that when expressed both lipoproteins reached the bacterial surface.

Overall, these data indicate that both Nm-fHbp and NHBA are exposed on the surface of *E. coli*. The fact that surface-exposed NHBA is only slightly digested by proteinase K might indicate that NHBA is embedded into the membrane, making it visible to anti-NHBA antibodies but not easily accessible to the proteolytic enzyme. Alternatively, NHBA could also be resistant to proteolytic attack under the conditions used.

As already pointed out, in *Neisseria*, in the absence of the Slam1 membrane protein, some lipoproteins, including the Nm-fHbp used in this study, were not exposed on the surface of *E. coli* BL21-C43 strain (17). These data appear to be in contrast with data previously published by Konar *et al.* (20) and our findings. To ensure that the *ompA* deletion in BL21 strain was not affecting cell surface expression of Nm-fHbp and NHBA, we also analyzed their localization in the wild-type BL21 strain. As shown in supplemental Fig. S2, FACS analysis and confocal microscopy revealed that both proteins reached the cell surface with efficiencies like that observed in BL21Δ*ompA* strain. We also analyzed the expression and surface localization of Nm-fHbp in *E. coli* BL21-C43, the same strain used by Hooda and coworkers for the heterologous expression of Nm-fHbp in *E. coli*. The protein was efficiently expressed in this strain, as indicated by the fact that it was visible by SDS-PAGE analysis of total cell extracts (supplemental Fig. S3*A*). However, in agreement with the data published by Hooda *et al,* it was not detected on the cell surface. (supplemental Fig. S3*B*). Interestingly, when we analyzed the expression of Nm-fHbp in *E. coli* BL21-C43 by confocal microscopy, we found that anti-Nm-fHbp antibodies could stain the bacterial cells only after membrane permeabilization. Therefore, differently from both wild-type *E. coli* BL21 and BL21Δ*ompA* strains, it appears that *E. coli* BL21-C43 does not efficiently expose Nm-fHbp on its surface.

To fully reconcile our data with the data from Hooda *et al.*, we coexpressed Slam1 with Nm-fHbp in the three *E. coli* strains BL21Δ*ompA*, BL21, and BL21-C43. The full length *slam1* gene, including the region coding for the leader peptide for secretion, was PCR-amplified from MC58 chromosomal DNA and inserted into pACYC plasmid under the control of the T7 inducible promoter, generating pACYC-slam1 plasmid (see Materials and Methods for details). Because pET21b^+^ and pACYC plasmids have compatible origins of replication they can coexist in the same cell. The plasmid was then used to transform the three *E. coli* strains carrying pET-Nm-fHbp. As a control the same strains were transformed with the “empty” pACYC vector not carrying the *slam1* gene. The recombinant strains were grown in LB medium up to OD_600_ = 0.5 and after 2 h incubation in 1 mm IPTG bacterial cells were collected and analyzed by FACS as described above, supplemental Fig. S4 illustrates the results of the experiment. We found that the presence of Slam1 enhanced the fraction of bacterial cells carrying surface-exposed Nm-fHbp, and the increment was particularly evident in BL21-C43 strain. To further confirm the specificity of the antibody binding to surface-exposed Nm-fHbp, an antibody binding competition experiment was carried out. Bacterial cells from BL21Δ*ompA*(pET_Nm-fHbp)(pACYC) and BL21Δ*ompA* (pET_Nm-fHbp) (pACYC_slam1) cultures were mixed with different concentration of the Nm-fHbp-derived peptide*_35–50_* or the NHBA-derived peptide*_408–421_* and subsequently the binding of anti-Nm-fHbp antibodies was followed by FACS analysis. As shown in supplemental Fig. S4*B*, only the Nm-fHbp-derived peptide*_35–50_* prevented the binding of anti-Nm-fHbp antibodies to both BL21Δ*ompA*(pET_Nm-fHbp)(pACYC) and BL21Δ*ompA*(pET_Nm-fHbp)(pACYC_slam1) strains in a dose-dependent manner.

Finally, we coexpressed NHBA with Slam1 by transforming BL21Δ*ompA* (pET_NHBA) with pACYC_slam1 plasmid and we followed the surface exposition of NHBA by FACS analysis. As shown in supplemental Fig. S5, the presence of Slam1 did not affect the level of NHBA exposition.

From these data we conclude that Slam1 has a quantitative effect on Nm-fHbp surface exposition in *E. coli* and such effect was particularly pronounced in BL21-C43 strain. In addition, our data unequivocally confirmed that the transport of Nm-fHbp through the *E. coli* outer membrane efficiently occurred independently from Slam1. Finally, the surface localization of NHBA was not substantially influenced by the presence of Slam1.

##### The Factor H Binding Protein from A. actinomycetemcomitans Is Also Surface-exposed When Expressed in E. coli

**Fig. 5.**
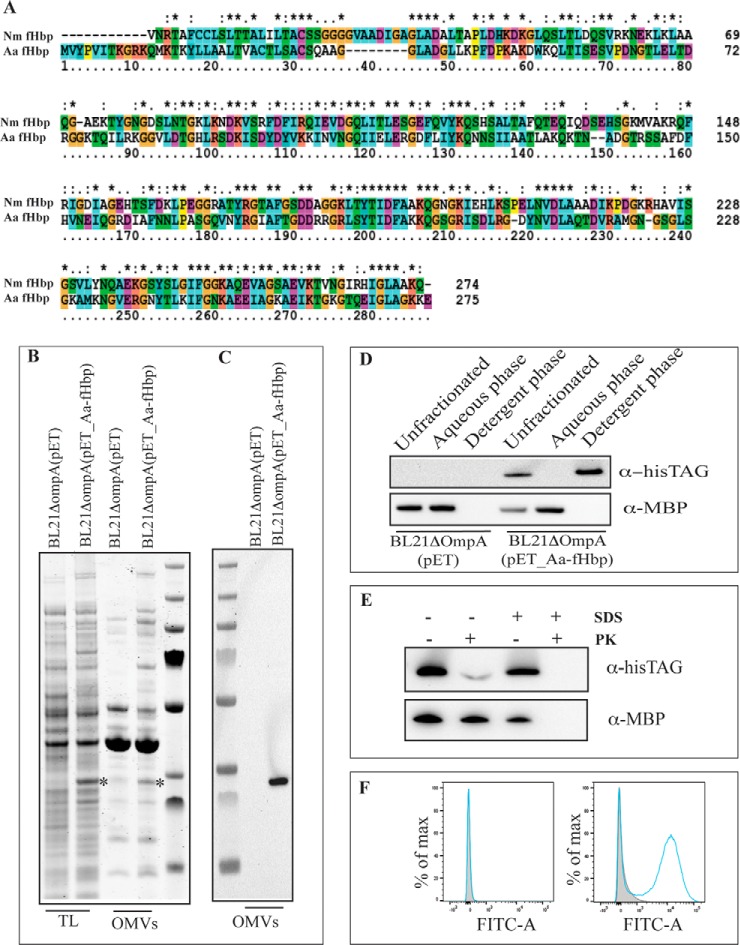
**Expression and surface localization of Aa-fHbp in BL21Δ*ompA* and derived OMVs.** *A*, Protein sequence alignment of Nm-fHbp and Aa-fHbp. *B*, SDS-PAGE analysis of total cell extracts (TL) and OMVs isolated from BL21Δ*ompA*(pET_Aa-fHbp) strain. *C*, Western blotting analysis of OMVs purified from BL21Δ*ompA*(pET_Aa-fHbp) strain. *D*, Analysis of Aa-fHbp lipidation by Triton X-114 fractionation of OMV proteins (see Fig. 2 for experimental details). *E*, Assessment of Aa-fHbp localization by proteinase K surface shaving. *F*, Assessment of Aa-fHbp localization by FACS analysis (see Fig. 3 for experimental details). Aa-fHbp detection was carried out using anti-His-tag antibodies.

The experiments described above indicate that two lipoproteins naturally surface-exposed in *N. meningitidis* reach the same cellular compartment when expressed in a different Gram-negative species. Therefore, whatever mechanism is involved in lipoprotein transport through the OM, it appears to be sufficiently conserved between these two organisms. To further support this observation, we studied the sorting fate of a third lipoprotein, the factor H binding protein from *A. actinomycetemcomitans* (Aa-fHbp). *A. actinomycetemcomitans* is a human pathogen associated with aggressive periodontitis and endocarditis (50, 51). The genome sequence of the pathogen revealed the presence of an open reading frame (ORF) encoding a lipoprotein (Aa-fHbp) sharing 41% amino acid identity with Nm-fHbp var.1 (Fig. 5*A*). Recent proteomic analyses showed that Aa-fHbp localized in the culture supernatants and/or in OMVs of *A. actinomycetemcomitans* (52, 53). Although no direct evidence has been reported so far on the localization of Aa-fHbp, its predicted Factor H binding capacity strongly suggests that the protein protrudes out of the outer membrane of *A. actinomycetemcomitans*.

To investigate whether Aa-fHbp was surface-associated when expressed in *E. coli*, the gene encoding the entire Aa-fHbp preprolipoprotein was chemically synthesized and cloned into pET21b^+^ plasmid, fused to a histidine tag at the C terminus. The pET_Aa-fHbp-His_8_ recombinant plasmid was used to transform *E. coli* BL21Δ*ompA* and the expression and localization of Aa-fHbp-His_8_ was analyzed as described previously for Nm-fHbp and NHBA. Fig. 5 illustrates the results of all experiments. Aa-fHbp-His_8_ was well expressed in the *E. coli* BL21Δ*ompA* strain and compartmentalized in the OMVs (Fig. 5*B* and 5*C*). Aa-fHbp was lipidated as suggested by its partitioning in the organic phase after Triton X-114 extraction (Fig. 5*D*) and reached the surface of the cell, as demonstrated by the Proteinase K shaving assay and FACS analysis using anti-His-tag antibodies on *E. coli* BL21Δ*ompA* (pET_Aa-fHbp-His_8)_ strain (Fig. 5*E* and 5*F*).

##### Alteration of the N-terminal Glycine Motif and Deletion of Two Thirds of the Protein Length Do Not Prevent Nm-fHbp from Reaching the Surface

**Fig. 6.**
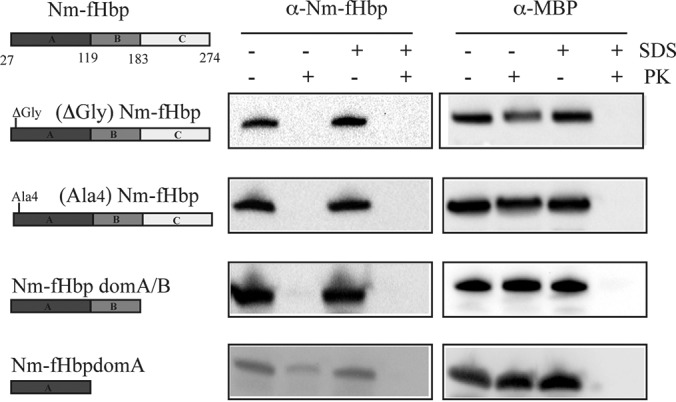
**Analysis of surface localization of Nm-fHbp mutants.** OMVs from recombinant strains expressing the different Nm-fHbp mutants (see Text for details) were treated with Proteinase K as described in Fig. 3 and subsequently analyzed by Western blotting using anti-Nm-fHbp specific antibodies. The periplasmic protein MBP was used as a control of the integrity of OMVs after PK treatment.

We finally investigated whether the capacity of Nm-fHbp to reach the bacterial surface involves specific amino acids and/or protein domains. To address this question, we focused on two features of the protein. The first one is the stretch of four glycine residues immediately following the N-terminal cysteine. Such glycine containing motifs are often found in *Neisseria* lipoproteins, as well as in lipoproteins from other Gram-negative bacteria. Therefore, we asked the question whether glycine substitution or deletion could impair Nm-fHbp surface sorting. Second, we created two extensive C-terminal deletions of Nm-fHbp and analyzed whether Nm-fHbp-Domain A, the domain that carries the key Arginine residue involved in Factor H binding (54), was still capable of reaching the bacterial surface. Fig. 6 is a schematic representation of the constructs that were generated. In the Nm-(ΔGly)fHbp construct, the four glycine residues were deleted, thus juxtaposing the N-terminal cysteine with the sixth amino acid of mature Nm-fHbp. Nm-(Ala_4_)fHbp is characterized by the presence of a four-Alanine stretch in place of the Glycine residues. The Nm-fHbpDomAB protein was created by deleting the last 90 amino acids which constitute Nm-fHbp Domain C. Finally, in the Nm-fHbpdomA construct, Nm-fHbp was truncated to remove both Domain B and Domain C. The four Nm-fHbp mutants were expressed in *E. coli* BL21Δ*ompA* strain and their compartmentalization in OMVs analyzed by SDS-PAGE and Western blotting. All four Nm-fHbp mutants were transported to the vesicular compartment (data not shown). The vesicles were then subjected to proteinase K treatment to investigate their localization on the OMV surface. As shown in Fig. 6, the four mutants were exposed on the surface of the vesicles as indicated by the disappearance of the corresponding protein band after proteolytic shaving of the vesicles.

##### Nm-fHbp and NHBA Can Efficiently Deliver Heterologous Polypeptides to the Surface of E. coli and to the Vesicle Compartment

Having demonstrated the surface localization of Nm-fHbp and NHBA, we wanted to know whether these surface-exposed lipoproteins could serve as vehicles to deliver foreign polypeptides to the *E. coli* surface. We focused our attention on two known cancer antigens, which have been shown to have promising immunotherapy potential in preclinical and clinical settings. The first antigen is a synthetic polypeptide, constituted of the fusion of three copies of the EGRFvIII 14 amino acid epitope. Epidermal growth factor receptor variant III (EGFRvIII) is a truncated variant of EGFR, which carries a unique 14 amino acid sequence (LEEKKGNYVVTDH) generated by the 801 bp deletion of exons 2–7. This 14-amino-acid peptide has been extensively used in preclinical and clinical cancer vaccine studies with promising results (55, 56). A peptide-based vaccine is currently in Phase III trial for the immunotherapy of glioblastoma (57).

The second antigen is a 100-amino-acid synthetic polypeptide constituted of five copies of the PDTRPAPGSTAPPAHGVTSA sequence found repeated 20 to 150 times in the extracellular domain of the transmembrane glycoprotein Mucin 1 (MUC1) and constituting the so called “variable number of tandem repeats region (VNTR)” (58). In normal epithelia, VNTR is highly glycosylated in Serine and Threonine whereas in most adenocarcinomas such as those of breast, ovary, colon, pancreas, lung, and in premalignant lesions MUC1 becomes over-expressed and hypoglycosylated. Several vaccines based on VNTR have been tested in PhaseI/II studies with some positive results (59).

**Fig. 7.**
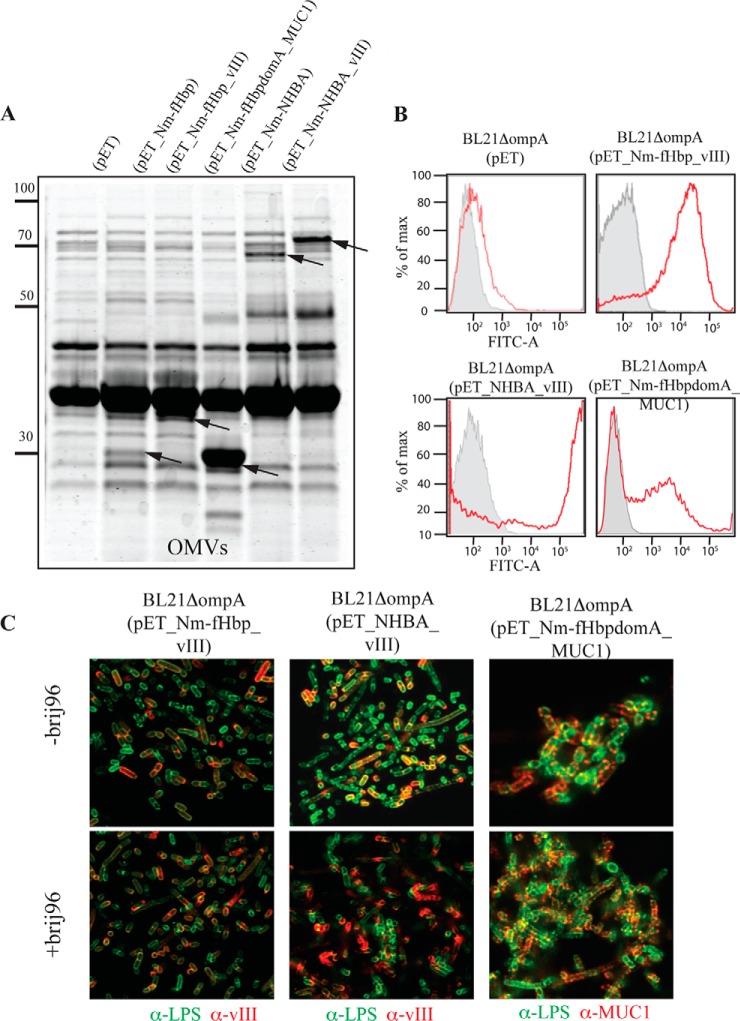
**Expression and surface localization of fHbp-VIII, NHBA-VIII, and fHbp-MUC1 fusion proteins in BL21Δ*ompA* strain and derived OMVs.** *A*, OMVs proteins from BL21Δ*ompA*(pET), BL21Δ*ompA*(pET_Nm-fHbp), BL21Δ*ompA*(pET_Nm-fHbp_vIII), BL21Δ*ompA*(pET_NHBA), BL21Δ*ompA*(pET_NHBA_vIII), and BL21Δ*ompA*(pET_Nm-fHbpDomA_MUC1) strains were purified as described in Fig. 1 and analyzed by SDS-PAGE. Bands corresponding to the heterologous fusion proteins are indicated by arrows. *B*, BL21Δ*ompA*(pET), BL21Δ*ompA*(pET_Nm-fHbp_vIII), BL21Δ*ompA*(pET_NHBA_vIII), and BL21Δ*ompA*(pET_Nm-fHbpDomA_MUC1) strains were grown in LB at 37 °C. At OD_600_ = 0.5, 1 mm IPTG was added and after 2 h bacterial cells were collected by centrifugation and incubated with (red histogram) or without (gray histogram) anti-EGFRvIII or anti-MUC1 specific antibodies, and subsequently with alexa fluor® 488-labeled anti-rabbit secondary antibodies. Fluorescence was measured by Fluorescence-activated Cell Sorting(FACS). *C*, Bacterial cells from BL21Δ*ompA*(pET), BL21Δ*ompA*(pET_Nm-fHbp_vIII), BL21Δ*ompA*(pET_NHBA_vIII), and BL21Δ*ompA*(pET_Nm-fHbpDomA_MUC1) strains were fixed with 2% formaldehyde solution and incubated with anti-EGFRvIII or anti-MUC1 antibodies and with mouse monoclonal antibodies specific for the core region of LPS. For intracellular staining, bacteria were first treated with 0.1% Brj96 for 5 min at room temperature RT (+brij96). The binding of anti-vIII and anti-MUC1 antibodies was visualized with alexa fluor 594®-labeled anti-rabbit antibodies (red), while anti-LPS antibody binding was followed using alexa fluor® 488-labeled anti-mouse antibody (green).

EGFRvIII polyepitope encoding DNA was fused to the 3′ end of Nm-fHbp and NHBA coding sequences, generating plasmids pET-Nm-fHbp-vIII and pET-NHBA-vIII. The two plasmids were used to transform *E. coli* BL21Δ*ompA* strain and after induction with IPTG the compartmentalization of the fused proteins in the outer membrane was indirectly verified by following their accumulation into the vesicular compartment. As shown in Fig. 7*A*, proteins species with molecular masses corresponding to the fused proteins were clearly visible by SDS-PAGE. The identity of the two-protein species was confirmed by Western blotting analysis using anti-EGFRvIII peptide antibodies (data not shown). We next asked the question whether, when fused to Nm-fHbp and NHBA, the EGFRvIII polypeptide could reach the surface of *E. coli*. To address this question, we incubated bacterial cells with anti-EGFRvIII antibodies and we followed antibody binding using FACS and confocal microscopy analyses. As shown in Fig. 7*B* and 7*C*, cells were efficiently recognized by anti-EGFRvIII antibodies, indicating that the C termini of the two chaperone proteins protrude out of the outer membrane and can expose the foreign EGFRvIII polypeptide to the surface.

To confirm the potential of lipoproteins, which spontaneously cross the outer membrane as surface delivery systems, the MUC1 polypeptide was fused to the C terminus of Nm-fHbpDomA by linking the synthetic MUC1X5 encoding minigene to the 3′ end of Nm-fHbpDomA gene, creating pET-Nm-fHbpDomA-MUC1 plasmid. Similarly, to the EGFRvIII polypeptide, the Nm-fHbpDomA-MUC1 fusion efficiently compartmentalized in OMVs and reached the bacterial surface as observed by FACS and confocal microscopy analysis using anti-MUC1 antibodies (Fig. 7*A*, 7*B*, and 7*C*).

## DISCUSSION

In Gram-negative bacteria, lipoproteins are destined to three main cellular compartments: the periplasmic side of the IM, the periplasmic side of the OM, and the external side of the OM. Once considered to be a relatively rare event, finding lipoproteins facing the external milieu has become a rule rather than an exception in certain Gram-negative species (14), particularly in pathogenic species such *Borrelia* spirochetes (15) and *Neisseria spp.*

The mechanisms involved in the translocation of lipoproteins through the outer membrane remain to be fully elucidated. From what is described in the literature, surface-exposed lipoproteins can be arbitrarily classified in two groups based on their propensity or recalcitrance to maintain surface topology when transplanted from one species to another. Lipoproteins that cross the OM supported by specific transport machineries such as T2SS, T5SS, Bam, and flippases (6–8, 11, 14) usually are species-specific. By contrast, a still poorly characterized second group of lipoproteins cross the outer membrane without dedicated ancillary systems and reach the surface even in heterologous hosts, probably using mechanisms conserved among Gram-negative species. The existence of such category of lipoproteins was reported some time ago in at least two publications describing the heterologous expression of *S. enterica* YaiW and *V. cholerae* VolA in *E. coli* (18, 19). Furthermore, partially in contrast to what subsequently published by Hooda and coworkers (17), Konar *et al.* showed that the *Neisseria* Factor H binding protein (Nm-fHbp) could spontaneously reach the *E. coli* surface in a structurally functional conformation, as judged by its capacity to bind its natural ligand, Factor H (20).

In this study we have further investigated the existence of the second group of lipoproteins, by following the compartmentalization of three surface-exposed lipoproteins from *N. meningitidis* (Nm-fHbp and NHBA) and *A. actinomycetemcomitans* (Aa-fHbp) in *E. coli* where, apart from few exceptions (10, 12, 13, 60–62), its more than 90 lipoproteins are intracellular. The data presented in this work indicate that Nm-fHbp, NHBA and Aa-fHbp crossed the *E. coli* IM and reached the OM as indicated by their compartmentalization in the OMVs and by their partitioning in the hydrophobic phase after Triton X-114 treatment. Our 1D gel, 2D gel, and mass spectrometry analyses provide strong evidence that the lipoproteins are actively transported to the outer membrane: the vesicles are not contaminated by cytoplasmic proteins and the three proteins accumulated into OMVs. Furthermore, we showed by FACS and confocal microscopy analyses that the three lipoproteins appear on the surface of *E. coli* cells.

Overall, our data indicate that Nm-fHbp, NHBA, and Aa-fHbp belong to the class of surface-exposed lipoproteins, which maintain their cellular compartment when transplanted from one Gram-negative species to another. Because Hooda *et al.* (17) reported that Nm-fHbp requires Slam1 to be transported to the surface, to reconcile our data with Hooda *et al*. data we analyzed the compartmentalization of Nm-fHbp in the presence or absence of Slam1 in three *E. coli* strains: BL21, BL21Δ*ompA,* and BL21-C43 (63), the latter being the same strain used by Hooda and coworkers. We found that the presence of Slam1 in BL21-C43 strain strongly enhanced the fraction of bacterial cells carrying surface-exposed Nm-fHbp (supplemental Fig. S4). However, in the other two strains Slam1 contributes to the transport of Nm-fHbp, but is clearly not strictly necessary. Therefore, Slam1 appears to act as a “facilitator” of Nm-fHbp transport, probably increasing the kinetics of protein accumulation on the *E. coli* surface. We cannot exclude that Slam1 might also affect the topology and/or surface distribution of Nm-fHbp, but analyses other than FACS and confocal microscopy should be employed to address this possibility.

As pointed out above, the three heterologous lipoproteins were exposed on the *E. coli* surface, but they were expressed in a fraction of whole bacterial population and the percentage of expressing *versus* nonexpressing cells varied from culture to culture. This was not because of structural/segregational instability of the pET-Nm-fHbp plasmid because we confirmed the genetic homogeneity of the cultures by plasmid extraction and Nm-fHbp sequence analysis from several colonies (data not shown). Therefore, we hypothesize that the two populations are the result of a phenomenon known as bistability. The existence of bistability, in which a genetically homogeneous bacterial culture separates into two populations, either expressing or not expressing a specific gene, has been well documented (48, 64, 65). A prototypic example of bistability is the expression of the lac operon, which at certain concentrations of the gratuitous inducer IPTG (the same inducer used for the expression of the lipoproteins in our system) is fully activated in some cells while not activated in others (48). Interestingly, we found that in BL21 and BL21Δ*ompA* strains cotransformed with pET-Nm-fHbp and pACYC vectors, the fraction of bacteria expressing Nm-fHbp was enhanced with respect to the same strains not carrying pACYC (supplemental Fig. S4). It is possible that coexistence in the same cell of two expression plasmids, both utilizing IPTG for driving transcription, might change the availability of the inducer for the expression of each gene, influencing bistability.

An open question remains about the mechanism exploited by lipoproteins to find their way through the OM. One possible mechanism is that once the lipid moiety is inserted into the inner leaflet of the OM, the C-terminal portion bends outward and gets inserted into the OM (66). For this to happen in an environment deprived of ATP and other high-energy carriers, lipoproteins should have structural features that allow them to interact with the hydrophobic layers of the outer membrane. Based on the available 3D structures, both Nm-fHbp and NHBA consist of an eight-stranded β-barrel that resembles the structural organization of the C-terminal domain of autotransporters and OM proteins (67). Therefore, they could theoretically reach the surface following a pathway like these families of proteins. Although this could be the case, our data indicate that when the B and C domains (the domains involved in the formation of the β-barrel) are removed, Nm-fHbp domain A is still capable of reaching the OM surface. Therefore, a β-barrel structure is not strictly necessary for cell surface localization. A second mechanism, serving as a generalized surface transport system, could involve BamA (beta-barrel assembly machine A). As shown by Konovalova *et al.* for RcsF (regulator of capsule synthesis) (10), BamA could act as general acceptor of the tri-acylated N termini of lipoproteins and once the lipids are tethered to the external side of the membrane, the C-terminal portion of lipoproteins cross the OM, possibly interacting with some integral membrane proteins. Finally, a third possible candidate of lipoprotein transport is the LPS delivery pathway (68, 69) that has several analogies with lipoprotein sorting (5, 70, 71). An attractive hypothesis is that lipoproteins are transferred from the lipoprotein localization factor B (LolB) to the two-protein component of the LPS assembly complex LptDE, which promotes the flipping out of LPS. Based on the structural data which have outlined the mechanisms of LPS transport (72–75), LptD (lipopolysaccharide transport D), the largest β-barrel integral membrane protein so far described in Gram-negative bacteria, could potentially eject the lipid moiety of lipoproteins using its greasy jellyroll domain, the same way it does with Lipid A. At the same time, it could allow the translocation of the protein moiety through its large hole also thanks to the lateral opening of the β-barrel domain.

Whatever mechanism is used for reaching the bacterial surface, the process is efficient and the amount of lipoproteins that ultimately accumulates on the surface can be remarkable. This feature can be biotechnologically exploited to chaperon foreign polypeptides to the cell surface. We tested this possibility by fusing two polypeptides consisting in repeated units of two known cancer epitopes (EGFRvIII and MUC1) to the C terminus of Nm-fHbp, Nm-fHbpDomA and NHBA. In all cases the polypeptides were efficiently transported to the bacterial surface and incorporated into the outer membrane vesicles. Considering that EGFRvIII and MUC1 peptides are promising candidates for cancer therapy, our results pave the way to test the ability of engineered bacteria and OMVs to elicit anti-EGFRvIII and anti-MUC1 specific immune responses.

EGFRvIII and MUC1 polypeptides have a length of 45 and 100 amino acids, respectively. It will be interesting to investigate the maximum size of the passenger polypeptide/protein that can be chaperoned to the surface by lipoproteins. This will establish the feasibility of this platform for future applications.

## DATA AVAILABILITY

The raw MS data files have been deposited to the ProteomeXchange Consortium (http://proteomecentral.proteomexchange.org) via the PRIDE partner repository (http://www.ebi.ac.uk/pride/archive/) with the data set identifier PXD005732.

## Supplementary Material

Supplemental Data

## Footnotes

^1^ The abbreviations used are:

OM: outer membrane
PIPE: polymerase incomplete primer extension
2D: two dimensional
IPG: immobilized pH gradient
PMF: protein mass ffingerprinting
FACS: fluorescence-activated cell sorting
OMV: outer membrane vesicles
VNTR: variable number of tandem repeats.

## References

[1] Kovacs-Simon A., Titball R. W., and Michell S. L. (2011) Lipoproteins of bacterial pathogens. Infect. Immun. 79, 548–561 20974828 10.1128/IAI.00682-10 PMC3028857

[2] Hutchings M. I., Palmer T., Harrington D. J., and Sutcliffe I. C. (2009) Lipoprotein biogenesis in Gram-positive bacteria: knowing when to hold 'em, knowing when to fold 'em. Trends Microbiol. 17, 13–21 19059780 10.1016/j.tim.2008.10.001

[3] Tokuda H. (2009) Biogenesis of outer membranes in Gram-negative bacteria. Biosci. Biotechnol. Biochem. 73, 465–473 19270402 10.1271/bbb.80778

[4] Tokuda H., and Matsuyama S. (2004) Sorting of lipoproteins to the outer membrane in E. coli. Biochim. Biophys. Acta 1694, IN1–IN9 15672528

[5] Bos M. P., Robert V., and Tommassen J. (2007) Biogenesis of the gram-negative bacterial outer membrane. Annu. Rev. Microbiol. 61, 191–214 17506684 10.1146/annurev.micro.61.080706.093245

[6] d'Enfert C., Ryter A., and Pugsley A. P. (1987) Cloning and expression in Escherichia coli of the Klebsiella pneumoniae genes for production, surface localization and secretion of the lipoprotein pullulanase. EMBO J. 6, 3531–3538 3322811 10.1002/j.1460-2075.1987.tb02679.x PMC553813

[7] van Ulsen P., van Alphen L., ten Hove J., Fransen F., van der Ley P., and Tommassen J. (2003) A Neisserial autotransporter NalP modulating the processing of other autotransporters. Mol. Microbiol. 50, 1017–1030 14617158 10.1046/j.1365-2958.2003.03773.x

[8] Oomen C. J., van Ulsen P., van Gelder P., Feijen M., Tommassen J., and Gros P. (2004) Structure of the translocator domain of a bacterial autotransporter. EMBO J. 23, 1257–1266 15014442 10.1038/sj.emboj.7600148 PMC381419

[9] Robert V., Volokhina E. B., Senf F., Bos M. P., Van Gelder P., and Tommassen J. (2006) Assembly factor Omp85 recognizes its outer membrane protein substrates by a species-specific C-terminal motif. PLoS Biol. 4, e377 17090219 10.1371/journal.pbio.0040377 PMC1634882

[10] Konovalova A., Perlman D. H., Cowles C. E., and Silhavy T. J. (2014) Transmembrane domain of surface-exposed outer membrane lipoprotein RcsF is threaded through the lumen of beta-barrel proteins. Proc. Natl. Acad. Sci. U.S.A. 111, E4350–E4358 25267629 10.1073/pnas.1417138111 PMC4205638

[11] Konovalova A., and Silhavy T. J. (2015) Outer membrane lipoprotein biogenesis: Lol is not the end. Philos. Trans. R. Soc. Lond. B. Biol. Sci. 370 10.1098/rstb.2015.0030 PMC4632606 26370942

[12] Cowles C. E., Li Y., Semmelhack M. F., Cristea I. M., and Silhavy T. J. (2011) The free and bound forms of Lpp occupy distinct subcellular locations in Escherichia coli. Mol. Microbiol. 79, 1168–1181 21219470 10.1111/j.1365-2958.2011.07539.x PMC3090202

[13] Michel L. V., Shaw J., MacPherson V., Barnard D., Bettinger J., D'Arcy B., Surendran N., Hellman J., and Pichichero M. E. (2015) Dual orientation of the outer membrane lipoprotein Pal in Escherichia coli. Microbiology 161, 1251–1259 25808171 10.1099/mic.0.000084 PMC4635515

[14] Zuckert W. R. (2014) Secretion of bacterial lipoproteins: through the cytoplasmic membrane, the periplasm and beyond. Biochim. Biophys. Acta 1843, 1509–1516 24780125 10.1016/j.bbamcr.2014.04.022 PMC4070597

[15] Schulze R. J., and Zuckert W. R. (2006) Borrelia burgdorferi lipoproteins are secreted to the outer surface by default. Mol. Microbiol. 59, 1473–1484 16468989 10.1111/j.1365-2958.2006.05039.x

[16] Schulze R. J., Chen S., Kumru O. S., and Zückert W. R. (2010) Translocation of Borrelia burgdorferi surface lipoprotein OspA through the outer membrane requires an unfolded conformation and can initiate at the C-terminus. Mol. Microbiol. 76, 1266–1278 20398211 10.1111/j.1365-2958.2010.07172.x PMC2999405

[17] Hooda Y., Lai C. C., Judd A., Buckwalter C. M., Shin H. E., Gray-Owen S. D., and Moraes T. F. (2016) Slam is an outer membrane protein that is required for the surface display of lipidated virulence factors in Neisseria. Nat. Microbiol. 1, 16009 27572441 10.1038/nmicrobiol.2016.9

[18] Arnold M. F., Caro-Hernandez P., Tan K., Runti G., Wehmeier S., Scocchi M., Doerrler W. T., Walker G. C., and Ferguson G. P. (2014) Enteric YaiW is a surface-exposed outer membrane lipoprotein that affects sensitivity to an antimicrobial peptide. J. Bacteriol. 196, 436–444 24214946 10.1128/JB.01179-13 PMC3911249

[19] Pride A. C., Herrera C. M., Guan Z., Giles D. K., and Trent M. S. (2013) The outer surface lipoprotein VolA mediates utilization of exogenous lipids by Vibrio cholerae. MBio 4, e00305–13 23674613 10.1128/mBio.00305-13 PMC3656444

[20] Konar M., Rossi R., Walter H., Pajon R., and Beernink P. T. (2015) A mutant library approach to identify improved meningococcal factor H binding protein vaccine antigens. PLoS ONE 10, e0128185 26057742 10.1371/journal.pone.0128185 PMC4461315

[21] Tozakidis I. E., Sichwart S., and Jose J. (2015) Going beyond E. coli: autotransporter based surface display on alternative host organisms. Nat. Biotechnol. 32, 644–650 10.1016/j.nbt.2014.12.008 25579193

[22] Schuurmann J., Quehl P., Festel G., and Jose J. (2014) Bacterial whole-cell biocatalysts by surface display of enzymes: toward industrial application. Appl. Microbiol. Biotechnol. 98, 8031–8046 25104026 10.1007/s00253-014-5897-y

[23] Lee S. H., Choi J. I., Han M. J., Choi J. H., and Lee S. Y. (2005) Display of lipase on the cell surface of Escherichia coli using OprF as an anchor and its application to enantioselective resolution in organic solvent. Biotechnol. Bioeng, 90, 223–230 15739170 10.1002/bit.20399

[24] Tanaka T., Kawabata H., Ogino C., and Kondo A. (2011) Creation of a cellooligosaccharide-assimilating Escherichia coli strain by displaying active beta-glucosidase on the cell surface via a novel anchor protein. Appl. Environ. Microbiol. 77, 6265–6270 21742905 10.1128/AEM.00459-11 PMC3165374

[25] Francisco J. A., Earhart C. F., and Georgiou G. (1992) Transport and anchoring of beta-lactamase to the external surface of Escherichia coli. Proc. Natl. Acad. Sci. U.S.A. 89, 2713–2717 1557377 10.1073/pnas.89.7.2713 PMC48732

[26] Fantappie L., de Santis M., Chiarot E., Carboni F., Bensi G., Jousson O., Margarit I., and Grandi G. (2014) Antibody-mediated immunity induced by engineered Escherichia coli OMVs carrying heterologous antigens in their lumen. J. Extracell. Vesicles 3 10.3402/jev.v3.24015 PMC4131003 25147647

[27] Klock H. E., and Lesley S. A. (2009) The Polymerase Incomplete Primer Extension (PIPE) method applied to high-throughput cloning and site-directed mutagenesis. Methods Mol. Biol. 498, 91–103 18988020 10.1007/978-1-59745-196-3_6

[28] Gorg A., Postel W., and Gunther S. (1988) The current state of two-dimensional electrophoresis with immobilized pH gradients. Electrophoresis 9, 531–546 3072185 10.1002/elps.1150090913

[29] Bjellqvist B., Pasquali C., Ravier F., Sanchez J. C., and Hochstrasser D. (1993) A nonlinear wide-range immobilized pH gradient for two-dimensional electrophoresis and its definition in a relevant pH scale. Electrophoresis 14, 1357–1365 8137802 10.1002/elps.11501401209

[30] Oakley B. R., Kirsch D. R., and Morris N. R. (1980) A simplified ultrasensitive silver stain for detecting proteins in polyacrylamide gels. Anal. Biochem. 105, 361–363 6161559 10.1016/0003-2697(80)90470-4

[31] Hochstrasser D. F., Patchornik A., and Merril C. R. (1988) Development of polyacrylamide gels that improve the separation of proteins and their detection by silver staining. Anal. Biochem. 173, 412–423 3189819 10.1016/0003-2697(88)90208-4

[32] Sinha P., Poland J., Schnölzer M., and Rabilloud T. (2001) A new silver staining apparatus and procedure for matrix-assisted laser desorption/ionization-time of flight analysis of proteins after two-dimensional electrophoresis. Proteomics 1, 835–840 11503208 10.1002/1615-9861(200107)1:7<835::AID-PROT835>3.0.CO;2-2

[33] Hellman U., Wernstedt C., Góñez J., and Heldin C. H. (1995) Improvement of an “In-Gel” digestion procedure for the micropreparation of internal protein fragments for amino acid sequencing. Anal. Biochem. 224, 451–455 7710111 10.1006/abio.1995.1070

[34] Soskic V., Görlach M., Poznanovic S., Boehmer F. D., and Godovac-Zimmermann J. (1999) Functional proteomics analysis of signal transduction pathways of the platelet-derived growth factor beta receptor. Biochemistry 38, 1757–1764 10026255 10.1021/bi982093r

[35] Gharahdaghi F., Weinberg C. R., Meagher D. A., Imai B. S., and Mische S. M. (1999) Mass spectrometric identification of proteins from silver-stained polyacrylamide gel: a method for the removal of silver ions to enhance sensitivity. Electrophoresis 20, 601–605 10217175 10.1002/(SICI)1522-2683(19990301)20:3<601::AID-ELPS601>3.0.CO;2-6

[36] Bordier C. (1981) Phase separation of integral membrane proteins in Triton X-114 solution. J. Biol. Chem. 256, 1604–1607 6257680

[37] Piet J. R., Brouwer M. C., Exley R., van der Veen S., van de Beek D., and van der Ende A. (2012) Meningococcal factor H binding protein fHbpd184 polymorphism influences clinical course of meningococcal meningitis. PLoS ONE 7, e47973 23110143 10.1371/journal.pone.0047973 PMC3479137

[38] Brehony C., Wilson D. J. and Maiden M. C. (2009) Variation of the factor H-binding protein of Neisseria meningitidis. Microbiology 155, 4155–4169 19729409 10.1099/mic.0.027995-0 PMC2801853

[39] Cantini F., Savino S., Scarselli M., Masignani V., Pizza M., Romagnoli G., Swennen E., Veggi D., Banci L., and Rappuoli R. (2006) Solution structure of the immunodominant domain of protective antigen GNA1870 of Neisseria meningitidis. J. Biol. Chem. 281, 7220–7227 16407174 10.1074/jbc.M508595200

[40] Beernink P. T., Shaughnessy J., Pajon R., Braga E. M., Ram S., and Granoff D. M. (2012) The effect of human factor H on immunogenicity of meningococcal native outer membrane vesicle vaccines with over-expressed factor H binding protein. PLoS Pathog. 8, e1002688 22589720 10.1371/journal.ppat.1002688 PMC3349754

[41] Esposito V., Musi V., de Chiara C., Veggi D., Serruto D., Scarselli M., Kelly G., Pizza M., and Pastore A. (2011) Structure of the C-terminal domain of Neisseria heparin binding antigen (NHBA), one of the main antigens of a novel vaccine against Neisseria meningitidis. J. Biol. Chem. 286, 41767–41775 21965688 10.1074/jbc.M111.289314 PMC3308885

[42] Ellis T. N., Leiman S. A., and Kuehn M. J. (2010) Naturally produced outer membrane vesicles from Pseudomonas aeruginosa elicit a potent innate immune response via combined sensing of both lipopolysaccharide and protein components. Infect. Immun. 78, 3822–3831 20605984 10.1128/IAI.00433-10 PMC2937433

[43] Ferrari G., Garaguso I., Adu-Bobie J., Doro F., Taddei A. R., Biolchi A., Brunelli B., Giuliani M. M., Pizza M., Norais N., and Grandi G. (2006) Outer membrane vesicles from group B Neisseria meningitidis delta gna33 mutant: proteomic and immunological comparison with detergent-derived outer membrane vesicles. Proteomics 6, 1856–1866 16456881 10.1002/pmic.200500164

[44] Berlanda Scorza F., Doro F., Rodríguez-Ortega M. J., Stella M., Liberatori S., Taddei A. R., Serino L., Gomes Moriel D., Nesta B., Fontana M. R., Spagnuolo A., Pizza M., Norais N., and Grandi G. (2008) Proteomics characterization of outer membrane vesicles from the extraintestinal pathogenic Escherichia coli DeltatolR IHE3034 mutant. Mol. Cell. Proteomics 7, 473–485 17982123 10.1074/mcp.M700295-MCP200

[45] Teng C. H., Tseng Y. T., Maruvada R., Pearce D., Xie Y., Paul-Satyaseela M., and Kim K. S. (2010) NlpI contributes to Escherichia coli K1 strain RS218 interaction with human brain microvascular endothelial cells. Infect. Immun. 78, 3090–3096 20421385 10.1128/IAI.00034-10 PMC2897387

[46] Gurung M., Moon D. C., Choi C. W., Lee J. H., Bae Y. C., Kim J., Lee Y. C., Seol S. Y., Cho D. T., Kim S. I., and Lee J. C. (2011) Staphylococcus aureus produces membrane-derived vesicles that induce host cell death. PLoS ONE, 6, e27958 22114730 10.1371/journal.pone.0027958 PMC3218073

[47] Fletcher L. D., Bernfield L., Barniak V., Farley J. E., Howell A., Knauf M., Ooi P., Smith R. P., Weise P., Wetherell M., Xie X., Zagursky R., Zhang Y., and Zlotnick G. W. (2004) Vaccine potential of the Neisseria meningitidis 2086 lipoprotein. Infect. Immun. 72, 2088–2100 15039331 10.1128/IAI.72.4.2088-2100.2004 PMC375149

[48] Ozbudak E. M., Thattai M., Lim H. N., Shraiman B. I., and Van Oudenaarden A. (2004) Multistability in the lactose utilization network of Escherichia coli. Nature 427, 737–740 14973486 10.1038/nature02298

[49] Smits W. K., Kuipers O. P., and Veening J. W. (2006) Phenotypic variation in bacteria: the role of feedback regulation. Nat. Rev. Microbiol. 4, 259–271 16541134 10.1038/nrmicro1381

[50] van Winkelhoff A. J., and Slots J. (1999) Actinobacillus actinomycetemcomitans and Porphyromonas gingivalis in nonoral infections. Periodontol. 2000 20, 122–135 10522225 10.1111/j.1600-0757.1999.tb00160.x

[51] Henderson B., Ward J. M., and Ready D. (2010) Aggregatibacter (Actinobacillus) actinomycetemcomitans: a triple A* periodontopathogen? Periodontol. 2000 54, 78–105 20712635 10.1111/j.1600-0757.2009.00331.x

[52] Kieselbach T., et al (2015) Proteomics of Aggregatibacter actinomycetemcomitans outer membrane vesicles. PLoS ONE 10, e0138591 26381655 10.1371/journal.pone.0138591 PMC4575117

[53] Zijnge V., Kieselbach T., and Oscarsson J. (2012) Proteomics of protein secretion by Aggregatibacter actinomycetemcomitans. PLoS ONE 7, e41662 22848560 10.1371/journal.pone.0041662 PMC3405016

[54] Beernink P. T., Shaughnessy J., Braga E. M., Liu Q., Rice P. A., Ram S., and Granoff D. M. (2011) A meningococcal factor H binding protein mutant that eliminates factor H binding enhances protective antibody responses to vaccination. J. Immunol. 186, 3606–3614 21325619 10.4049/jimmunol.1003470 PMC3098282

[55] Moscatello D. K., Montgomery R. B., Sundareshan P., McDanel H., Wong M. Y., and Wong A. J. (1996) Transformational and altered signal transduction by a naturally occurring mutant EGF receptor. Oncogene 13, 85–96 8700557

[56] Choi B. D., Kuan C. T., Cai M., Archer G. E., Mitchell D. A., Gedeon P. C., Sanchez-Perez L., Pastan I., Bigner D. D., and Sampson J. H. (2013) Systemic administration of a bispecific antibody targeting EGFRvIII successfully treats intracerebral glioma. Proc. Natl. Acad. Sci. U.S.A., 110, 270–275 23248284 10.1073/pnas.1219817110 PMC3538214

[57] Del Vecchio C. A., Li G., and Wong A. J. (2012) Targeting EGF receptor variant III: tumor-specific peptide vaccination for malignant gliomas. Expert Rev. Vaccines 11, 133–144 22309662 10.1586/erv.11.177

[58] Beatty P. L., and Finn O. J. (2013) Preventing cancer by targeting abnormally expressed self-antigens: MUC1 vaccines for prevention of epithelial adenocarcinomas. Ann. N.Y. Acad. Sci. 1284, 52–56 23651193 10.1111/nyas.12108

[59] Kimura T., and Finn O. J. (2013) MUC1 immunotherapy is here to stay. Expert. Opin. Biol. Ther. 13, 35–49 22998452 10.1517/14712598.2012.725719

[60] Manning P. A., Beutin L., and Achtman M. (1980) Outer membrane of Escherichia coli: properties of the F sex factor traT protein which is involved in surface exclusion. J. Bacteriol. 142, 285–294 6989806 10.1128/jb.142.1.285-294.1980 PMC293949

[61] Drummelsmith J., and Whitfield C. (2000) Translocation of group 1 capsular polysaccharide to the surface of Escherichia coli requires a multimeric complex in the outer membrane. EMBO J. 19, 57–66 10619844 10.1093/emboj/19.1.57 PMC1171777

[62] Robinson L. S., Ashman E. M., Hultgren S. J., and Chapman M. R. (2006) Secretion of curli fibre subunits is mediated by the outer membrane-localized CsgG protein. Mol. Microbiol. 59, 870–881 16420357 10.1111/j.1365-2958.2005.04997.x PMC2838483

[63] Miroux B., and Walker J. E. (1996) Over-production of proteins in Escherichia coli: mutant hosts that allow synthesis of some membrane proteins and globular proteins at high levels. J. Mol. Biol. 260, 289–298 8757792 10.1006/jmbi.1996.0399

[64] Dubnau D., and Losick R. (2006) Bistability in bacteria. Mol. Microbiol. 61, 564–572 16879639 10.1111/j.1365-2958.2006.05249.x

[65] De Angelis G., Moschioni M., Muzzi A., Pezzicoli A., Censini S., Delany I., Lo Sapio M., Sinisi A., Donati C., Masignani V., and Barocchi M. A. (2011) The Streptococcus pneumoniae pilus-1 displays a biphasic expression pattern. PLoS ONE 6, e21269 21731688 10.1371/journal.pone.0021269 PMC3120856

[66] Goolab S., Roth R. L., van Heerden H., and Crampton M. C. (2015) Analyzing the molecular mechanism of lipoprotein localization in Brucella. Front. Microbiol. 6, 1189 26579096 10.3389/fmicb.2015.01189 PMC4623201

[67] Jose J., and Meyer T. F. (2007) The autodisplay story, from discovery to biotechnical and biomedical applications. Microbiol. Mol. Biol. Rev. 71, 600–619 18063719 10.1128/MMBR.00011-07 PMC2168652

[68] Ruiz N., Kahne D., and Silhavy T. J. (2009) Transport of lipopolysaccharide across the cell envelope: the long road of discovery. Nat. Rev. Microbiol. 7, 677–683 19633680 10.1038/nrmicro2184 PMC2790178

[69] Raetz C. R., and Whitfield C. (2002) Lipopolysaccharide endotoxins. Annu. Rev. Biochem. 71, 635–700 12045108 10.1146/annurev.biochem.71.110601.135414 PMC2569852

[70] Polissi A., and Sperandeo P. (2014) The lipopolysaccharide export pathway in Escherichia coli: structure, organization and regulated assembly of the Lpt machinery. Mar Drugs, 12, 1023–1042 24549203 10.3390/md12021023 PMC3944529

[71] Okuda S., and Tokuda H. (2011) Lipoprotein sorting in bacteria. Annu. Rev. Microbiol. 65, 239–259 21663440 10.1146/annurev-micro-090110-102859

[72] Dong H., Xiang Q., Gu Y., Wang Z., Paterson N. G., Stansfeld P. J., He C., Zhang Y., Wang W., and Dong C. (2014) Structural basis for outer membrane lipopolysaccharide insertion. Nature 511, 52–56 24990744 10.1038/nature13464

[73] Qiao S., Luo Q., Zhao Y., Zhang X. C., and Huang Y. (2014) Structural basis for lipopolysaccharide insertion in the bacterial outer membrane. Nature 511, 108–111 24990751 10.1038/nature13484

[74] Bishop R. E., (2014) Structural biology: Lipopolysaccharide rolls out the barrel. Nature 511, 37–38 24990738 10.1038/nature13508 PMC5007118

[75] Gu Y., Stansfeld P. J., Zeng Y., Dong H., Wang W., and Dong C. (2015) Lipopolysaccharide is inserted into the outer membrane through an intramembrane hole, a lumen gate, and the lateral opening of LptD. Structure 23, 496–504 25684578 10.1016/j.str.2015.01.001 PMC4353691

