# Supplementary material for: Some Gram-negative Lipoproteins Keep Their Surface Topology When Transplanted from One Species to Another and Deliver Foreign Polypeptides to the Bacterial Surface
Source: Mol Cell Proteomics. 2017 May 8;16(7):1348–64. doi: 10.1074/mcp.M116.065094 (PMC5500766; doi:10.1074/mcp.M116.065094)
Supplement: Supplemental Data [file supp_16_7_1348__index.html]

Some Gram-negative lipoproteins keep their surface topology when transplanted from one species to another and deliver foreign polypeptides to the bacterial surface — Some Gram-negative Lipoproteins Keep Their Surface Topology When Transplanted from One Species to Another and Deliver Foreign Polypeptides to the Bacterial Surface — Lipoprotein Sorting in Gram-negative Bacteria — Supplemental Data 

# Some Gram-negative Lipoproteins Keep Their Surface Topology When Transplanted from One Species to Another and Deliver Foreign Polypeptides to the Bacterial Surface

## Supplemental Data

- Supplemental figures (.pdf, 4.8 MB) - Supplemental figures
- Supplemental figures legends (.pdf, 69 KB) - Supplemental figures legends
- Supplemental tables (.pdf, 197 KB) - Supplemental tables
- Supplemental mass spec material (.docx, 42.3 MB) - Supplemental mass spec material
