# Supplementary figures and images for: Some Gram-negative Lipoproteins Keep Their Surface Topology When Transplanted from One Species to Another and Deliver Foreign Polypeptides to the Bacterial Surface
Source: Mol Cell Proteomics. 2017 May 8;16(7):1348–64. doi: 10.1074/mcp.M116.065094 (PMC5500766; doi:10.1074/mcp.M116.065094)

**Figure S1**

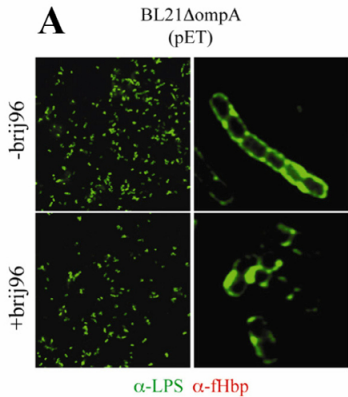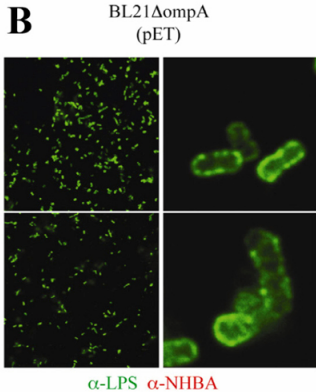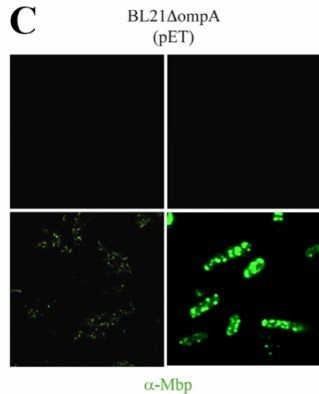

**Figure S2**

**A**

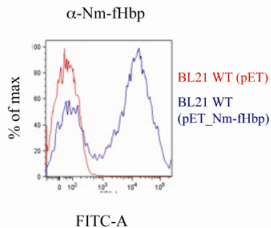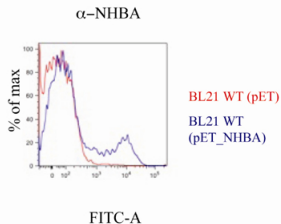

**B**

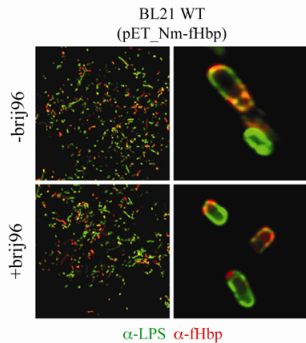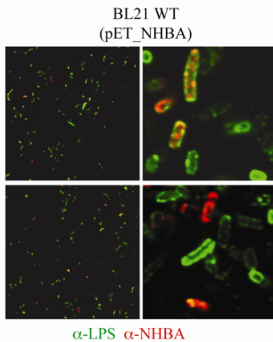

Figure S3

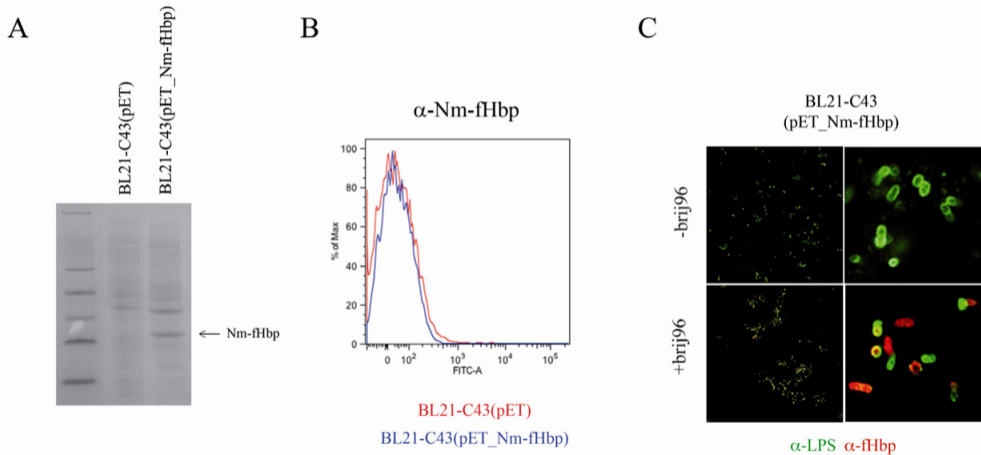

# Figure S4

**A**

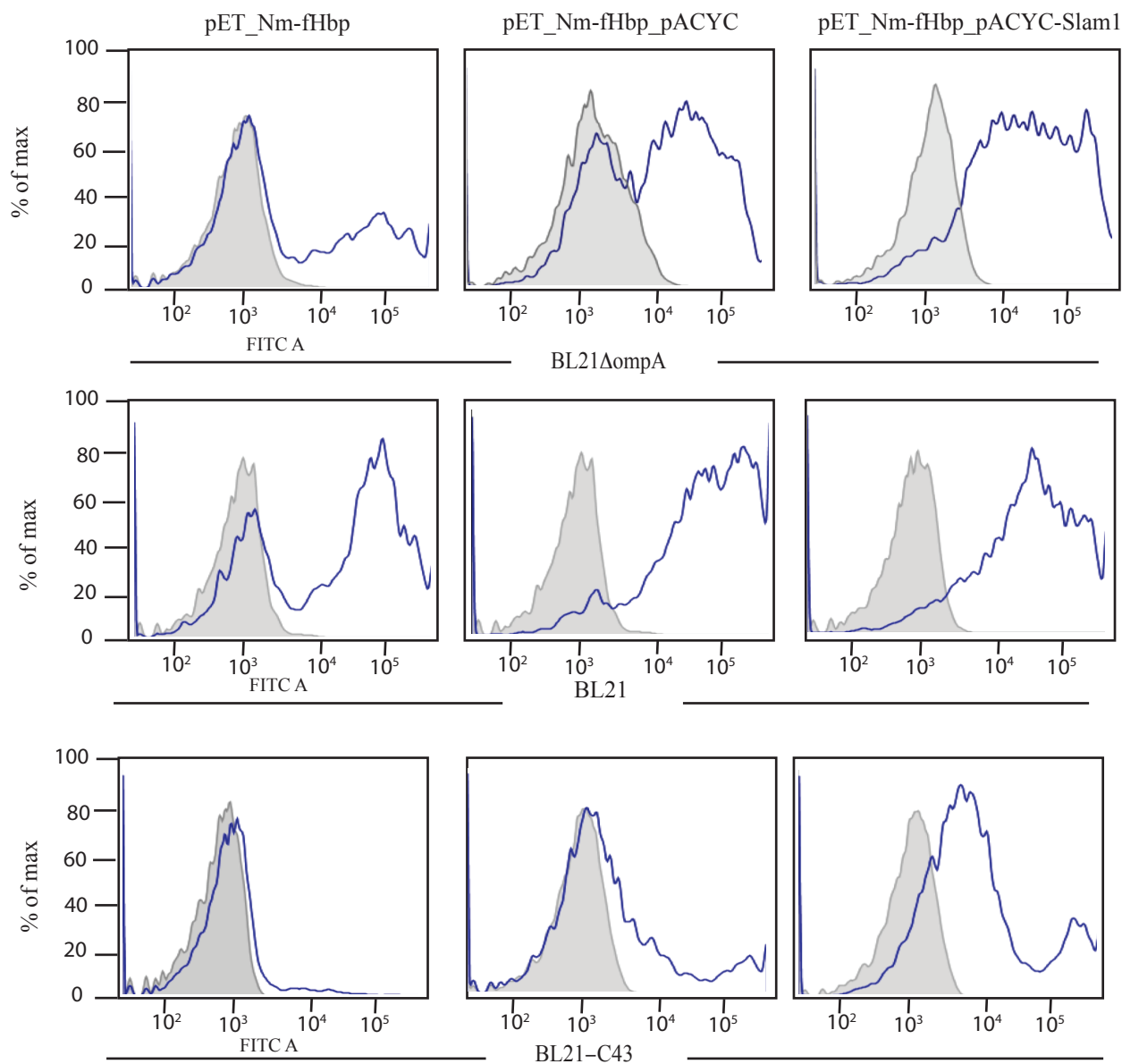

**B**

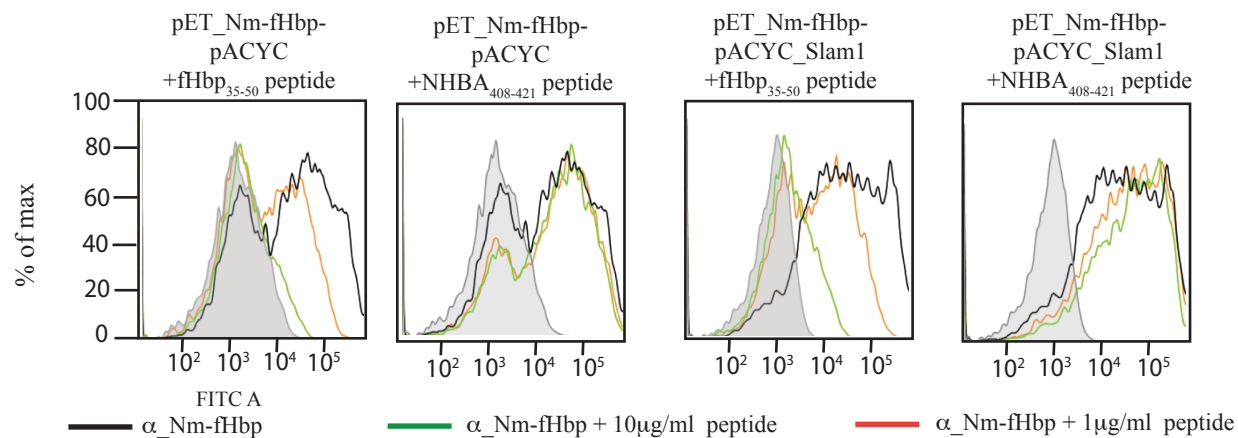

**Figure S5**

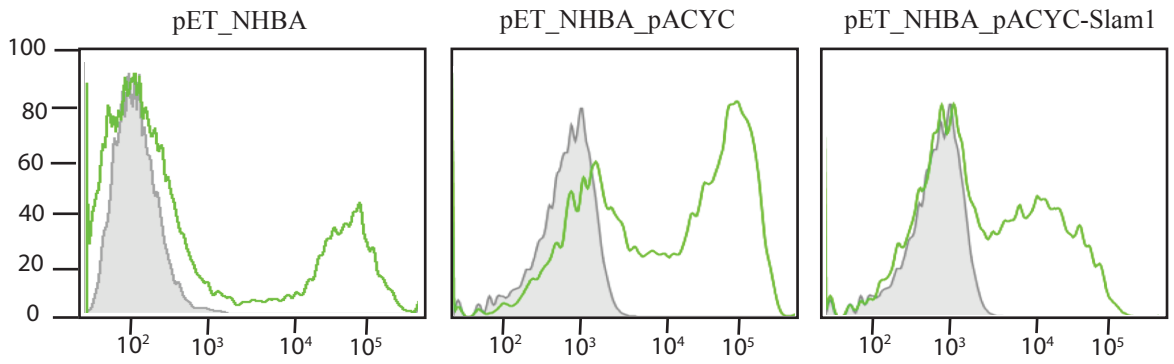

Supplement: Supplemental Data [file 10.1074_M116.065094_mcp.M116.065094-1.pdf]
