# Supplementary material for: Some Gram-negative Lipoproteins Keep Their Surface Topology When Transplanted from One Species to Another and Deliver Foreign Polypeptides to the Bacterial Surface
Source: Mol Cell Proteomics. 2017 May 8;16(7):1348–64. doi: 10.1074/mcp.M116.065094 (PMC5500766; doi:10.1074/mcp.M116.065094)
Supplement: Supplemental Data [file 10.1074_M116.065094_mcp.M116.065094-2.pdf]

Figure S1

Control of anti Nm-fHbp, anti-NHBA, anti-LPS and anti-Mbp antibody specificity on *E.coli* BL21*ΔompA* recipient strain. Bacterial cells from BL21*ΔompA*(pET) strain were fixed with 2% formaldehyde solution and incubated with specific antibodies in the presence (lower panels) or absence (upper panels) of membrane permeabilization by 0,1% Brij96. Anti Nm-fHbp or anti-NHBA antibody binding on the recipient strain was followed by incubating cells with alexa fluor 594-labelled anti-rabbit antibodies (red), while anti-LPS and anti-Mbp antibody binding was followed using alexa fluor 488-labelled anti-mouse antibody (green). In A, B, C, each right panel represents a magnification of the corresponding left panel. **A**) Cells were incubated with rabbit anti-Nm-fHbp and mouse anti-LPS; **B**) Cells were incubated antibodies rabbit anti-NHBA and mouse anti-LPS antibodies; **C)** Cells were incubated and mouse anti-Mbp antibody.

Figure S2

Assessment of Nm-fHbp and NHBA localization by FACS analysis and confocal microscopy in BL21 WT strain. **A)** After IPTG induction bacterial cells from BL21(pET) (red histograms), BL21(pET_Nm-fHbp) and BL21(pET_NHBA) (blue histograms) strains were incubated first with anti-Nm-fHbp and anti-NHBA specific antibodies, respectively, and subsequently with FITC-labelled anti-rabbit secondary antibodies. Fluorescence was measured by Fluorescence-activated Cell Sorting (FACS). **B)** Bacterial cells from BL21 (pET_Nm-fHbp) (left) and BL21(pET_NHBA) (right) strains were fixed with 2% formaldehyde solution and incubated with anti-Nm-fHbp and anti-NHBA antibodies and with a mouse

monoclonal antibody specific for the core region of LPS. To analyze an intracellular staining samples were first treated with 0,1% Brj96 5 min at room temperature RT (+brij96). The binding of anti-Nm-fHbp and anti-NHBA antibodies was visualized with alexa fluor 594-labelled anti-rabbit antibodies (red), while anti-LPS antibody binding was followed using alexa fluor 488-labelled anti-mouse antibody (green).

Figure S3

Assessment of localization of Nm-fHbp expressed in *E. coli* BL21-C43 strain by FACS analysis and confocal microscopy. **A)** SDS-PAGE analysis of total cell extracts from BL21-C43 and BL21-C43 (pET_Nm-fHbp) strains. **B)** BL21-C43 (pET_Nm-fHbp) (blue histogram) and BL21-C43 (pET) (red histogram) strains were incubated first with rabbit anti-Nm-fHbp specific antibody, and subsequently with FITC-labelled anti-rabbit secondary antibodies. Fluorescence was measured by Fluorescence-activated Cell Sorting (FACS). **C)** Bacterial cells from BL21-C43 (pET_Nm-fHbp) strain were fixed with 2% formaldehyde solution and incubated with rabbit anti-Nm-fHbp antibody in the presence of a mouse monoclonal antibody specific for the core region of LPS in the presence (lower panels) or absence (upper panels) of permeabilization treatment with 0,1% Brij96. The binding of anti-Nm-fHbp antibody was visualized with alexa fluor 594-labelled anti-rabbit antibodies (red), while anti-LPS antibody binding was followed using alexa fluor 488-labelled anti-mouse antibody (green). Each right panel represents a magnification of the corresponding left panel.

Figure S4

Effect of Slam1 on surface localization of Nm-fHbp. **A)** BL21*ΔompA*, BL21 WT and BL21-C43 strains transformed with pET_Nm-fHbp, pET_Nm-fHbp+pACYC and pET_Nm-fHbp+pACYC_slam1 plasmids were grown in LB at 37°C. At OD_{600} = 0.5, 1 mM IPTG was added and after 2 hours bacterial cells were collected by centrifugation and incubated with anti-Nm-fHbp specific antibodies and subsequently with FITC-labelled anti-rabbit secondary antibodies. Fluorescence was measured by Fluorescence-activated Cell Sorting (FACS). **B)** BL21*ΔompA*(pET_Nm-fHbp)(pACYC) and BL21*ΔompA*(pET_Nm-fHbp)(pACYC_slam1) strains were grown as described above and the binding of anti-Nm-fHbp antibodies was followed by FACS in the presence or absence of different concentration of either fHbp-derived $peptide_{35-50}$ or NHBA-derived $peptide_{408-421}$

Figure S5

Effect of Slam1 on surface localization of NHBA in BL21*ΔompA.* Bacterial cells co-transformed with either pET_NHBA + pACYC or pET_NHBA + pACYC_slam1 plasmids were grown in LB at 37°C. At OD_{600} = 0.5, 1 mM IPTG was added and after 2 hours bacterial cells were collected by centrifugation and incubated with anti-NHBA specific rabbit antibodies and subsequently with FITC-labelled anti-rabbit secondary antibodies. Fluorescence was measured by Fluorescence-activated Cell Sorting (FACS).
