# Supplementary material for: Some Gram-negative Lipoproteins Keep Their Surface Topology When Transplanted from One Species to Another and Deliver Foreign Polypeptides to the Bacterial Surface
Source: Mol Cell Proteomics. 2017 May 8;16(7):1348–64. doi: 10.1074/mcp.M116.065094 (PMC5500766; doi:10.1074/mcp.M116.065094)
Supplement: Supplemental Data [file 10.1074_M116.065094_mcp.M116.065094-3.pdf]

Table S1: Proteins separated by 2D gels analysis and identified by MALDI-TOF analysis

Spot	Protein description	AC[a]	Mascot search results			Localization
			Score	N. of peptides	Seq. coverage (%)	
**2**	Putative lipoprotein AcfD homolog	ACFD_ECOLI	76	9/13	6	Inner membrane
**10**	Cellulose synthase operon protein C	BCSC_ECOLI	112	10/11	8	Outer membrane
**15**	Protease 3	PTRA_ECO57	112	11/15	14	Periplasm
**25**	LPS-assembly protein LptD	LPTD_ECO57	113	11/18	17	Outer membrane
**26**	LPS-assembly protein LptD<br>Outer membrane protein assembly factor BamA	LPTD_ECO57<br>BAMA_ECO24	216<br>112	24/49<br>16/49	42<br>27	Outer membrane<br>Outer membrane
**27**	Outer membrane protein assembly factor BamA	BAMA_ECO24	371	34/44	53	Outer membrane
**28**	Maltoporin	LAMB_ECOBW	162	16/32	52	Outer membrane
**32**	Ferrienterobactin receptor	FEPA_ECOLI	108	8/9	15	Outer membrane
**33**	Ferrienterobactin receptor	FEPA_ECOLI	247	24/35	40	Outer membrane
**34**	Ferrienterobactin receptor	FEPA_ECOLI	278	30/52	52	Outer membrane
**37**	Ferrichrome-iron receptor	FHUA_ECOLI	116	8/8	13	Outer membrane
**38**	Ferrichrome-iron receptor	FHUA_ECOLI	184	20/34	33	Outer membrane
**39**	Probable TonB-dependent receptor YncD<br>Ferrichrome-iron receptor<br>Ferrienterobactin receptor	YNCD_ECOLI<br>FHUA_ECOLI<br>FEPA_ECOLI	162<br>106<br>68	28/96<br>23/96<br>15/96	52<br>35<br>32	Outer membrane,<br>Outer membrane,<br>Outer membrane
**40**	Ferrichrome-iron receptor	FHUA_ECOLI	136	10/11	18	Outer membrane
**42**	Fe(3+) dicitrate transport protein FecA	FECA_ECOLI	148	11/12	18	Outer membrane
**47**	Periplasmic beta-glucosidase	BGLX_ECOLI	100	8/10	13	Periplasm
**48**	Periplasmic beta-glucosidase	BGLX_ECOLI	198	18/26	30	Periplasm
**53**	Tail-specific protease	PRC_ECOLI	154	14/19	24	Inner membrane
**55**	FhuE receptor	FHUE_ECOLI	104	7/7	13	Outer membrane
**57**	Penicillin-binding protein activator LpoA	LPOA_ECOLI	127	9/10	19	Outer membrane
**58**	Penicillin-binding protein activator LpoA	LPOA_ECOLI	225	18/24	34	Outer membrane
**59**	Penicillin-binding protein activator LpoA	LPOA_ECOLI	330	31/46	52	Outer membrane
**60**	Penicillin-binding protein activator LpoA	LPOA_ECOLI	147	12/16	22	Outer membrane
**64**	Penicillin-binding protein activator LpoA	LPOA_ECOLI	121	8/8	16	Outer membrane
**65**	Colicin I receptor	CIRA_ECOLI	123	8/8	18	Outer membrane
**68**	Outer membrane protein assembly factor BamA	BAMA_ECO24	104	9/12	12	Outer membrane
**78**	Alpha-amylase	AMY1_ECOLI	140	11/14	15	Outer membrane
**79**	2',3'-cyclic-nucleotide 2'-phosphodiesterase/3'-nucleotidase	CPDB_ECOLI	127	9/10	16	Periplasm
**80**	2',3'-cyclic-nucleotide 2'-phosphodiesterase/3'-nucleotidase	CPDB_ECOLI	171	13/16	24	Periplasm
**88**	Soluble lytic mureintransglycosylase	SLT_ECO57	208	17/19	24	Periplasm
**100**	Protein UshA	USHA_ECOLI	138	9/9	17	Periplasm
**102**	Putative periplasmictrehalase	TREA_ECO57	125	8/8	16	Periplasm
**103**	Uncharacterized sulfataseYdeN	YDEN_ECOLI	79	6/9	18	Outer membrane
**105**	Uncharacterized sulfataseYdeN	YDEN_ECOLI	146	9/9	23	Outer membrane
**109**	Glucans biosynthesis protein D	OPGD_ECO24	215	15/17	26	Periplasm
**110**	Periplasmicoligopeptide-binding protein	OPPA_ECOLI	135	10/13	20	Periplasm
**111**	Periplasmicoligopeptide-binding protein	OPPA_ECOLI	264	22/35	42	Periplasm
**115**	Polysialic acid transport protein KpsD	OPGG_ECO24	110	7/7	20	Periplasm
**114**	Glucans biosynthesis protein G	KPSD1_ECOLX	139	11/16	24	Periplasm
**116**	Glucans biosynthesis protein G	OPGG_ECO24	232	19/24	41	Periplasm

**120**	Glycerophosphoryldiesterphosphodiesterase	GLPQ_ECOLI	103	8/13	27	Periplasm
**137**	Periplasmic serine endoproteaseDegP	DEGP_ECO57	134	8/8	25	Periplasm
**138**	Periplasmic serine endoproteaseDegP	DEGP_ECO57	176	15/21	40	Periplasm
**146**	Outer membrane protein TolC	TOLC_ECOLI	206	16/24	43	Outer membrane
**147**	Outer membrane protein TolC	TOLC_ECOLI	99	6/6	17	Outer membrane
**150**	Outer membrane protein TolC	TOLC_ECOLI	155	12/18	31	Outer membrane
**156**	Protein YhjJ	YHJJ_ECOLI	185	13/16	34	Periplasm
**157**	Periplasmic pH-dependent serine endoproteaseDegQ	DEGQ_ECOLI	218	16/20	46	Periplasm
**158**	Cell division proteinFtsP	FTSP_ECOLI	180	15/22	33	Periplasm
**164**	Maltoporin	LAMB_ECO24	119	11/17	32	Outer membrane
**165**	Maltoporin	LAMB_ECOBW	144	14/26	44	Outer membrane
**166**	Maltoporin	LAMB_ECOBW	168	19/37	46	Outer membrane
**167**	Maltoporin	LAMB_ECOBW	181	20/40	55	Outer membrane
**168**	Maltoporin	LAMB_ECOBW	203	20/37	52	Outer membrane
**169**	Maltoporin	LAMB_ECO24	82	6/10	23	Outer membrane
**170**	Rare lipoprotein A	RLPA_ECOLI	166	13/24	33	Outer membrane
**171**	Rare lipoprotein A	RLPA_ECOLI	147	11/18	22	Outer membrane
**183**	Chaperone SurA	SURA_ECO57	234	24/40	55	Periplasm
**192**	Putative acyl-CoA thioester hydrolase YbhC	YBHC_ECOLI	116	11/17	27	Outer membrane
**193**	Elongation factor Tu 1<br>Putative acyl-CoA thioester hydrolase YbhC	EFTU1_ECO24<br>YBHC_ECOLI	107<br>89	10/26<br>9/26	37<br>27	Cytoplasm<br>Outer membrane
**194**	Glucose-1-phosphatase	AGP_ECOLI	102	9/20	22	Periplasm
**195**	Glucose-1-phosphatase	AGP_ECOLI	135	13/27	30	Periplasm
**196**	Protein TolB	TOLB_ECO24	151	9/9	23	Periplasm
**199**	Protein TolB	TOLB_ECO24	207	17/27	46	Periplasm
**202**	Protein TolB	TOLB_ECO24	215	17/25	49	Periplasm
**205**	Protein TolB	TOLB_ECO24	117	7/7	18	Periplasm
**210**	Periplasmic serine endoproteaseDegP	DEGP_ECO57	125	8/9	25	Periplasm
**211**	Periplasmic serine endoproteaseDegP	DEGP_ECO57	134	8/8	21	Periplasm
**225**	Maltose-bindingperiplasmicprotein<br>Glycerophosphoryl diester phosphodiesterase<br>Erythronate-4-phosphate dehydrogenase	MALE_ECO57<br>GLPQ_ECOLI<br>PDXB_ECOBW	144<br>78<br>68	15/49<br>11/49<br>9/49	49<br>38<br>32	Periplasm,<br>Periplasm,<br>Cytoplasm
**226**	Maltose-bindingperiplasmicprotein<br>Glycerophosphoryl diester phosphodiesterase	MALE_ECO57<br>GLPQ_ECOLI	136<br>71	17/66<br>12/66	52<br>38	Periplasm,<br>Periplasm,
**227**	Maltose-bindingperiplasmicprotein	MALE_ECO57	112	7/8	23	Periplasm
**242**	UncharacterizedproteinYncE	YNCE_ECOLI	126	8/10	31	Outer membrane
**244**	Outer membrane protein assembly factor BamB	BAMB_ECOLI	222	19/30	66	Outer membrane
**245**	Outer membrane protein F	OMPF_ECOLI	189	13/20	56	Outer membrane
**246**	Outer membrane protein assembly factor BamC	BAMC_ECOLI	110	7/9	27	Outer membrane
**247**	Outer membrane protein assembly factor BamC	BAMC_ECOLI	231	20/33	62	Outer membrane
**248**	Outer membrane protein assembly factor BamC	BAMC_ECOLI	117	7/8	27	Outer membrane
**249**	Iron uptake system component EfeO	EFEO_ECO57	102	6/6	16	Periplasm
**263**	Maltose operon periplasmic protein	MALM_ECOLI	138	10/14	25 C-frag.	Periplasm
**264**	Glycine betaine/prolinebetaine-binding periplasmic protein	PROX_ECO57	101	6/7	24	Periplasm
**266**	Lipoprotein NlpI	NLPI_ECO57	188	10/10	38	Inner membrane *
**267**	Long-chain fatty acid transport protein	FADL_ECO57	89	7/12	19	Outer membrane
**268**	Phospholipase A1	PA1_ECO57	106	7/11	33	Outer membrane
**273**	Cell division coordinator CpoB	CPOB_ECOLI	104	8/13	23	Periplasm
**272**	Beta-lactamase TEM	BLAT_ECOLX	151	11/16	43	Periplasm
**274**	Beta-lactamase TEM	BLAT_ECOLX	280	22/36	70	Periplasm

**275**	Beta-lactamase TEM	BLAT_ECOLX	148	8/8	32	Periplasm
**284**	Probable L,D-transpeptidaseYbiS	YBIS_ECO57	133	8/10	46	Periplasm
**286**	FKBP-type peptidyl-prolylcis-trans isomeraseFkpA	FKBA_ECO57	154	10/15	44	Periplasm
**293**	Glutamate/aspartate import solute-binding protein	GLTI_ECOLI	100	6/7	21	Periplasm
**301**	Lipoprotein NlpE	NLPE_ECOLI	118	8/10	42	Outer membrane
**302**	Nucleoside-specific channel-forming protein tsx	TSX_ECO57	107	6/7	26	Outer membrane
**305**	Nucleoside-specific channel-forming protein tsx	TSX_ECO57	137	11/23	42	Outer membrane
**306**	MltA-interacting protein	MIPA_ECOLI	112	8/13	47	Outer membrane
**307**	MltA-interacting protein	MIPA_ECOLI	139	11/18	56	Outer membrane
**312**	MetalloproteaseLoiP	LOIP_ECOLI	125	7/8	34	Outer membrane
**308**	Outer membrane protein assembly factor BamD	BAMD_ECO57	134	7/7	29	Outer membrane
**313**	Outer membrane protein assembly factor BamD	BAMD_ECO57	264	22/34	75	Outer membrane
**315**	Penicillin-binding protein activator LpoB	LPOB_ECO57	81	5/5	29	Outer membrane
**318**	ABC transporter arginine-binding protein 1	ARTJ_ECOLI	103	7/9	31	Periplasm
**324**	Probable phospholipid-binding protein MlaC	MLAC_ECOLI	111	7/10	33	Periplasm
**327**	Quorum-sensing regulator protein G	QSEG_ECO57	119	7/9	24	Outer membrane
**333**	Outer-membrane lipoprotein LolB	LOLB_ECOL6	59	3/3	18 N-frag.	Outer membrane
**341**	LPS-assembly lipoprotein LptE	LPTE_ECO57	88	5/6	24	Outer membrane
**364**	Uncharacterized lipoprotein YajG	YAJG_ECOL6	140	9/12	30	Inner membrane *
**365**	Outer membrane protein slp	SLP_ECOLI	63	3/3	26	Outer membrane
**369**	Uncharacterized protein YraP	YRAP_ECO57	193	15/24	62	Periplasm

a) Accession number in UniProtKB.

Table S2: Bacterial strains used in this study

Strain name	Plasmid presence	Reference
BL21(DE3)*ΔompA*	none	[1]
BL21(DE3)*ΔompA*(pET)	pET21b	[1]
BL21(DE3)*ΔompA(*pET21_Nm-fHbp)	pET21_Nm-fHbp	This study
BL21(DE3)*ΔompA(*pET21_NHBA)	pET21_NHBA	This study
BL21(DE3)*ΔompA(*pET21_Nm-(ΔGly)fHbp)	pET21_Nm-(ΔGly)fHbp	This study
BL21(DE3)*ΔompA(*pET21_Nm-(Ala_4)fHbp)	pET21_Nm-(Ala_4)fHbp	This study
BL21(DE3)*ΔompA*(pET21_Nm-fHbpDomA)	pET21_Nm-fHbpDomA	This study
BL21(DE3)*ΔompA*(pET21_Nm-fHbpDomAB)	pET21_Nm-fHbpDomAB	This study
BL21(DE3)*ΔompA*(pET21_Nm-fHbp_C>A)	pET21_Nm-fHbp_C>A	This study
BL21(DE3)*ΔompA*(pET21_NHBA_C>A)	pET21_NHBA_C>A	This study
BL21(DE3)*ΔompA*(pET21_Ag-fHbp)	pET21_Ag-fHbp	This study
BL21(DE3)*ΔompA*(pET21_Nm-fHbp-vIII)	pET21_Nm-fHbp-vIII	This study
BL21(DE3)*ΔompA*(pET21_NHBA-vIII)	pET21_NHBA-vIII	This study
BL21(DE3)*ΔompA*(pET21_Nm-fHbpDomA-MUC1)	pET21_Nm-fHbpDomA-MUC1	This study
BL21(DE3)-C43	None	[2, 3]
BL21(DE3)-C43(pET)	pET21b	This study
BL21(DE3)-C43(pET21_Nm-fHbp)	pET21_Nm-fHbp	This study
BL21(DE3) WT	None	[4]
BL21(DE3) WT(pET)	pET21b	This study
BL21(DE3) WT*(*pET21_Nm-fHbp)	pET21_Nm-fHbp	This study
BL21(DE3) WT*(*pET21_NHBA)	pET21_NHBA	This study
BL21(DE3)*ΔompA(*pACYC_slam1, pET21_Nm-fHbp)	pACYC_slam1 and pET21_Nm-fHbp	This study
BL21(DE3) WT*(*pACYC_slam1, pET21_Nm-fHbp)	pACYC_slam1 and pET21_Nm-fHbp	This study
BL21(DE3)-C43*(*pACYC_slam1, pET21_Nm-fHbp)	pACYC_slam1 and pET21_Nm-fHbp	This study

BL21(DE3)*ΔompA(*pACYC pET21_Nm-fHbp)	pACYC and pET21_Nm-fHbp	This study
BL21(DE3) WT*(*pACYC, pET21_Nm-fHbp)	pACYC and pET21_Nm-fHbp	This study
BL21(DE3)-C43*(*pACYC, pET21_Nm-fHbp)	pACYC and pET21_Nm-fHbp	This study
BL21(DE3)*ΔompA(*pACYC_slam1 pET21_NHBA)	pACYC_slam1 and pET21_Nm-NHBA	This study
BL21(DE3)*ΔompA(*pACYC pET21_NHBA)	pACYC and pET21_Nm-NHBA	This study

Table S3 - Oligonucleotides used in this study

Name	Sequence
fHbp-F	GGAGATATACATATGGTGAATCGAACTGCCTTCTGCTGCC
fHbp-R	GTGATGGTGATGTTATTATTGCTTGGCGGCAAGGC
NHBA-F	GGAGATATACATATGTTTAAACGCAGCGTAATC
NHBA-R	GTGATGGTGATGTTATCAATCCTGCTCTTTTTTG
pet-R	CATATGTATATCTCCTTCTTAAAGTTAAAC
pET-ΔHIS-F	TAACATCACCATCACCATCACGATTACAAAGA
fHbpΔgly-F	AGCAGCGTCGCCGCCGACATCGGTG
fHbpΔgly-R	GGCGACGCTGCTGCAGGCGGTCAG
fHbpGly>ALA-F	GCGGCCGCGGCCGTCGCCGCCGACATCGGTG
fHbpGly>ALA-R	GGCCGCGGCCGCGCTGCTGCAGGCGGTCAG
fHbpΔdomB/domC-R	GTGATGGTGATGTTACACCTCTCAAGGTTCATATGTTTGTT
fHbpΔdomC-R	GTGATGGTGATGTTATCCGCCGGCATCGTCTG
NHBA-C>A-R	GGCAAAAATACAAGCCATTGCGATTACGCTGCG
NHBAC>A-F	GCTTGTATTTTTGCCCTTTCAGCCgcCGGGGGC
fHbpC>A-R	TATCAGGGCAGTGGTCAGAGAAAGGCAGCAGAA
fHbpC>A-F	ACCACTGCCCTGATTCTGACCGCCGCC AGCAGC
pET2-R	CATATGTATATCTCCTTCTTAAAGTTAAACAAAATTATTTC
pETHIS-F	CATCACCATCACCATCACTAAGATTACAAAGACGATGATGACAAGtga
AgfHbp-F	GAAGGAGATATACAT ATG GTT TAC CCT GTT ATA ACG
AgfHbp-R	ATGGTGATGGTGATGTTCTTT TTTACCTGCCAA ACC
Nohisflag	TAACATCACCATCACCATCACGATTACAAAGA
fHbp-R2	TTATTGCTTGGCGGCAAGGC
vIII-R	GTGATGGTGATGTTATTAGCCGGAATGGTCGGTAACCAC
vIII-F	CTTGCCGCCAAGCAAGGTTCCCTGGAAGAAAAGAAGGG
NHBA-VIII-F1	CCGGCAAAAAAGAGCAGGAT ggttccctggaagaaaagaaggg

NHBA-VIII-R1	GTGATGGTGATGTTA gccggaatggtcggtaaccac
NHBA-VIII-R	ATCCTGCTCTTTTTTGCCGG
NHBA-VIII-F	TAACATCACCATCACCATCACGATTACAAAGA
R-MUCFH	GTGATGGTGATGGTGATGTTAATGCGCCGGCGGAGC
F-MUCDomA	CAAGTATACAAACAAGGGGTGACGAGCGCG
FHBP-F1	TAACATCACCATCACCATCACGATTACAAAGA
FHBPDA-R	TTGTTTGTATACTTGGAACTCTCCACTCTC
F1-MUC	GGGGTGACGAGCGCGCCAGATACACGTCCGGCTCCT
R1-MUC	ATGCGCCGGCGGGGCCGTCGAGCCAGGAGCCGGACG
F2-MUC	CCGCCGGCGCATGGAGTAACGTCAGCACCAGACACGCGCCCG
R2-MUC	GTGGGCAGGGGGAGCGGTGGATCCCGGTGCCGGGCGCGTGTC
F3-MUC	CCCCCTGCCCACGGTGTTACTAGTGCGCCCGATACCCGTCCA
R3-MUC	ATGCGCCGGCGGCGCGGTGGAGCCCGGTGCTGGACGGGTATC
F4-MUC	CCGCCGGCGCATGGAGTCACGTCAGCACCGGACACTCGTCCA
R4-MUC	GTGTGCTGGAGGCGCGGTTGAACCCGGGGCTGGACGAGTGTC
F5-MUC	CCTCCAGCACACGGCGTCACCTCAGCTCCAGATACGCGCCCG
R5-MUC	ATGTGCCGGCGGAGCGGTACTGCCTGGGGCCGGGCGCGTATC
slam-F	ATAAGGAGATATACC ATGGTTATTTTTTATTTTTGTGGGAAGAC
slam-R	TGATGGCTGCTGCCTTAGAACGTTTTATTAAACTCGACAAACGCC
pacyc-F	TAAGGCAGCAGCCATCACCATCATCACCAC
pacyc-R	CATGGTATATCTCCTTATTAAAGTTAAAC
pacyc-up1	GGATCTCGACGCTCTCCCT
pacyc-down1	GATTATGCGGCCGTGTACAA

1. Fantappie, L., et al., *Antibody-mediated immunity induced by engineered Escherichia coli OMVs carrying heterologous antigens in their lumen.* J Extracell Vesicles, 2014. **3**.
2. Dubnau, D. and R. Losick, *Bistability in bacteria.* Mol Microbiol, 2006. **61**(3): p. 564-72.
3. Wagner, S., et al., *Tuning Escherichia coli for membrane protein overexpression.* Proc Natl Acad Sci U S A, 2008. **105**(38): p. 14371-6.
4. Studier, F.W. and B.A. Moffatt, *Use of bacteriophage T7 RNA polymerase to direct selective high-level expression of cloned genes.* J Mol Biol, 1986. **189**(1): p. 113-30.
