# Supplementary material for: Some Gram-negative Lipoproteins Keep Their Surface Topology When Transplanted from One Species to Another and Deliver Foreign Polypeptides to the Bacterial Surface
Source: Mol Cell Proteomics. 2017 May 8;16(7):1348–64. doi: 10.1074/mcp.M116.065094 (PMC5500766; doi:10.1074/mcp.M116.065094)
Supplement: Supplemental Data [file 10.1074_M116.065094_mcp.M116.065094-4.docx]

**Spectra, mass lists, and MASCOT searching results of the identified proteins listed in Table S1**

**Name and release of sequence database actually searched: SwissProt 2016_07 (551705 sequences; 197114987 residues)**

Spot N. 2 (ACFD_ECOLI)

m/z

799.386

910.516

942.533

1108.604

1141.579

1152.639

1240.680

1268.719

1459.704

1484.770

1487.849

2120.170

2420.357


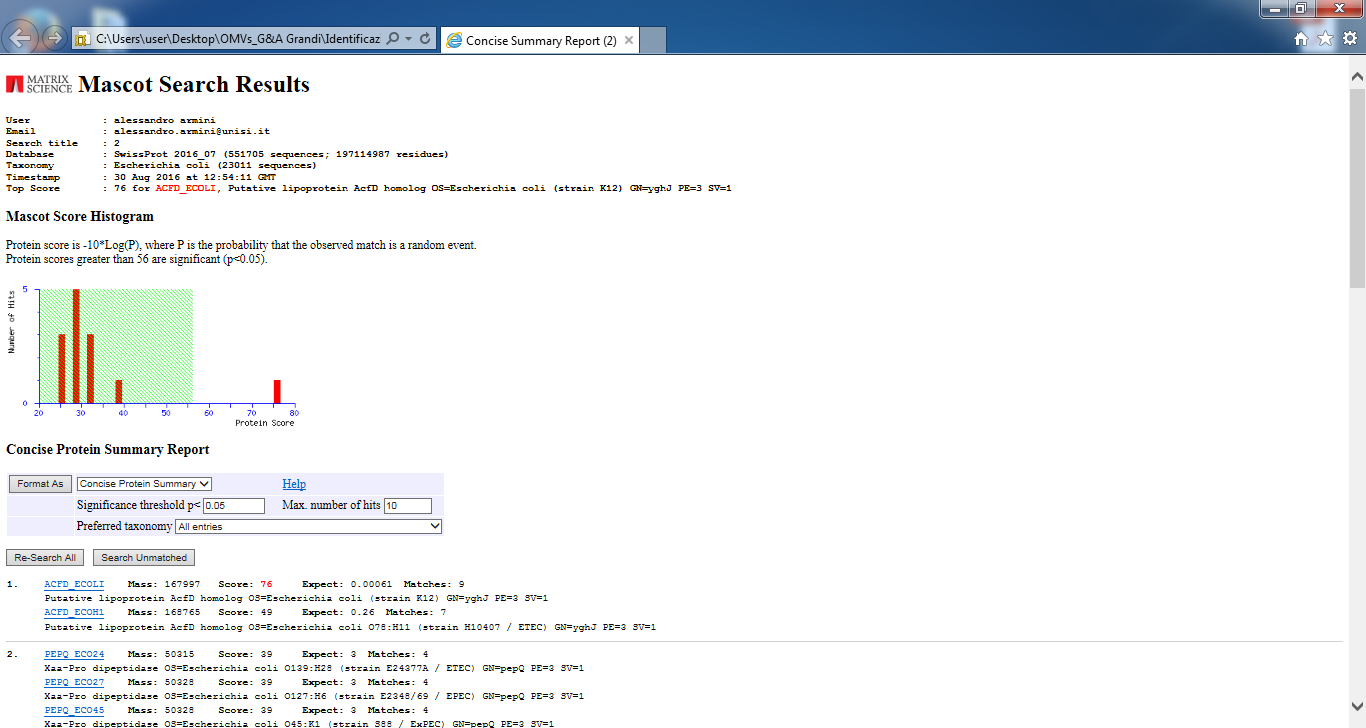


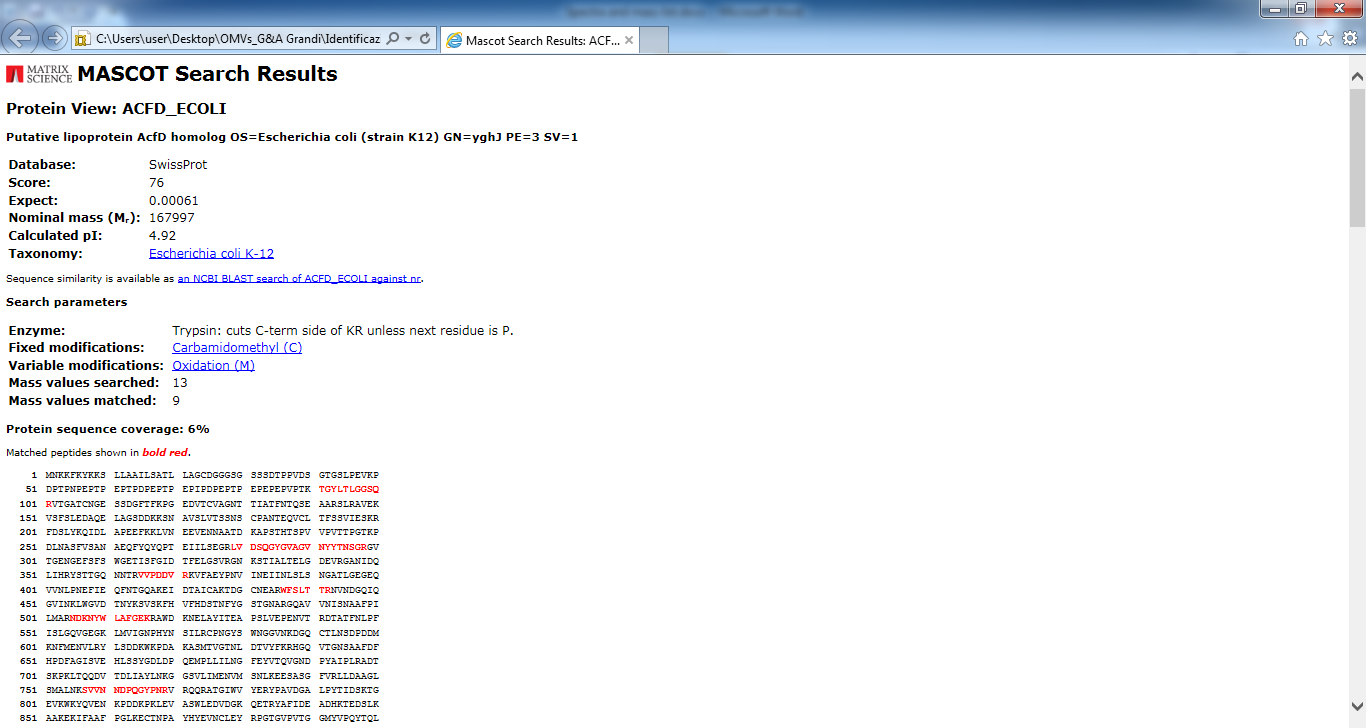

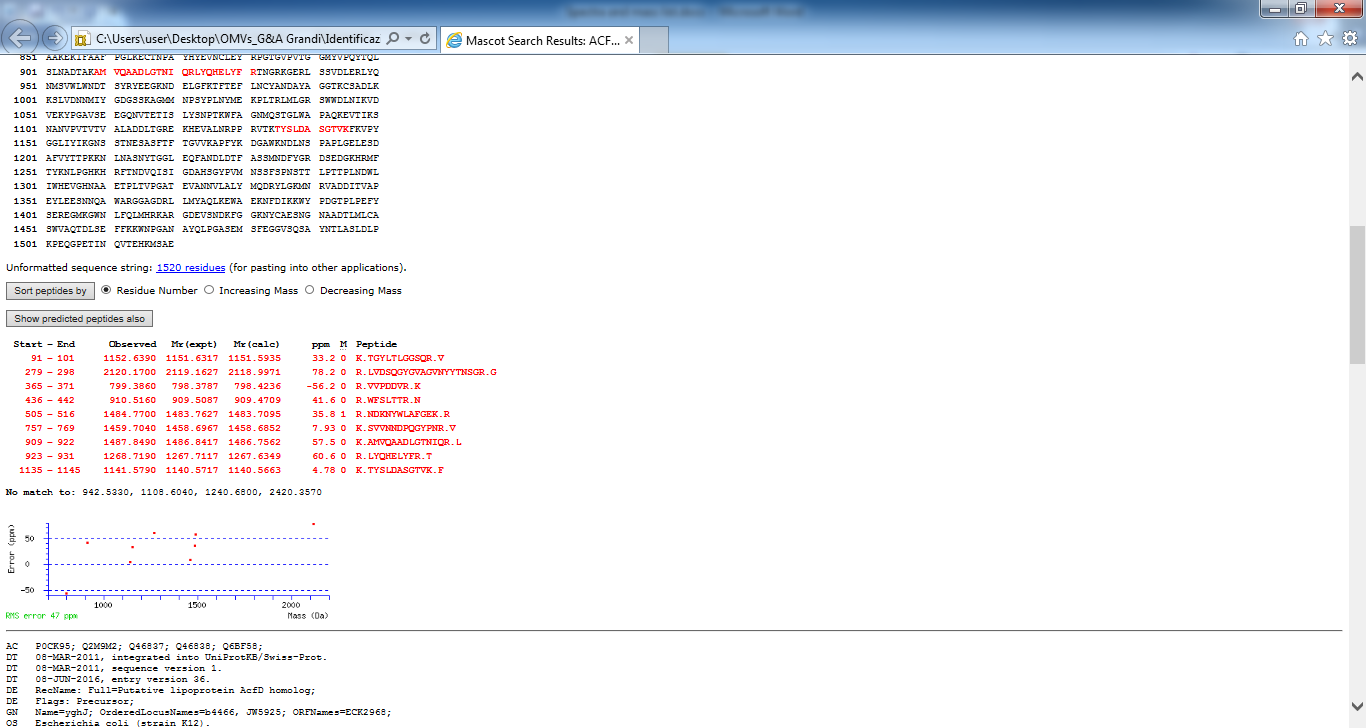


Spot N. 10 (BCSC_ECOLI)

m/z

762.410

917.440

932.464

971.489

1014.573

1124.625

1301.639

1406.743

1481.775

1583.820

1631.717


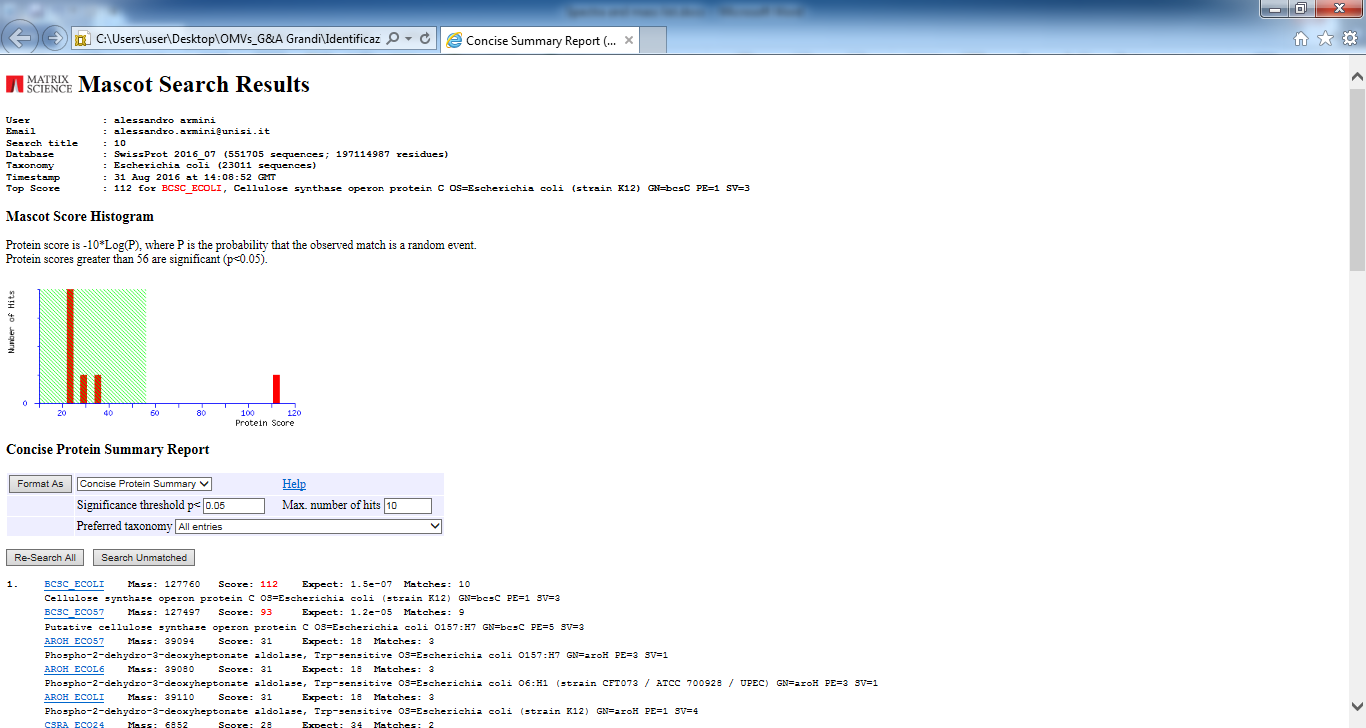


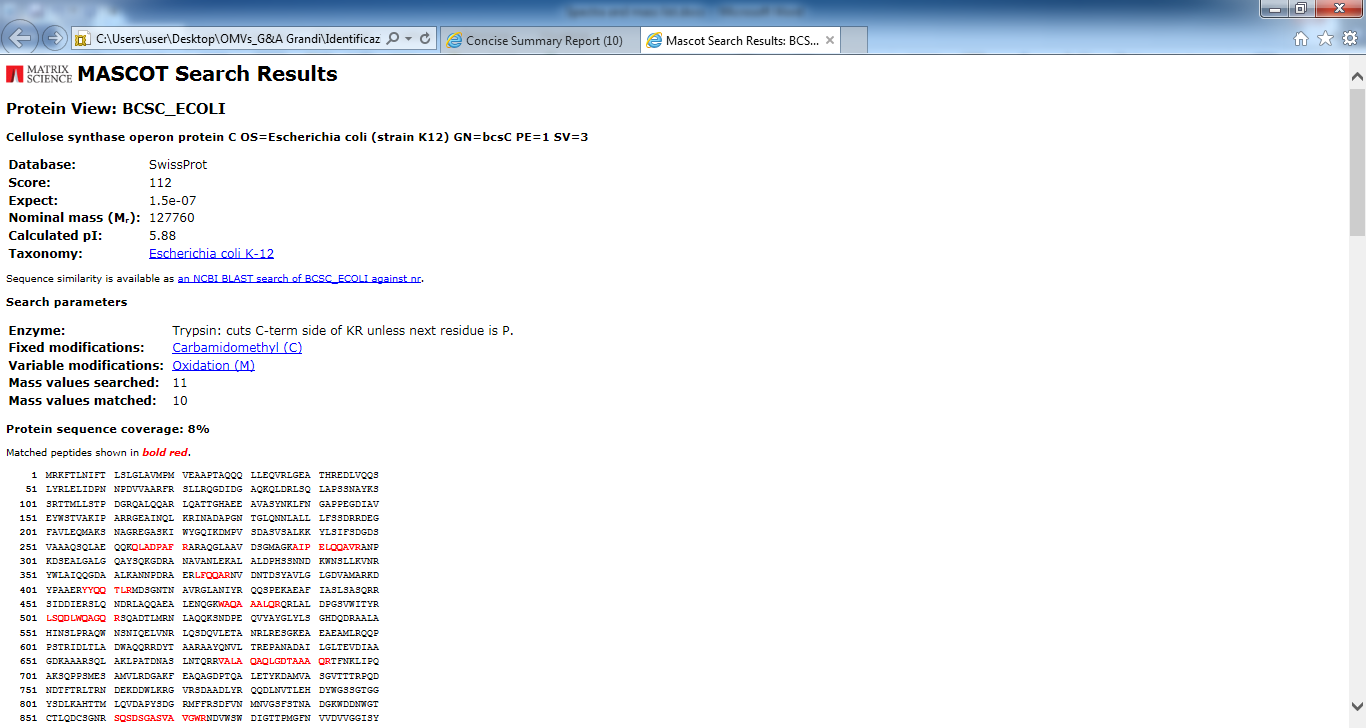

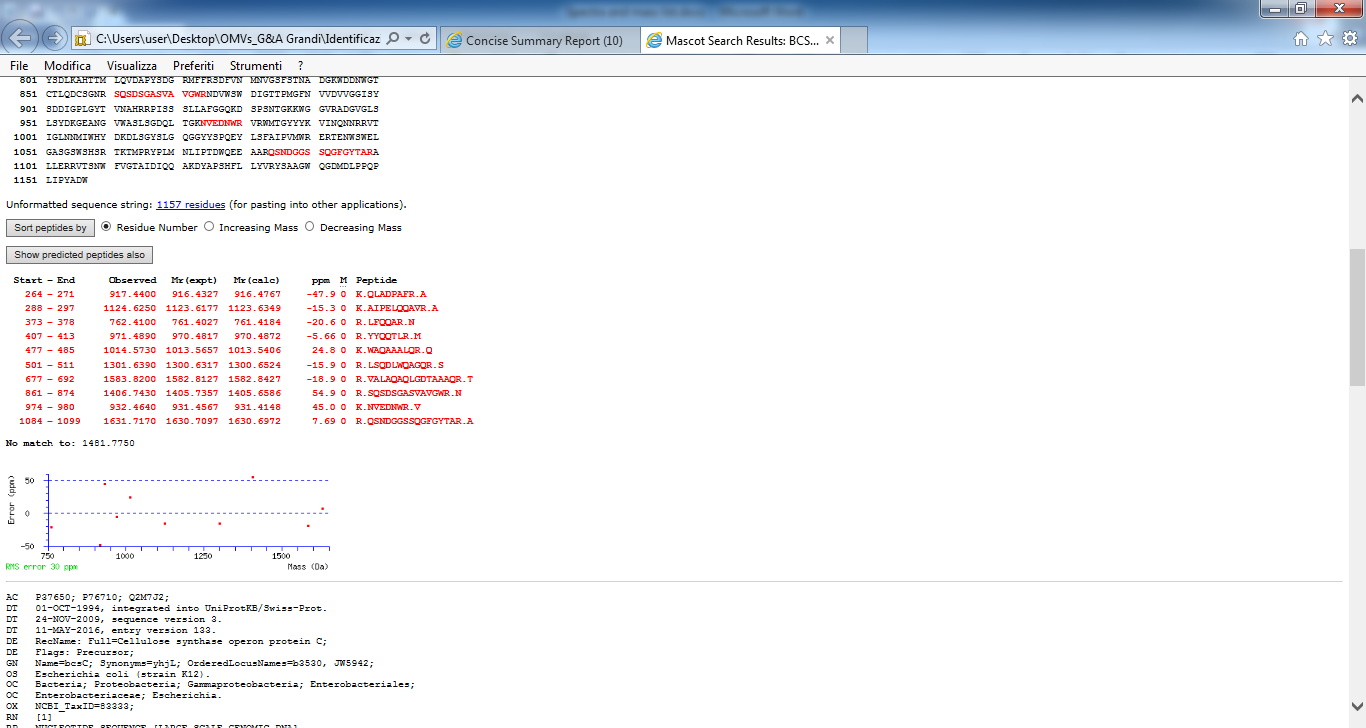


Spot N. 15 (PTRA_ECO57)

m/z

1062.591

1132.601

1179.669

1278.572

1292.676

1307.731

1348.882

1457.805

1524.833

1525.823

1622.941

1638.915

1827.866

1980.039

2001.937


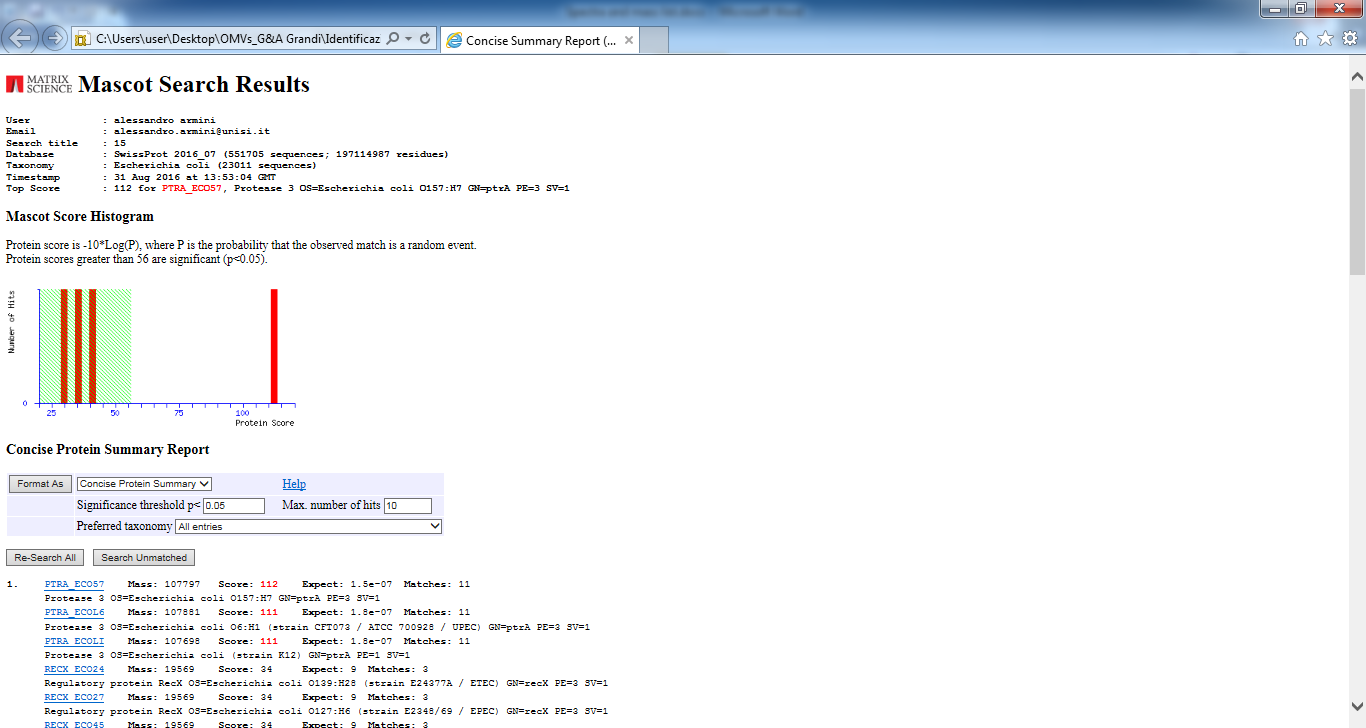


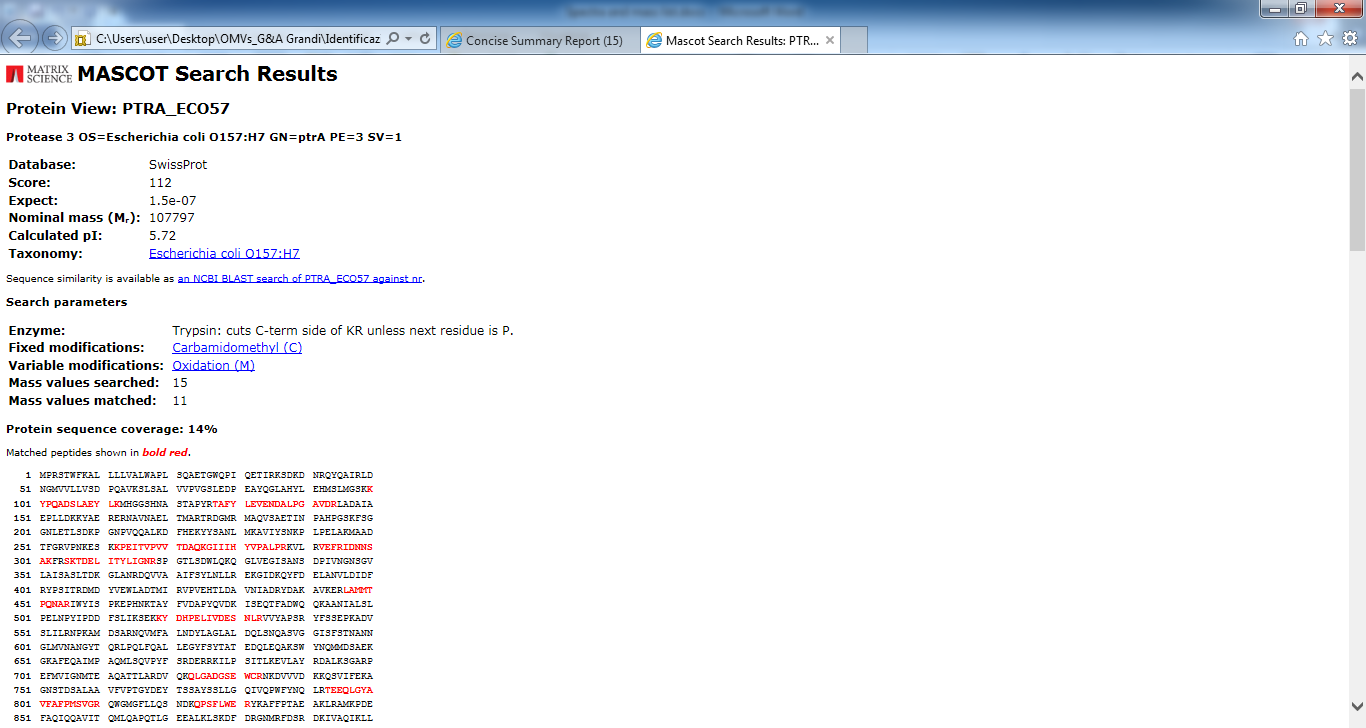

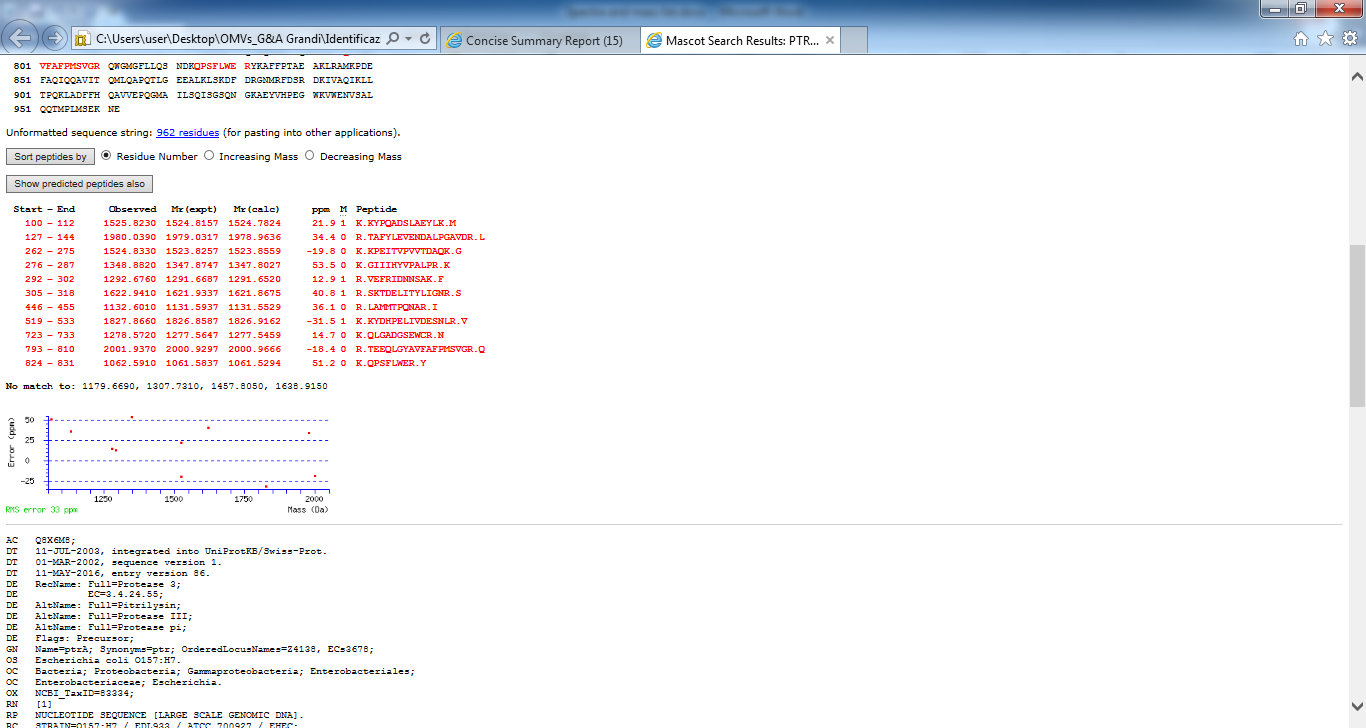


Spot N. 25 (LPTD_ECO57)

m/z

832.378

889.424

905.498

1064.600

1172.593

1193.600

1277.557

1295.567

1357.693

1404.704

1625.781

1669.730

1740.839

1791.705

1823.883

1851.908

2146.072

2286.136


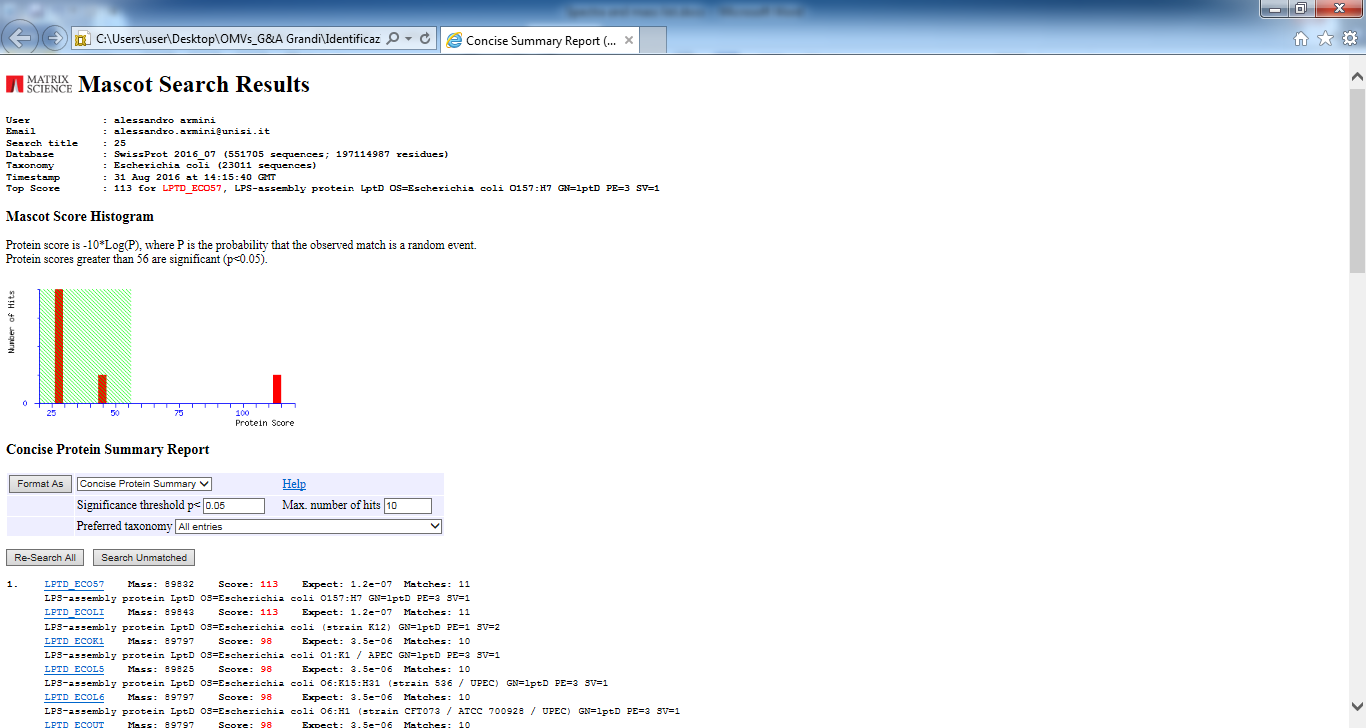


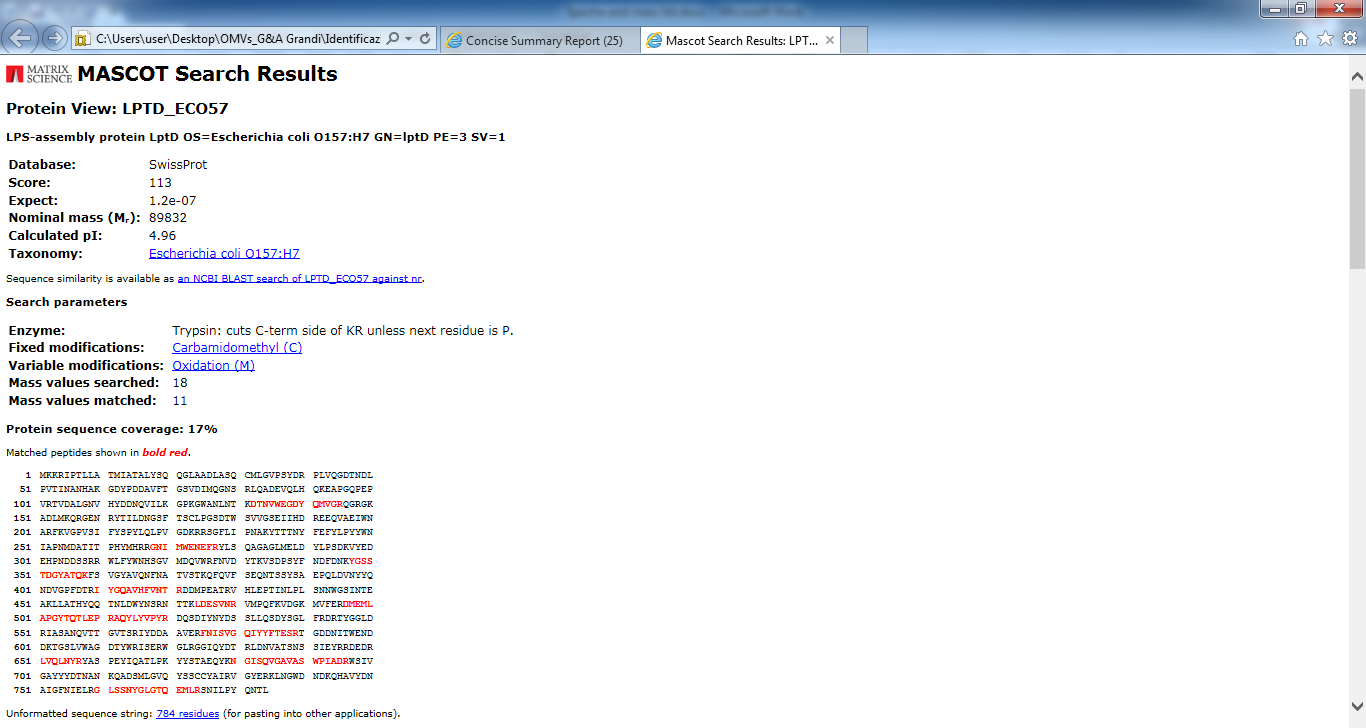

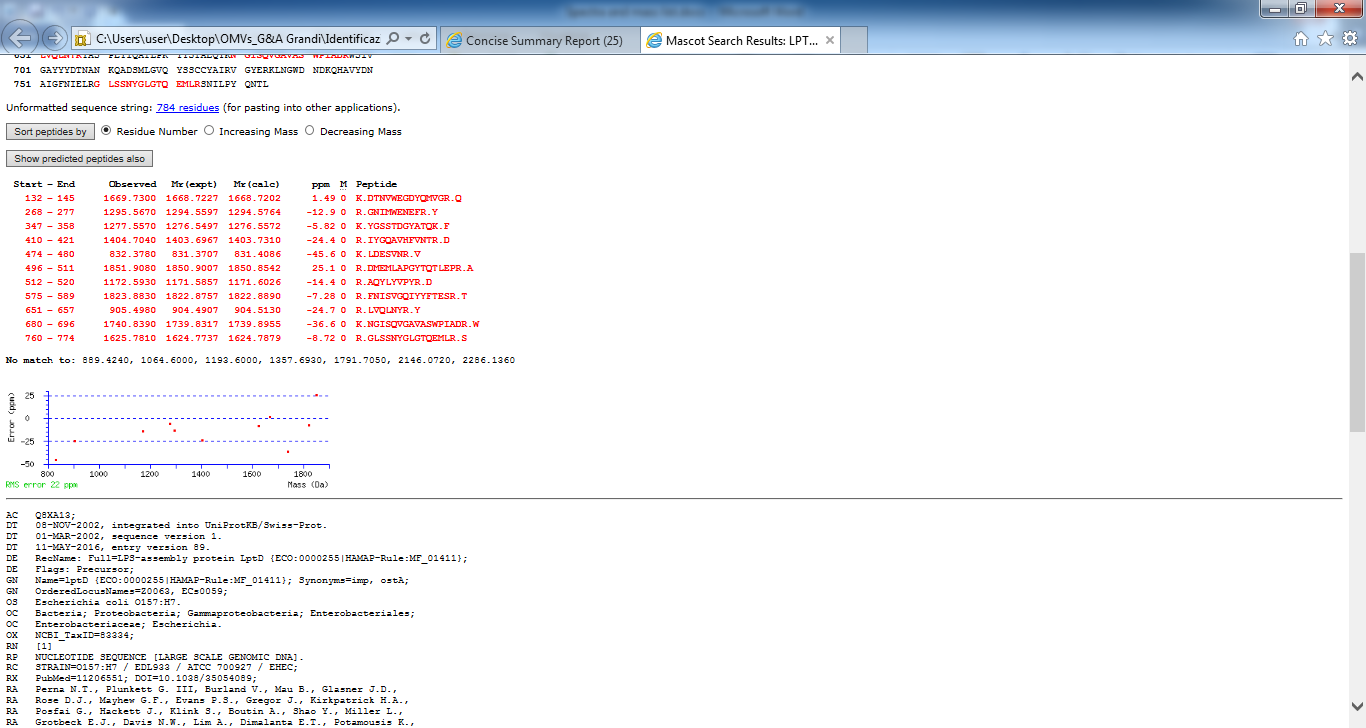


Spot N. 26 (LPTD_ECO57, BAMA_ECO24)

m/z

781.350

889.422

905.511

909.456

999.494

1051.515

1079.564

1114.594

1172.637

1179.629

1277.690

1283.766

1295.608

1307.664

1339.699

1344.668

1404.761

1451.709

1471.711

1520.821

1528.745

1568.761

1625.814

1638.837

1649.793

1657.804

1669.745

1740.894

1741.887

1791.719

1823.905

1844.914

1851.877

1859.928

1954.851

2018.919

2122.979

2136.923

2146.017

2154.969

2243.960

2320.162

2383.933

2547.175

2586.117

2705.098

2866.146

2912.374

3015.293


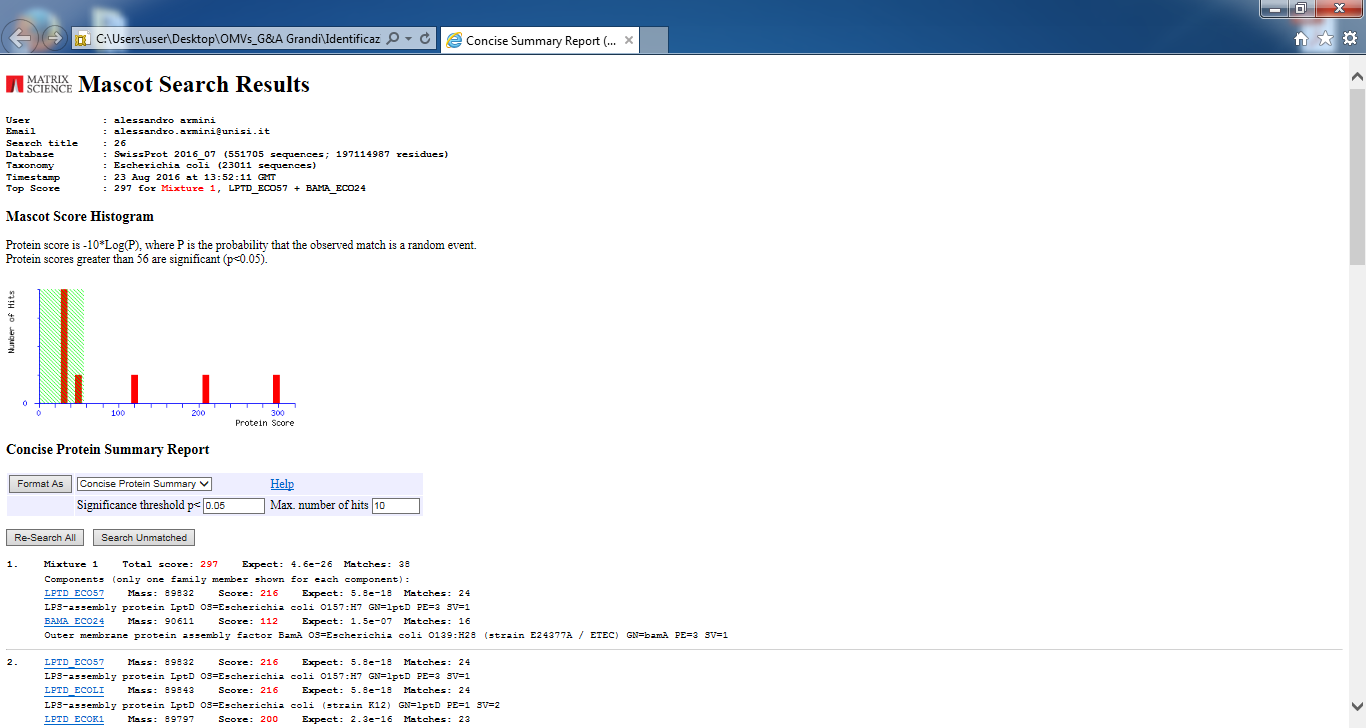


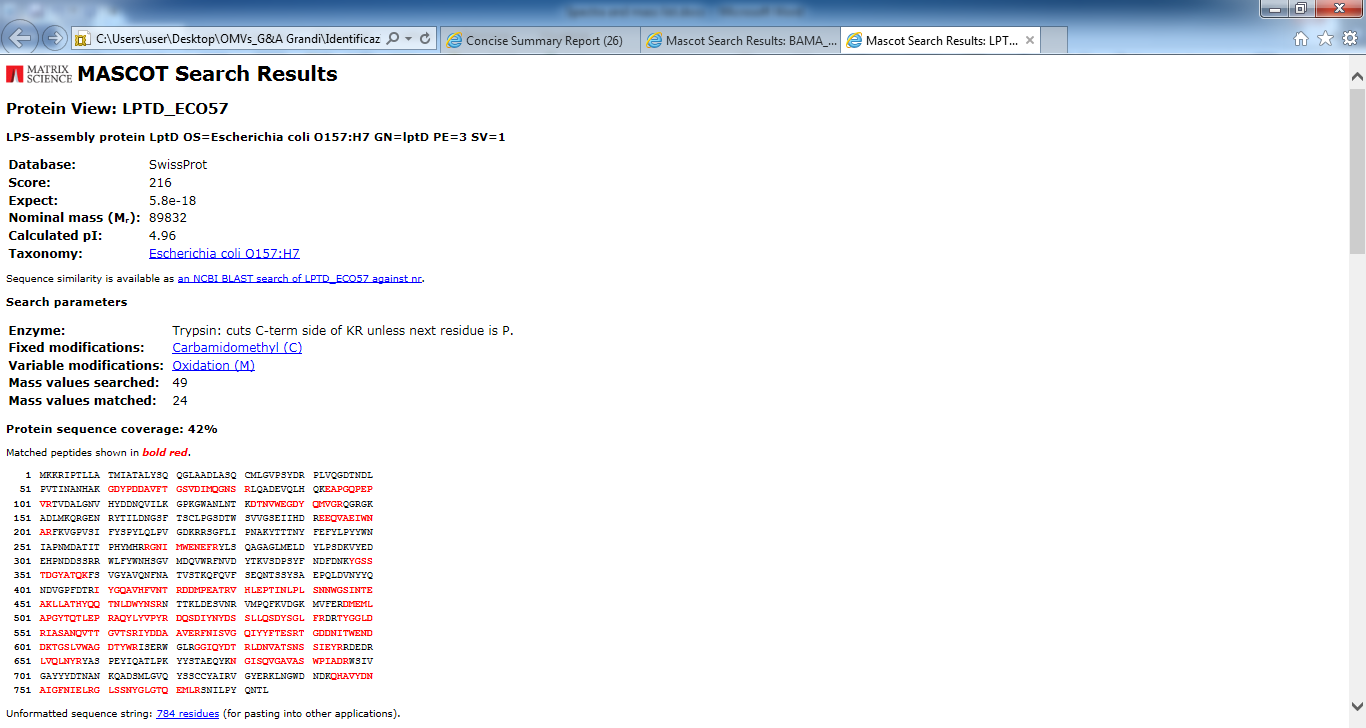


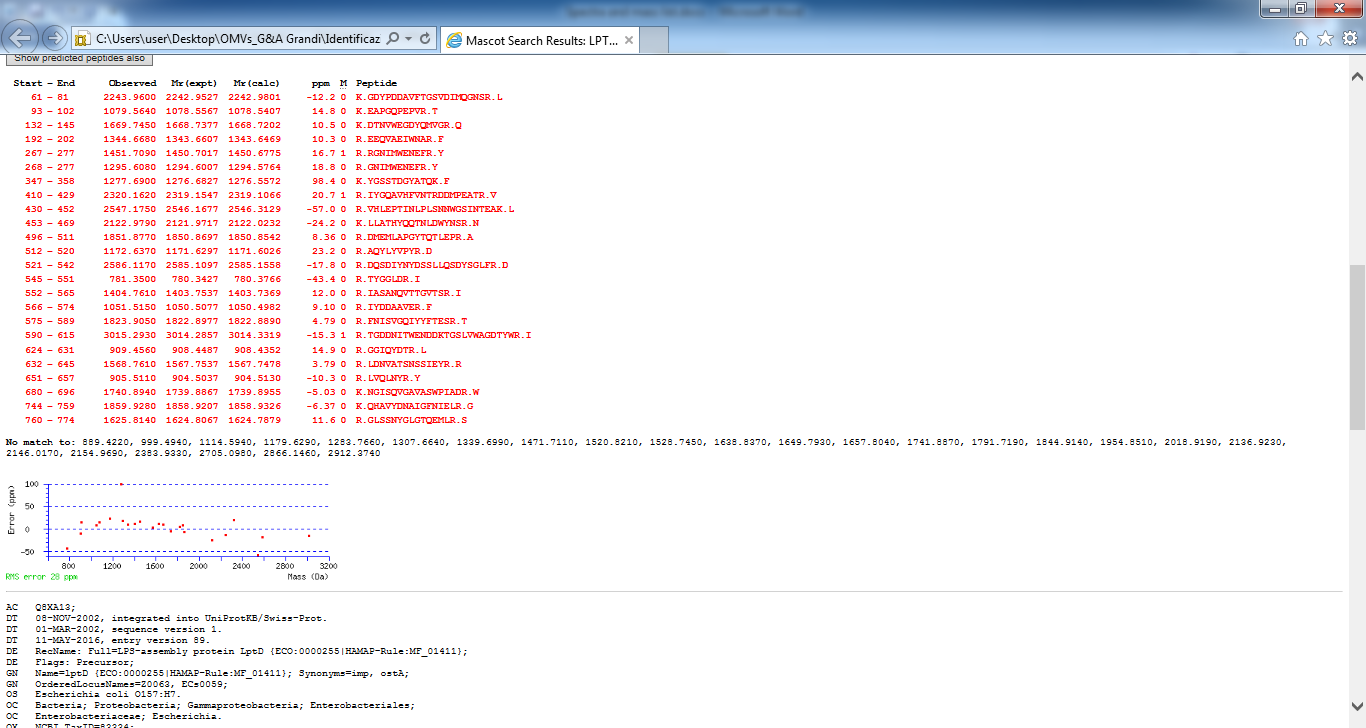


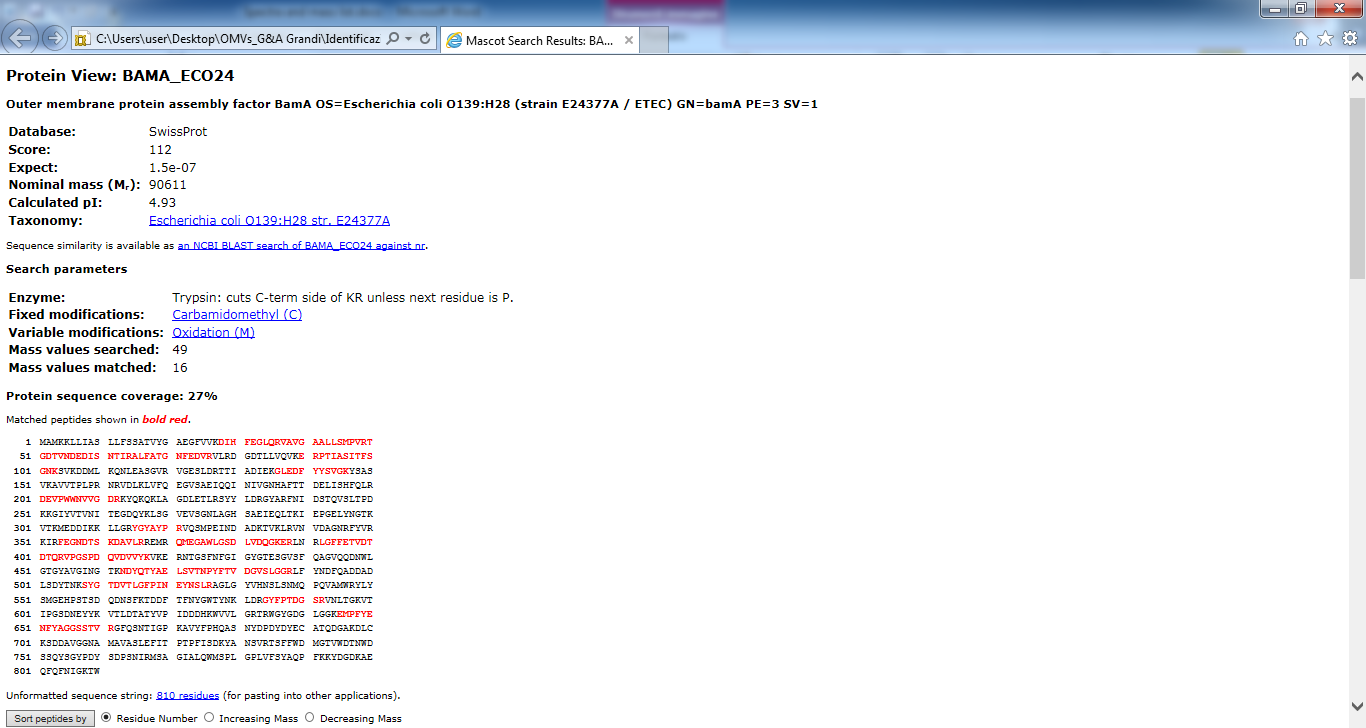


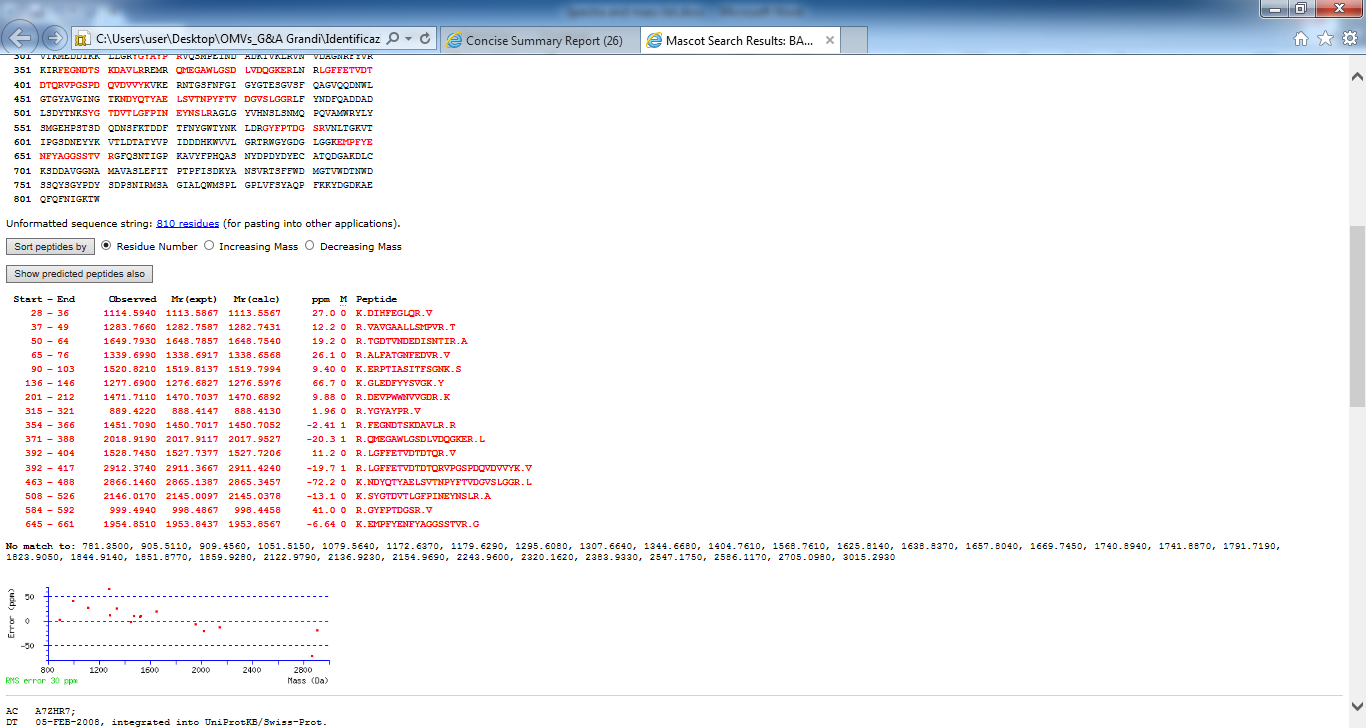


Spot N. 27 (BAMA_ECO24)

m/z

816.384

852.529

889.425

956.482

973.492

987.541

999.444

1009.429

1048.515

1114.556

1220.525

1221.574

1277.552

1283.696

1299.706

1339.638

1351.610

1385.604

1451.651

1455.796

1471.635

1502.682

1520.760

1528.689

1649.702

1692.787

1699.754

1720.827

1759.756

1844.879

1915.726

1954.812

1970.825

2001.893

2018.912

2146.049

2155.937

2158.014

2239.186

2255.001

2513.379

2545.366

2866.580

2912.715


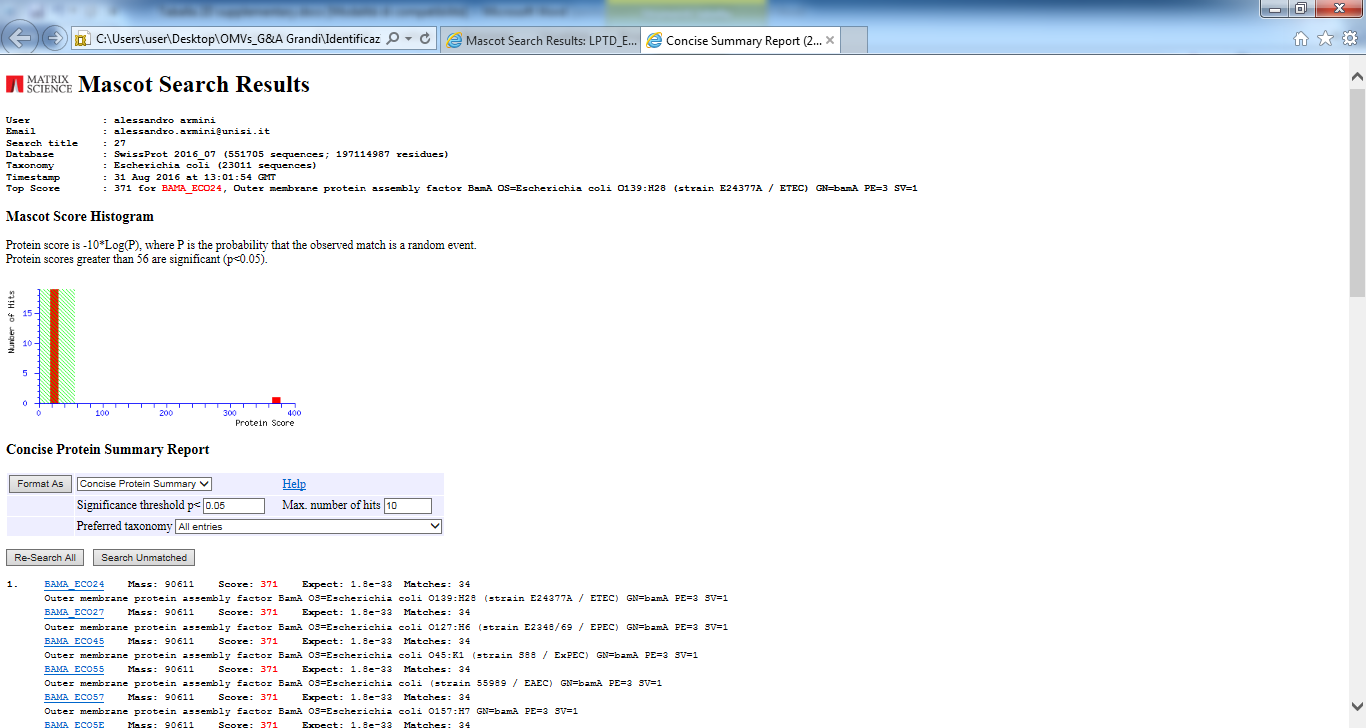


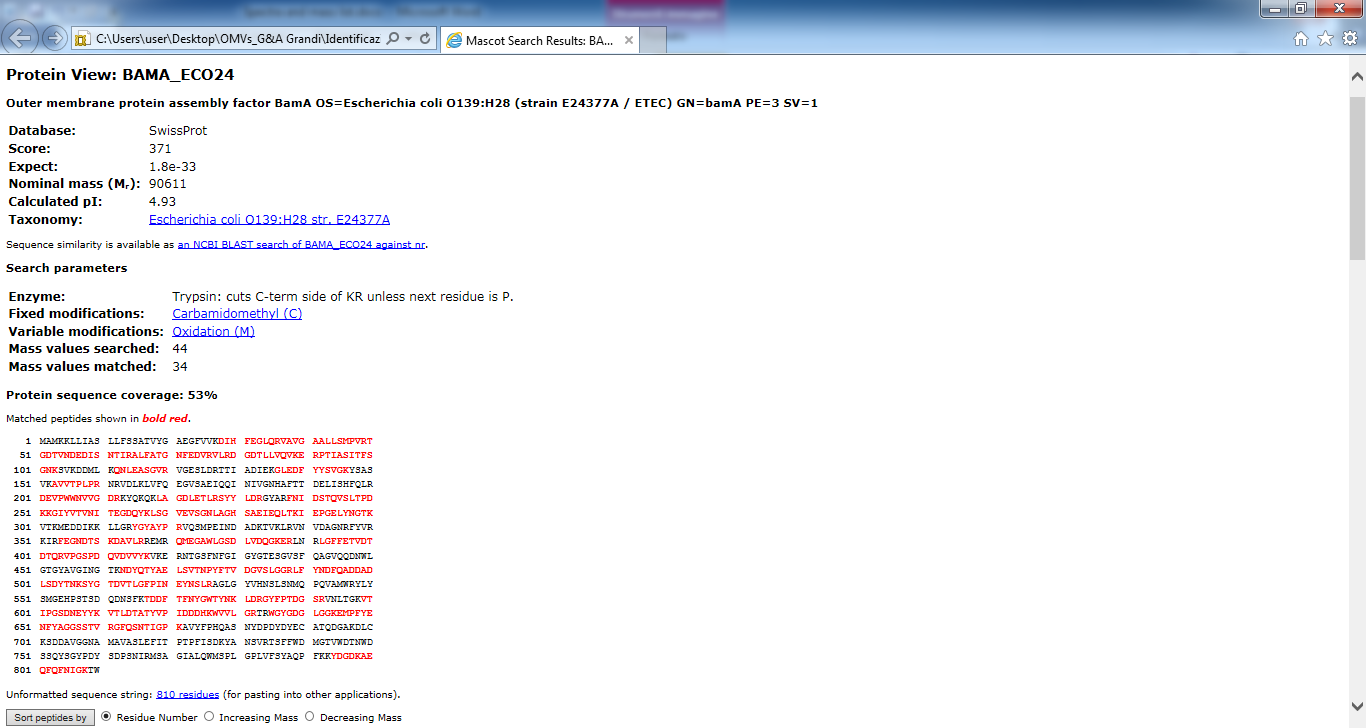


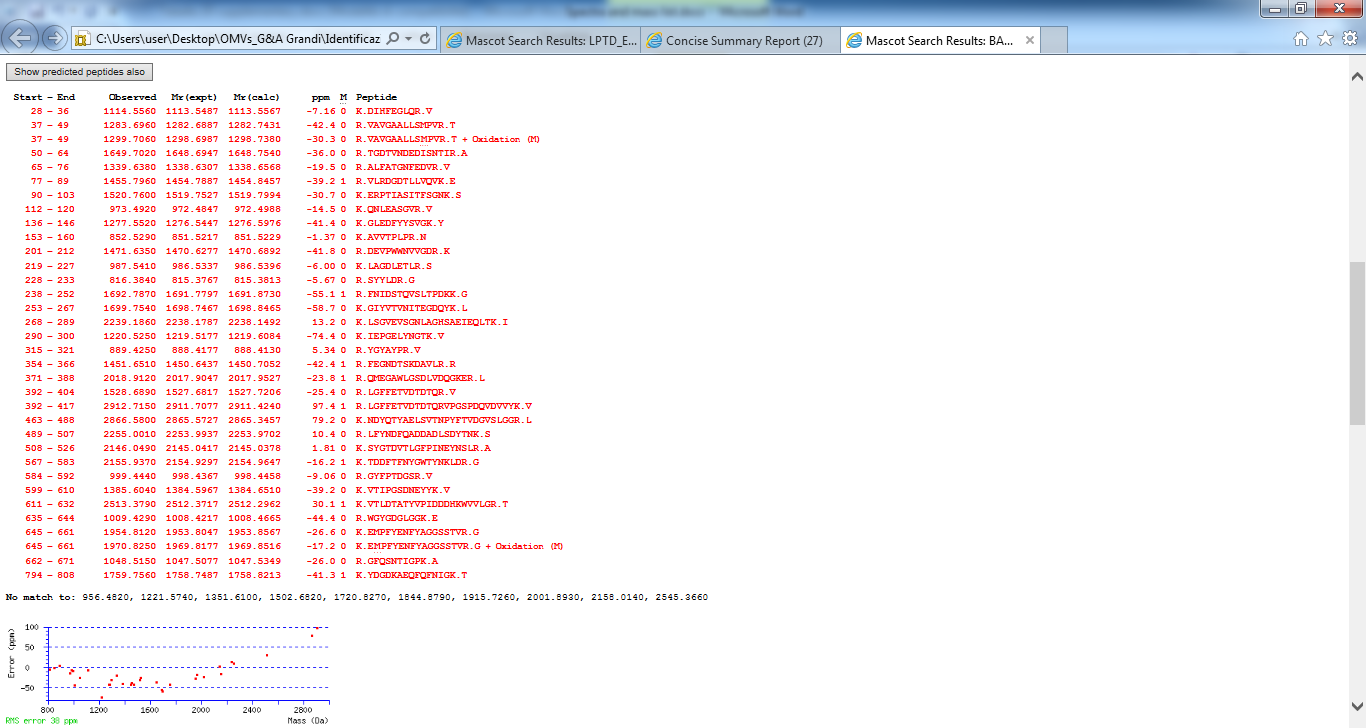


Spot N. 28 (LAMB_ECOBW)

m/z

731.415

859.484

917.525

949.464

964.533

1106.707

1145.599

1161.605

1294.712

1408.673

1436.717

1524.738

1563.798

1564.881

1661.790

1688.832

1731.950

1859.878

1892.003

2094.116

2133.153

2149.139

2297.274

2317.115

2329.262

2356.204

2368.215

2372.224

2465.123

2954.487

3042.333

3217.227


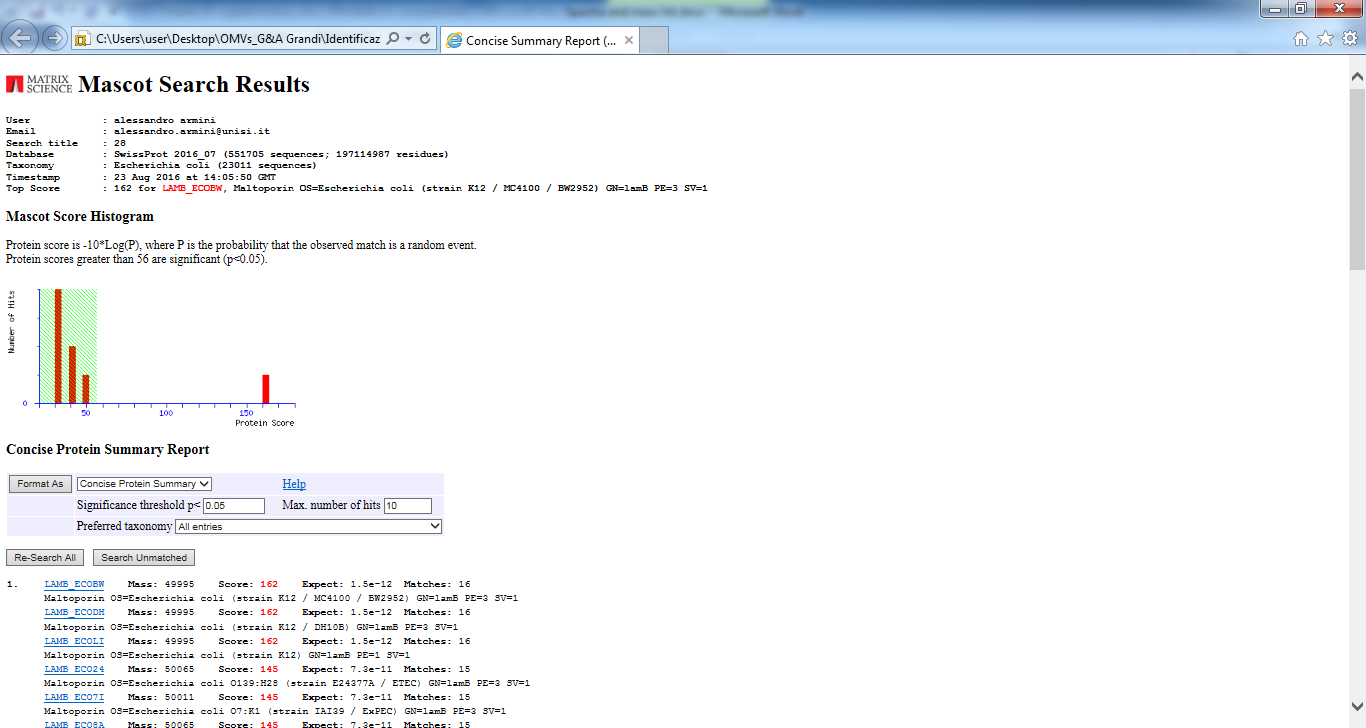

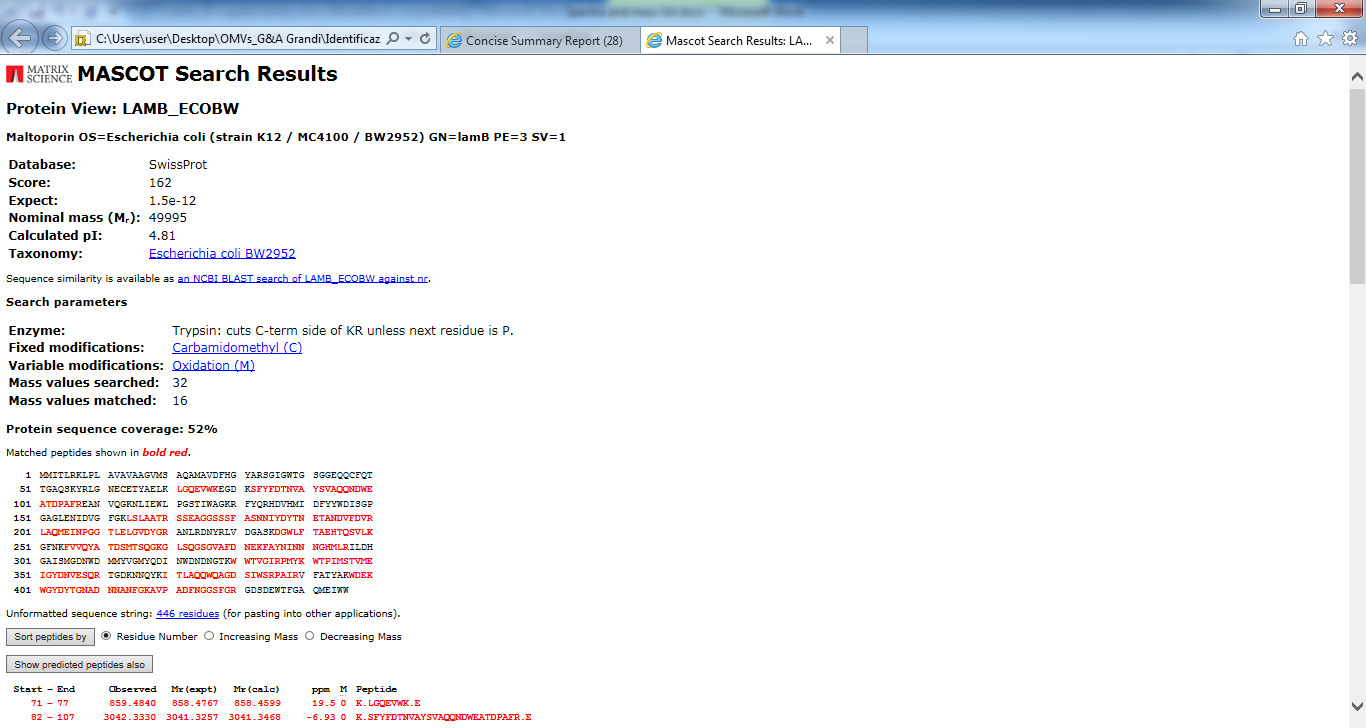

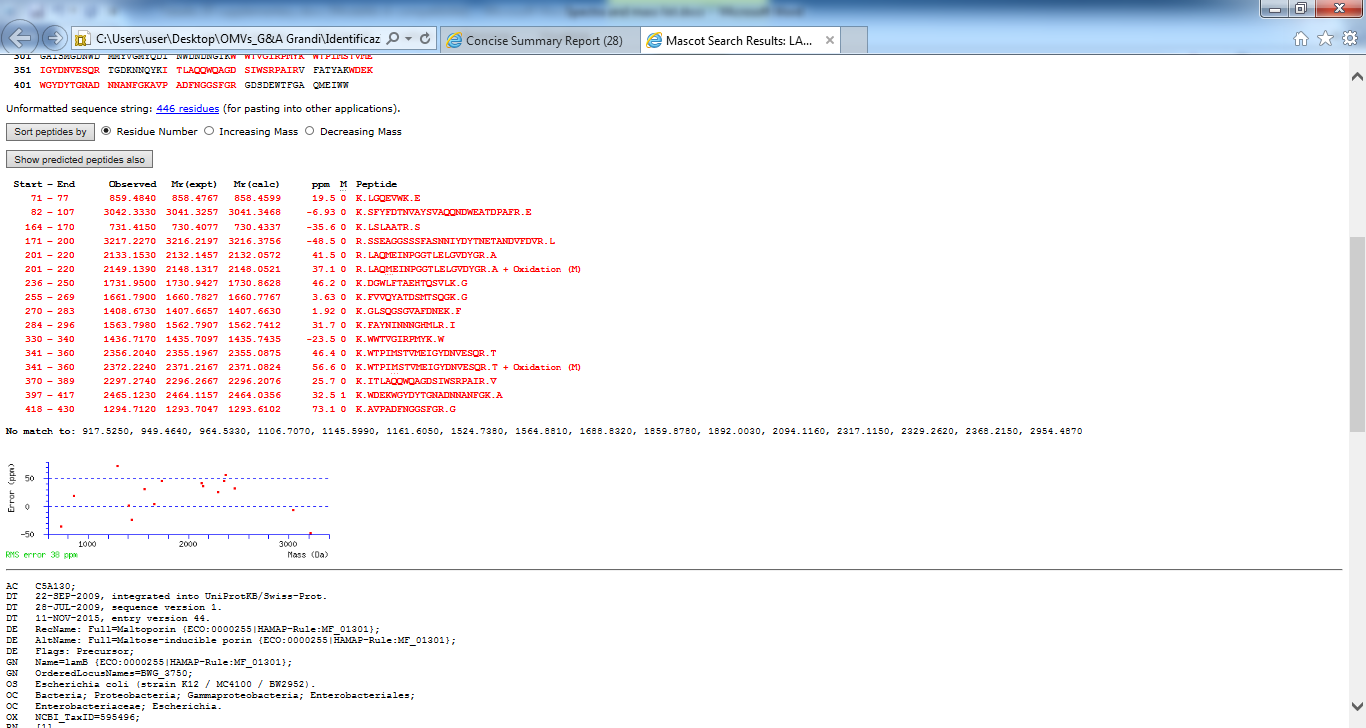


Spot N. 32 (FEPA_ECOLI)

m/z

888.460

1152.625

1178.558

1619.746

1634.769

1787.781

1797.775

1983.025

2105.048


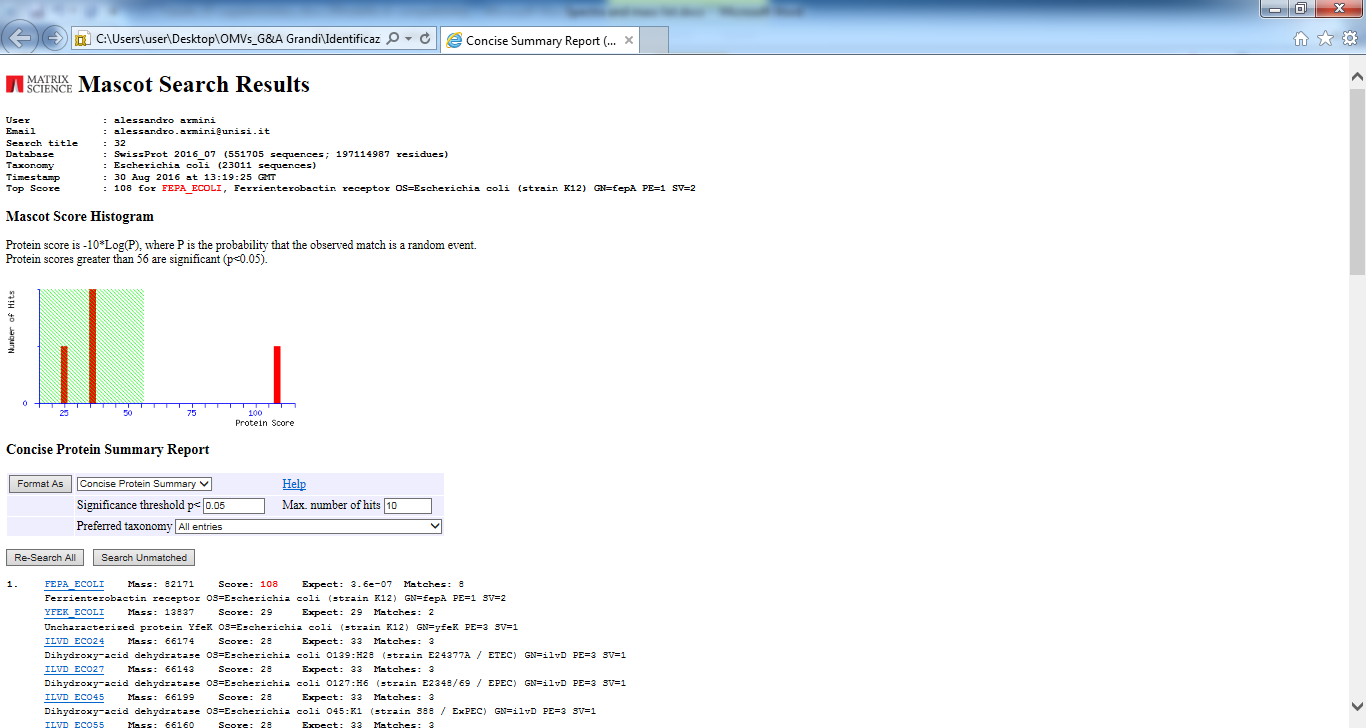


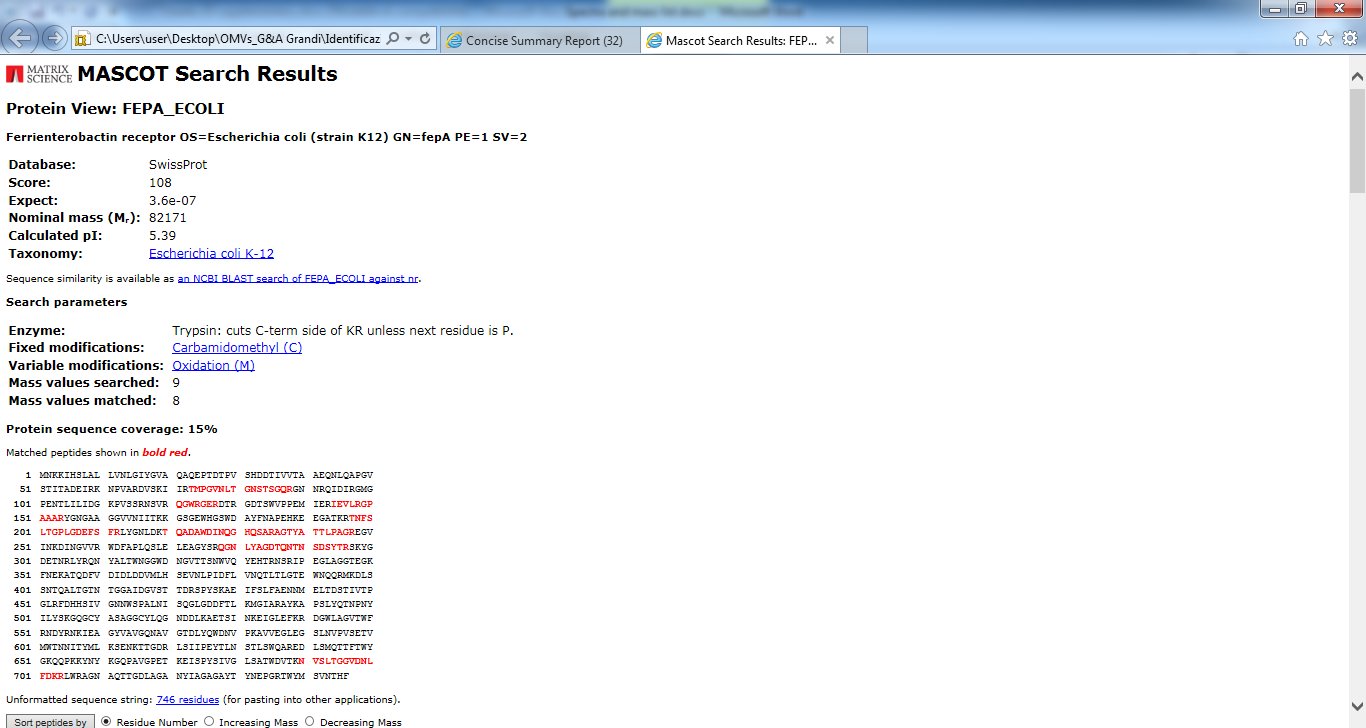

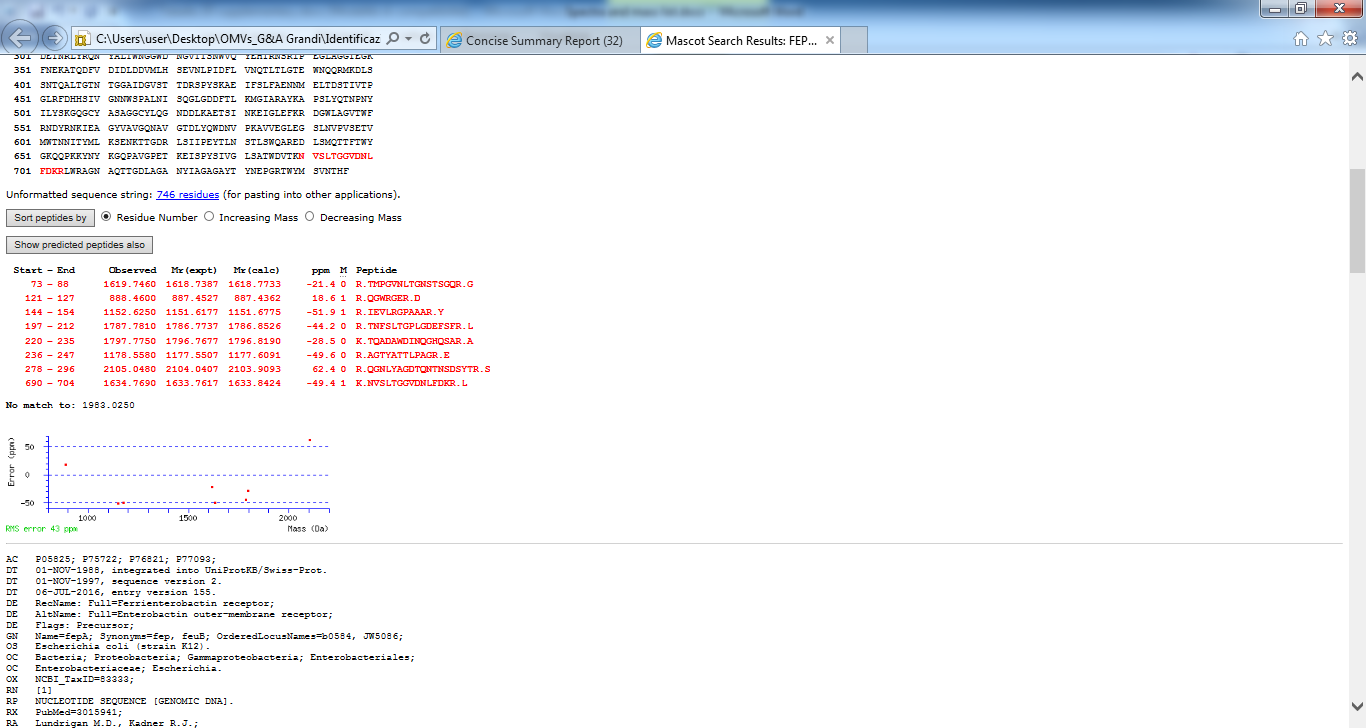


Spot N. 33(FEPA_ECOLI)

m/z

831.485

871.414

888.457

991.550

1069.481

1152.662

1178.591

1286.548

1307.622

1339.601

1371.590

1412.731

1413.722

1516.658

1532.671

1619.726

1634.787

1646.786

1787.808

1797.790

1799.812

1871.889

1888.818

1944.994

1981.951

1984.010

2000.001

2003.981

2013.916

2104.898

2116.901

2601.347

2633.347

2768.532

2844.536


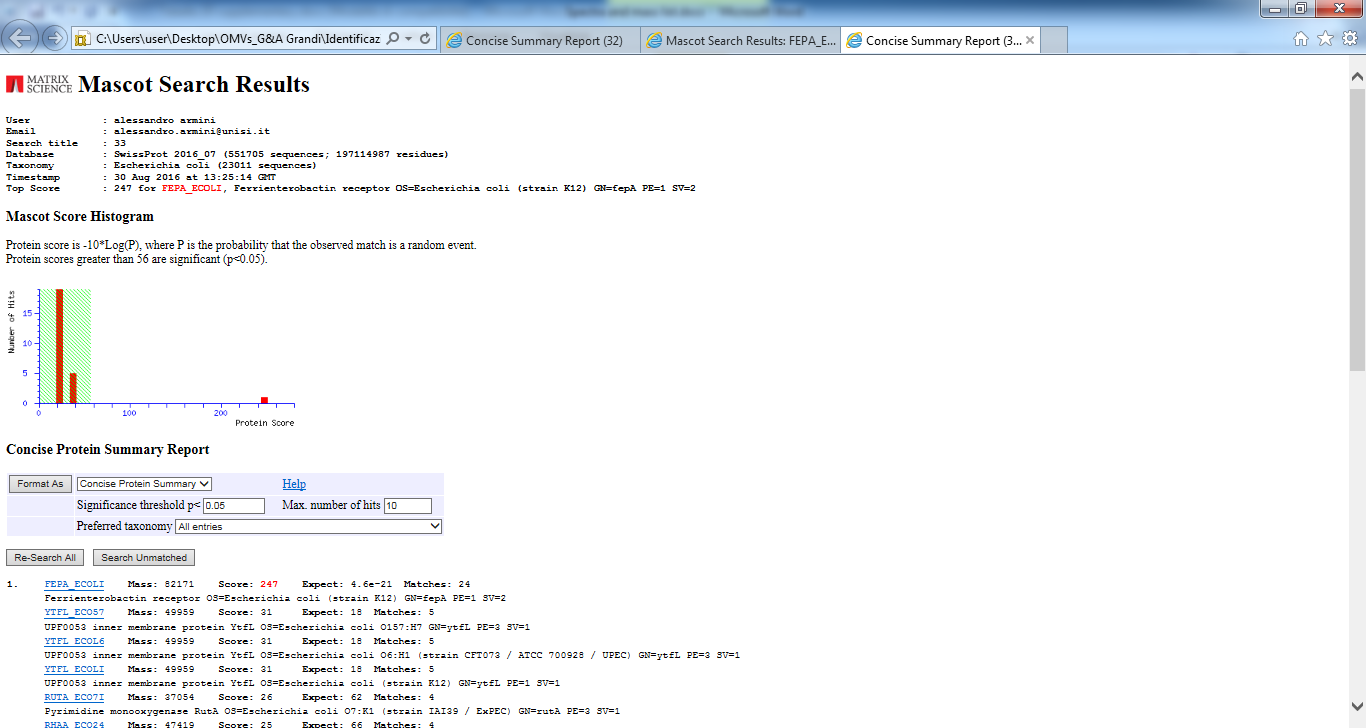

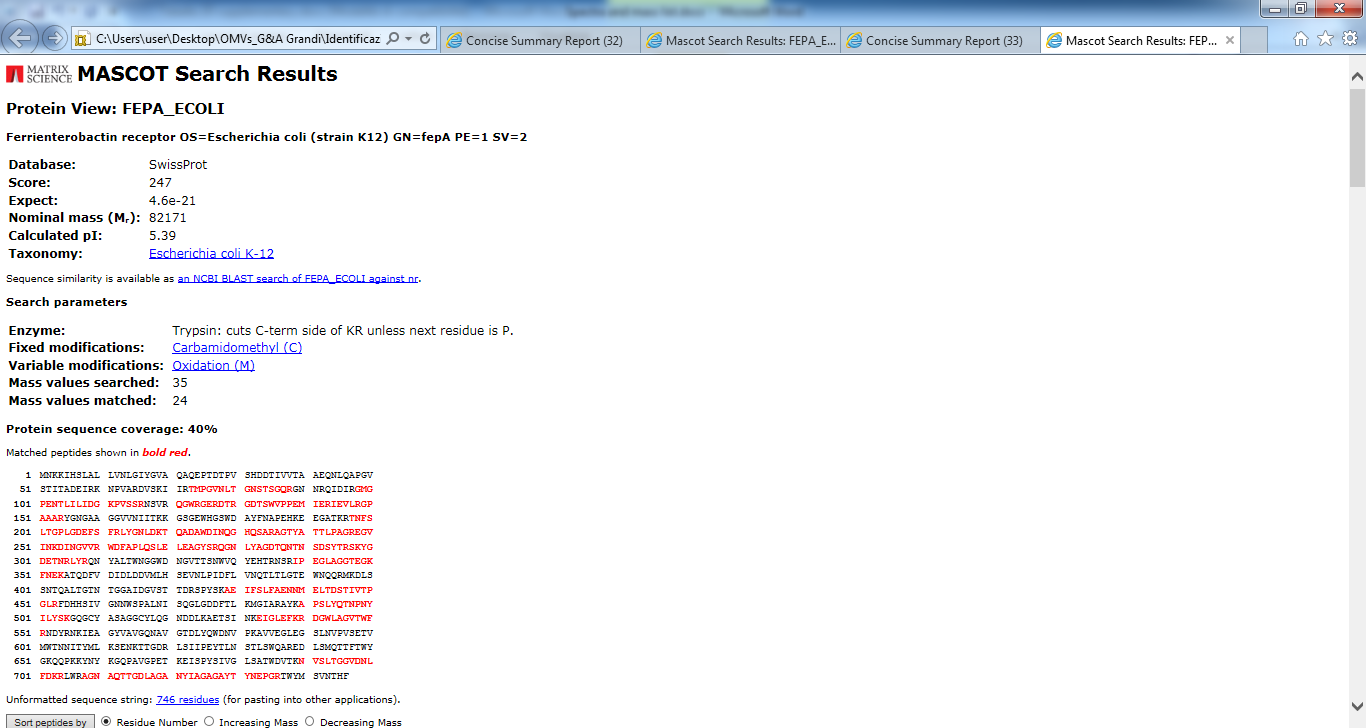

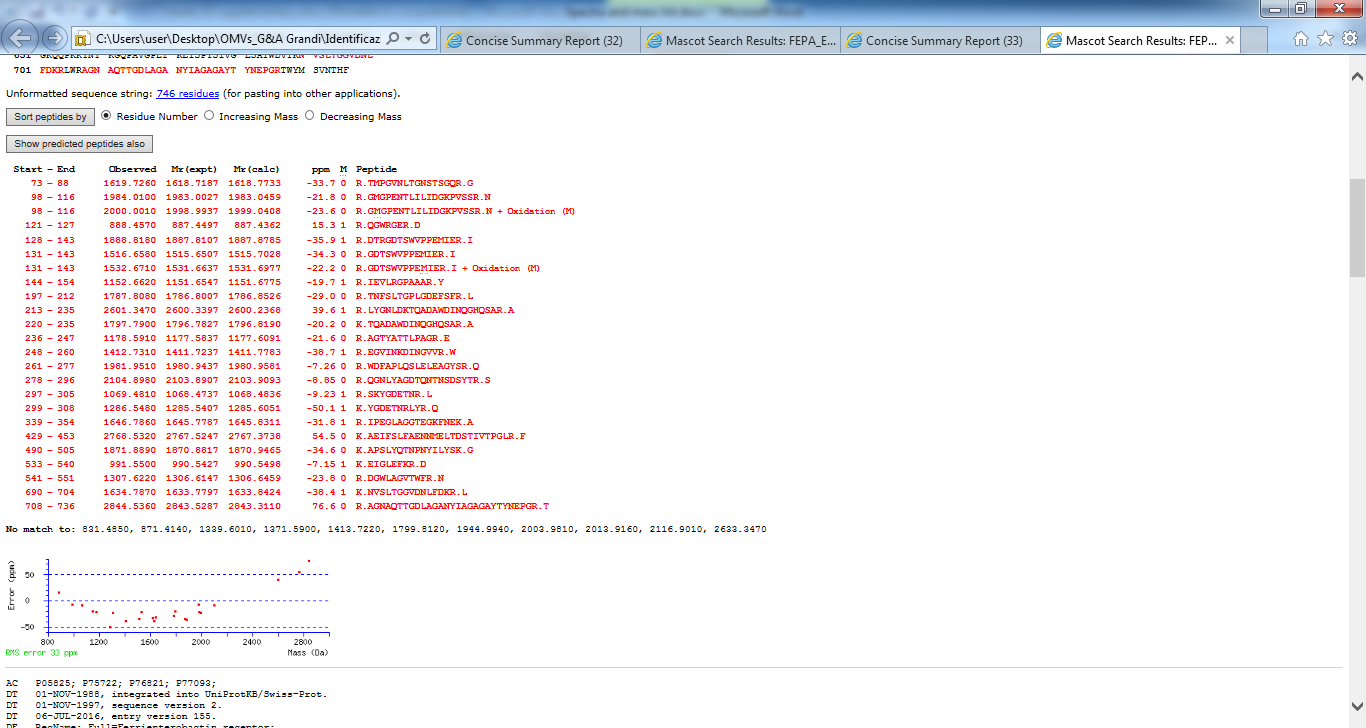


Spot N. 34(FEPA_ECOLI)

m/z

831.467

871.432

888.449

991.547

1069.475

1178.583

1285.509

1307.604

1323.609

1339.594

1355.592

1371.581

1412.720

1477.707

1516.636

1532.643

1580.760

1619.692

1634.767

1646.764

1787.768

1797.749

1799.787

1871.848

1888.796

1944.918

1965.899

1981.889

1983.978

1997.919

1999.975

2003.935

2013.892

2090.204

2092.039

2104.845

2552.169

2601.220

2729.226

2768.402

2784.411

2811.387

2844.381

2946.436

2978.447

3074.569

3082.658

3114.690

3198.654

3230.672

3261.697

3373.941


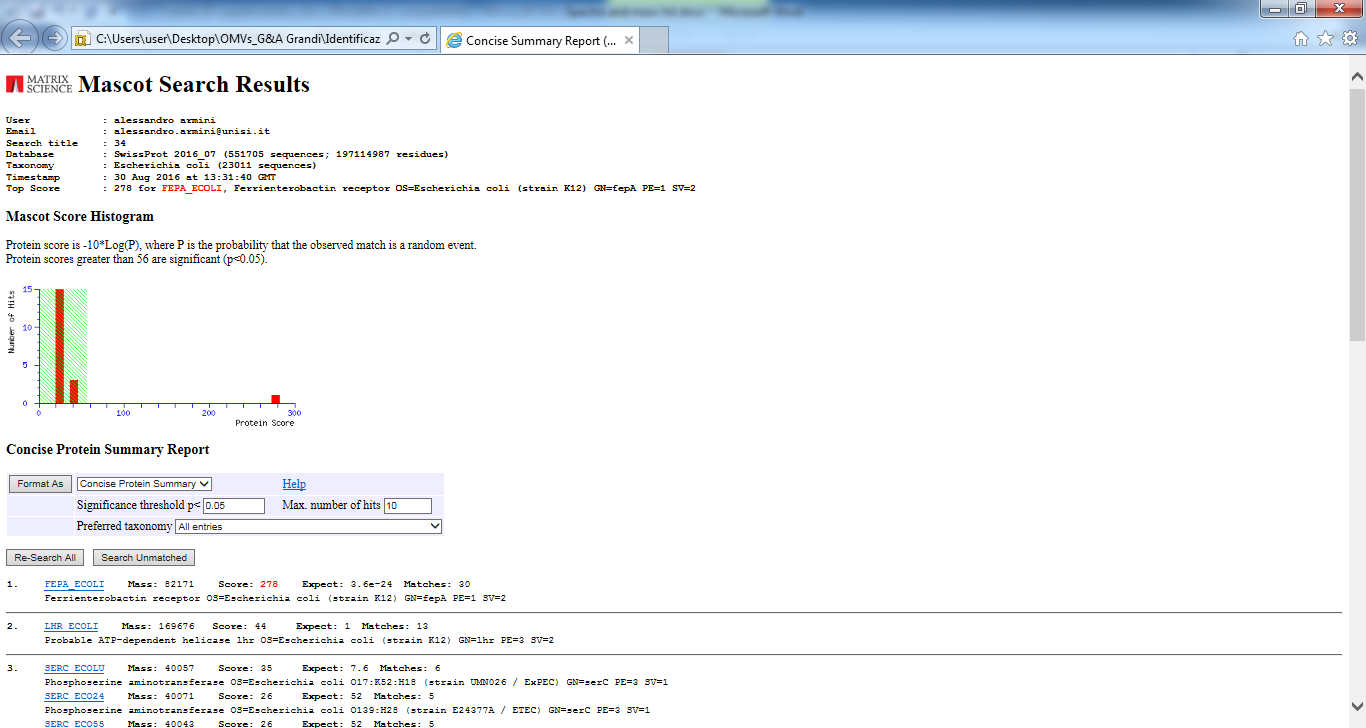

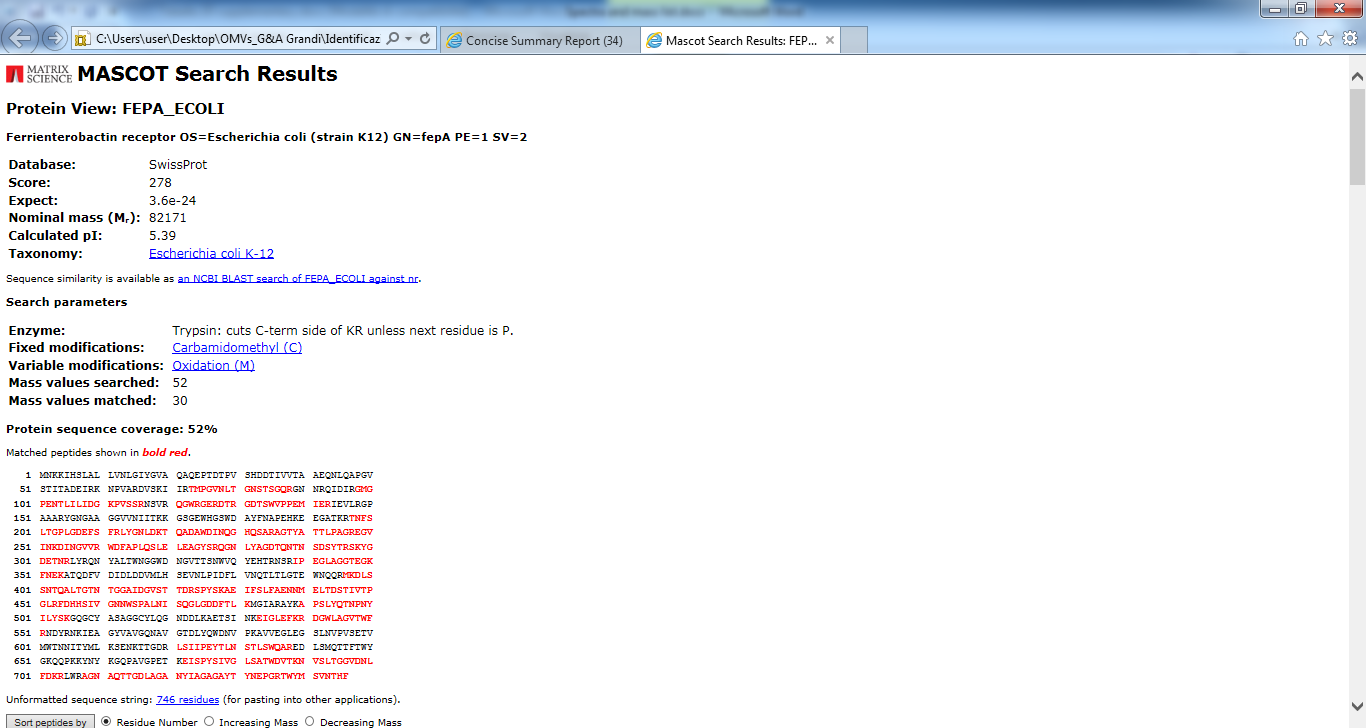

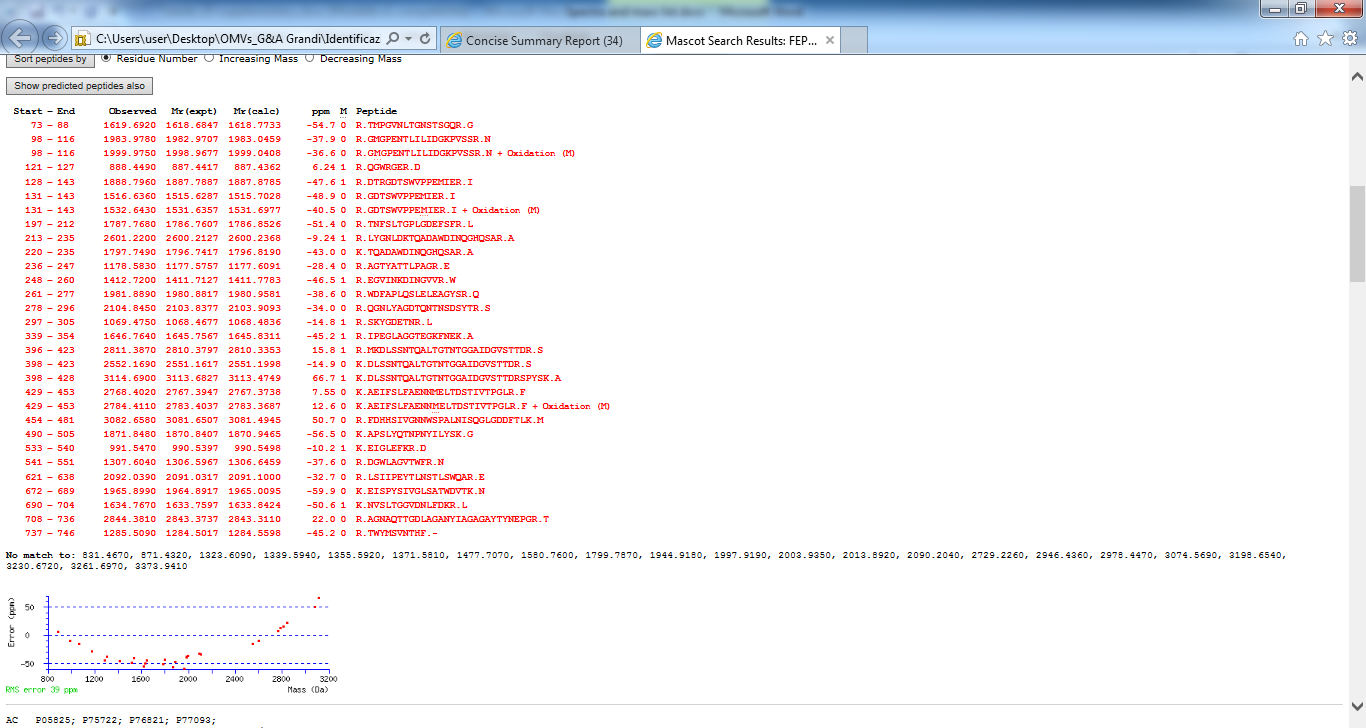


Spot N. 37 (FHUA_ECOLI)

m/z

1139.565

1247.731

1278.773

1359.756

1396.726

1436.781

1650.969

2134.167


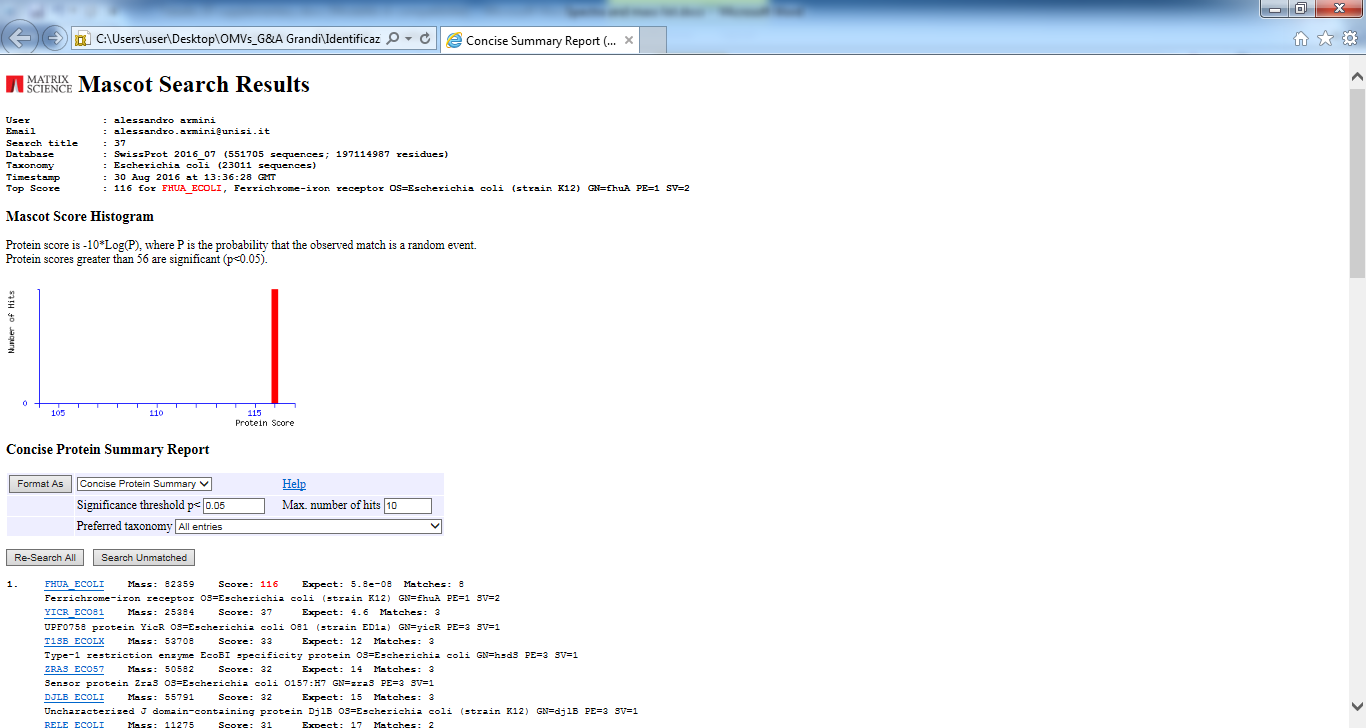

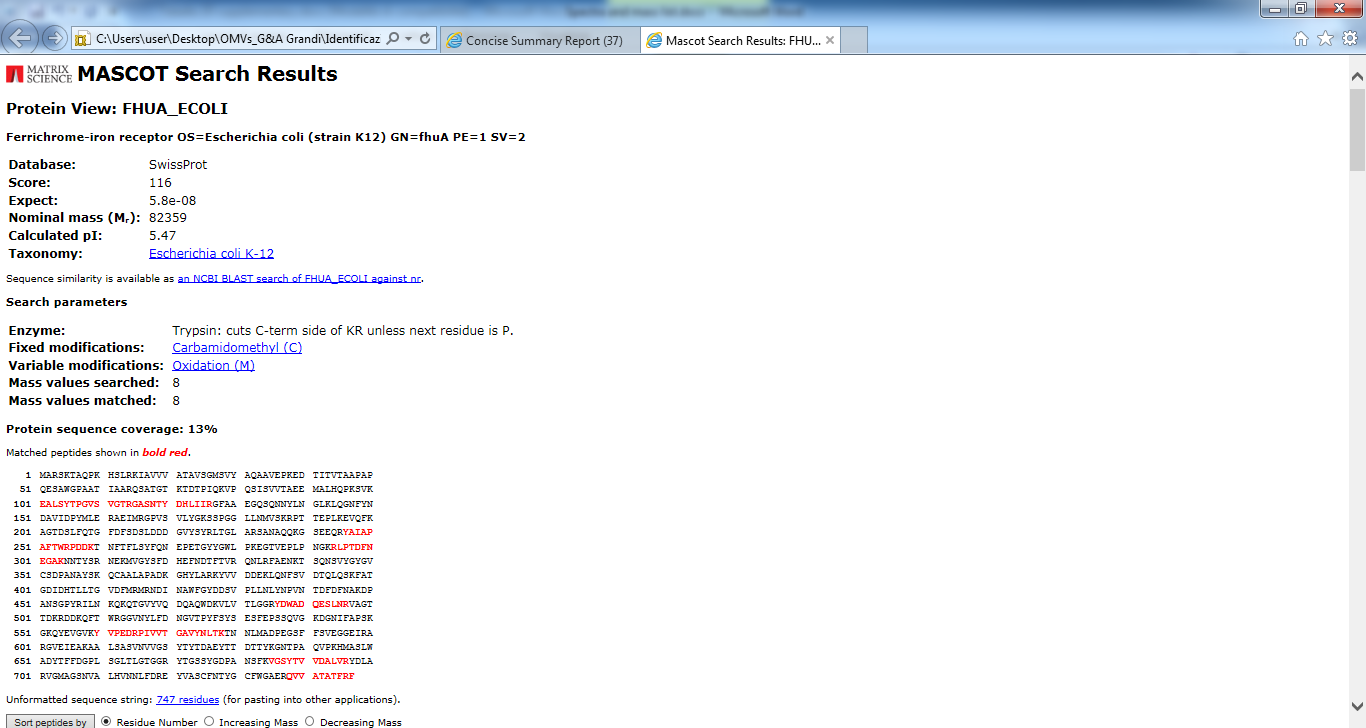

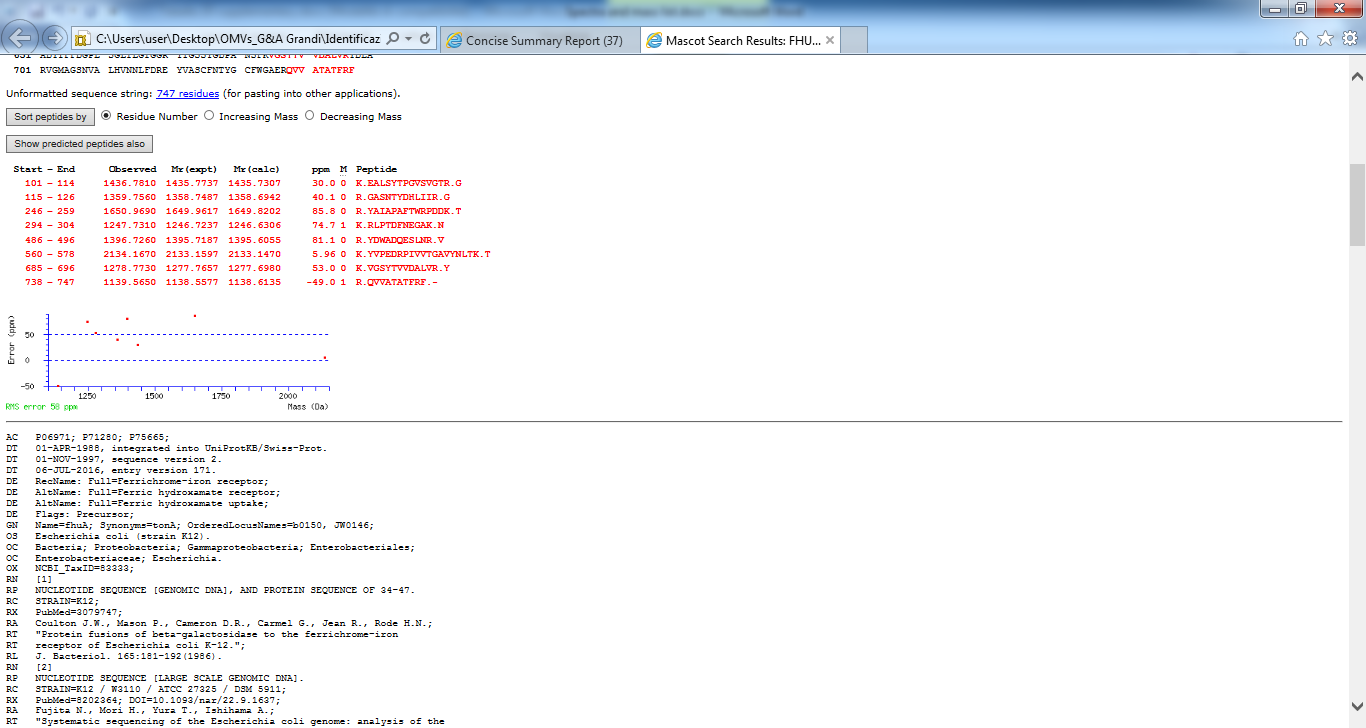


Spot N. 38 (FHUA_ECOLI)

m/z

976.439

1095.505

1139.598

1173.532

1247.607

1278.676

1359.663

1396.576

1436.703

1448.708

1493.611

1526.709

1572.828

1650.787

1682.783

1750.887

1755.845

1811.804

1955.010

1969.939

2008.945

2025.921

2064.902

2080.901

2134.128

2230.982

2270.033

2286.050

2354.169

2395.951

2595.360

2862.388

3119.862

3151.861


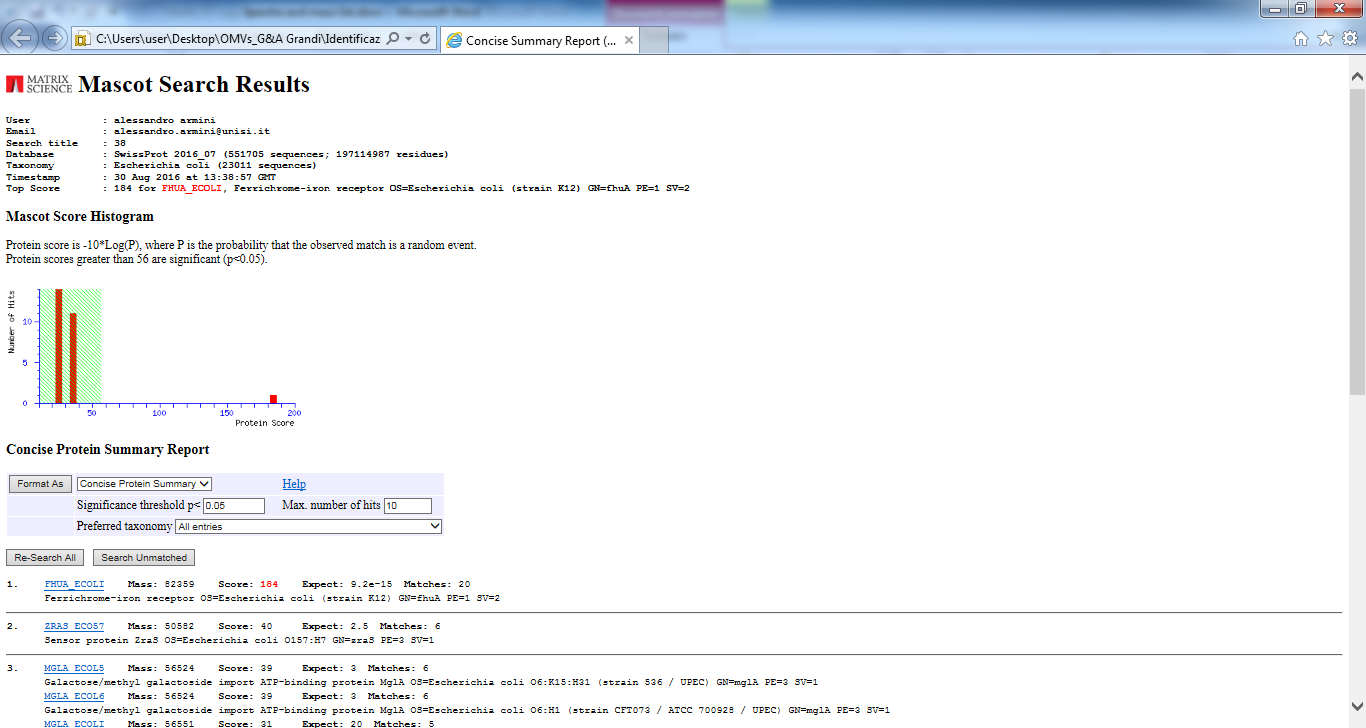

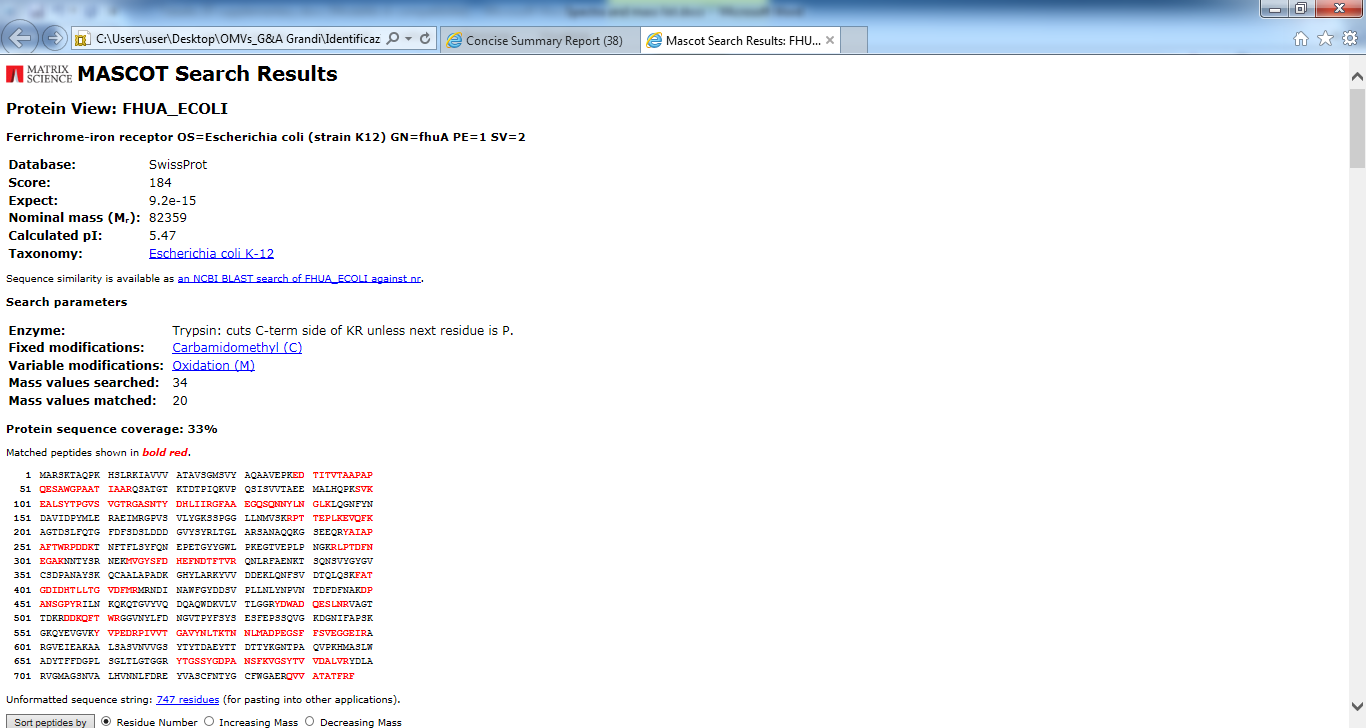

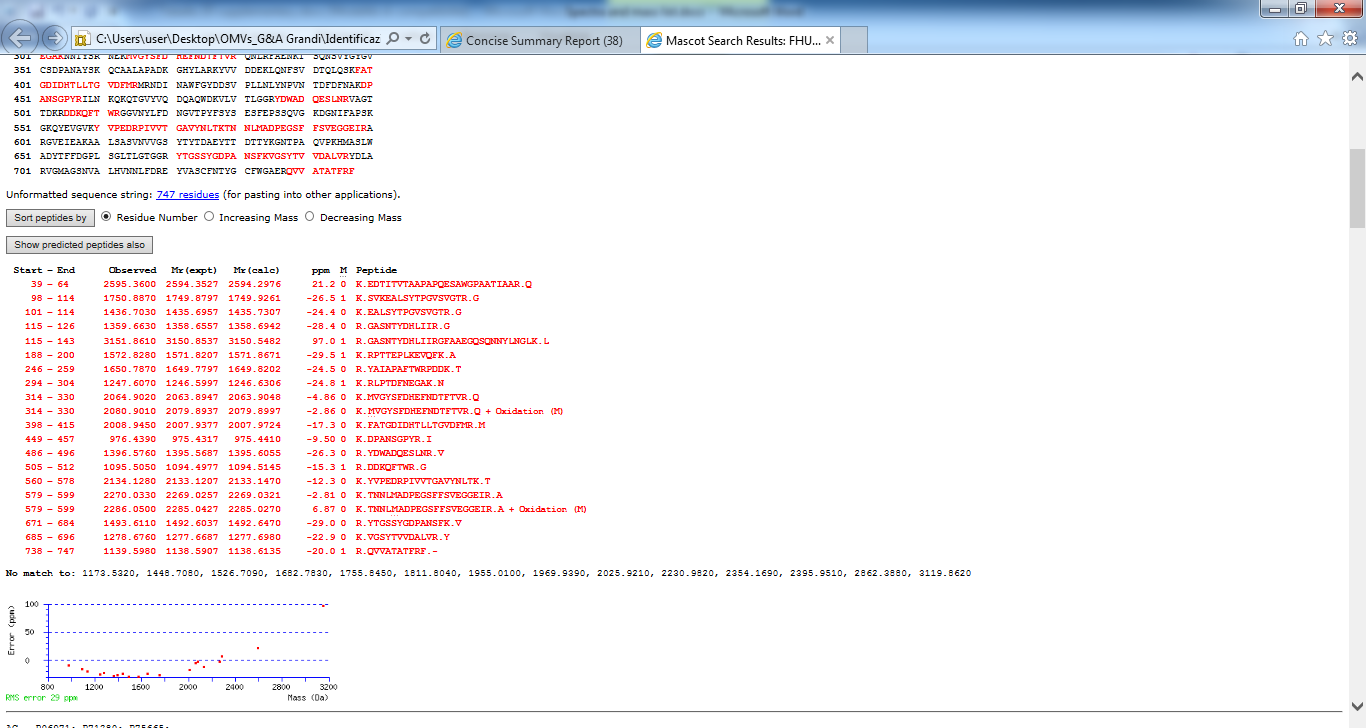


Spot N. 39 (YNCD_ECOLI, FHUA_ECOLI, FEPA_ECOLI)

m/z

814.524

881.449

888.468

968.458

975.481

976.474

987.482

992.572

1095.536

1139.620

1173.577

1178.610

1247.644

1278.709

1307.648

1312.602

1329.674

1339.638

1359.710

1371.660

1396.626

1404.562

1408.614

1431.711

1436.560

1448.760

1460.755

1516.716

1526.756

1538.709

1556.701

1572.759

1582.758

1594.669

1611.791

1614.744

1619.760

1634.835

1650.835

1665.777

1676.791

1682.818

1715.805

1731.781

1741.873

1747.793

1755.881

1787.856

1797.824

1799.865

1811.855

1874.942

1913.943

1929.952

1955.056

1966.928

1969.960

1981.971

1984.058

2008.981

2013.925

2025.958

2064.951

2080.948

2091.009

2104.929

2130.035

2134.010

2142.004

2146.047

2170.016

2231.001

2244.968

2270.091

2282.088

2286.097

2354.224

2391.143

2407.157

2428.220

2484.342

2556.341

2595.435

2601.345

2627.408

2768.510

2844.507

2862.471

2880.650

2898.568

2937.731

2953.721

3120.137

3405.688

3416.697

3444.599


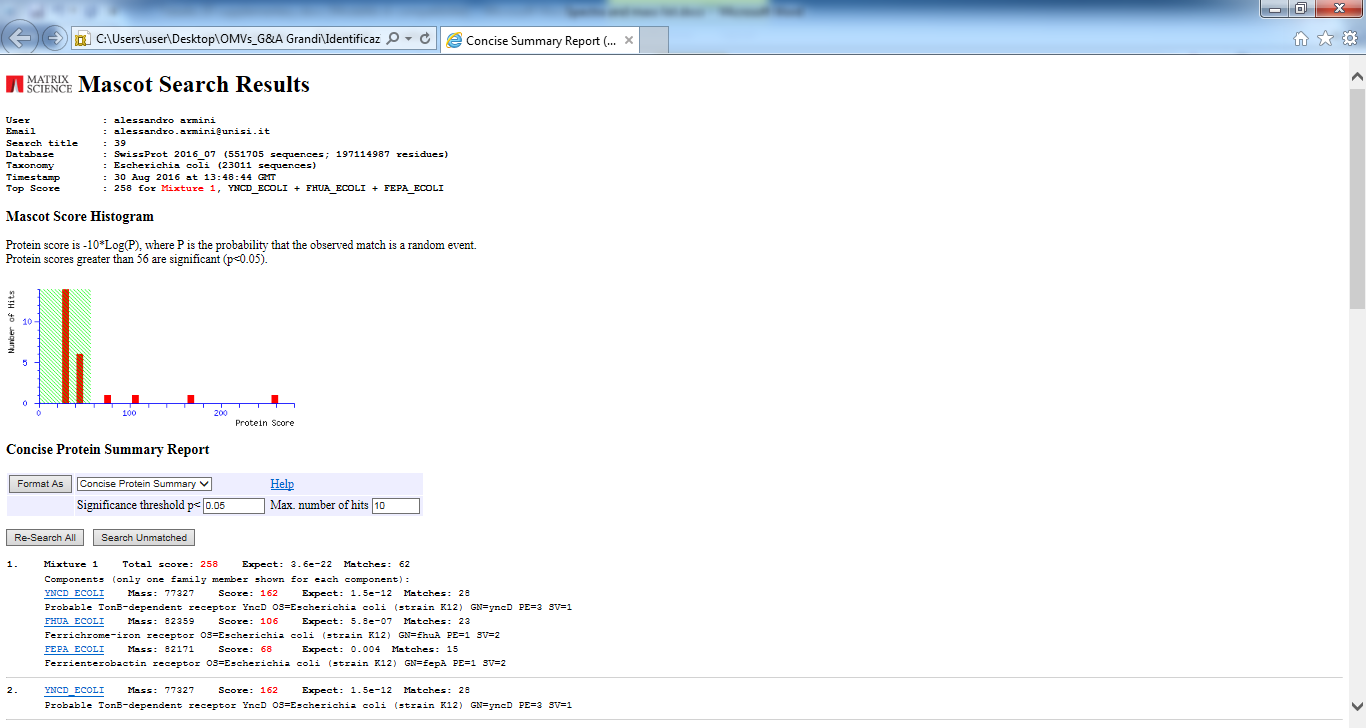


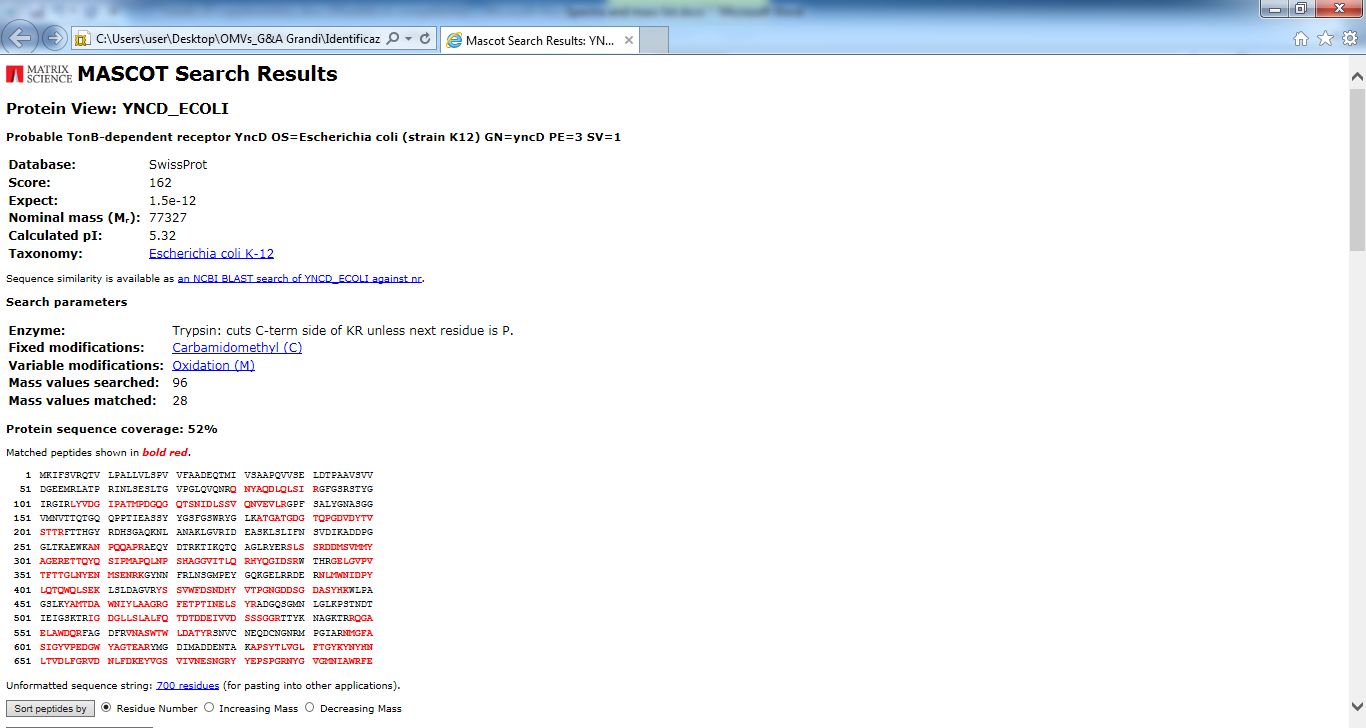


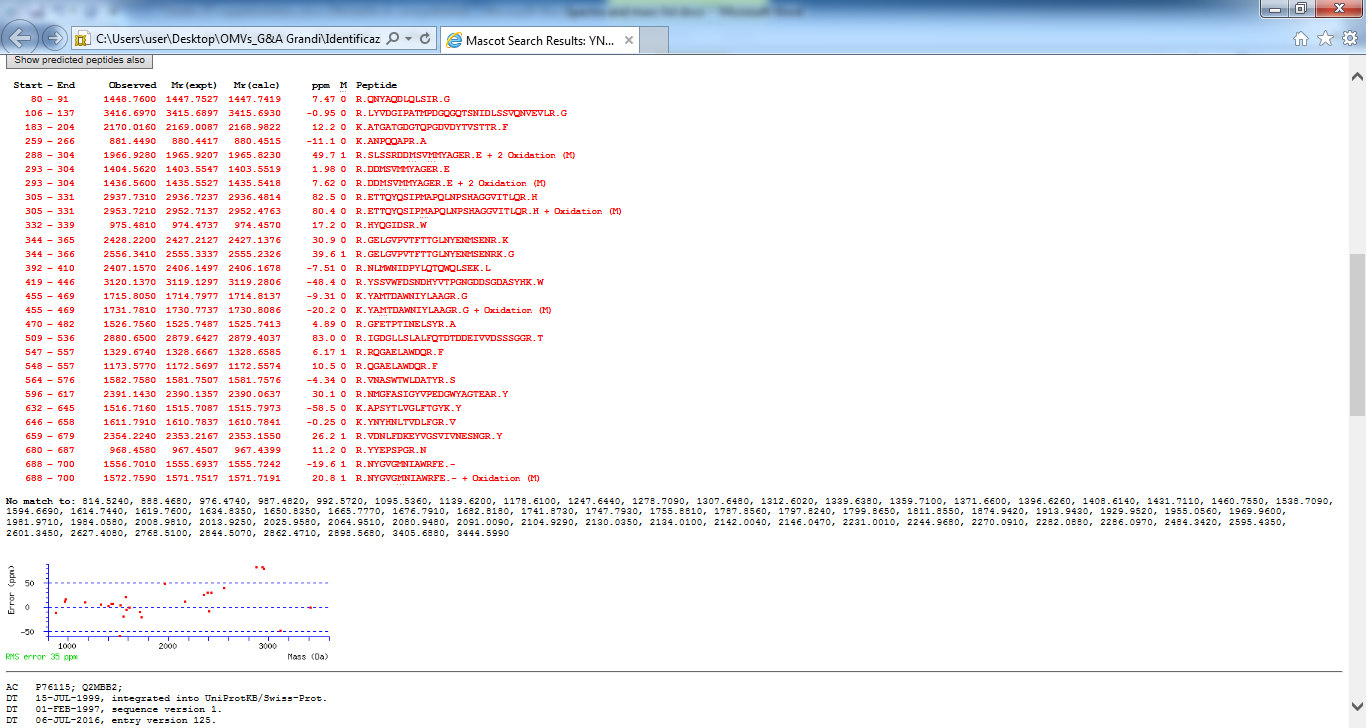


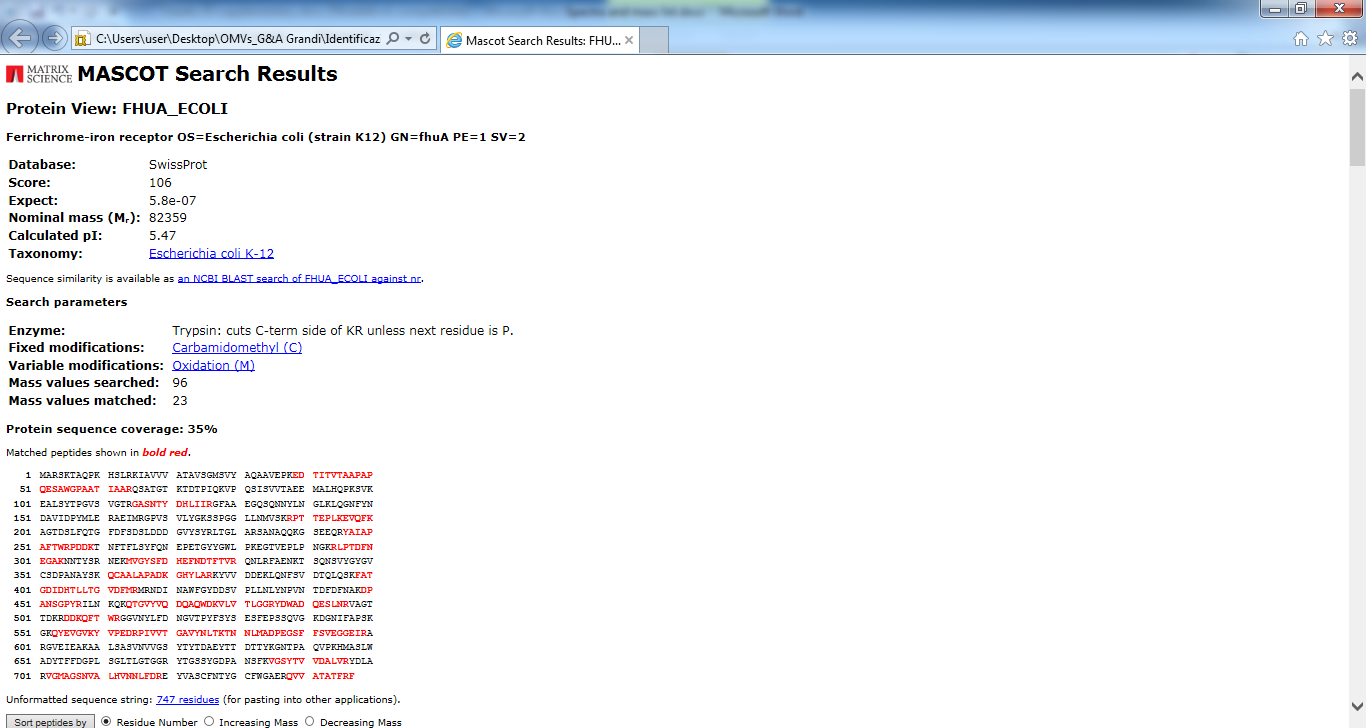


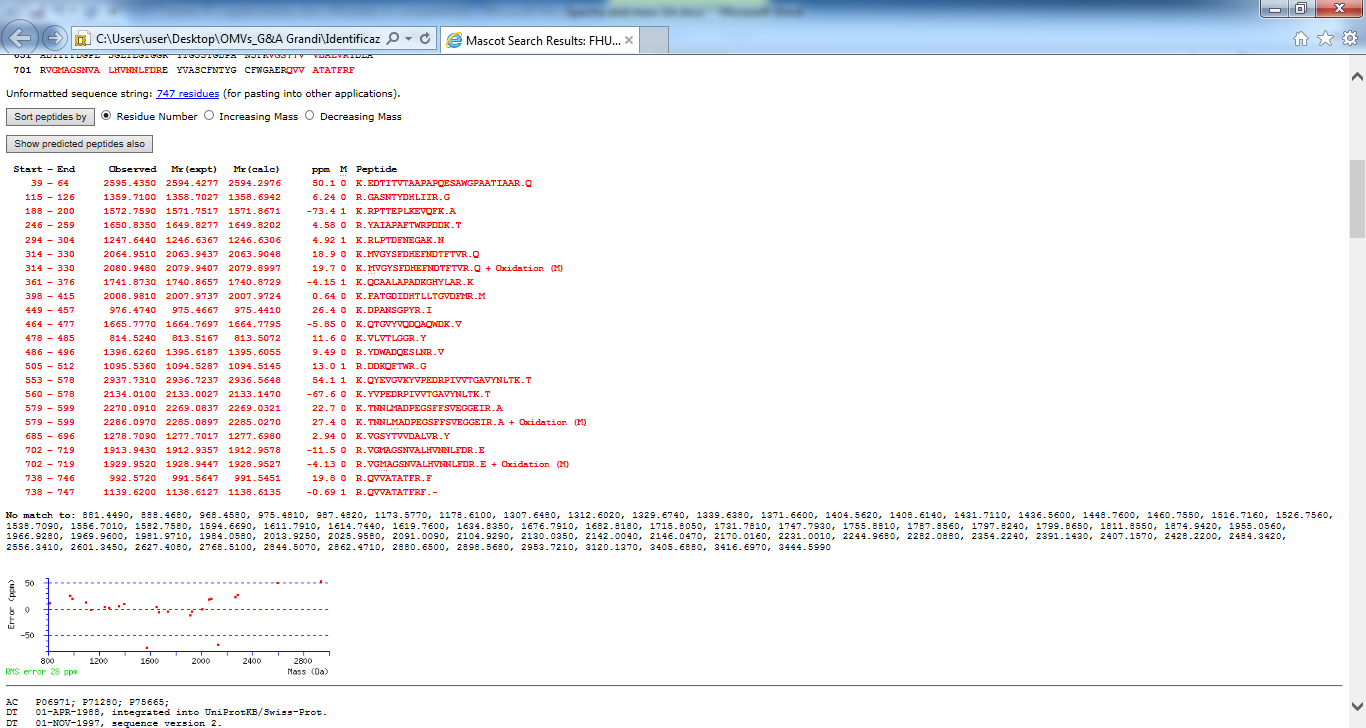


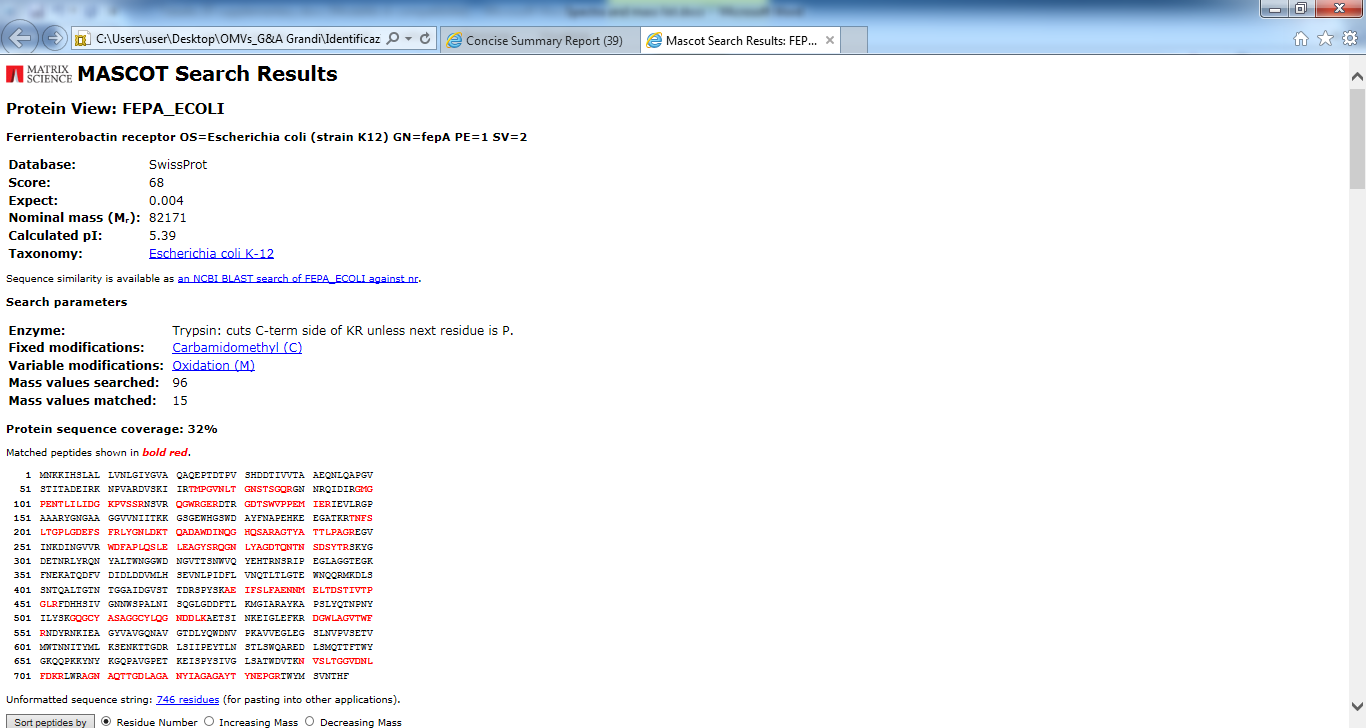

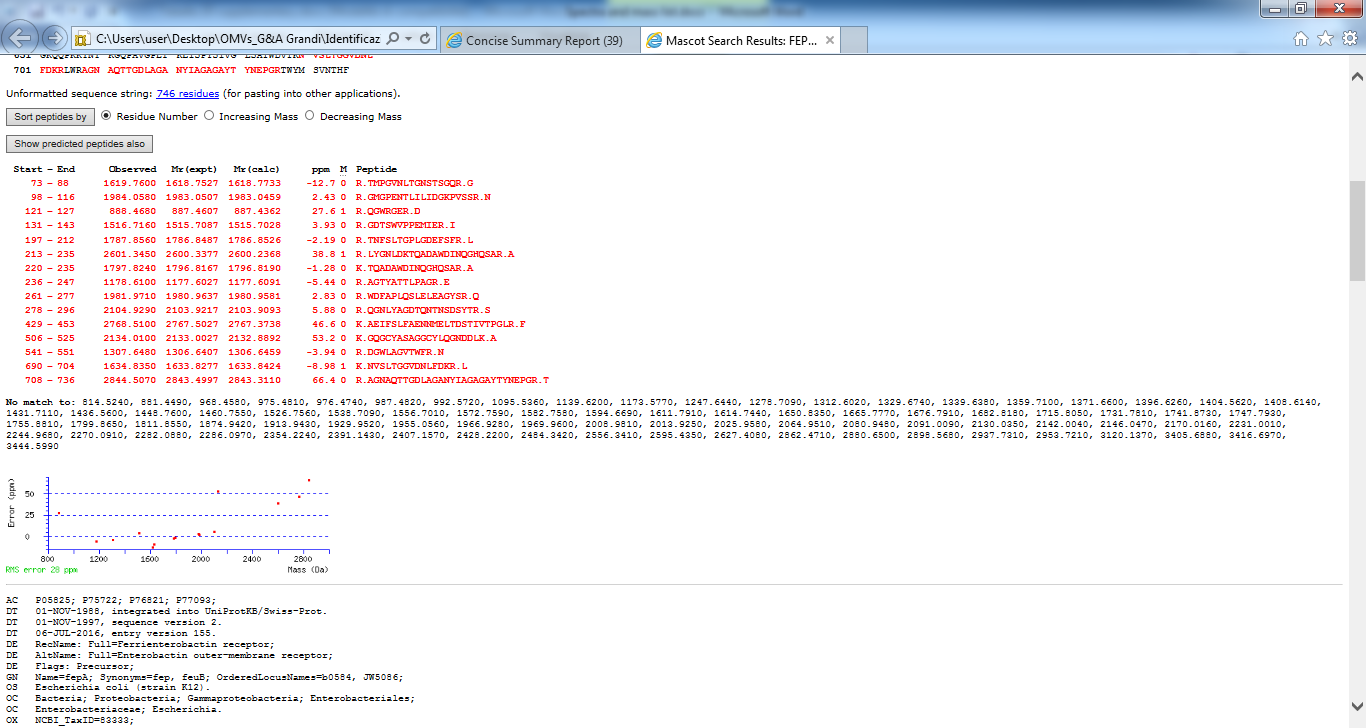


Spot N. 40 (FHUA_ECOLI)

m/z

1095.483

1247.536

1278.603

1359.578

1396.559

1436.669

1526.758

1572.821

1650.858

2134.038

2270.074


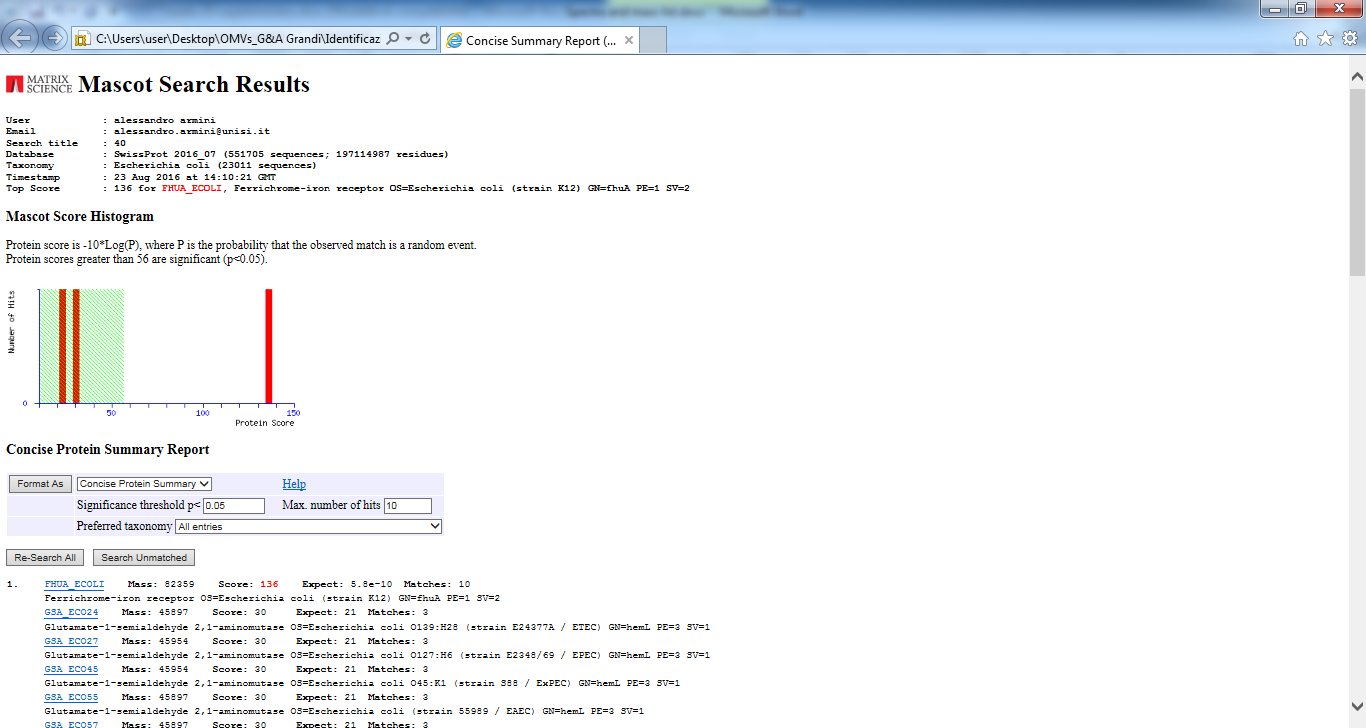


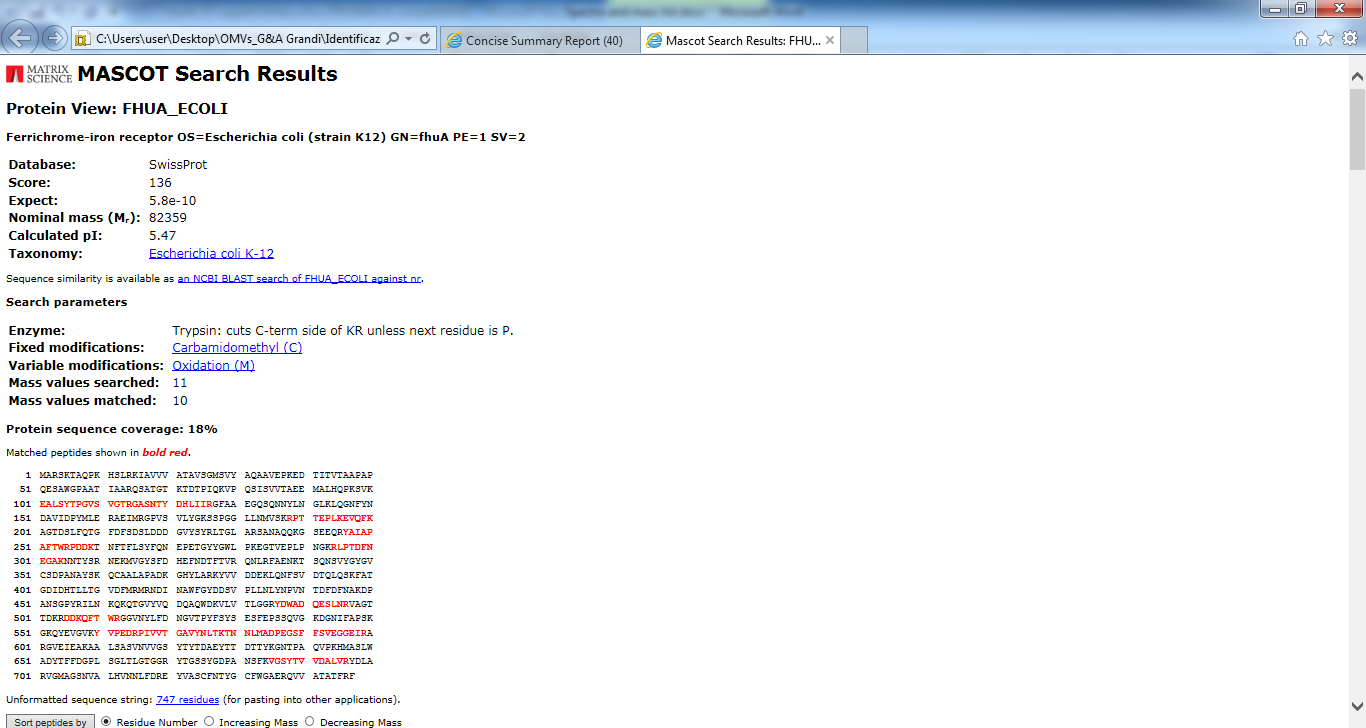

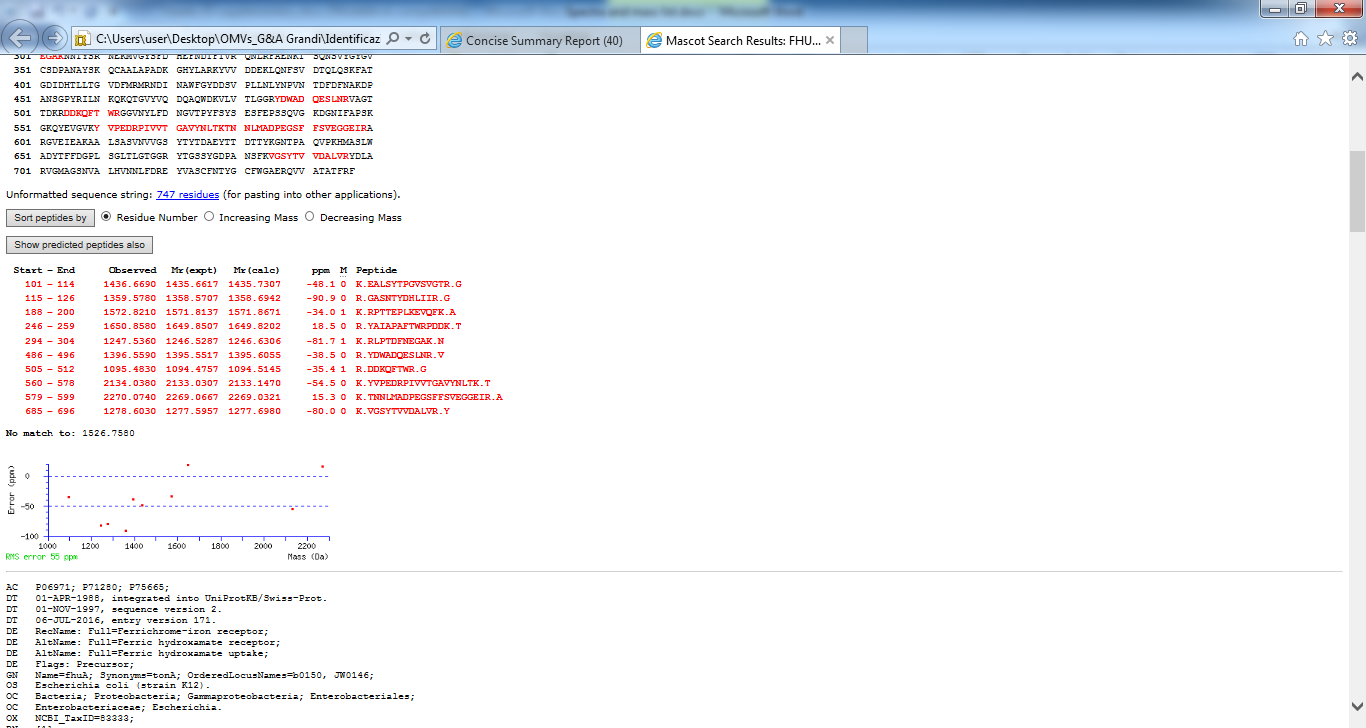


Spot N. 42 (FECA_ECOLI)

m/z

737.342

1012.551

1037.560

1244.594

1265.569

1330.660

1384.706

1579.815

1616.783

1697.773

2036.849

2110.983


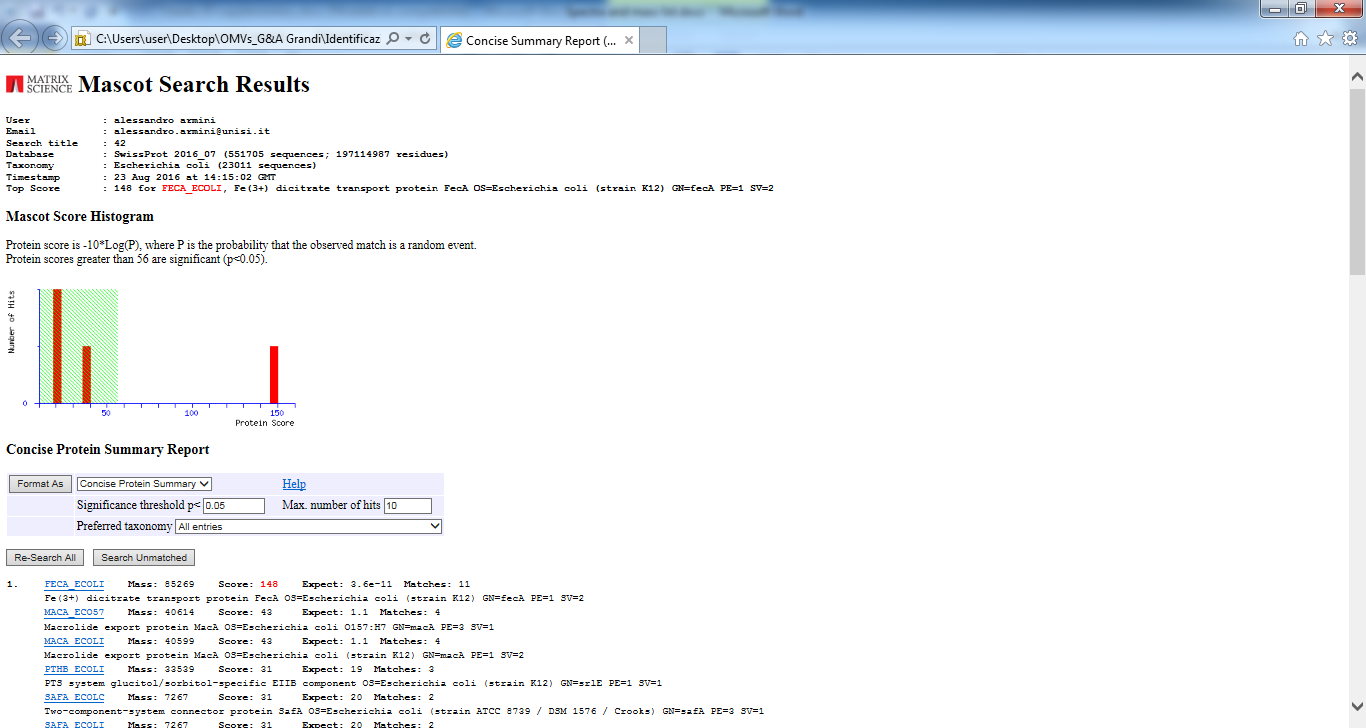


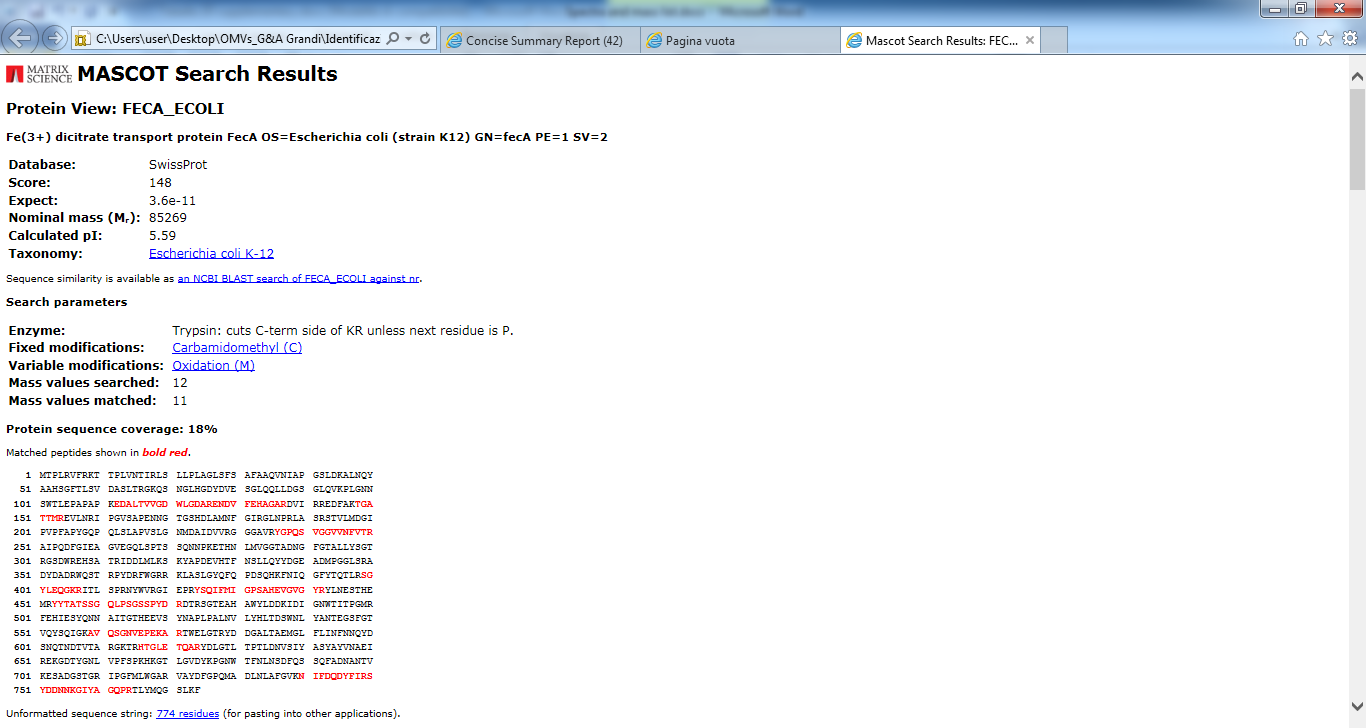

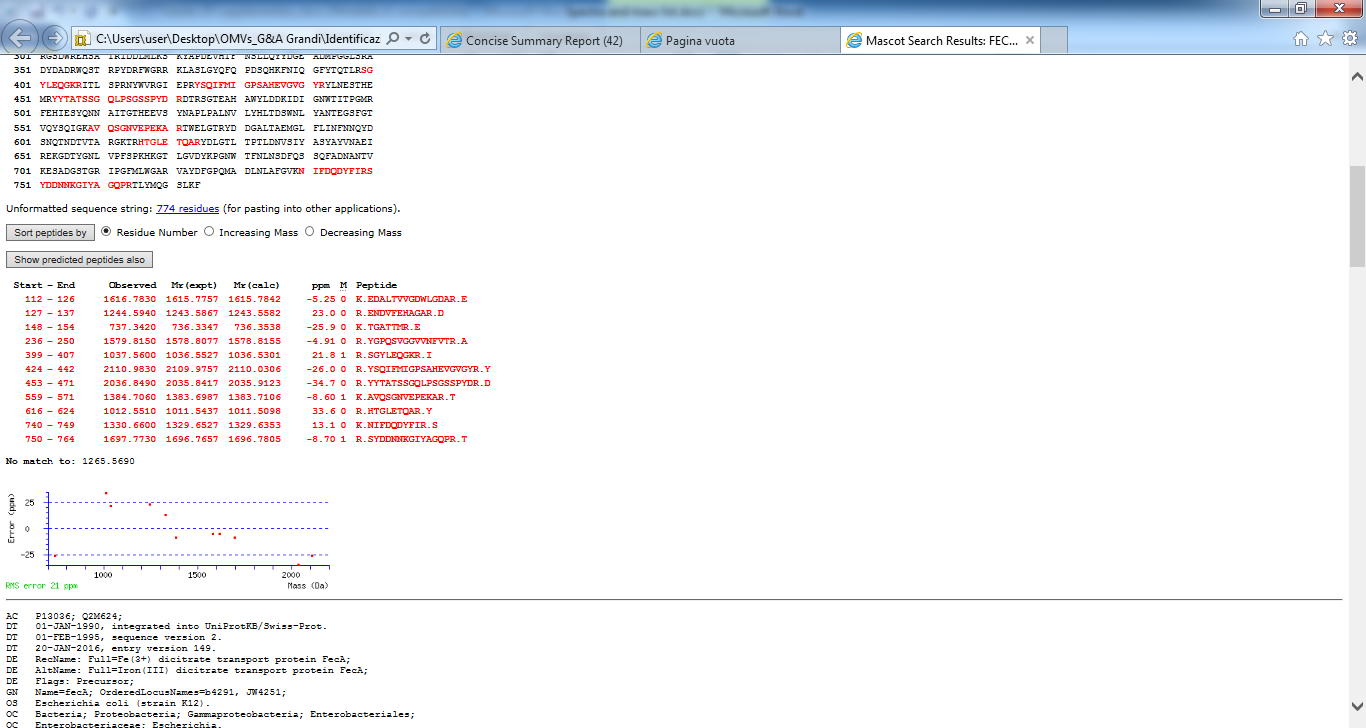


Spot N. 47 (BGLX_ECOLI)

m/z

1158.638

1226.620

1249.662

1307.536

1339.560

1377.692

1484.812

1581.762

1986.933

2227.018


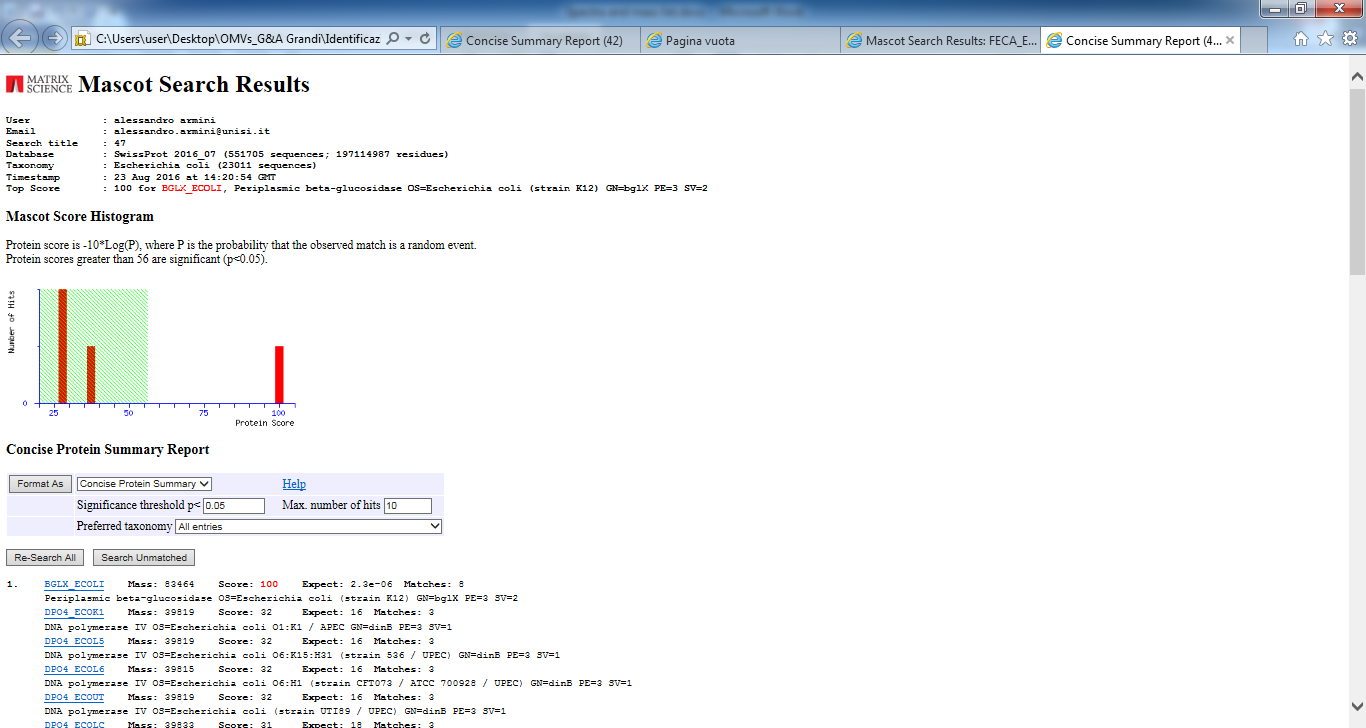


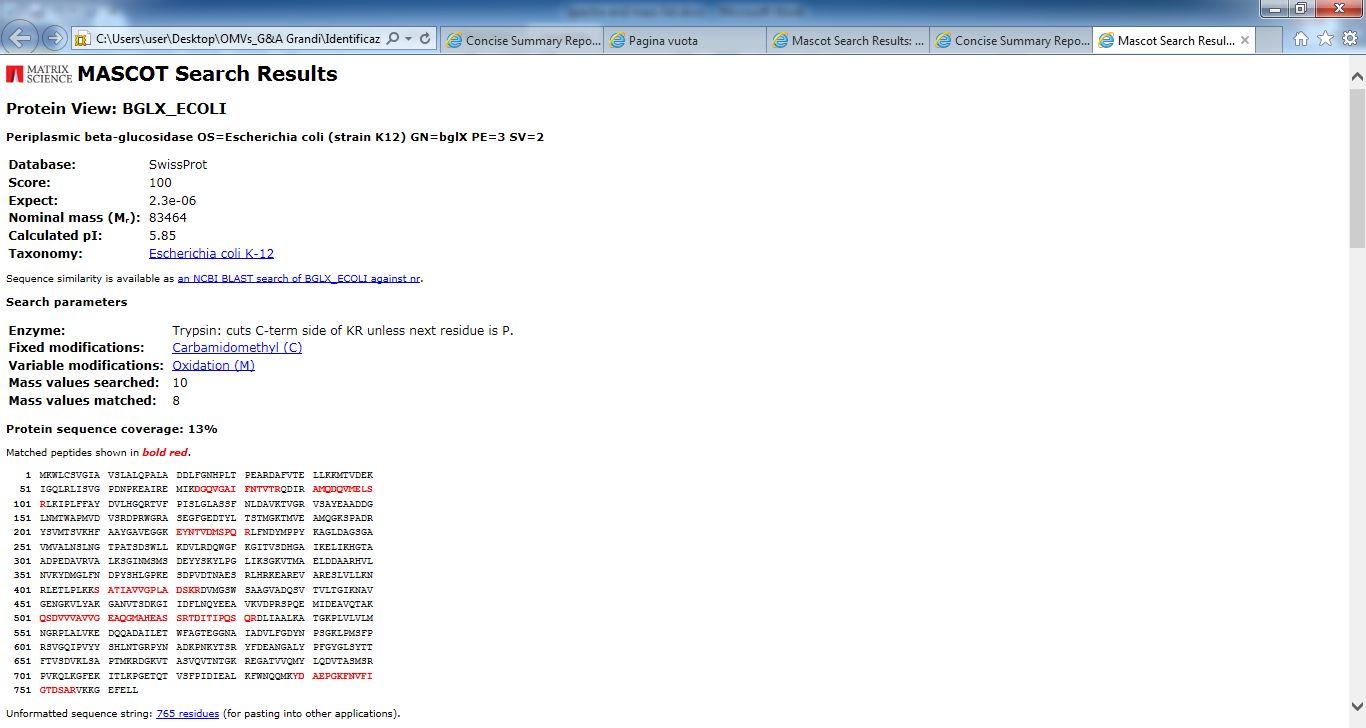

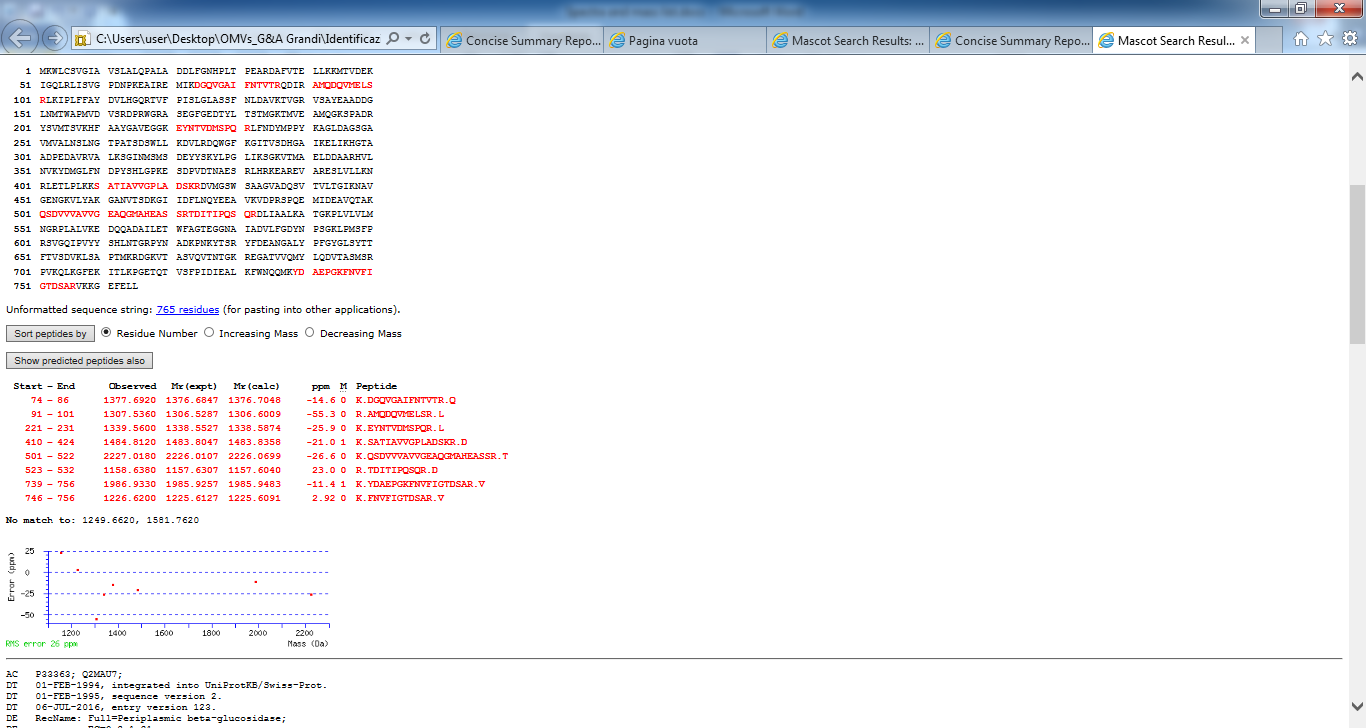


Spot N. 48 (BGLX_ECOLI)

m/z

1158.574

1211.711

1226.562

1238.599

1249.524

1250.649

1289.634

1307.585

1319.559

1339.590

1351.607

1377.704

1440.721

1484.831

1508.829

1521.741

1546.717

1581.711

1675.896

1790.813

1793.684

1878.920

1986.848

2226.969

2410.079

2878.339


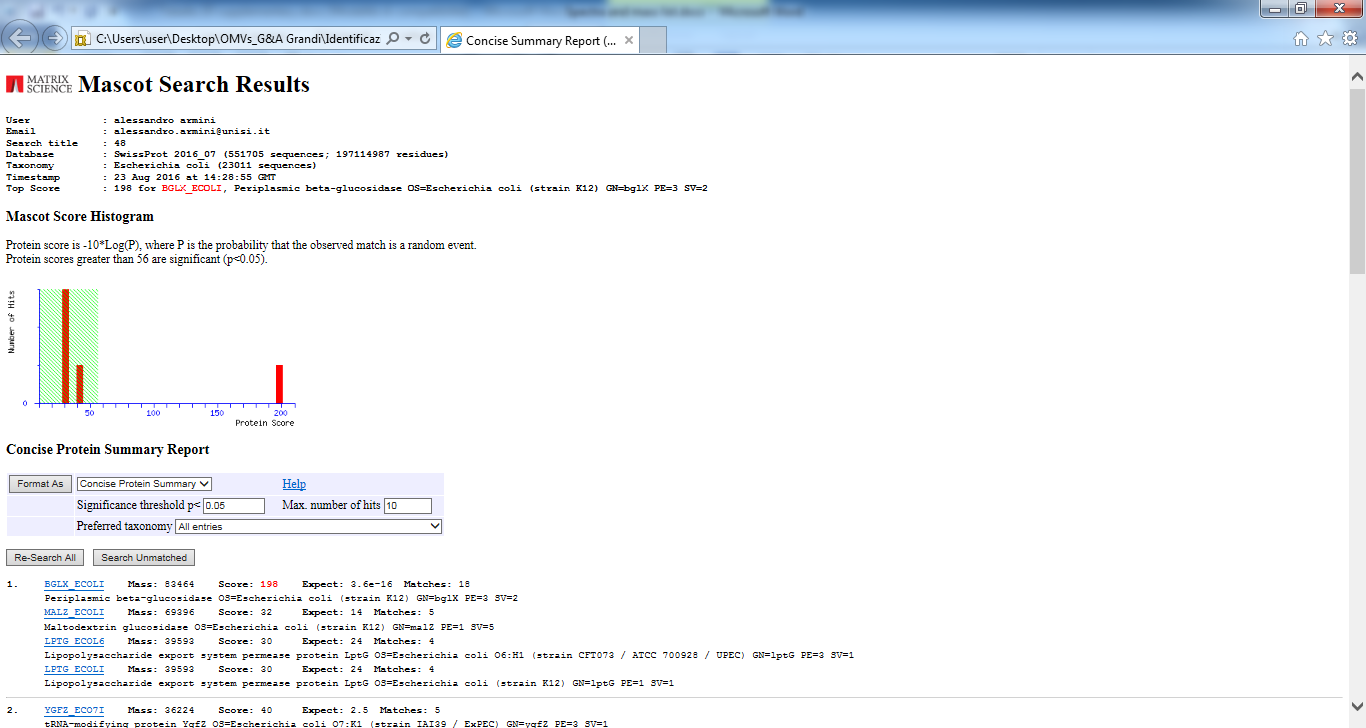


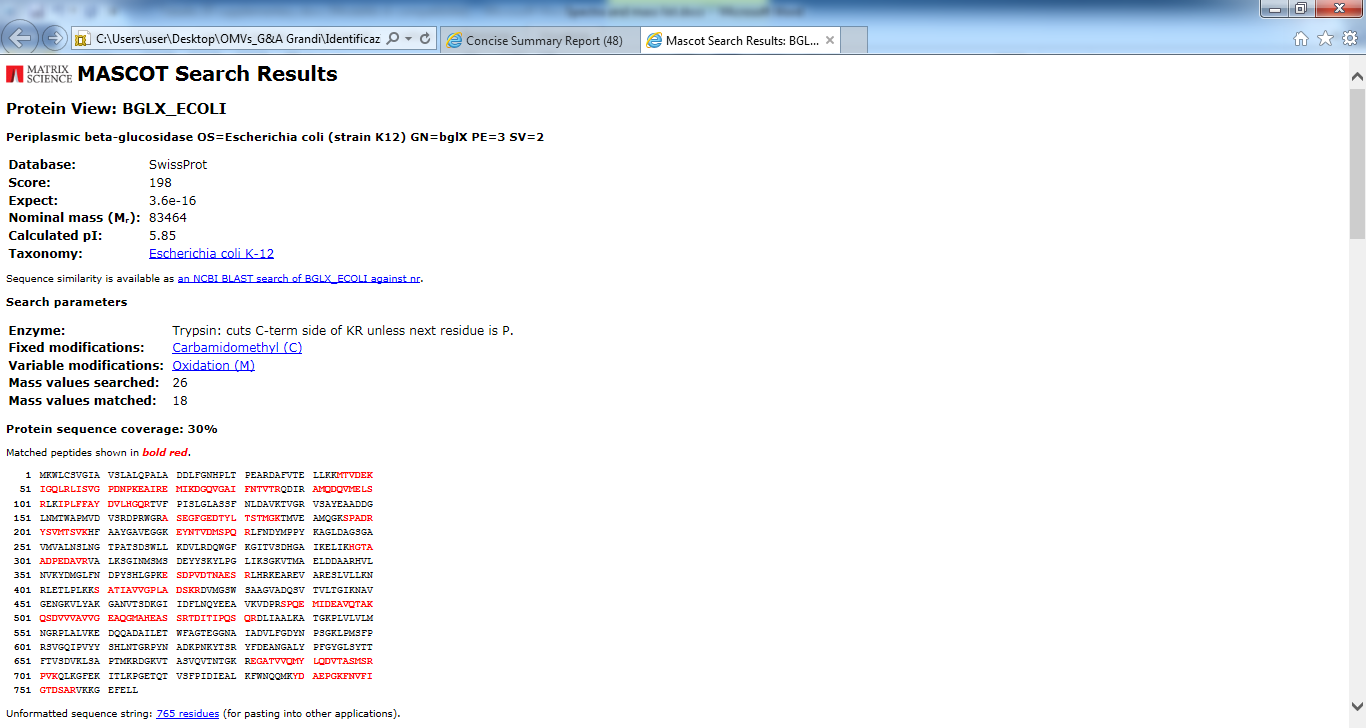

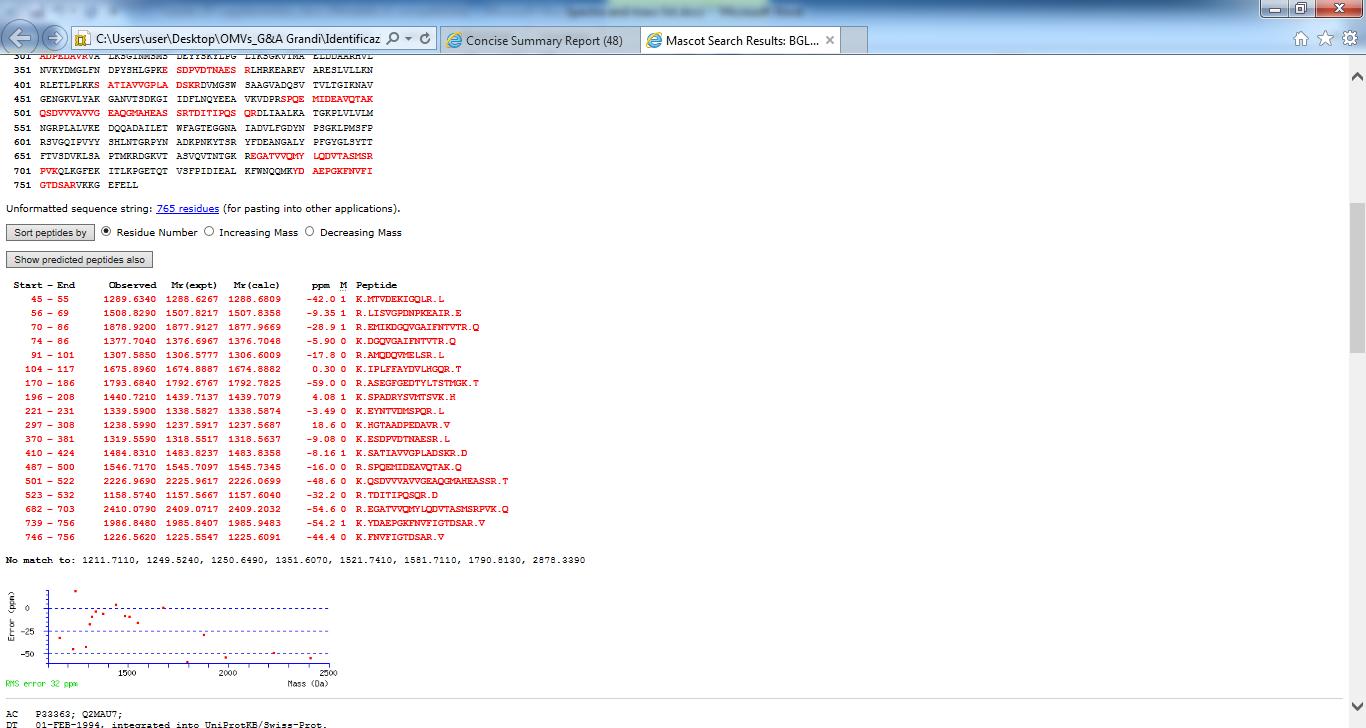


Spot N. 53 (PRC_ECOLI)

m/z

851.424

1095.663

1117.569

1139.647

1148.611

1163.634

1243.670

1269.534

1441.661

1882.822

1888.946

1921.763

1950.038

1954.995

2033.000

2071.982

2088.008

2151.137

2610.365


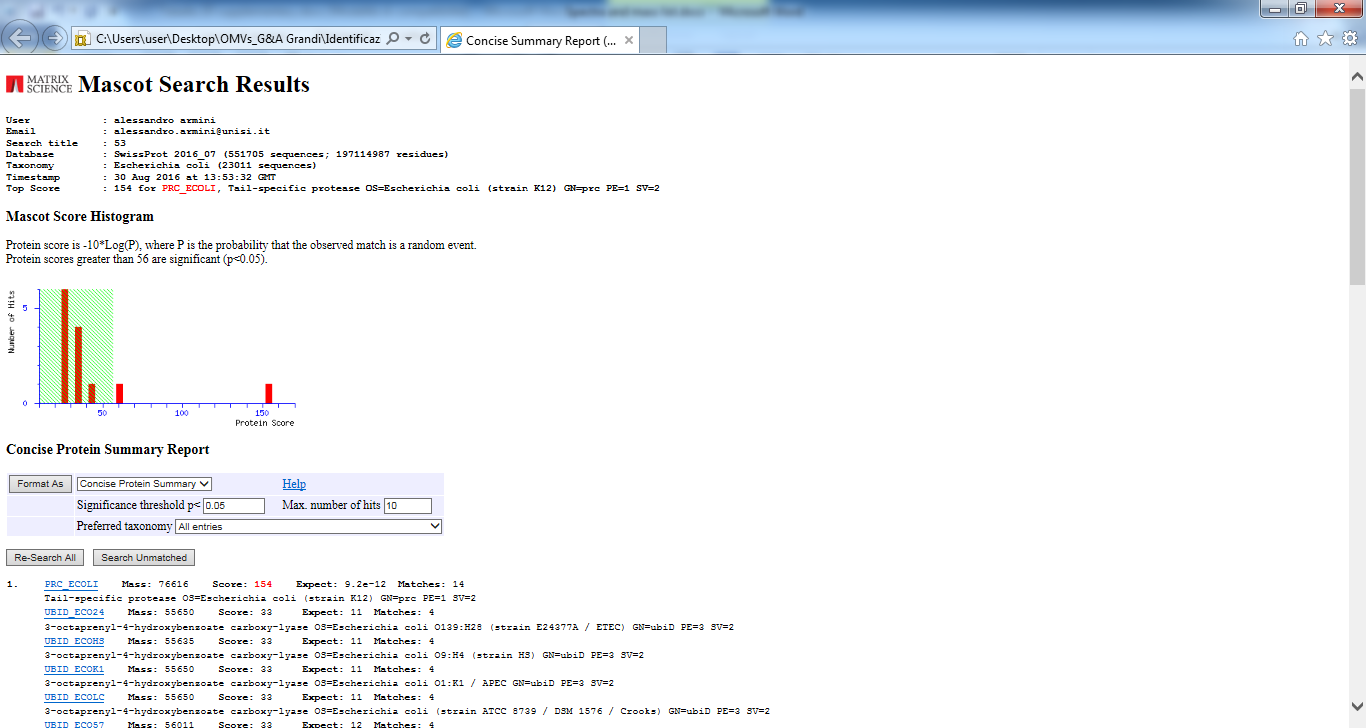


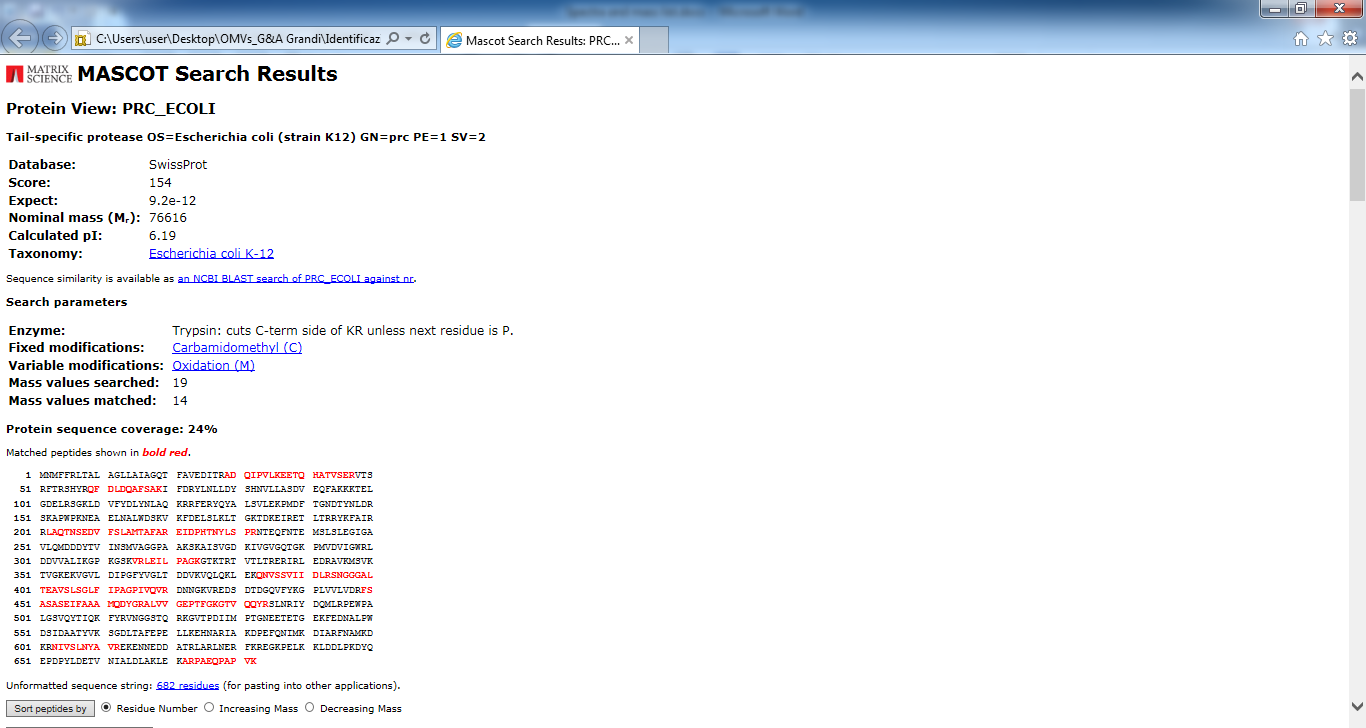

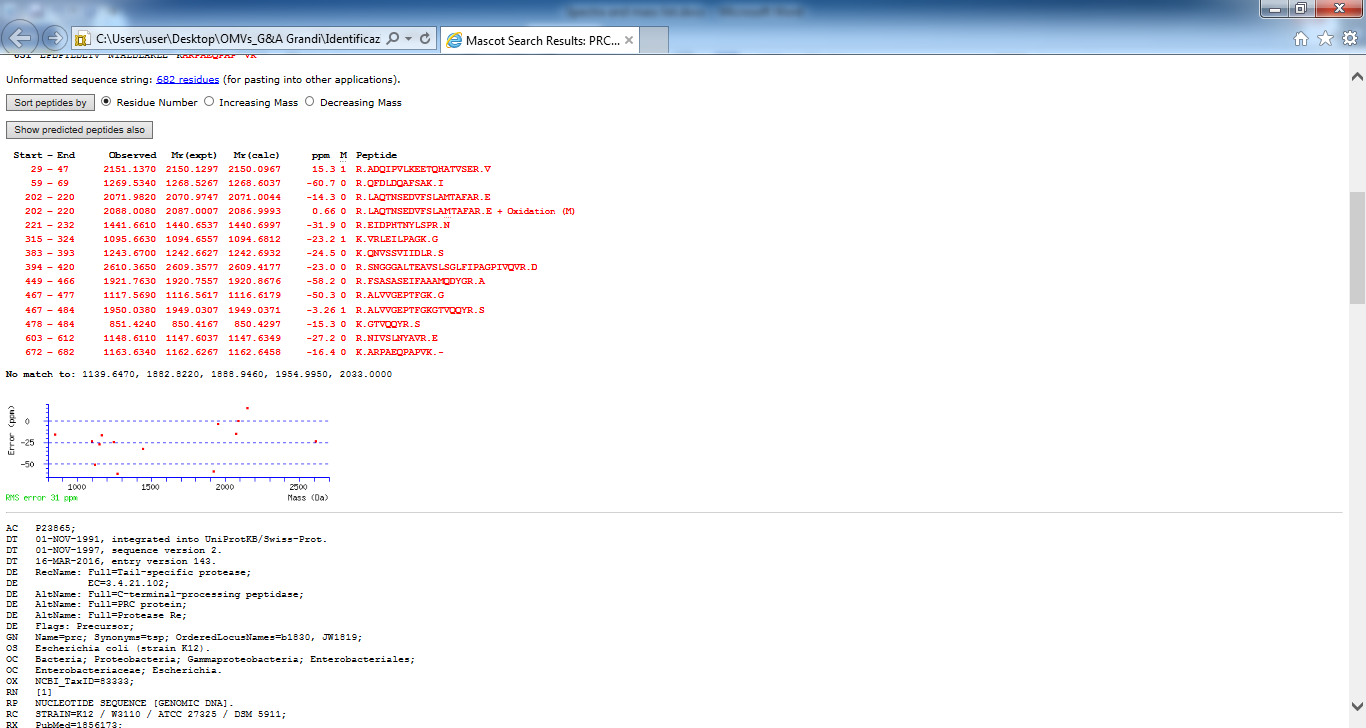


Spot N. 55 (FHUE_ECOLI)

m/z

1411.706

1470.805

1489.006

1519.862

1569.886

1805.062

1885.963


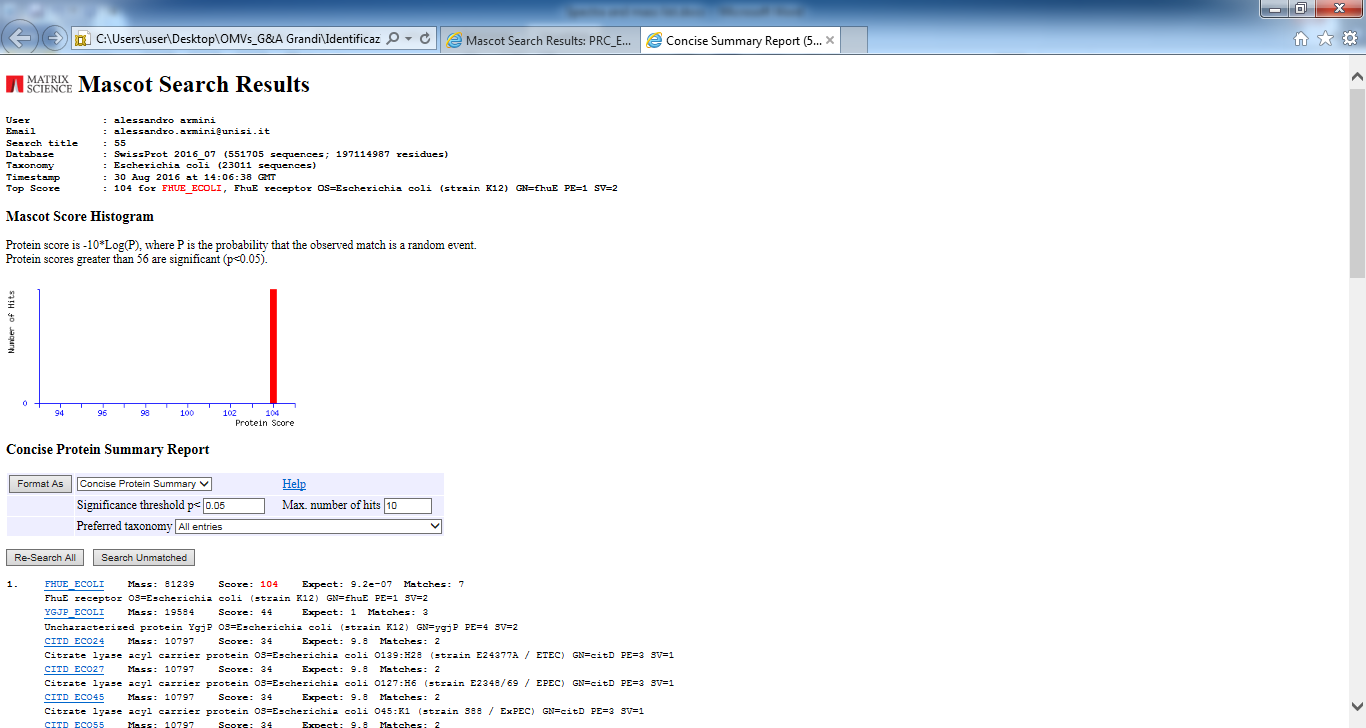


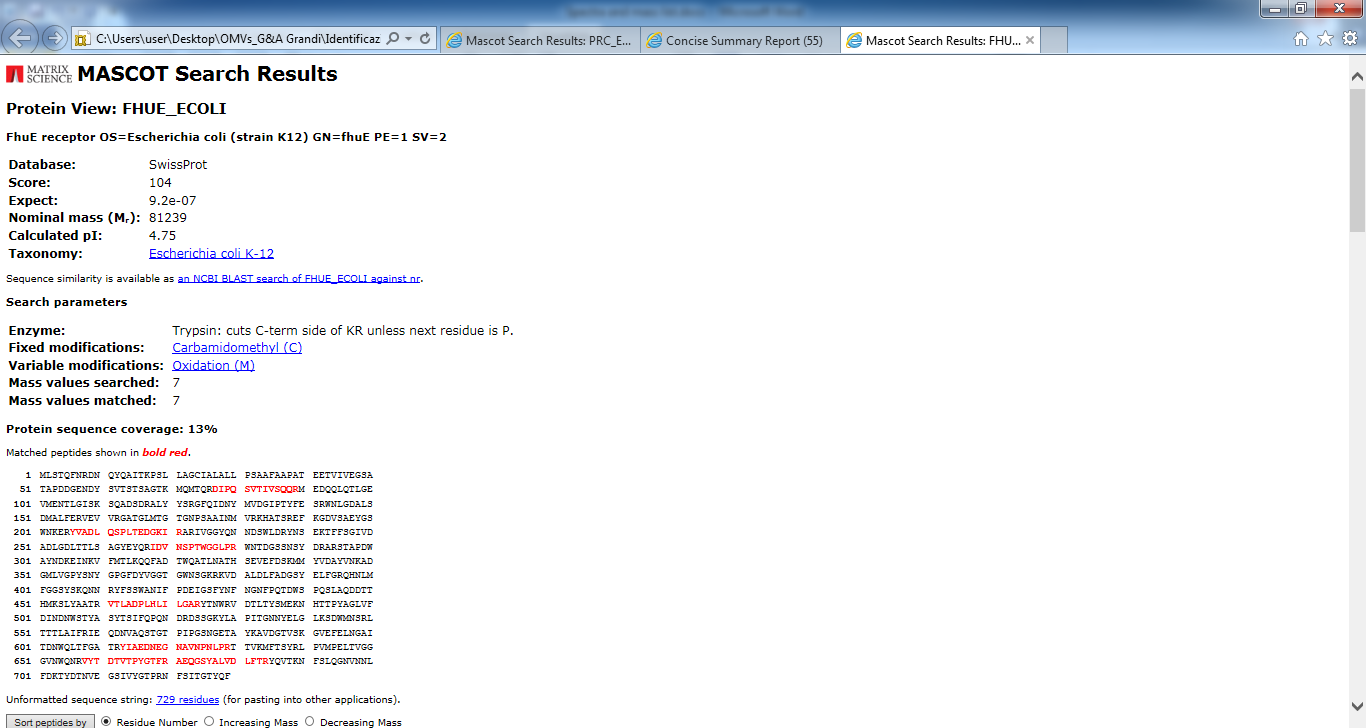

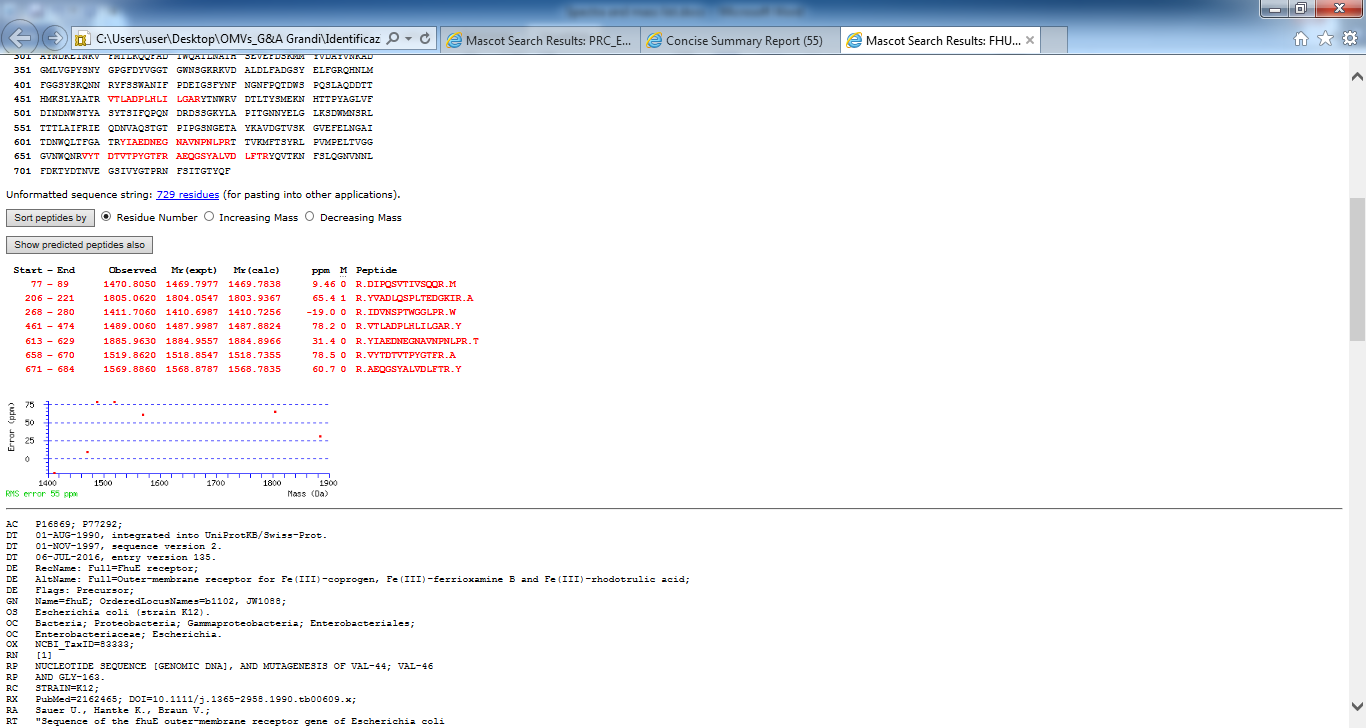


Spot N. 57 (LPOA_ECOLI)

m/z

1006.625

1106.510

1158.630

1339.647

1483.716

1540.800

1652.834

1740.887

2136.071

2271.081


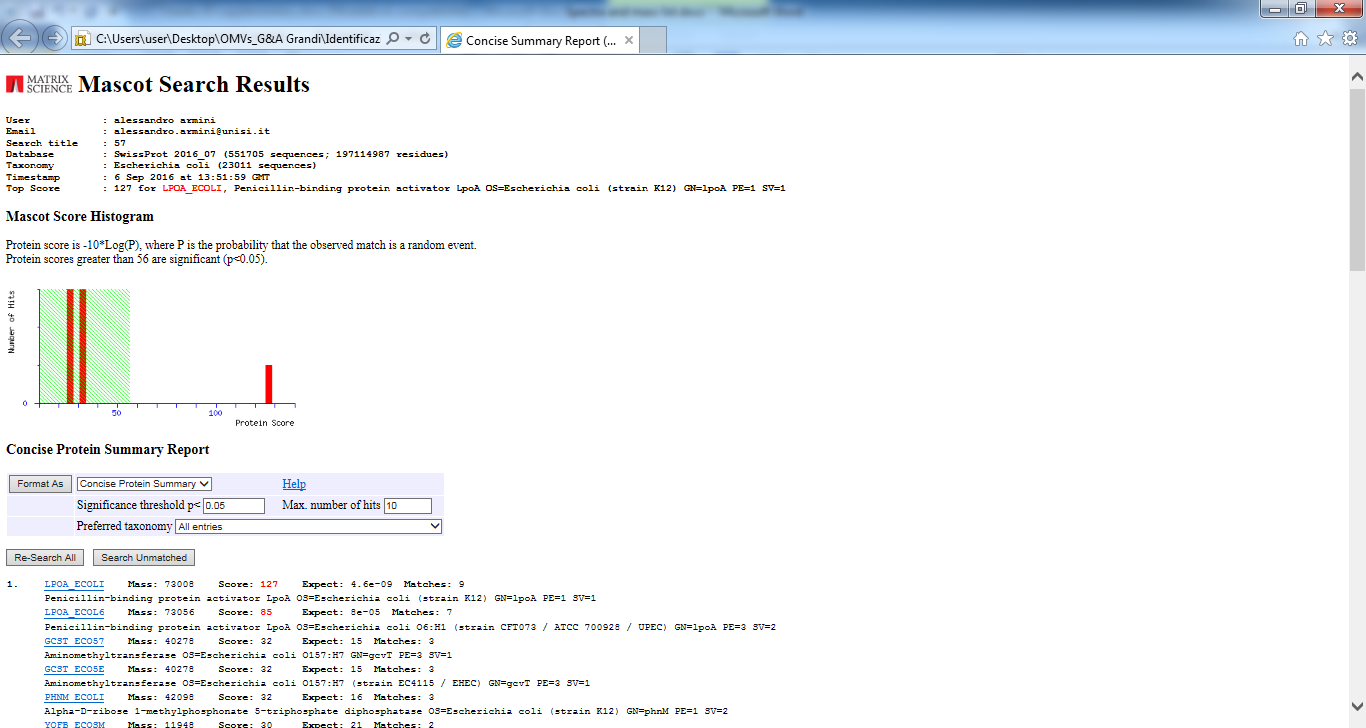


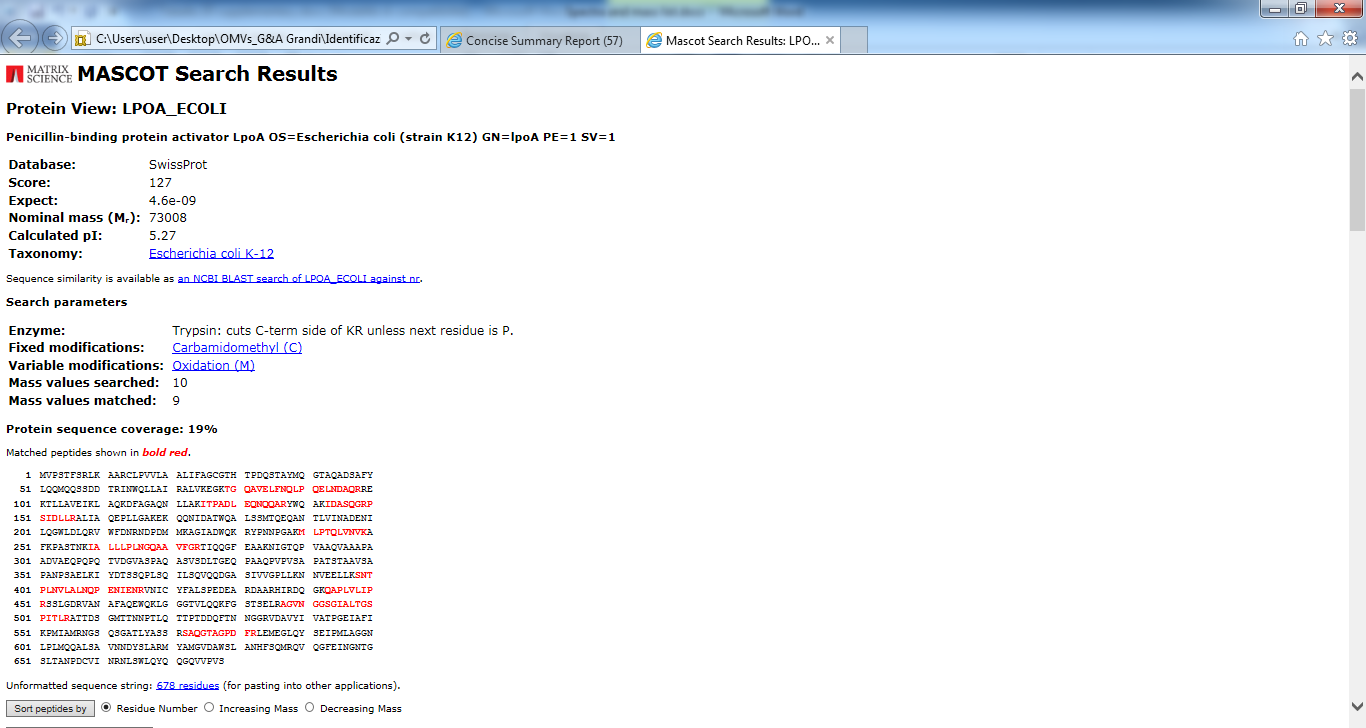

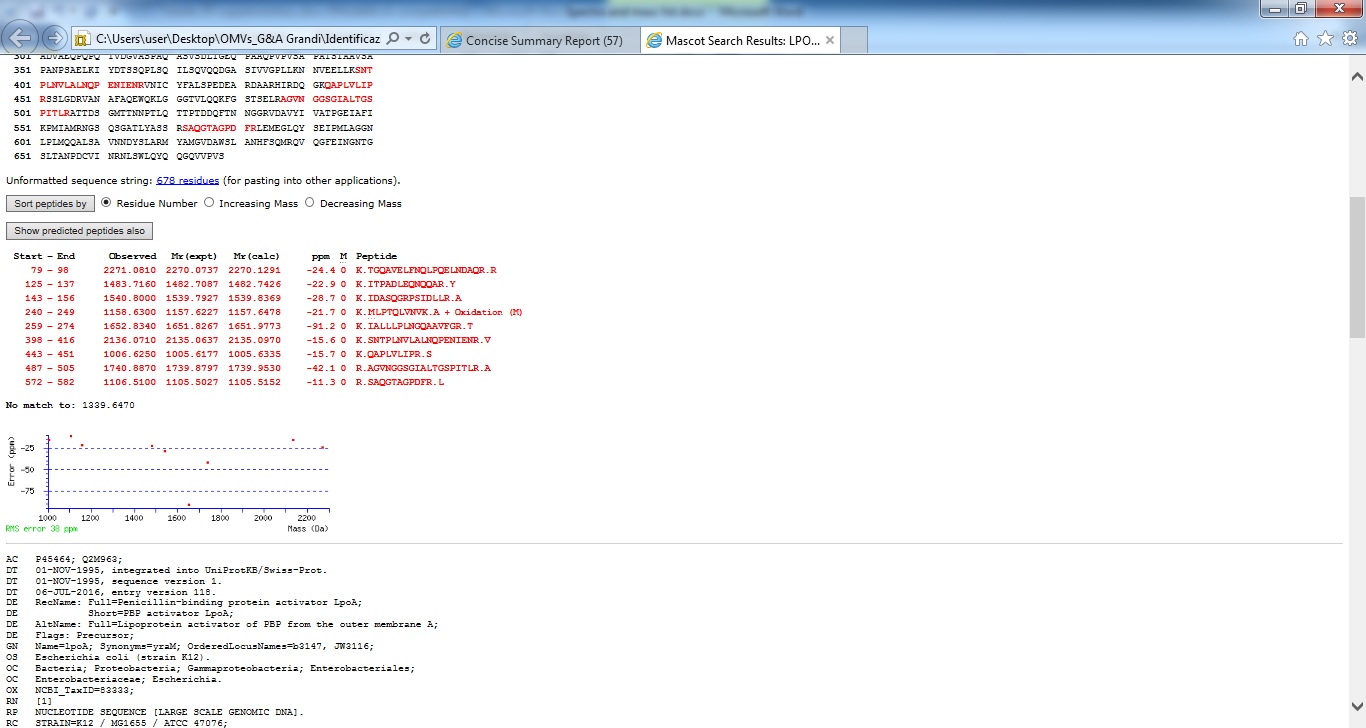


Spot N. 58 (LPOA_ECOLI)

m/z

865.470

896.459

1006.665

1044.599

1106.551

1126.697

1158.700

1291.669

1398.683

1483.792

1540.885

1652.930

1653.942

1667.764

1741.989

1797.878

1906.967

1938.965

2136.129

2271.168

2427.265

2618.289

2619.295

3056.473


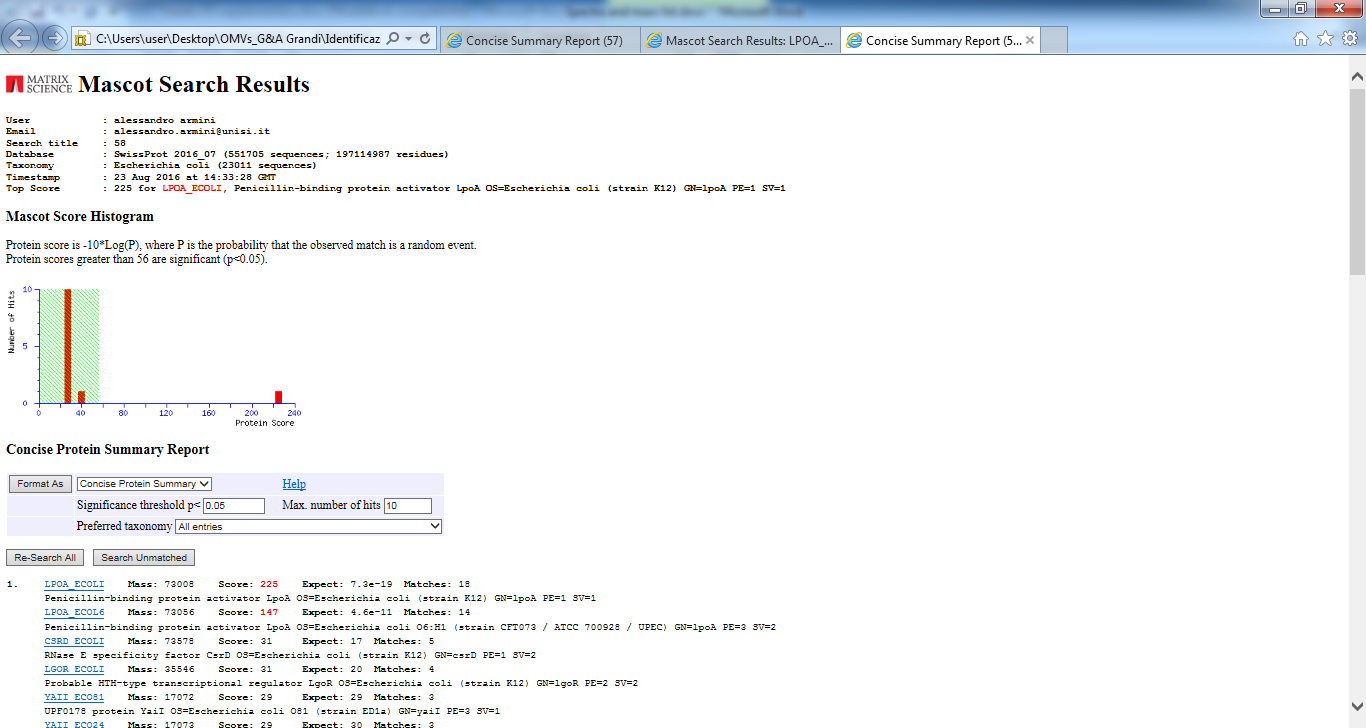


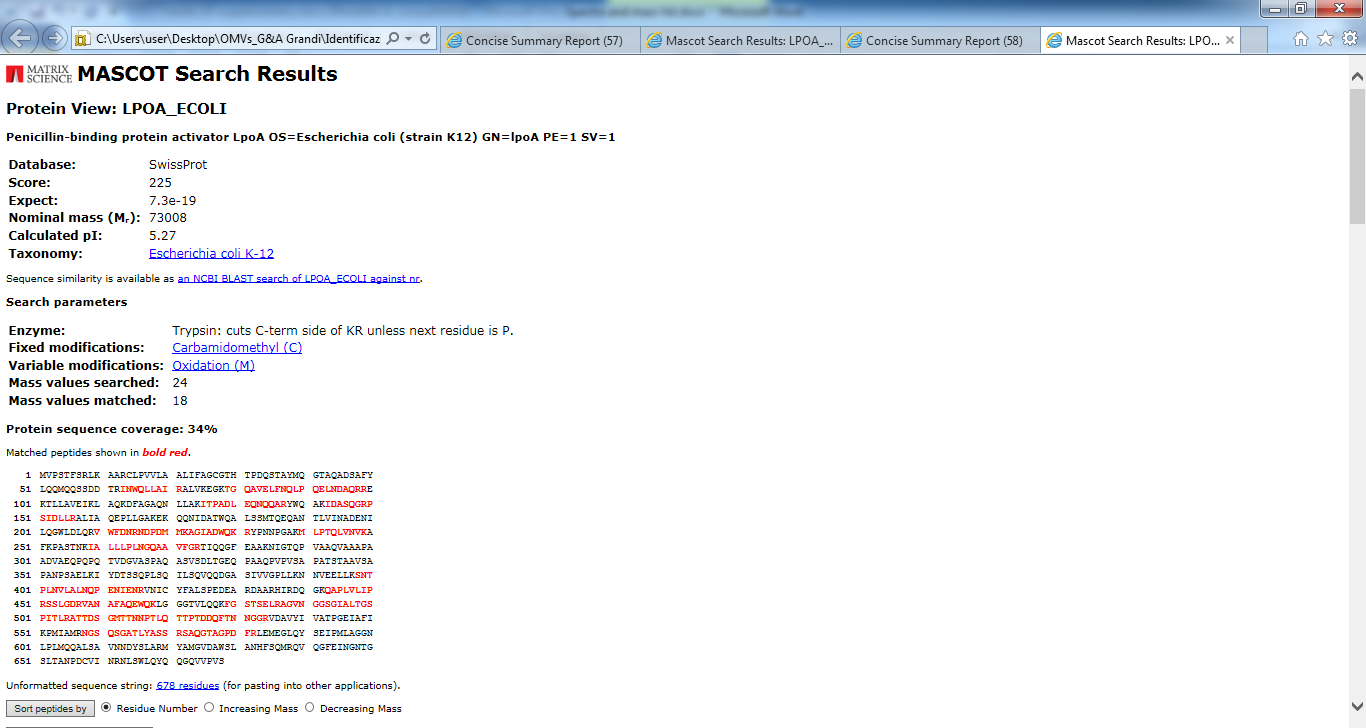

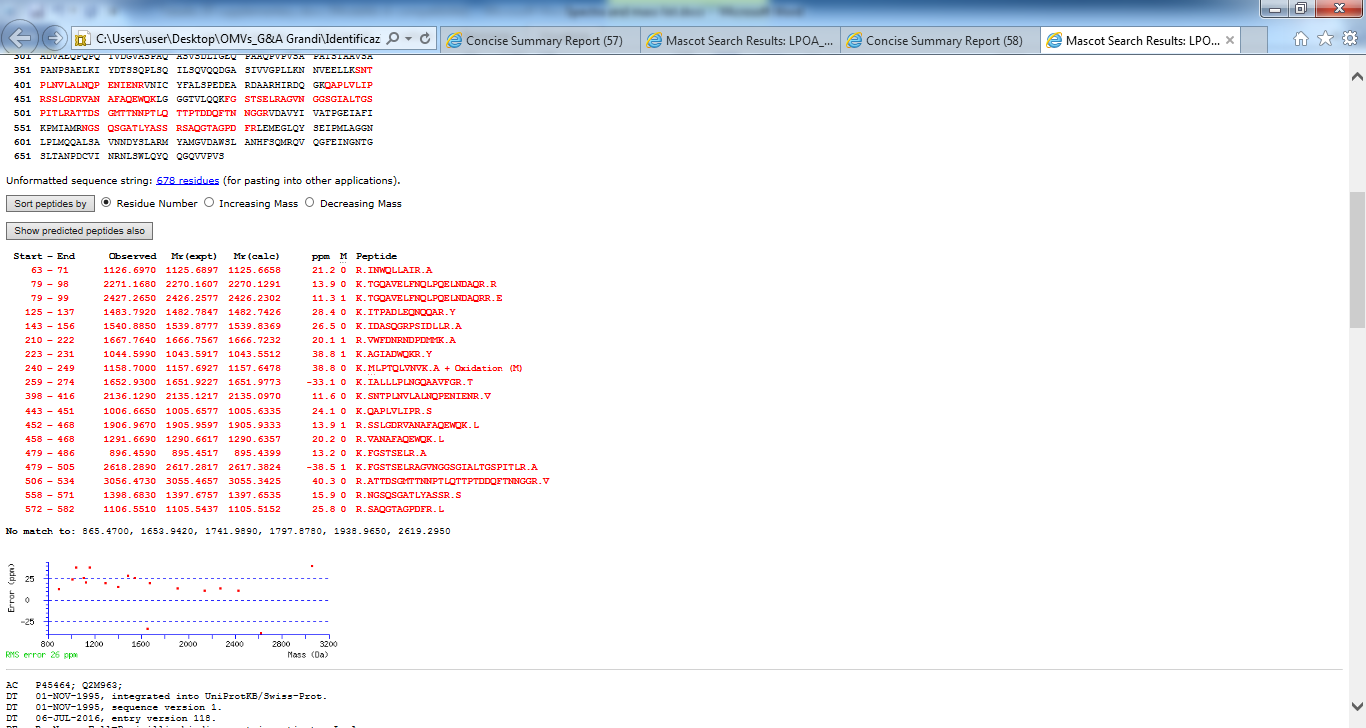


Spot N. 59 (LPOA_ECOLI)

m/z

836.436

853.483

860.448

888.493

896.480

1006.676

1016.567

1092.603

1106.557

1126.708

1138.703

1147.634

1158.701

1170.702

1223.776

1291.682

1398.696

1399.687

1483.787

1495.787

1504.759

1540.894

1628.799

1652.882

1653.907

1667.778

1683.775

1726.863

1741.007

1742.007

1783.888

1797.916

1809.916

2098.222

2136.204

2148.207

2215.107

2271.274

2412.729

2427.416

2505.538

2604.464

2618.507

2630.484

3056.342

3072.478


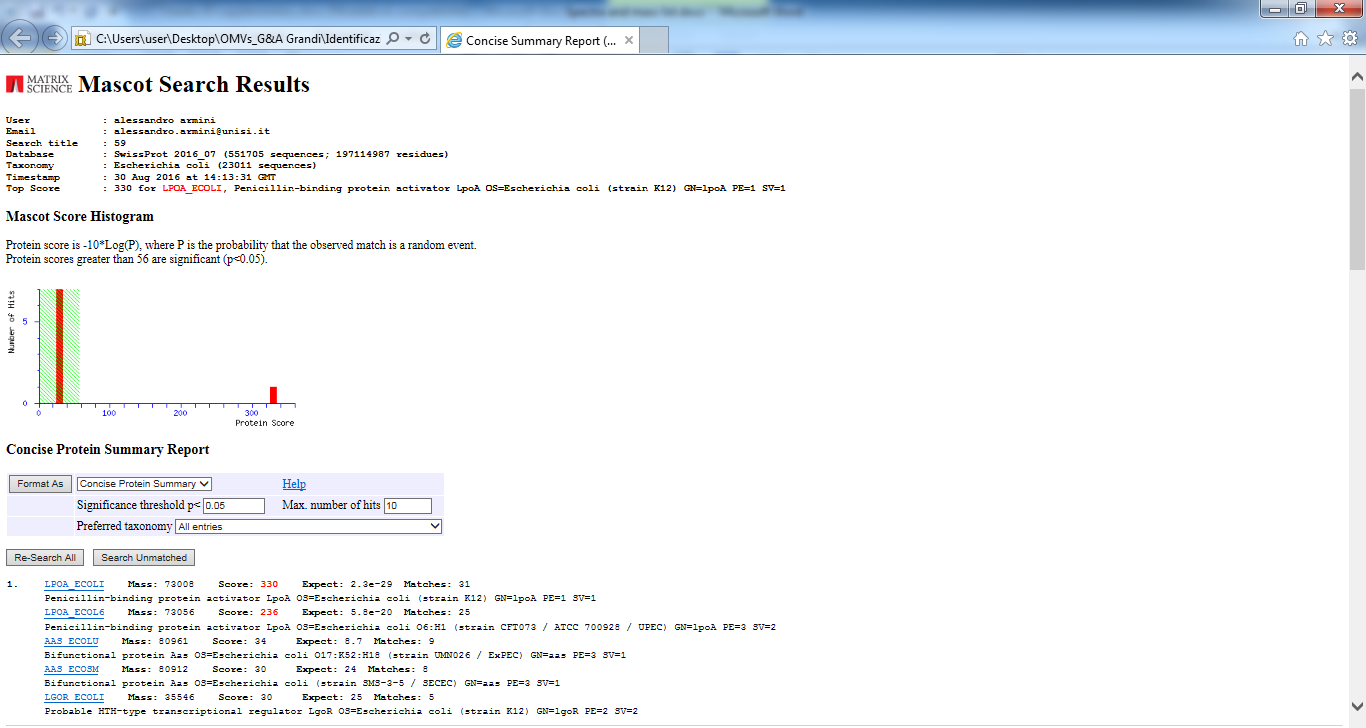


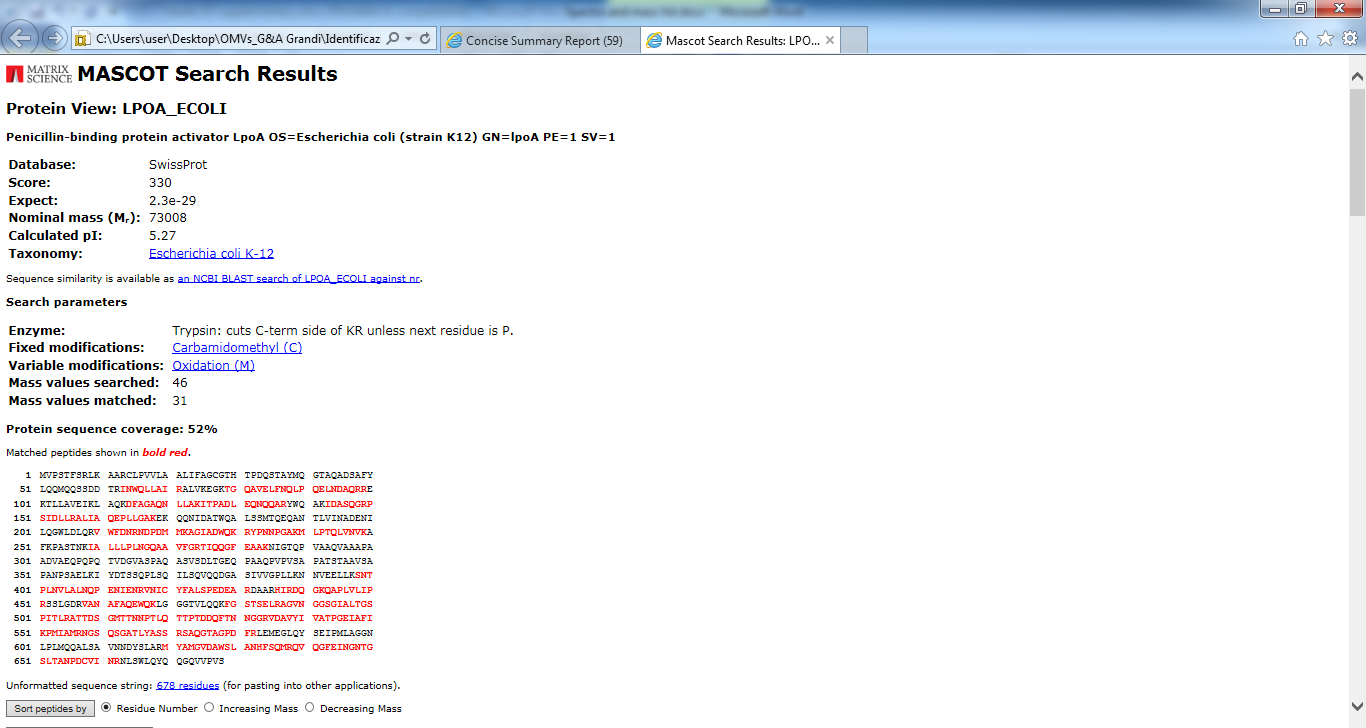


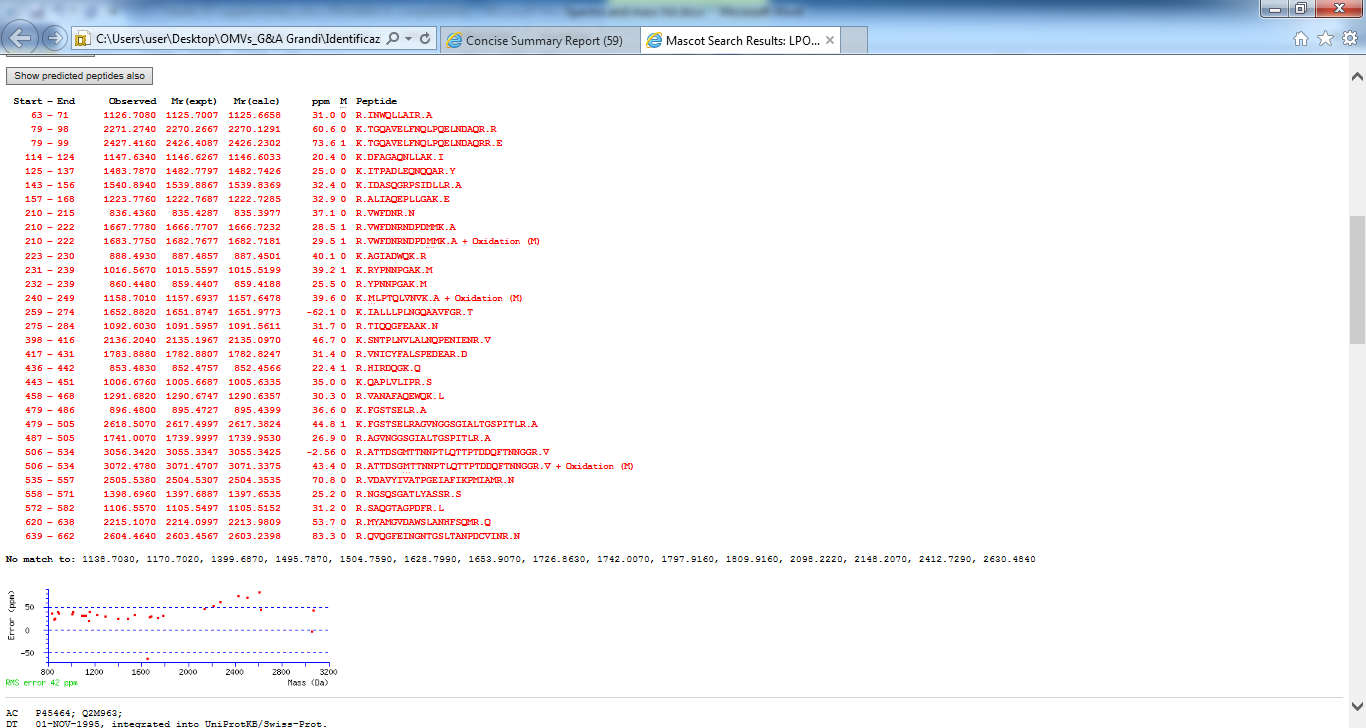


Spot N. 60 (LPOA_ECOLI)

m/z

896.428

1006.627

1106.530

1126.636

1158.647

1223.696

1291.567

1399.636

1483.727

1540.806

1652.947

1653.948

1741.911

1797.839

2136.105

2271.167


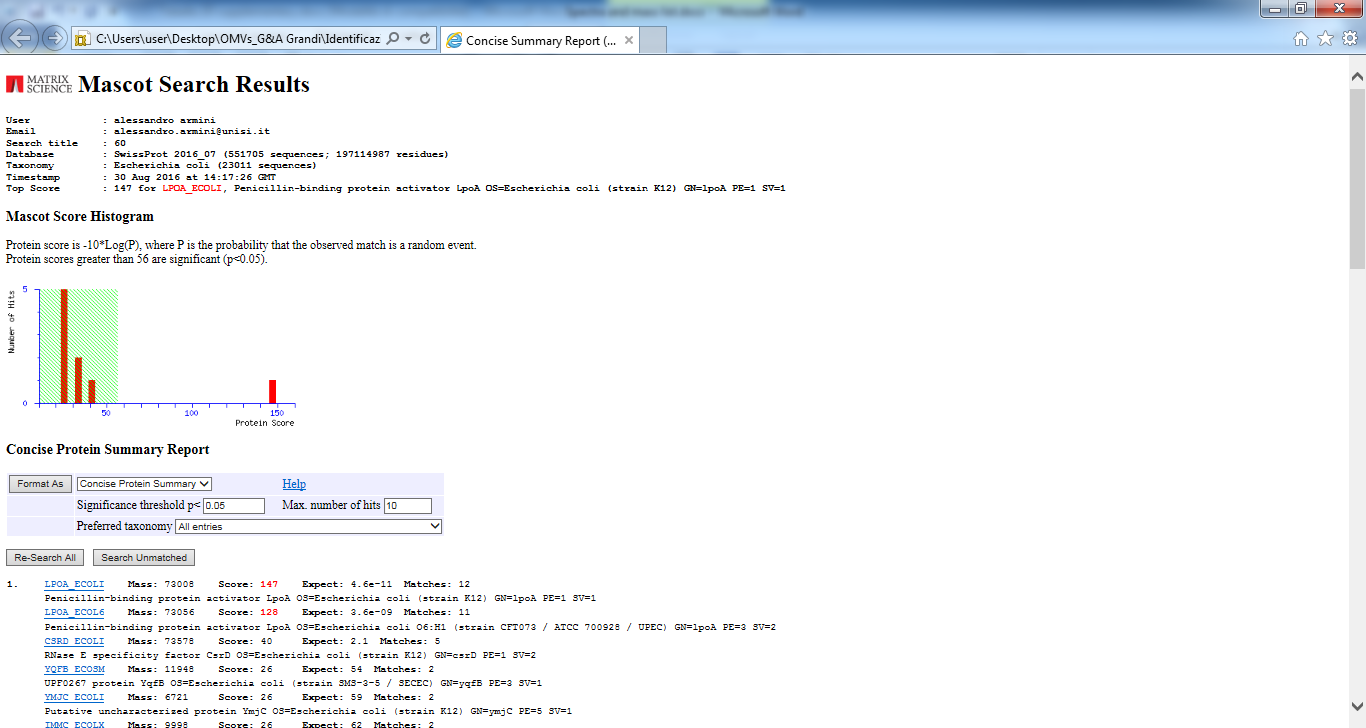


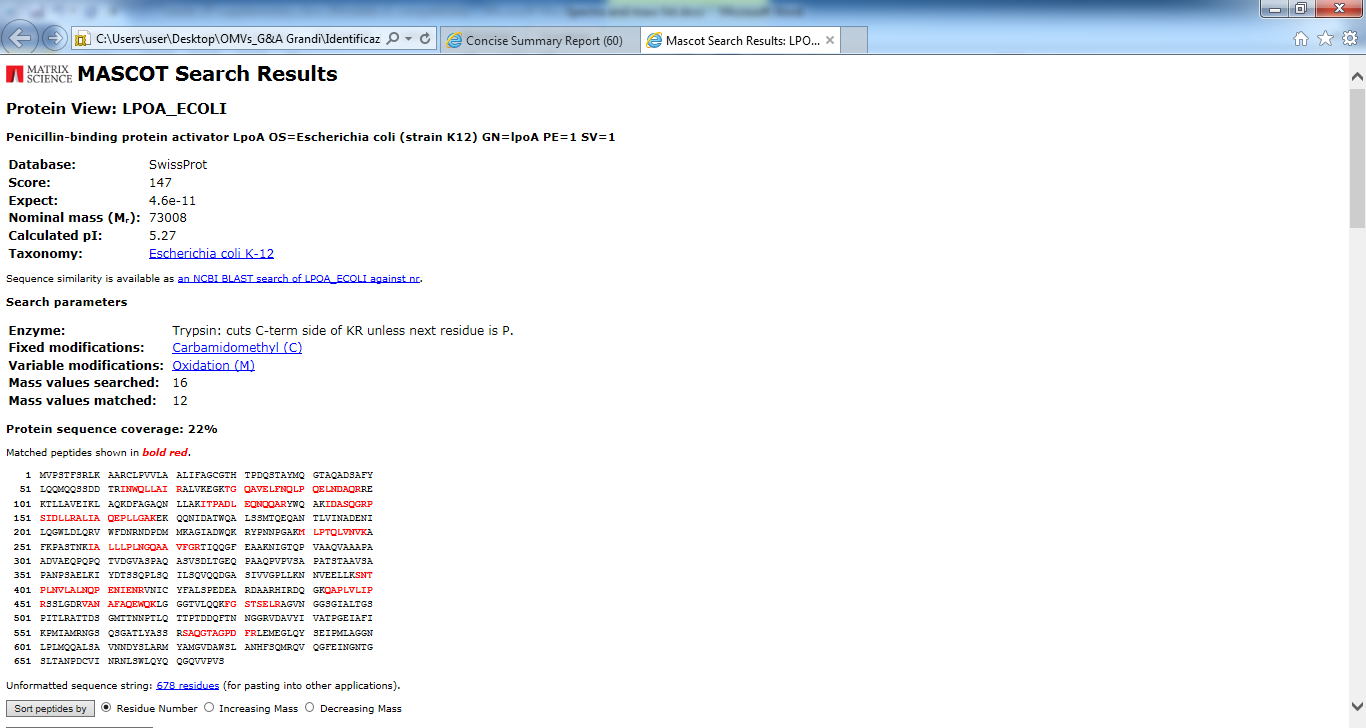

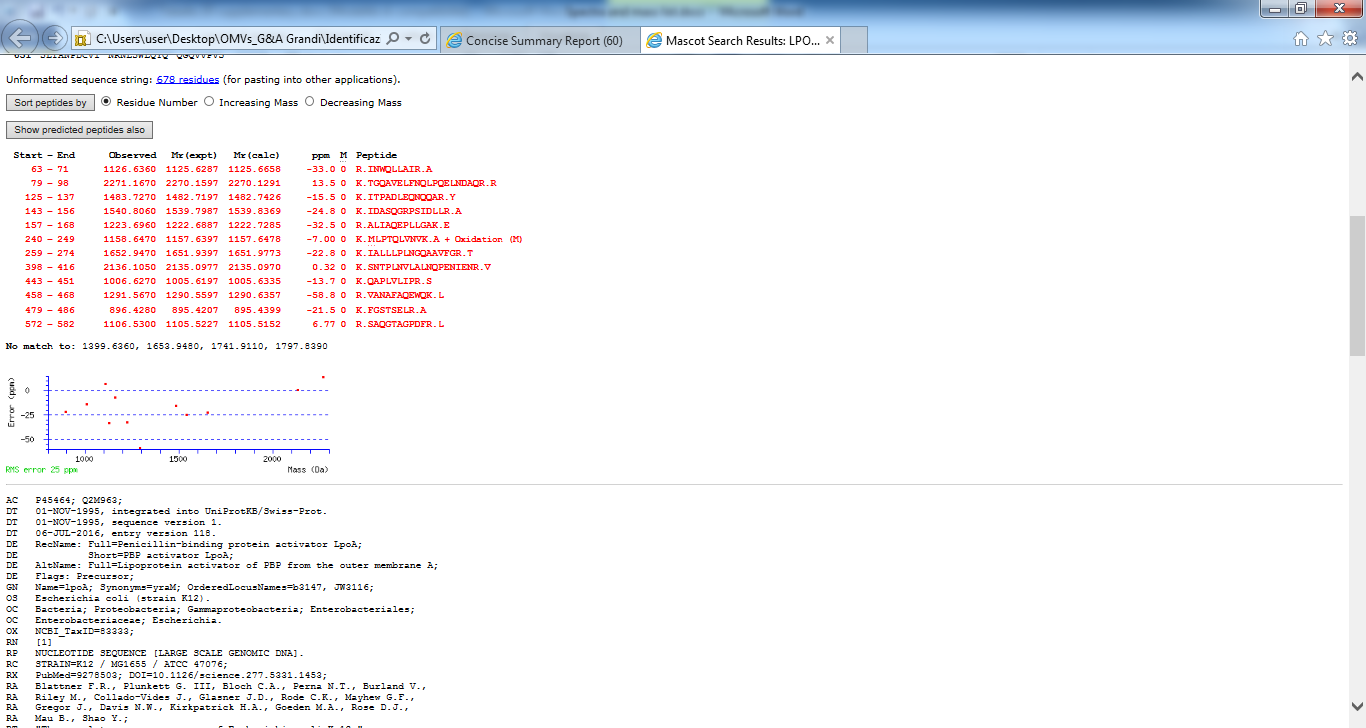


Spot N. 64 (LPOA_ECOLI)

m/z

1006.676

1126.706

1158.702

1483.809

1540.917

1653.021

1740.933

2136.073


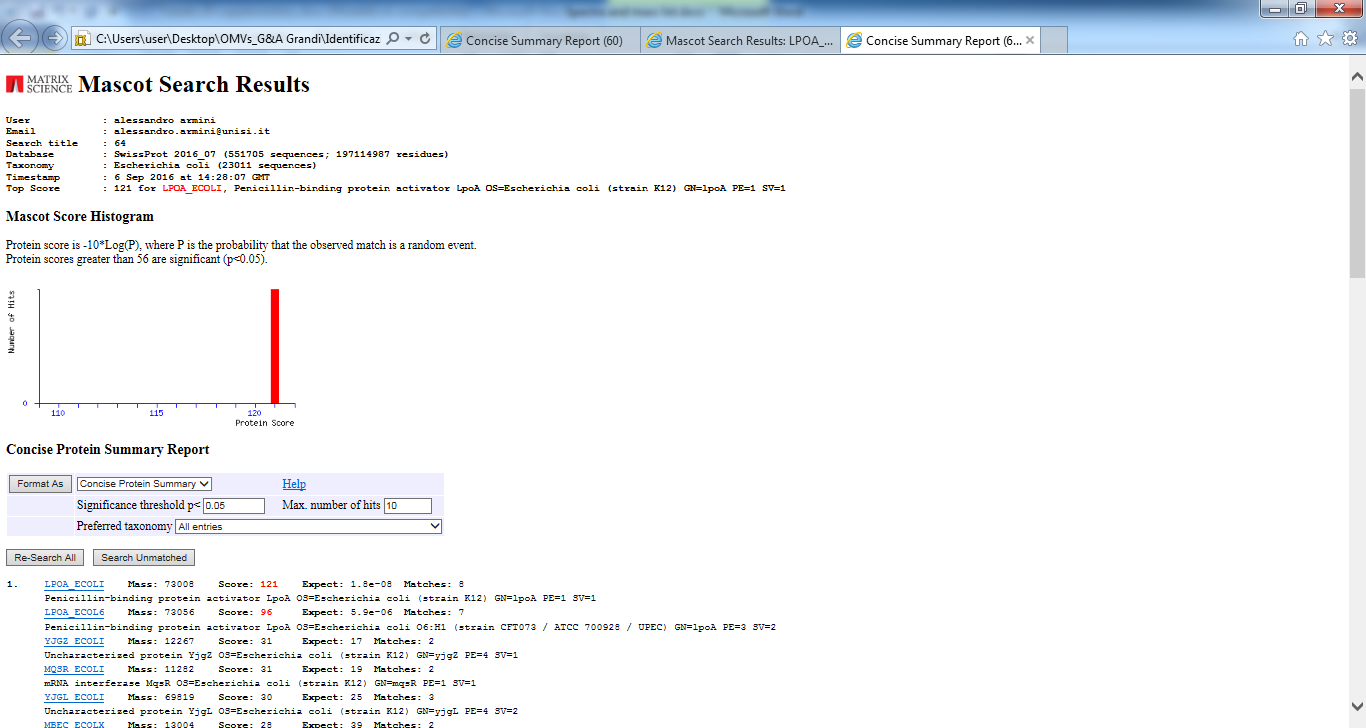


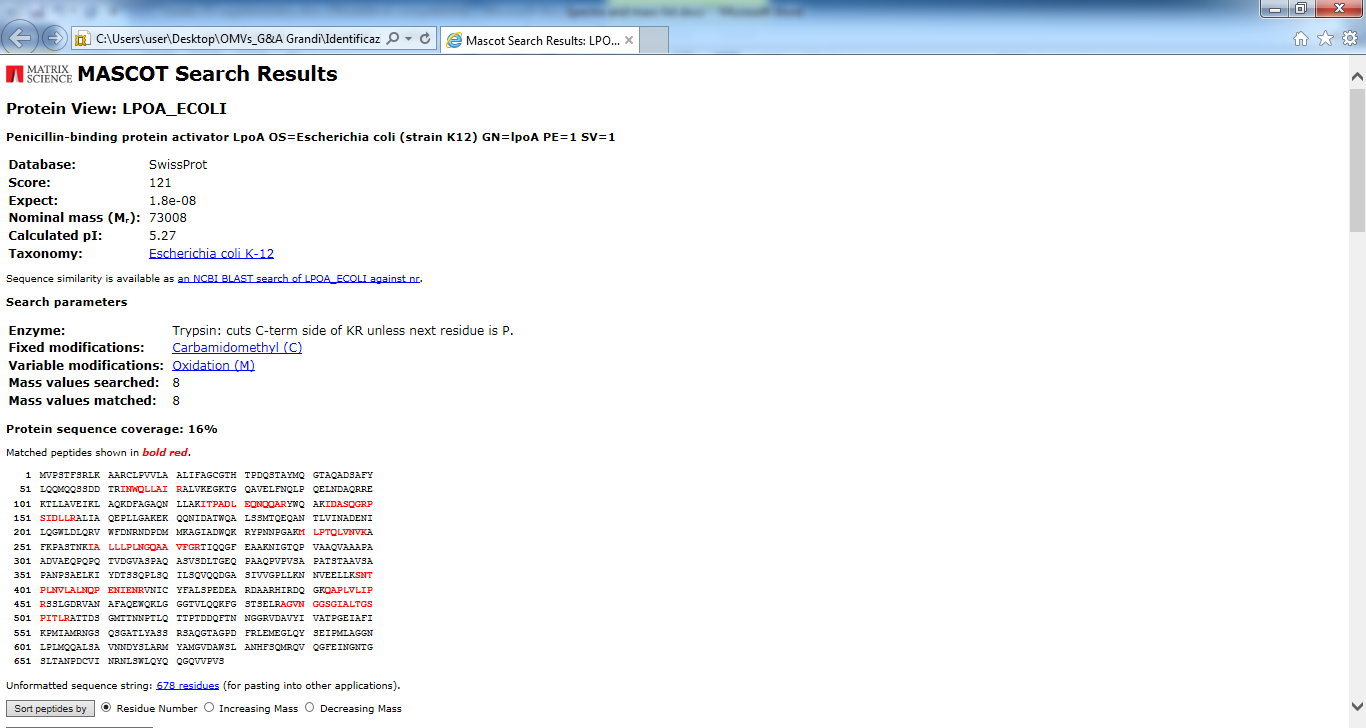

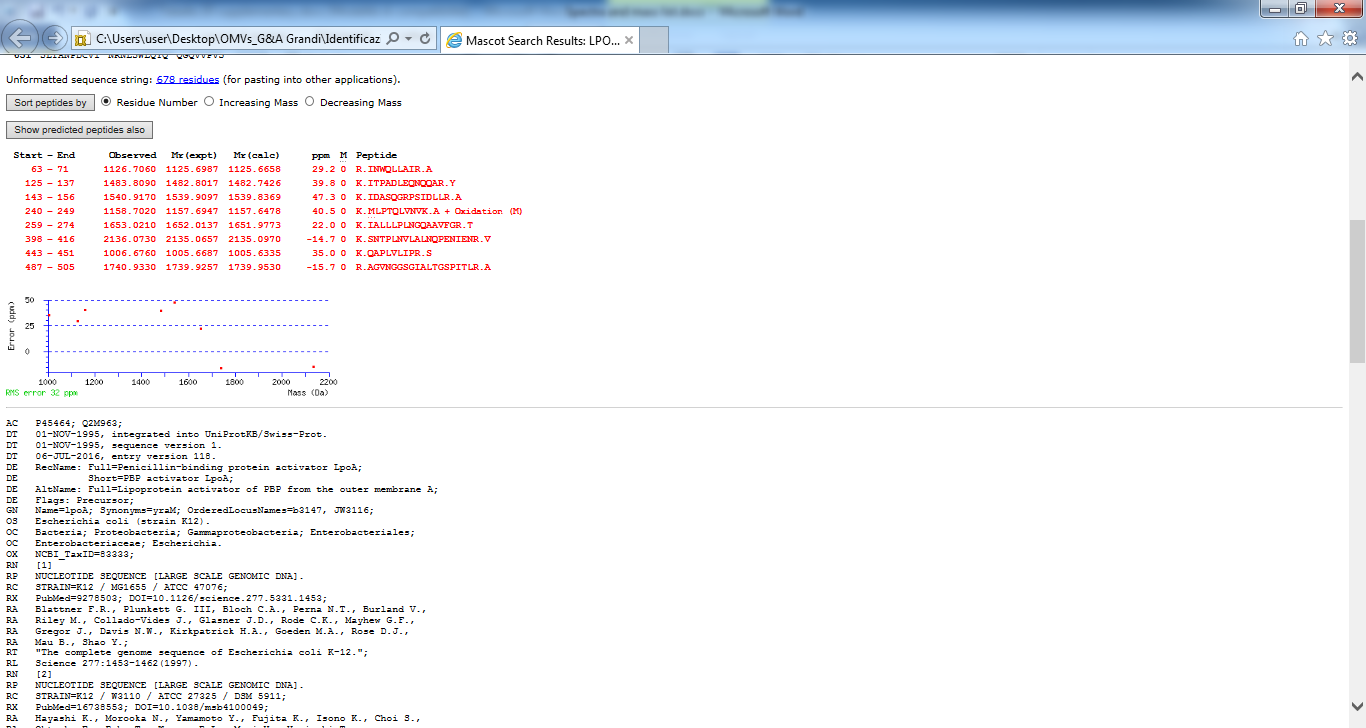


Spot N. 65 (CIRA_ECOLI)

m/z

1211.534

1316.724

1636.846

1729.820

1742.855

1759.676

1969.939

2372.081


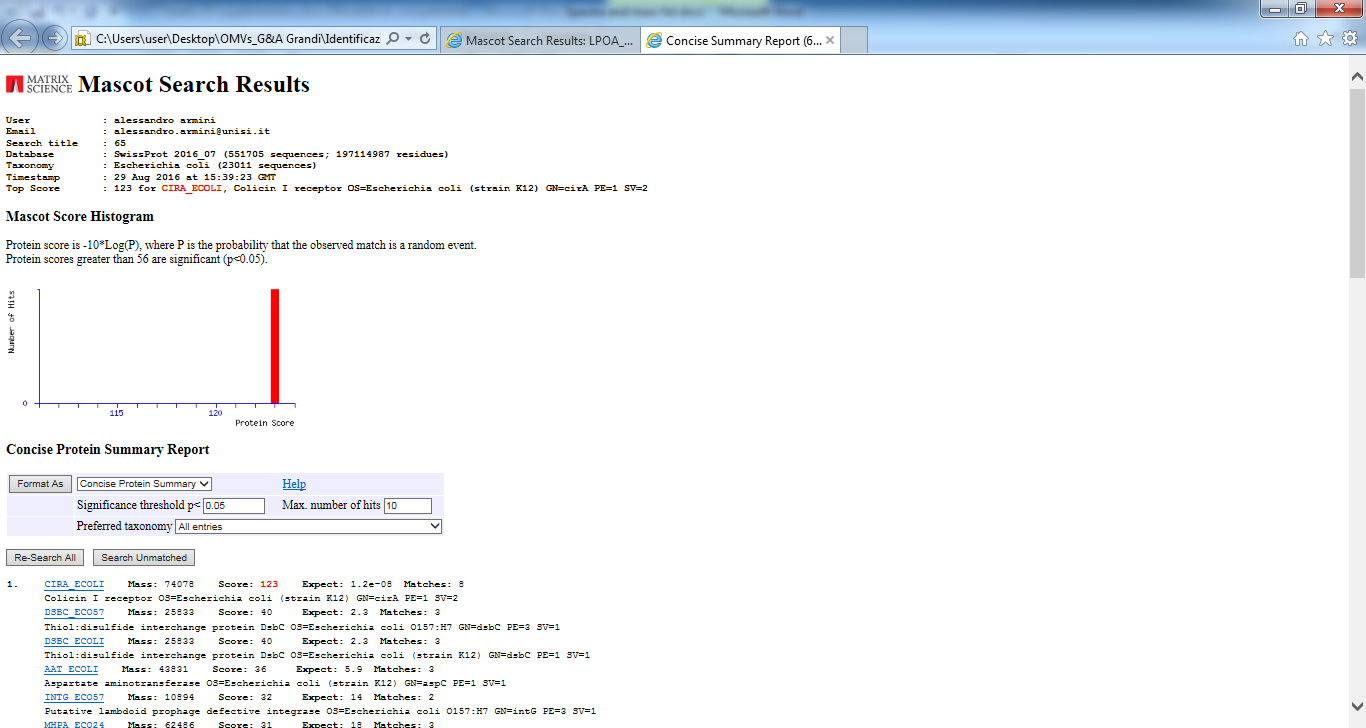


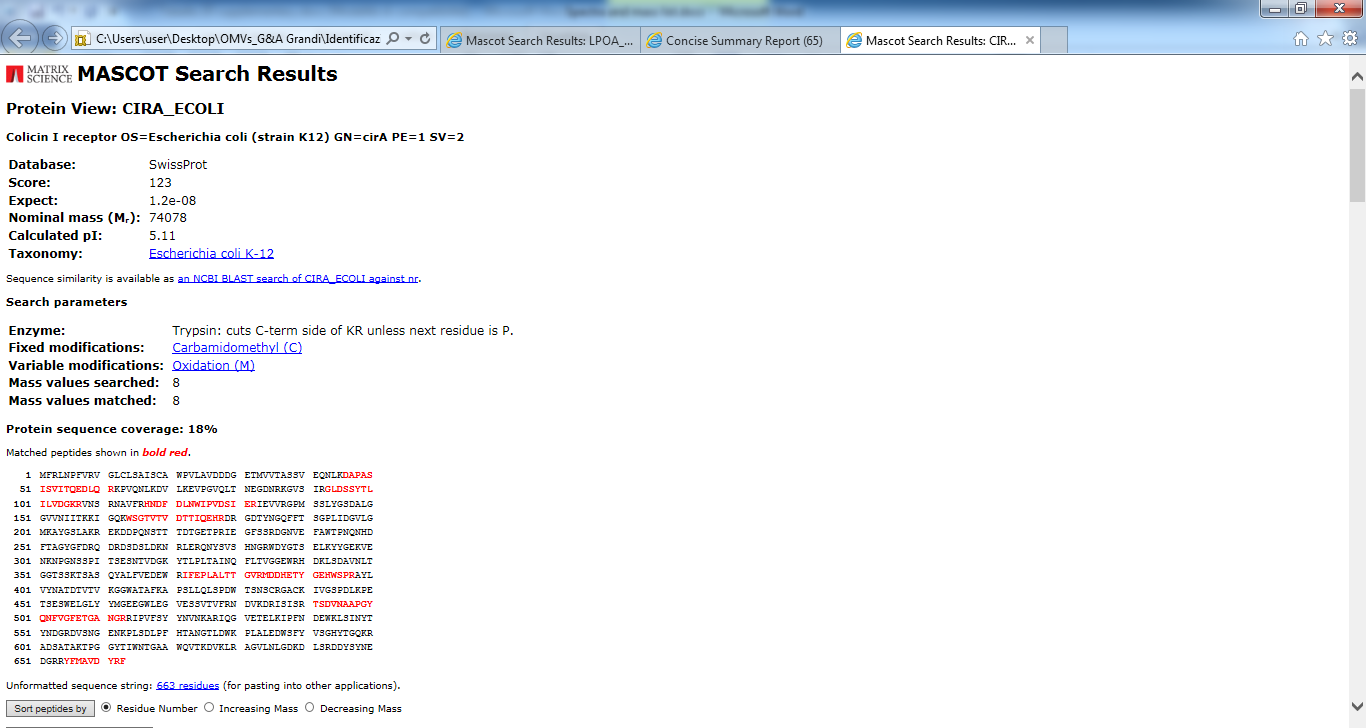

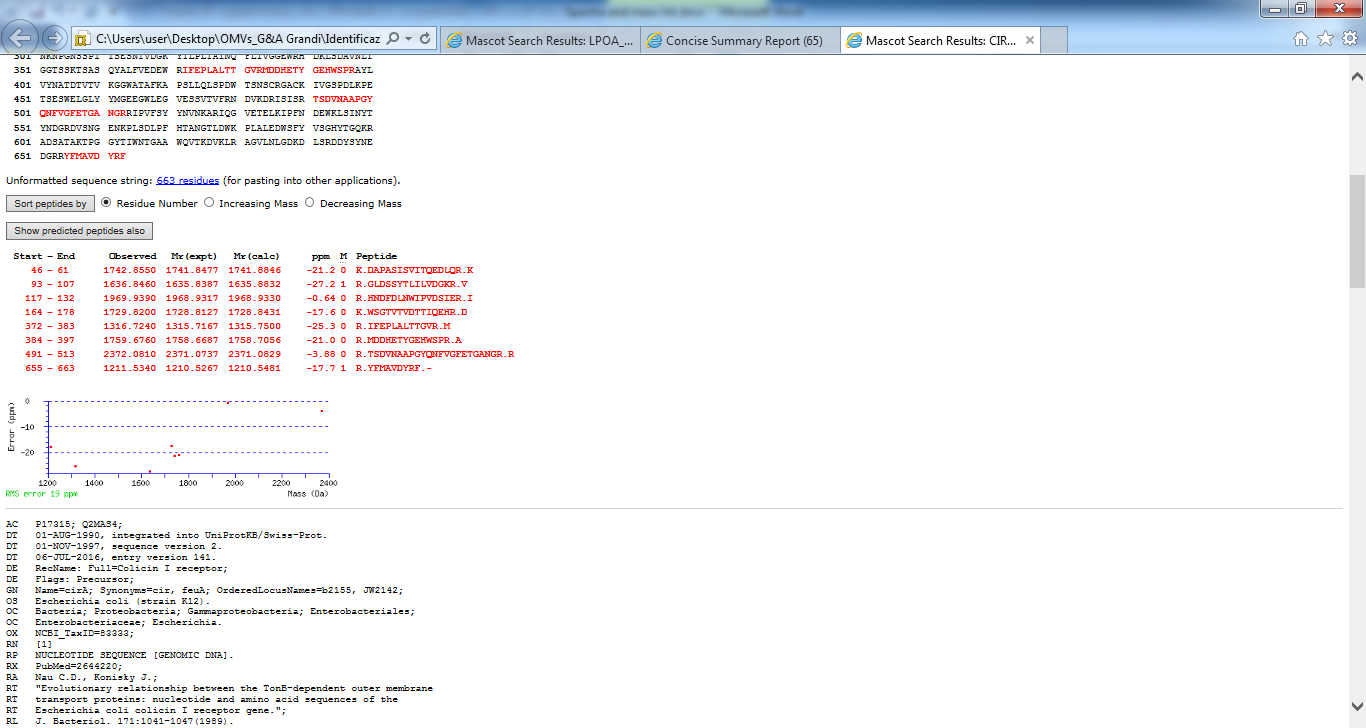


Spot N. 68 (BAMA_ECO24)

m/z

889.460

999.453

1114.550

1339.654

1520.740

1528.832

1844.953

2146.025


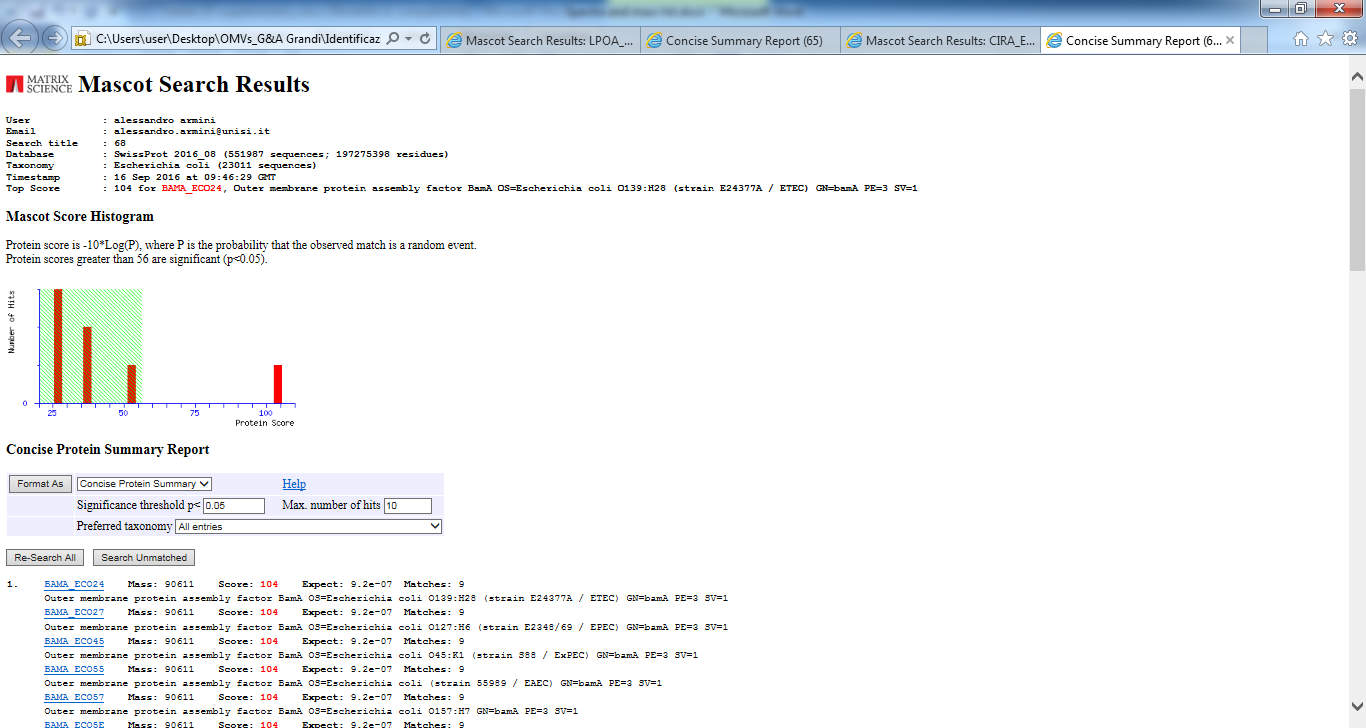


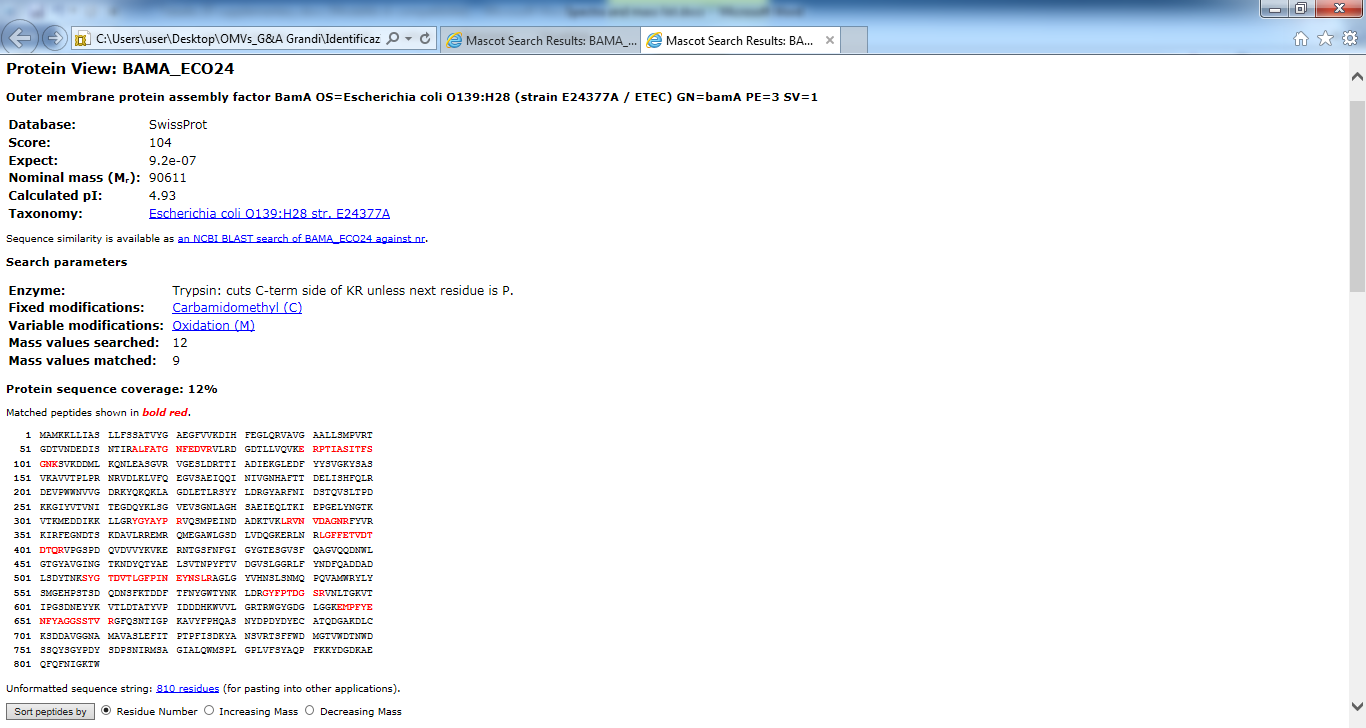

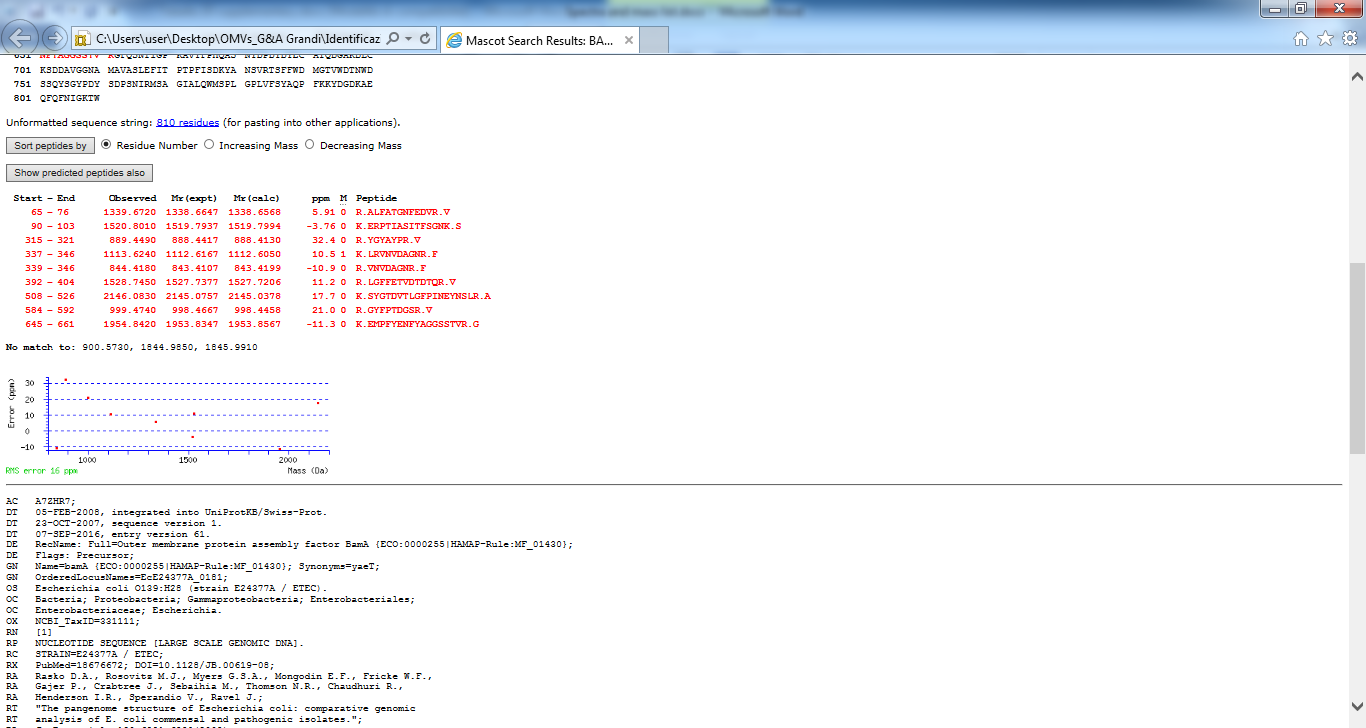


Spot N. 78 (AMY1_ECOLI)

m/z

826.429

864.454

892.422

942.395

1026.498

1310.571

1456.622

1575.622

1726.766

1836.808

1840.753

1852.794

2140.046

2255.100


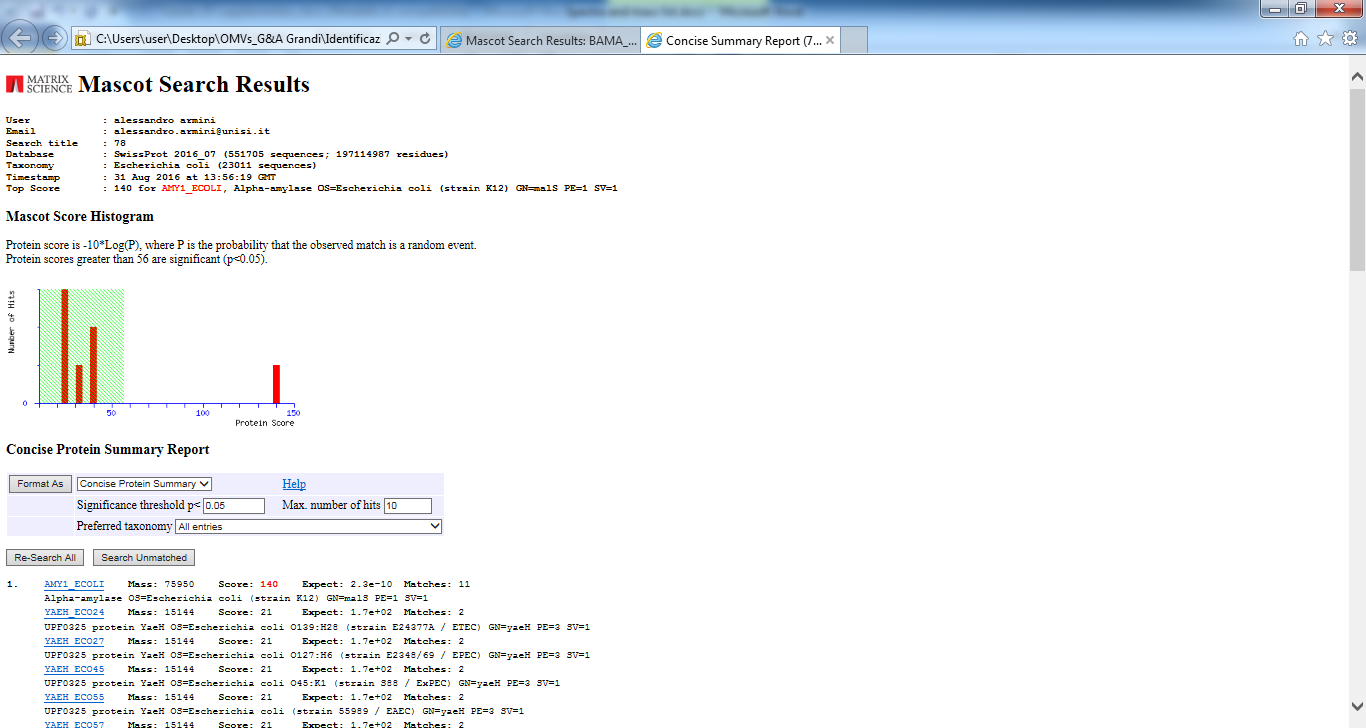


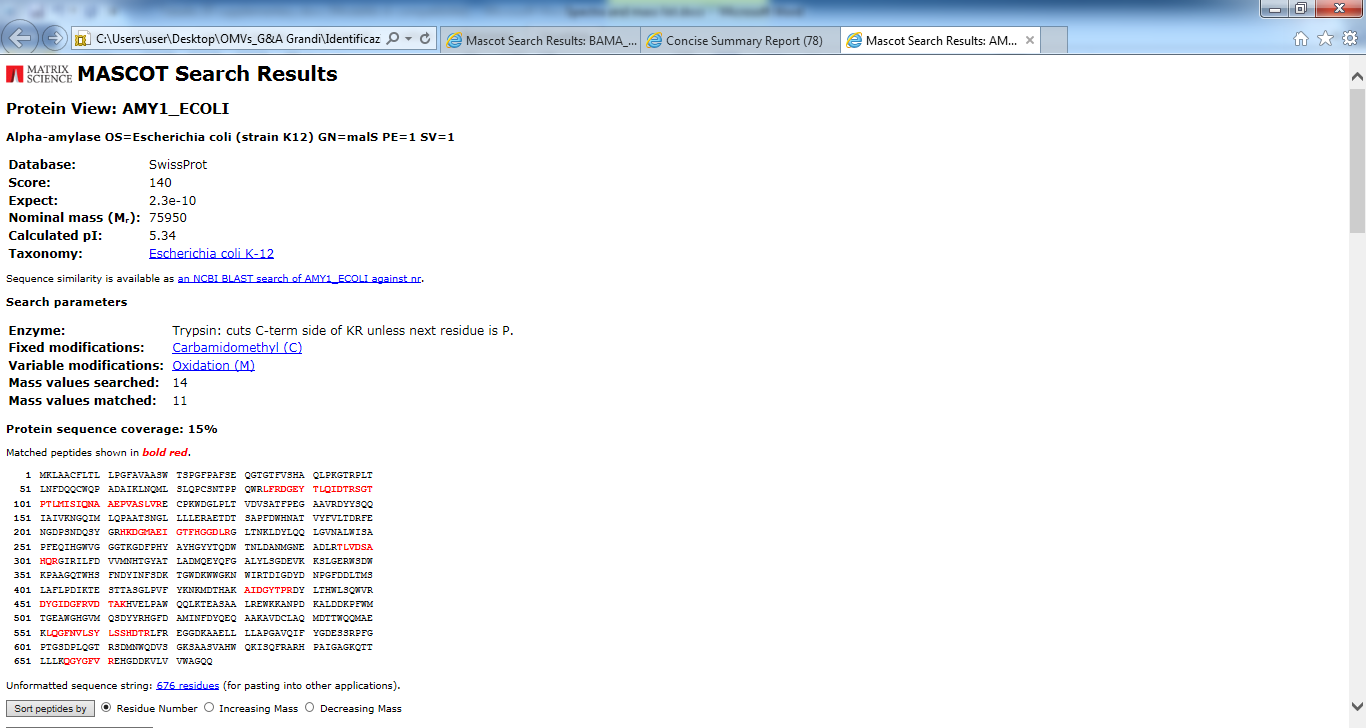

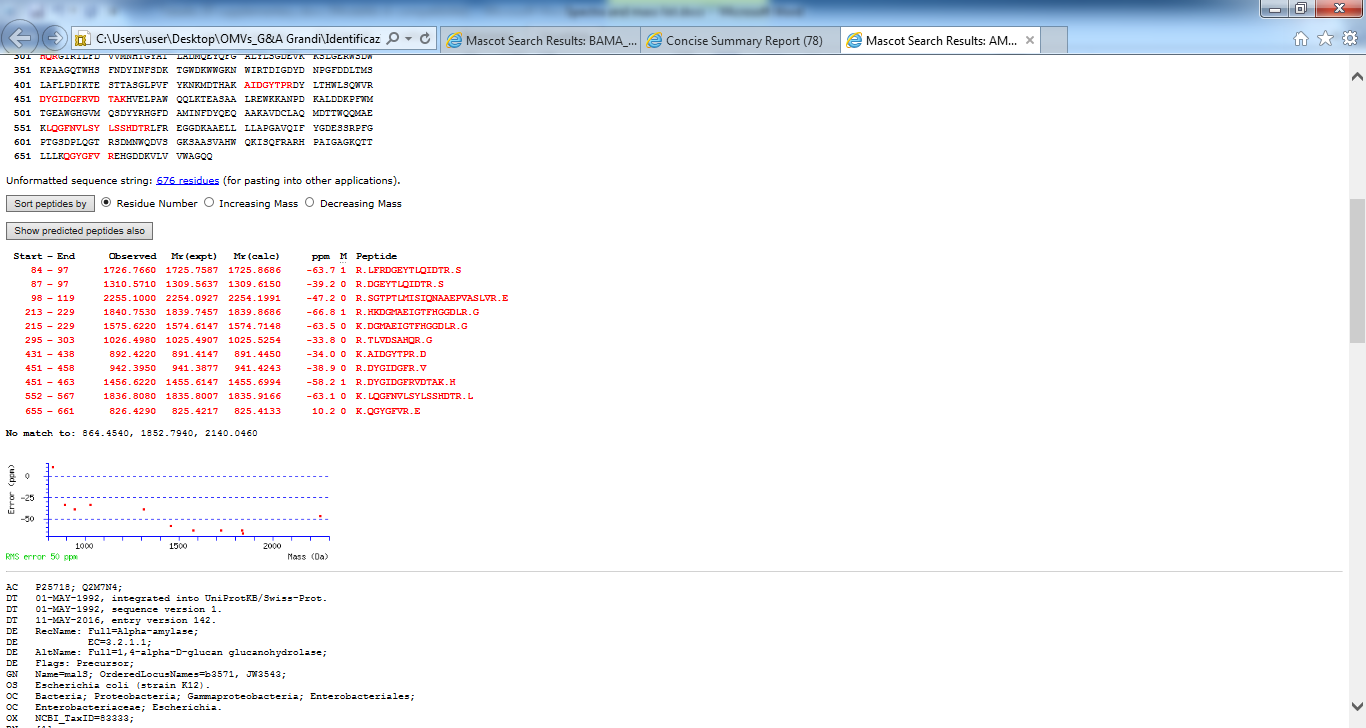


Spot N. 79 (CPDB_ECOLI)

m/z

1113.682

1246.619

1360.699

1378.695

1445.706

1450.639

1477.715

1488.776

1854.954

1891.866


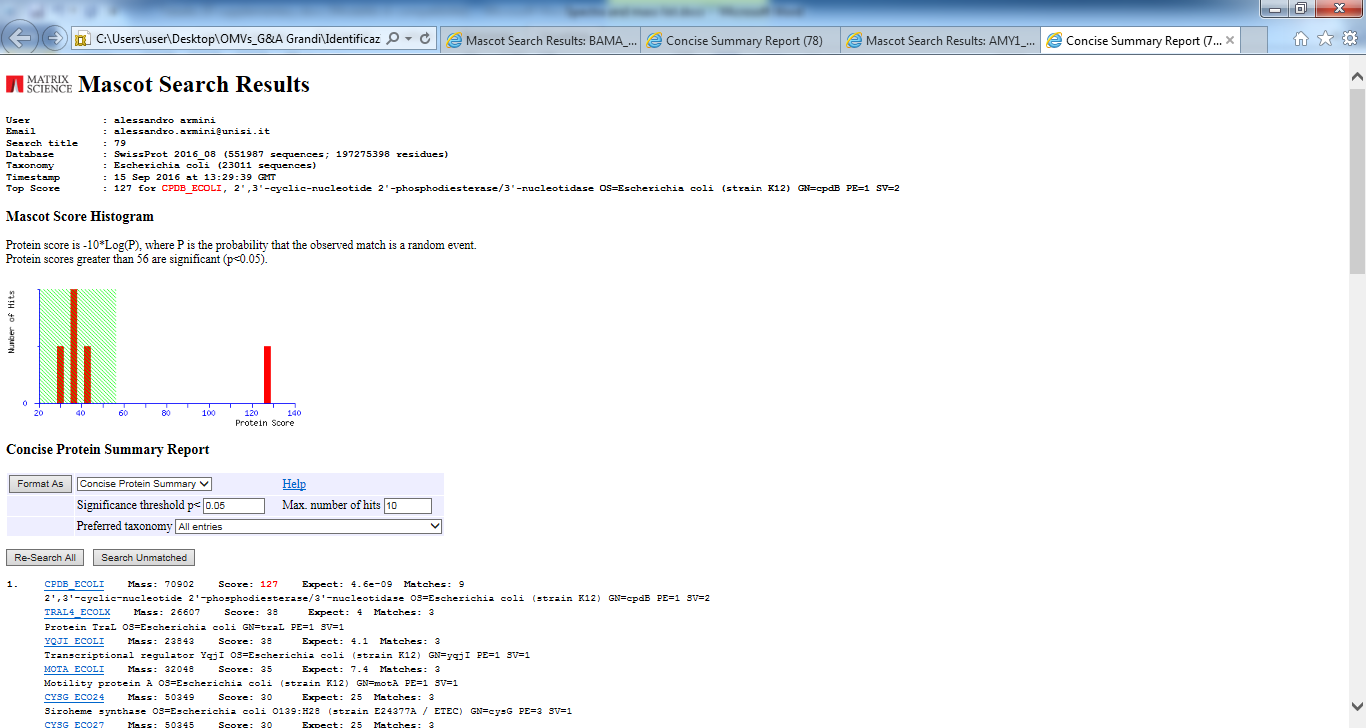


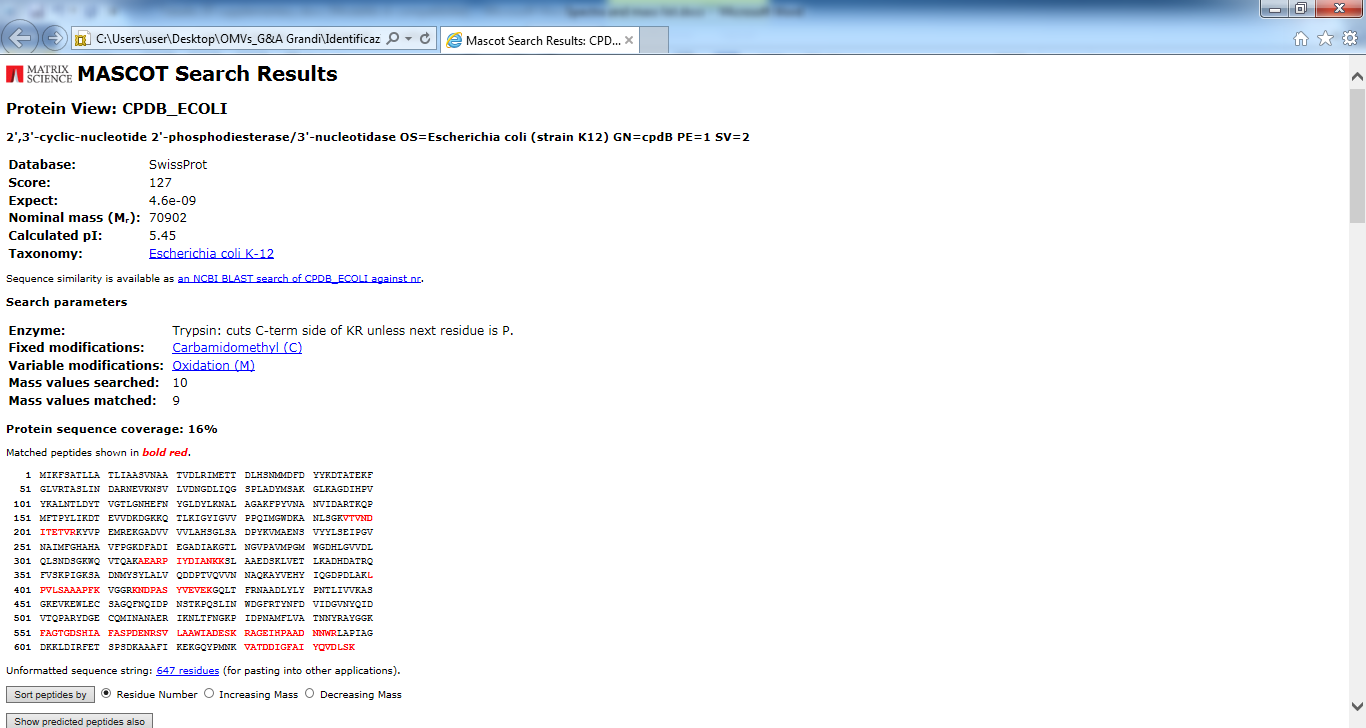

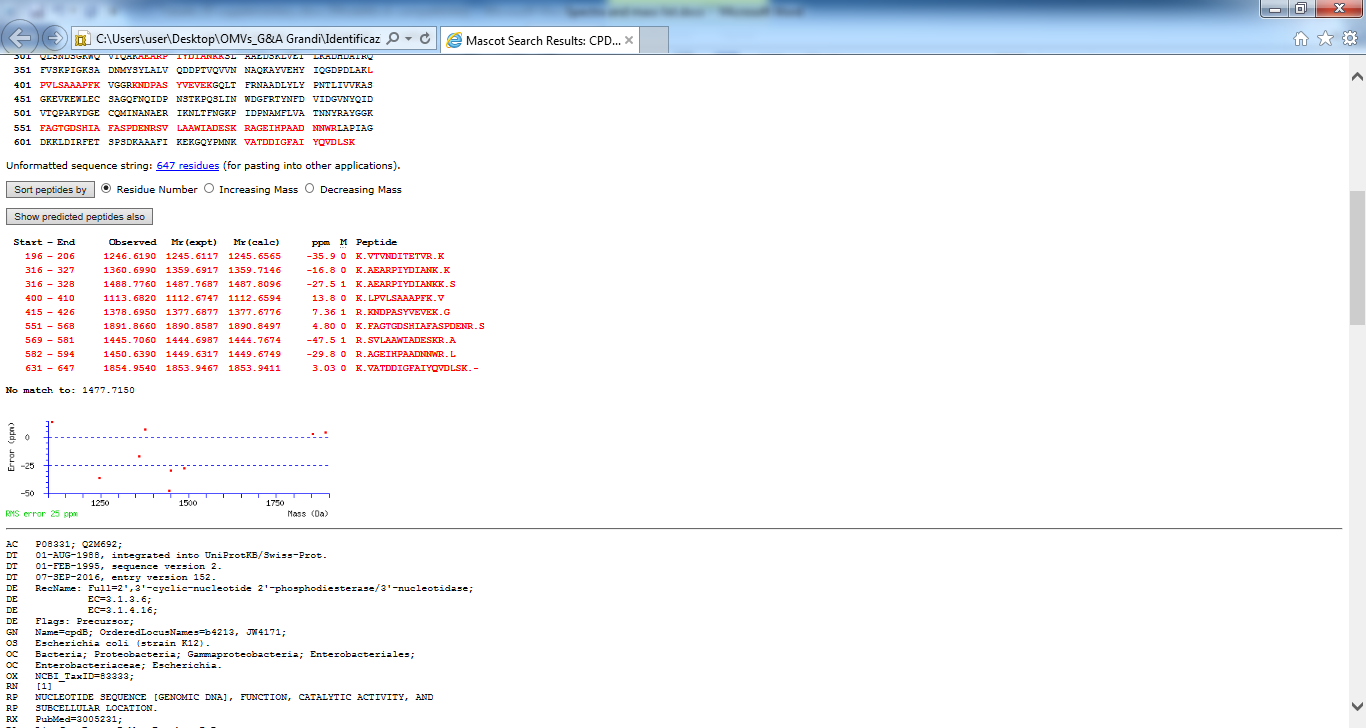


Spot N. 80 (CPDB_ECOLI)

m/z

922.504

960.506

1246.619

1360.625

1378.654

1430.710

1450.618

1468.759

1477.718

1488.760

1854.803

1891.853

2081.038

2093.045

2270.119

2428.023


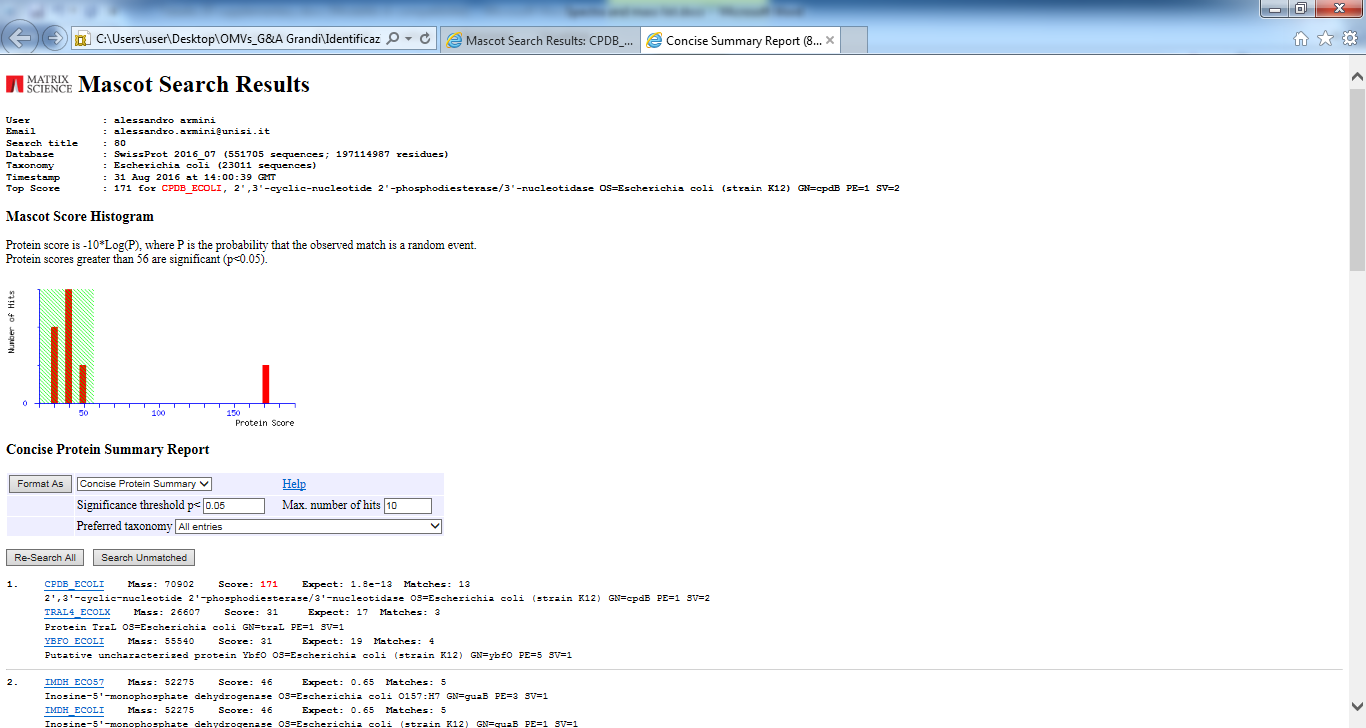


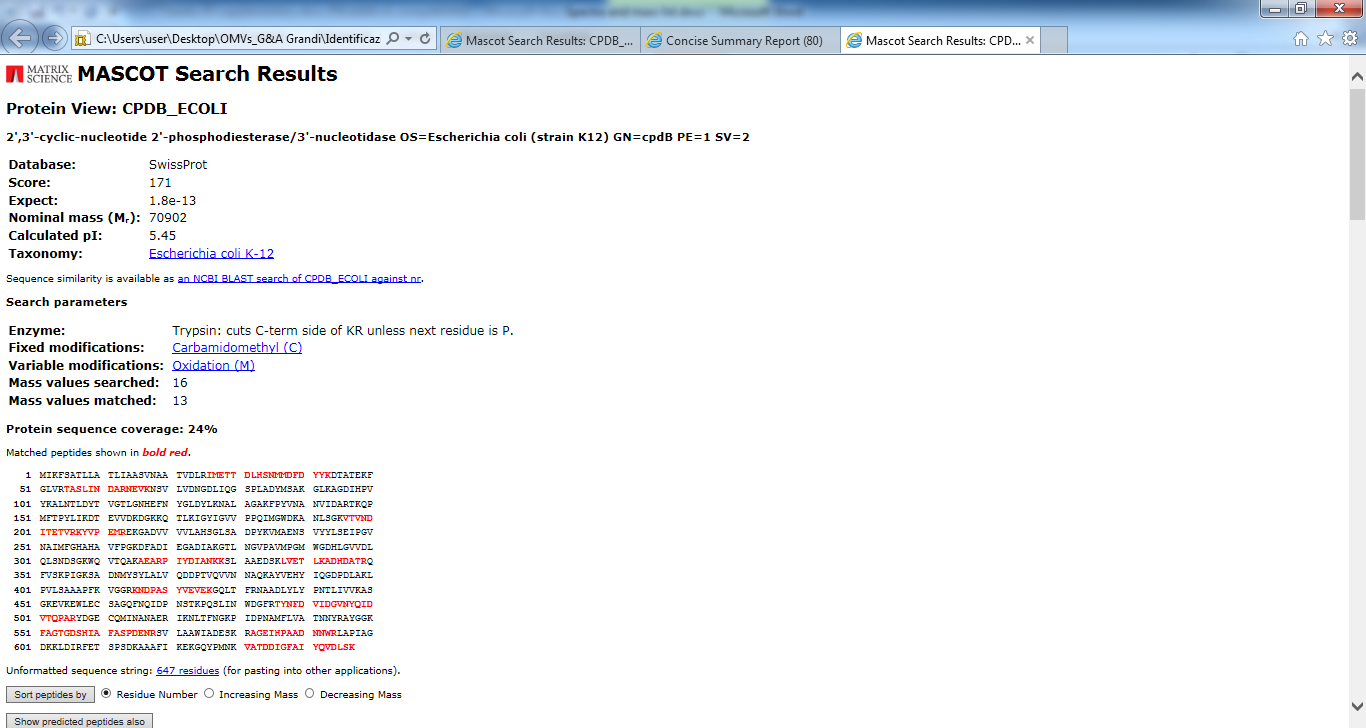

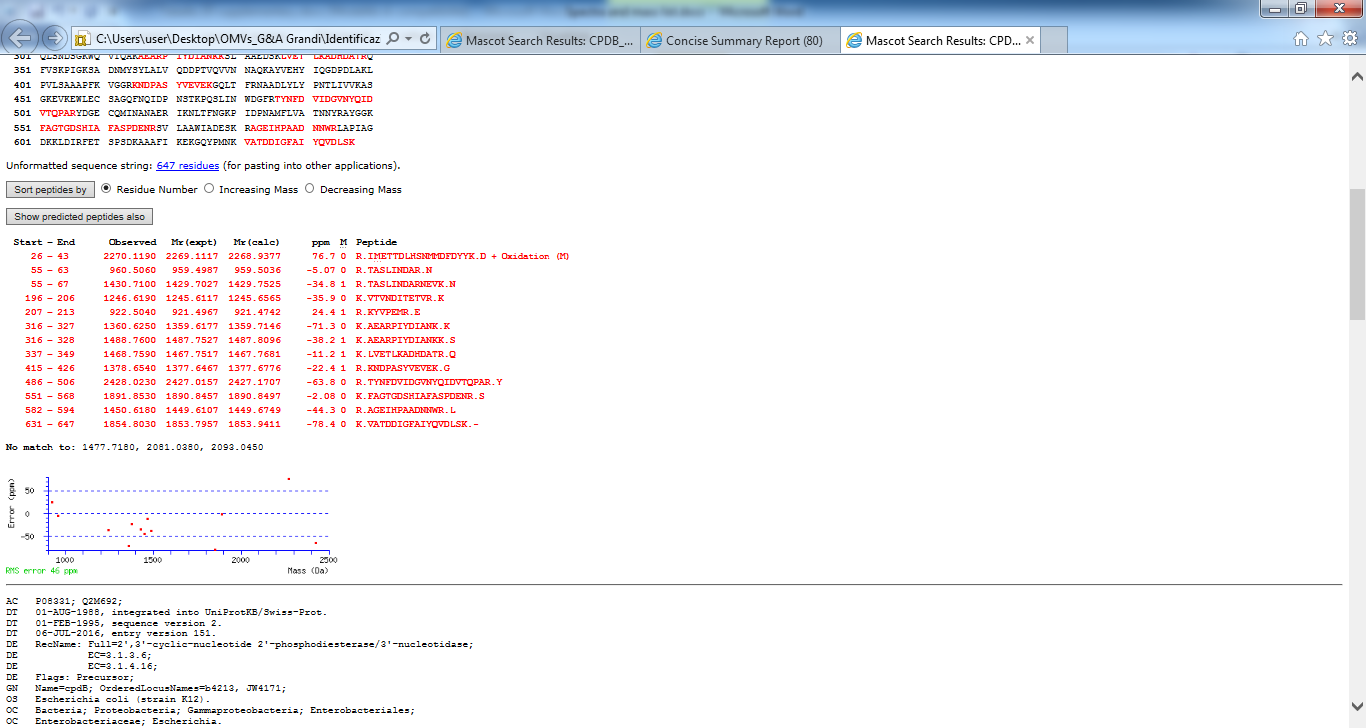


Spot N. 88 (SLT_ECO57)

m/z

789.364

930.539

936.489

961.486

962.513

1104.524

1131.576

1139.606

1155.576

1221.658

1247.624

1306.652

1310.635

1338.669

1349.694

1447.745

1525.684

1780.886

2162.055


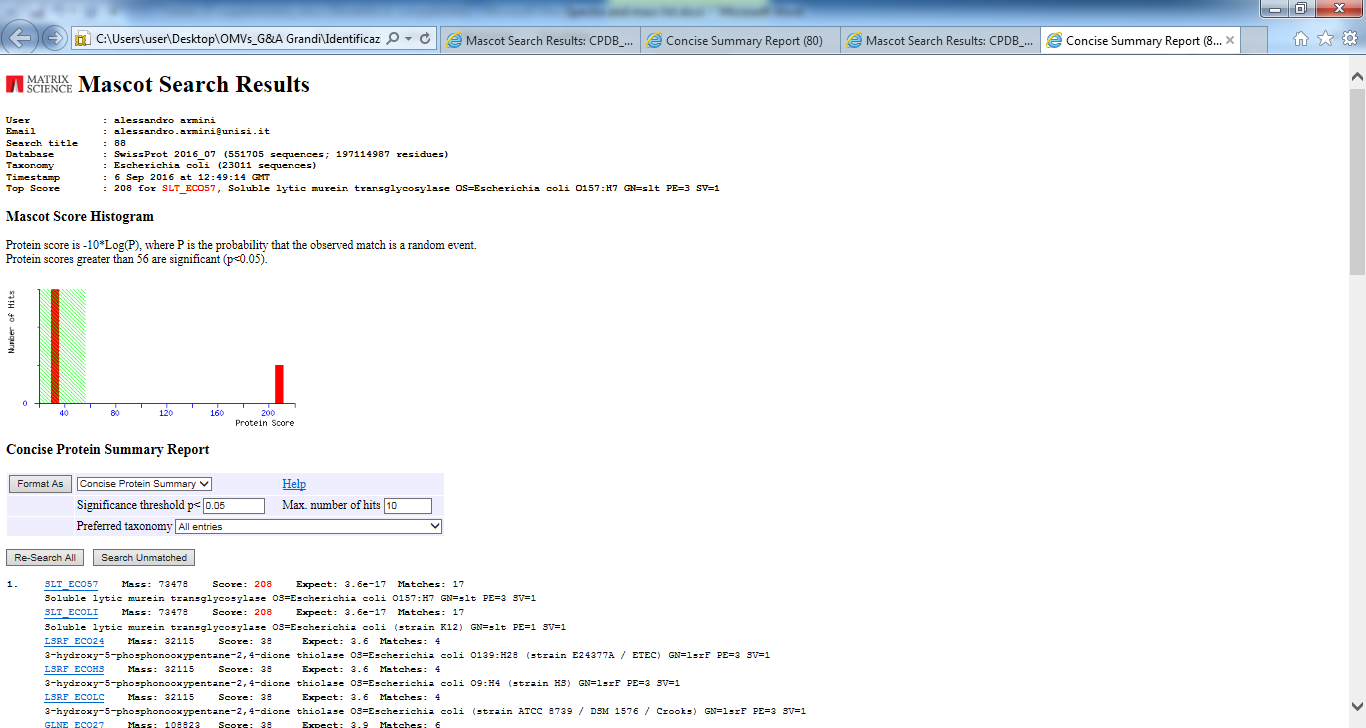


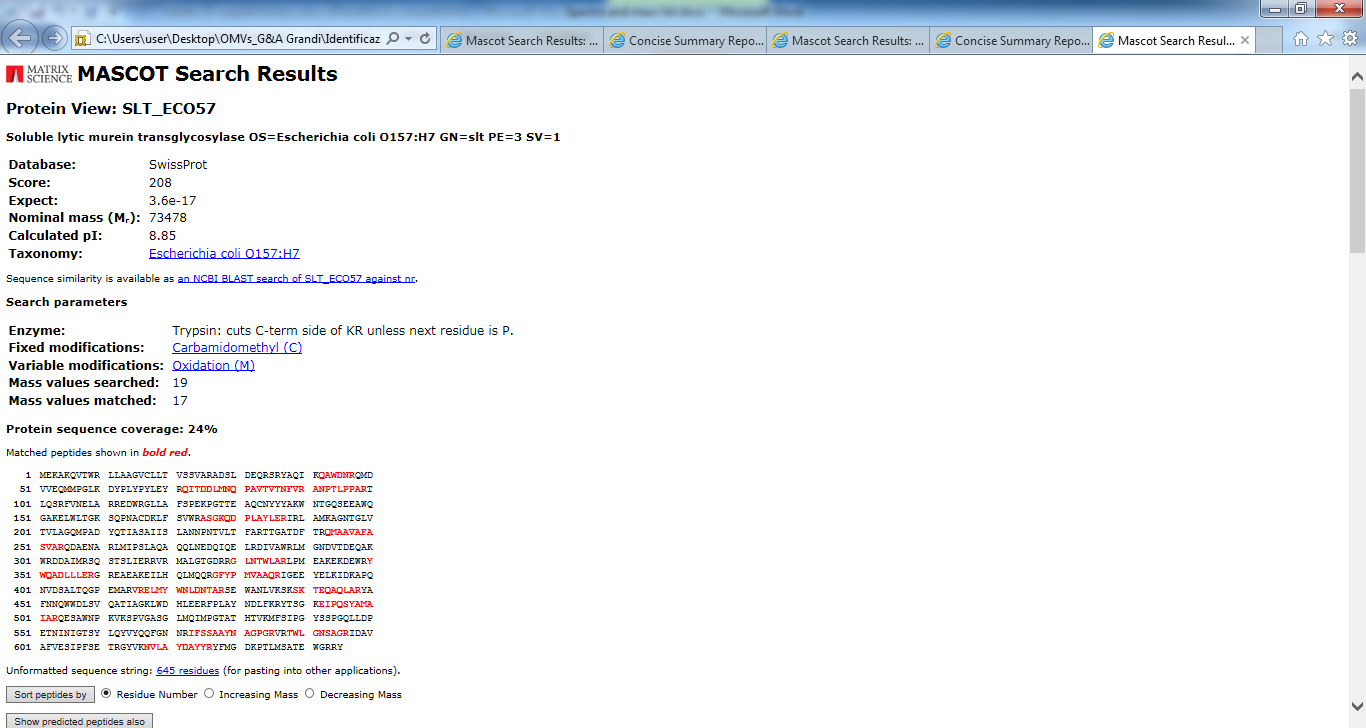

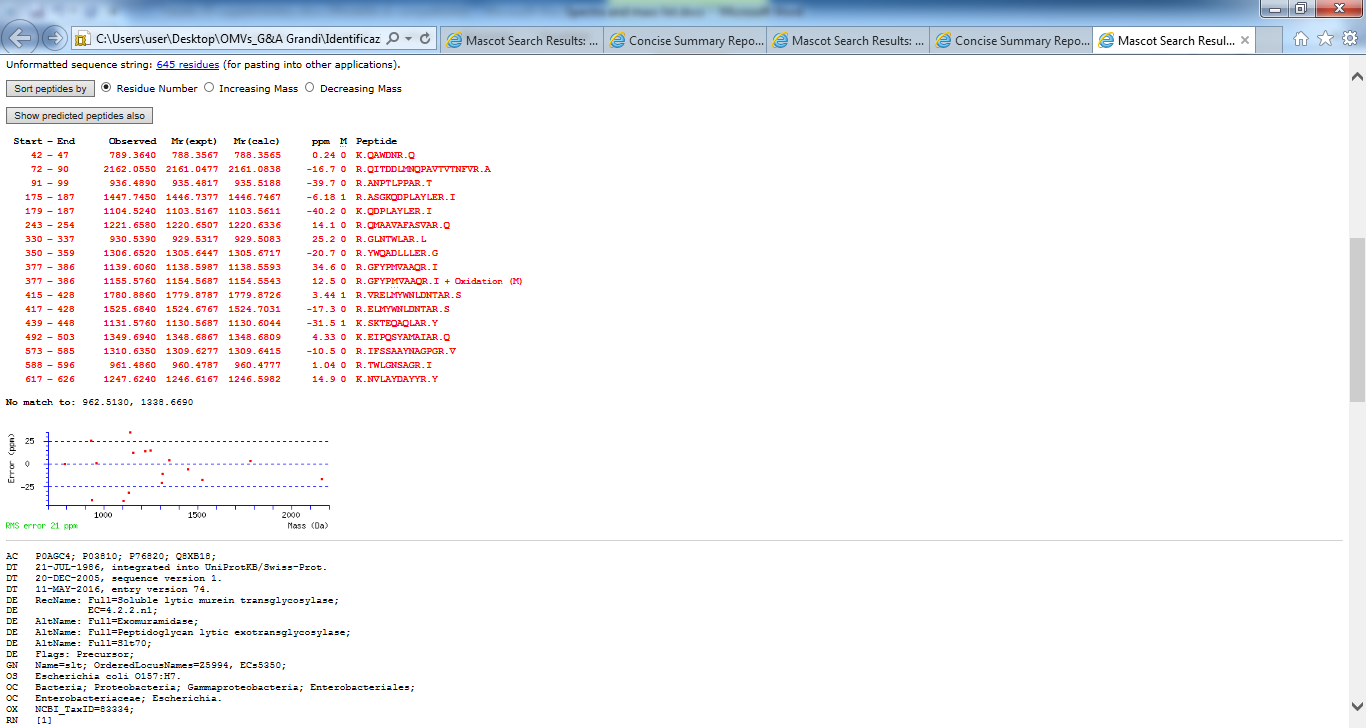


Spot N. 100 (USHA_ECOLI)

m/z

784.403

952.425

1030.487

1206.623

1293.707

1338.621

1600.702

1684.700

2340.214


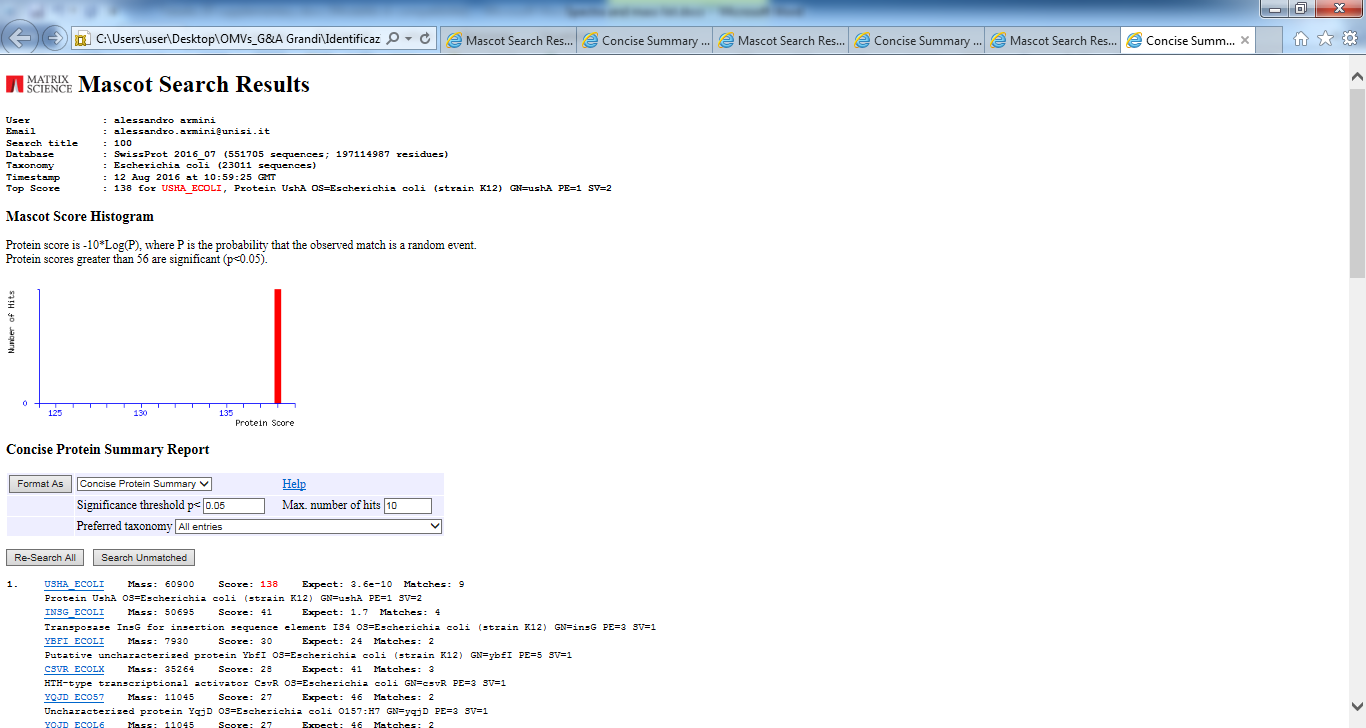


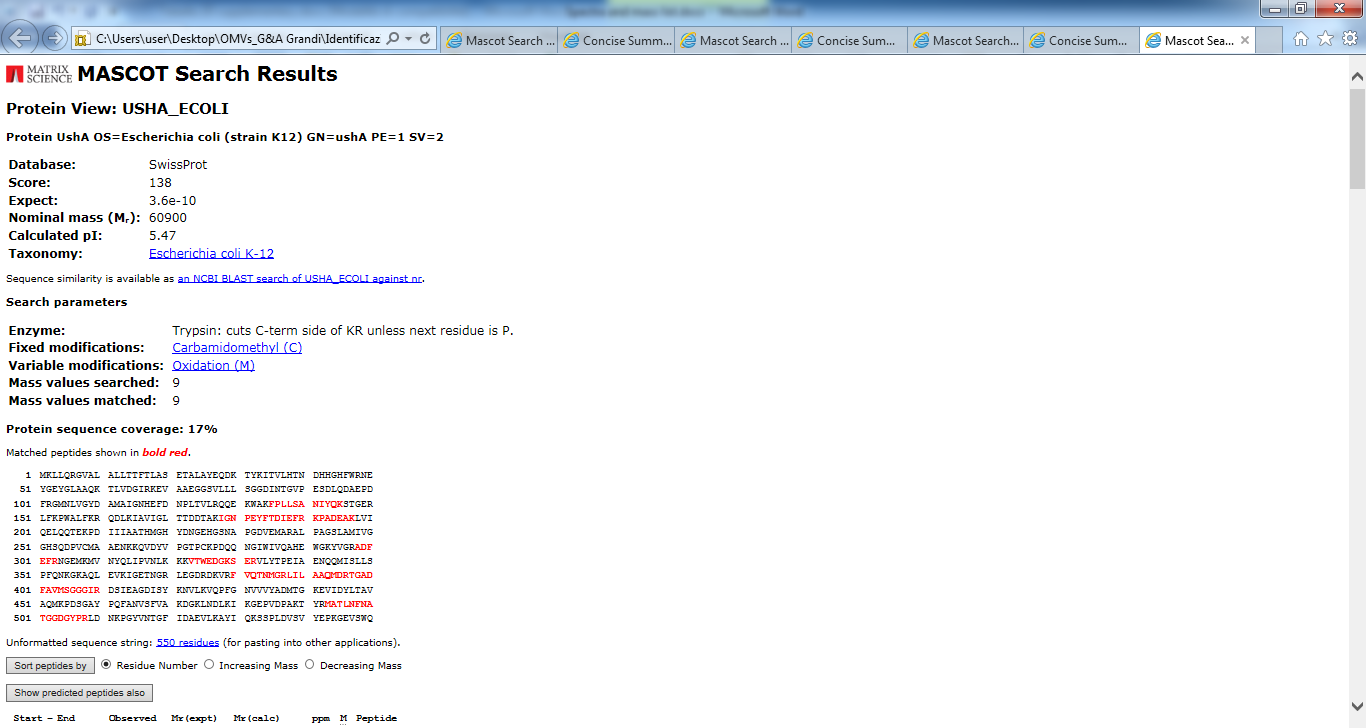

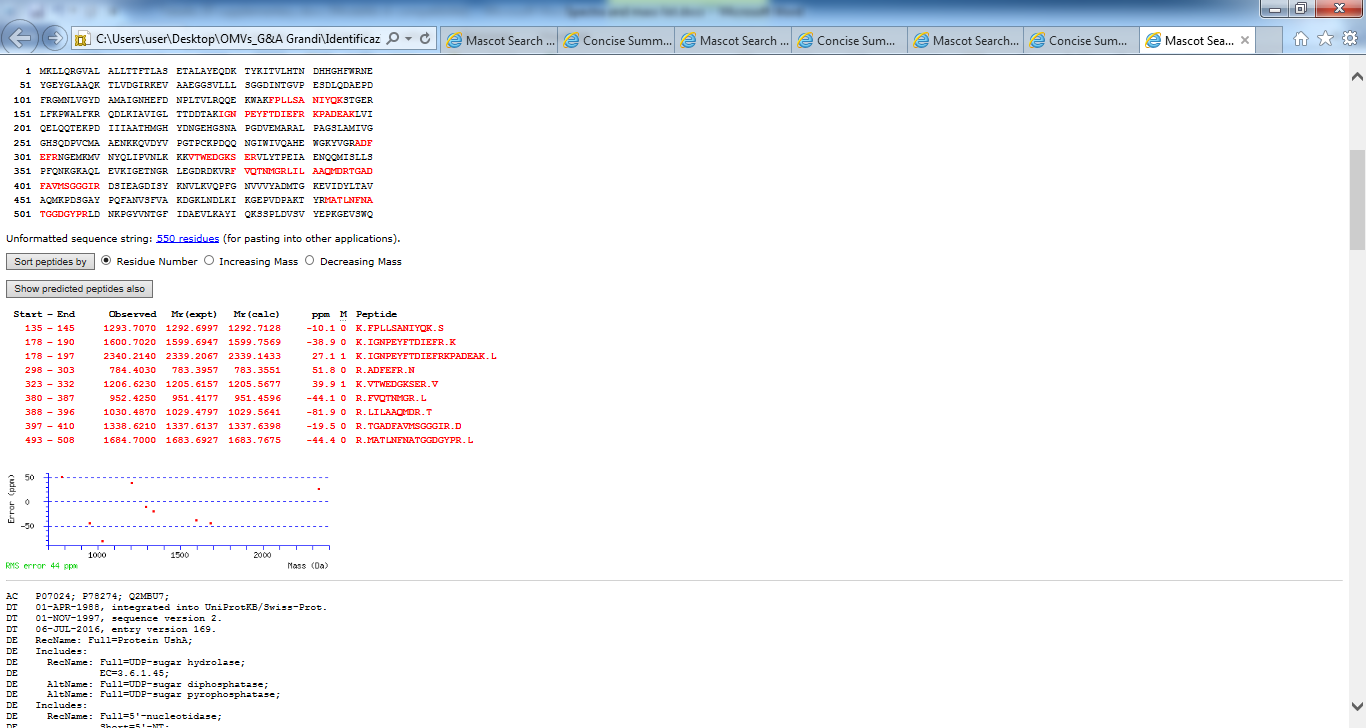


Spot N. 102 (TREA_ECO57)

m/z

788.431

1145.554

1241.543

1323.597

1417.690

1435.786

1515.718

1880.848


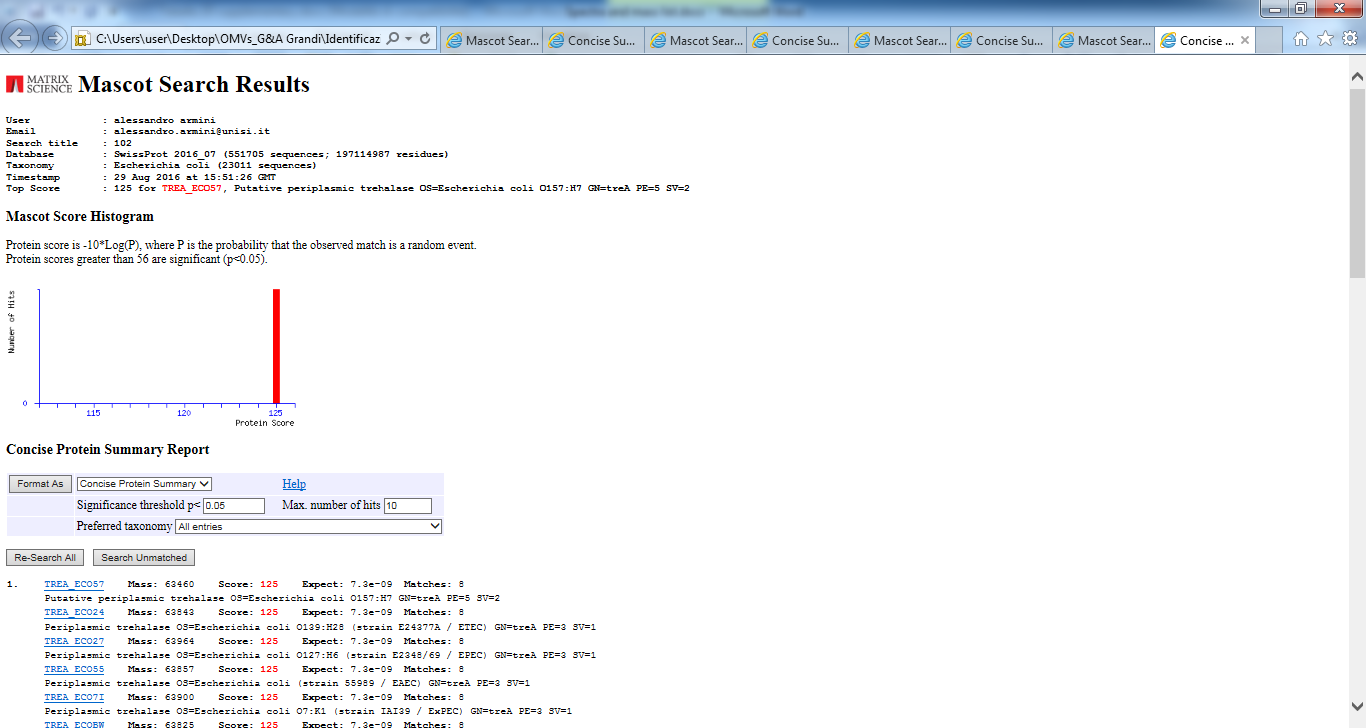


Spot N. 103 (YDEN_ECOLI)

m/z

1482.776

1518.750

1548.694

1600.785

1638.867

1684.804

1850.943

2385.095

2750.381

Spot N. 105 (YDEN_ECOLI)

m/z

818.428

1482.877

1518.882

1807.941

1851.012

2050.198

2247.016

2385.058

2750.198

Spot N. 109 (OPGD_ECO24)

m/z

1018.635

1030.686

1154.645

1167.651

1186.659

1203.610

1315.792

1323.779

1344.750

1360.762

1458.732

1516.862

1524.819

1674.938

1721.996

1796.993

2279.324

Spot N. 110 (OPPA_ECOLI)

m/z

916.492

917.473

958.511

990.498

1030.437

1042.493

1273.639

1274.699

1629.782

1767.860

2142.817

2232.080

2442.260

Spot N. 111 (OPPA_ECOLI)

m/z

752.446

818.446

917.525

958.587

990.579

1030.572

1042.579

1059.693

1080.595

1091.698

1094.589

1273.786

1274.855

1409.796

1629.903

1635.853

1767.955

1791.960

1823.949

2002.949

2130.024

2143.039

2175.034

2209.139

2232.259

2337.226

2380.292

2412.274

2442.310

2551.424

2589.346

2606.394

2607.372

2662.427

2693.389

Spot N. 114 (KPSD1_ECOLX)

m/z

919.520

1261.644

1290.698

1672.868

1777.009

1844.946

2756.336

Spot N. 115 (OPGG_ECO24)

m/z

907.566

940.595

1075.630

1254.793

1326.715

1482.799

1545.860

1562.859

1577.853

1638.958

1759.031

1761.008

1852.023

1917.114

2129.104

2323.175

Spot N. 116 (OPGG_ECO24)

m/z

919.553

1154.679

1261.798

1273.816

1290.802

1535.862

1545.900

1672.941

1777.014

1793.003

1805.996

1821.047

1844.970

1853.033

1860.984

1948.095

2023.046

2218.191

2323.220

2503.303

2628.384

2637.440

2756.526

2891.571

Spot N. 120 (GLPQ_ECOLI)

m/z

1083.558

1290.566

1380.656

1381.649

1440.671

1490.785

1491.763

1599.650

1638.708

1679.814

1723.709

2301.158

2806.382

Spot N. 137 (DEGP_ECO57)

m/z

785.509

1339.737

1475.748

1496.733

1652.946

1817.939

2017.111

2240.106

Spot N. 138 (DEGP_ECO57)

m/z

785.521

878.412

1339.761

1440.818

1475.784

1496.739

1652.947

1781.033

1817.933

1833.944

2017.103

2029.095

2048.072

2087.091

2103.080

2192.091

2223.105

2225.395

2240.092

2252.115

2750.574

Spot N. 146 (TOLC_ECOLI)

m/z

788.484

801.455

802.484

828.453

914.528

989.470

1285.538

1340.734

1361.560

1379.548

1391.570

1395.662

1550.644

1562.646

1616.743

1737.812

1763.751

1829.798

2017.860

2034.899

2046.910

2185.075

2227.090

2750.435

Spot N. 147 (TOLC_ECOLI)

m/z

914.542

1285.675

1550.784

1616.874

2034.973

2185.067

Spot N. 150 (TOLC_ECOLI)

m/z

788.473

914.544

989.553

1285.634

1328.719

1345.613

1346.593

1361.634

1379.650

1391.661

1395.680

1550.759

1562.761

1616.856

1763.852

2034.975

2185.140

2227.100

Spot N. 156 (YHJJ_ECOLI)

m/z

733.348

811.468

1052.431

1066.460

1119.469

1171.521

1377.650

1471.659

1544.666

1577.725

1859.839

2020.945

2035.941

2043.035

2429.364

2568.539

Spot N. 157 (DEGQ_ECOLI)

m/z

890.576

939.649

962.563

974.573

1105.736

1195.631

1207.621

1211.630

1405.725

1443.810

1576.025

1605.828

1631.925

1698.916

1925.124

2009.171

2116.276

2117.322

2155.285

2191.283

Spot N. 158 (FTSP_ECOLI)

m/z

765.459

819.479

959.463

960.448

991.450

1126.564

1159.537

1176.524

1191.506

1346.575

1347.552

1397.677

1403.635

1413.726

1588.670

1729.822

1756.737

1769.845

1945.850

1948.859

1987.879

2083.929

Spot N. 164 (LAMB_ECO24)

m/z

964.415

1021.538

1294.606

1295.564

1408.578

1440.523

1563.726

1580.557

1731.780

1745.651

1784.972

1891.800

2132.991

2148.979

2356.151

2372.115

3217.481

Spot N. 165 (LAMB_ECOBW)

m/z

964.471

1021.526

1294.605

1295.589

1408.672

1525.809

1563.722

1564.720

1580.701

1638.839

1661.743

1731.860

1745.810

1759.816

1763.841

1859.925

1891.915

1906.804

1923.914

2133.067

2149.064

2317.047

2356.135

2372.117

2465.103

3217.464

Spot N. 166 (LAMB_ECOBW)

m/z

859.452

964.444

976.441

1021.488

1033.489

1294.572

1295.546

1408.630

1426.609

1524.780

1563.689

1564.678

1579.717

1661.730

1731.820

1745.770

1759.790

1763.815

1859.895

1891.889

1906.819

1923.885

2093.995

2133.060

2149.067

2297.241

2317.030

2329.219

2356.141

2372.138

2386.144

2388.142

2465.130

2954.606

3042.461

3074.716

3217.880

Spot N. 167 (LAMB_ECOBW)

m/z

917.482

949.475

964.478

976.483

1294.710

1295.678

1408.781

1426.748

1436.841

1440.778

1524.883

1525.891

1563.848

1564.832

1565.847

1579.859

1661.885

1684.989

1731.970

1745.911

1759.928

1763.983

1860.022

1892.017

1906.894

1923.999

2094.017

2133.127

2149.117

2297.190

2316.972

2356.099

2372.091

2386.087

2388.093

2465.006

2954.144

3042.124

3074.097

3217.214

Spot N. 168 (LAMB_ECOBW)

m/z

859.483

964.465

976.468

1021.512

1294.604

1295.572

1408.654

1426.624

1524.793

1525.783

1563.719

1564.721

1580.723

1661.755

1731.849

1745.797

1759.811

1763.847

1859.915

1891.912

1906.810

1923.909

2094.001

2133.080

2149.084

2297.238

2317.023

2329.243

2356.143

2372.150

2386.154

2388.149

2465.140

2953.616

3042.304

3074.470

3217.553

Spot N. 169 (LAMB_ECO24)

m/z

964.535

1030.641

1276.776

1294.686

1408.722

1563.818

1666.959

1731.944

2133.133

3217.233

Spot N. 170 (RLPA_ECOLI)

m/z

778.346

814.460

858.497

960.578

1003.533

1030.560

1042.563

1062.554

1240.531

1246.538

1276.628

1288.633

1319.698

1666.817

1678.812

1913.995

1926.042

1945.065

1961.034

1966.955

1973.078

2656.540

2670.572

3483.352

Spot N. 171 (RLPA_ECOLI)

m/z

778.373

814.466

858.507

960.576

1003.544

1030.567

1062.555

1276.625

1288.629

1319.691

1666.799

1791.743

1851.938

1926.032

1945.061

1961.036

1966.954

2670.561

Spot N. 183 (SURA_ECO57)

m/z

772.463

813.434

825.435

954.605

957.581

966.592

976.550

988.558

1104.630

1125.634

1139.609

1156.628

1227.771

1250.594

1262.598

1462.731

1474.735

1530.867

1546.859

1598.722

1628.886

1686.971

1687.851

1708.918

1726.828

1738.830

1742.832

1810.034

1822.020

1849.045

1861.049

1865.051

1877.055

2181.134

2182.168

2197.145

2213.135

2421.353

2481.378

2995.407

Spot N. 192 (YBHC_ECOLI)

m/z

1012.465

1028.464

1123.543

1254.635

1351.698

1498.802

1574.773

1590.770

1600.843

1601.846

1611.807

1622.768

1623.805

1755.922

1767.930

1938.014

1965.028

Spot N. 193 (EFTU1_ECO24, YBHC_ECOLI)

m/z

837.504

1012.437

1027.594

1123.519

1254.605

1351.666

1567.769

1574.827

1600.801

1601.770

1611.771

1617.776

1623.764

1631.783

1728.819

1741.887

1755.881

1767.857

1780.920

1795.930

1803.876

1937.969

1962.022

1964.967

2117.176

2238.039

Spot N. 194 (AGP_ECOLI)

m/z

1190.609

1302.693

1346.785

1350.719

1368.746

1380.747

1498.775

1511.745

1683.816

1684.811

1924.992

1925.990

1937.013

1938.006

1951.973

1986.026

1990.998

2007.010

2125.019

2139.032

Spot N. 195 (AGP_ECOLI)

m/z

791.410

1190.637

1302.715

1346.812

1368.774

1450.825

1472.835

1498.806

1511.778

1527.775

1613.846

1683.850

1684.848

1925.030

1937.035

1952.011

1969.031

1986.068

1991.044

2007.034

2008.070

2023.034

2024.081

2125.055

2139.099

2374.207

2785.657

Spot N. 196 (TOLB_ECO24)

m/z

804.483

1025.564

1142.661

1412.894

1698.871

1838.036

1955.091

Spot N. 199 (TOLB_ECO24)

m/z

761.427

804.488

980.551

1025.602

1026.576

1142.644

1147.633

1154.651

1213.681

1284.673

1412.879

1413.851

1453.732

1684.844

1698.859

1710.868

1799.025

1838.020

1854.028

1936.914

1955.096

1956.106

1966.115

1967.121

1977.072

3298.674

3330.608

Spot N. 202 (TOLB_ECO24)

m/z

761.425

804.449

980.526

1025.564

1026.554

1142.609

1147.611

1159.618

1284.613

1412.846

1413.808

1453.699

1684.803

1698.846

1710.845

1733.890

1798.962

1837.990

1849.997

1854.001

1936.951

1955.082

1967.101

3298.633

3310.789

Spot N. 205 (TOLB_ECO24)

m/z

804.464

1142.589

1147.611

1412.818

1698.797

1955.024

Spot N. 210 (DEGP_ECO57)

m/z

785.481

1283.668

1339.660

1475.741

1496.703

1652.912

1817.904

2017.072

2240.050

Spot N. 211 (DEGP_ECO57)

m/z

785.514

1339.696

1475.723

1496.654

1652.897

1817.870

2017.042

2240.011

Spot N. 225 (MALE_ECO57, GLPQ_ECOLI, PDXB_ECOBW)

m/z

820.433

959.528

971.503

1083.630

1089.598

1113.618

1189.713

1267.630

1279.653

1283.632

1290.635

1299.623

1336.646

1337.761

1348.645

1359.707

1380.681

1384.656

1440.734

1457.699

1474.729

1486.733

1490.730

1502.734

1571.809

1583.816

1593.810

1656.777

1670.796

1723.811

1766.872

1788.842

1856.910

1888.892

1897.848

2079.059

2097.029

2110.006

2119.004

2139.146

2161.123

2213.085

2229.155

2245.088

2301.145

2356.214

2490.291

3090.935

3650.428

Spot N. 226 (MALE_ECO57, GLPQ_ECOLI)

m/z

820.465

959.565

971.569

1057.648

1058.650

1083.677

1089.641

1095.647

1189.772

1211.691

1236.638

1267.700

1279.704

1290.712

1299.700

1336.718

1348.728

1349.767

1359.755

1380.752

1381.716

1384.742

1423.811

1440.810

1457.773

1474.812

1486.810

1490.813

1502.811

1571.902

1583.893

1599.848

1656.864

1670.883

1682.871

1723.909

1735.911

1766.978

1788.941

1856.994

1897.929

1909.923

2029.042

2047.170

2071.033

2079.152

2097.141

2103.034

2110.125

2119.118

2126.134

2139.274

2158.124

2161.233

2213.254

2229.256

2245.256

2301.312

2323.281

2356.391

2490.445

2698.585

2975.859

3090.144

3618.674

3650.721

Spot N. 227 (MALE_ECO57)

m/z

959.576

1267.715

1299.663

1336.692

1474.816

1571.883

1766.868

2213.211

Spot N. 242 (YNCE_ECOLI)

m/z

989.440

1072.551

1224.567

1236.565

1399.595

1487.652

1554.763

1865.823

1871.821

2204.138

Spot N. 244 (BAMB_ECOLI)

m/z

849.470

1011.561

1135.625

1277.676

1531.832

1534.798

1546.810

1547.859

1690.863

1803.879

1815.894

1864.939

1896.937

1903.957

1919.967

1935.960

1946.014

1951.952

1953.002

2157.123

2179.114

2326.203

2358.169

2666.294

2877.451

3146.726

3329.813

3361.811

3629.165

3630.169

Spot N. 245 (OMPF_ECOLI)

m/z

822.385

955.453

1003.457

1021.485

1131.547

1249.484

1261.513

1369.608

1745.764

1746.710

1762.749

1847.842

2132.922

2203.026

2544.270

2768.351

2773.546

2909.854

2925.553

3554.576

Spot N. 246 (BAMC_ECOLI)

m/z

895.474

1029.569

1102.595

1134.576

1249.575

1708.854

1845.066

1905.915

2012.042

Spot N. 247 (BAMC_ECOLI)

m/z

895.483

1029.562

1034.600

1102.619

1134.609

1189.609

1280.642

1639.950

1658.826

1670.943

1671.952

1682.918

1691.851

1700.918

1708.879

1711.742

1720.889

1808.911

1845.119

1905.901

1937.076

1973.047

2012.082

2028.089

2239.037

2278.118

2290.109

2294.119

2333.214

2829.355

2845.353

2939.699

2954.379

Spot N. 248 (BAMC_ECOLI)

m/z

895.454

1102.534

1134.513

1280.586

1708.751

1844.988

1905.927

2011.980

Spot N. 249 (EFEO_ECO57)

m/z

764.380

791.435

1134.610

1322.846

1960.988

1990.000

Spot N. 263 (MALM_ECOLI)

m/z

845.453

955.563

1212.756

1470.801

1506.868

1517.834

1518.825

1959.910

1975.907

1981.889

1997.855

2087.995

2109.970

2664.430

Spot N. 264 (PROX_ECO57)

m/z

966.479

1611.805

1808.913

2533.253

Spot N. 266 (NLPI_ECO57)

m/z

947.436

969.471

1092.556

1619.748

1692.762

1776.785

2204.116

2252.890

2332.211

2496.086

Spot N. 267 (FADL_ECO57)

m/z

786.384

1122.494

1134.502

1497.689

1528.688

1569.701

1587.719

1599.724

1652.801

1998.928

2030.864

2359.103

Spot N. 268 (PA1_ECO57)

m/z

993.469

1343.696

1377.700

1493.558

1495.621

1604.790

1632.777

1649.800

1825.865

1857.849

2631.188

Spot N. 272 (BLAT_ECOLX)

m/z

1086.458

1242.674

1275.660

1286.779

1307.570

1351.744

1414.837

1783.891

1832.946

2178.120

Spot N. 273 (CPOB_ECOLI)

m/z

1035.548

1348.705

1476.801

1526.809

1588.818

1769.940

1785.940

1825.003

1865.943

1946.051

2109.100

2300.185

2315.239

Spot N. 274 (BLAT_ECOLX)

m/z

974.464

1029.533

1041.540

1086.584

1101.672

1142.724

1201.640

1239.599

1242.679

1275.716

1286.809

1297.706

1298.786

1307.737

1351.800

1363.797

1376.712

1379.732

1414.891

1442.887

1701.854

1711.770

1740.846

1756.842

1783.913

1799.916

1832.936

2021.952

2043.991

2076.015

2083.085

2096.041

2115.096

2131.098

2178.087

2245.076

Spot N. 275 (BLAT_ECOLX)

m/z

1201.562

1275.626

1286.712

1307.618

1351.719

1414.857

1783.818

2177.973

Spot N. 284 (YBIS_ECO57)

m/z

806.369

828.423

1205.654

1292.777

1459.800

1716.970

1989.032

2511.364

2709.497

3114.737

Spot N. 286 (FKBA_ECO57)

m/z

805.422

1031.471

1065.598

1199.754

1235.587

1307.717

1638.884

1715.854

1877.952

1889.955

2021.092

2093.060

2171.157

2203.135

2673.508

Spot N. 293 (GLTI_ECOLI)

m/z

950.538

1041.608

1511.832

1609.804

1767.968

1851.977

1877.978

Spot N. 301 (NLPE_ECOLI)

m/z

1107.570

1218.727

1293.730

1309.728

1587.824

2148.058

2425.204

2457.199

2917.335

3151.316

Spot N. 302 (TSX_ECO57)

m/z

1463.697

1882.918

2162.086

2263.920

2539.237

2825.183

2857.182

Spot N. 305 (TSX_ECO57)

m/z

1463.554

1541.566

1591.633

1843.789

1875.795

1882.799

1894.816

1898.825

1914.812

1930.826

2161.982

2245.868

2249.190

2263.915

2275.904

2489.123

2539.191

2617.283

2825.159

2857.516

2868.445

3116.560

3148.502

Spot N. 306 (MIPA_ECOLI)

m/z

945.406

1559.859

1581.833

1629.801

2605.137

2612.278

2628.276

2644.282

2660.281

2724.299

2756.287

3366.661

3399.561

Spot N. 307 (MIPA_ECOLI)

m/z

945.432

1047.589

1144.603

1559.908

1581.777

1629.887

2197.038

2213.016

2605.018

2612.172

2628.156

2644.161

2660.174

2724.165

2736.139

2756.161

3366.474

3398.426

Spot N. 308 (BAMD_ECO57)

m/z

888.474

1292.685

1367.782

1371.786

1572.752

1601.817

1884.968

Spot N. 312 (LOIP_ECOLI)

m/z

1078.585

1216.690

1277.703

1391.741

1438.659

1452.719

1577.713

1687.776

Spot N. 313 (BAMD_ECO57)

m/z

872.401

888.437

890.550

900.435

918.536

971.546

1066.493

1081.531

1194.565

1268.674

1292.645

1308.652

1353.720

1367.745

1371.744

1572.693

1601.769

1751.828

1783.979

1824.894

1840.892

1863.878

1875.869

1879.879

1884.908

1895.872

1896.893

1912.898

2042.954

2064.952

2087.980

2108.972

2127.012

2143.004

Spot N. 315 (LPOB_ECO57)

m/z

1423.696

1499.742

1547.774

2088.013

2104.008

Spot N. 318 (ARTJ_ECOLI)

m/z

766.480

1256.569

1566.717

1694.808

1706.844

1710.790

1762.863

1763.818

2148.128

Spot N. 324 (MLAC_ECOLI)

m/z

864.408

896.389

974.431

1116.528

1125.586

1128.531

1148.517

1345.652

1433.747

1644.821

Spot N. 327 (QSEG_ECO57)

m/z

778.435

934.553

1230.671

1379.691

1411.692

1452.739

1470.754

1754.920

1859.025

Spot N. 333 (LOLB_ECOL6)

m/z

1227.678

1312.661

1882.046

Spot N. 341 (LPTE_ECO57)

m/z

800.484

928.514

1368.634

1487.820

1570.862

1835.989

Spot N. 364 (YAJG_ECOL6)

m/z

1118.608

1217.598

1229.600

1399.684

1485.628

1502.789

1573.797

1601.791

1666.941

1705.869

1721.867

1733.867

Spot N. 365 (SLP_ECOLI)

m/z

1295.634

1970.960

2213.164

Spot N. 369 (YRAP_ECO57)

m/z

774.411

993.562

1027.602

1183.693

1475.749

1553.879

1575.877

1662.901

1839.915

1855.987

1861.877

1878.899

1894.951

1910.964

2016.007

2038.992

2078.033

2094.051

2184.107

2223.131

2239.134

2410.240

2738.428

2916.602
